# Abnormal local markers of sleep homeostasis in patients with focal epilepsy

**DOI:** 10.1093/braincomms/fcag294

**Published:** 2026-07-31

**Authors:** Rosario Ciliento, Brinda Sevak, Cynthia Papantonatos, Zhixin Wang, Benjamin Jones, Ruben Verhagen, Elsa Juan, Kyle Shoger, Akshayaa Lakshmanan, Dillon Scott, Keith Nakamura, Graham Findlay, Tom Bugnon, Mariel Kalkach Aparicio, Beril Mat, Rama Maganti, Aaron F Struck, Giulio Tononi, Melanie Boly

**Affiliations:** Department of Neurology, University of Wisconsin-Madison, Madison, WI 53792, USA; Department of Biomedical, Metabolic and Neural Sciences, University of Modena and Reggio Emilia, Modena 41125, Italy; Department of Neurology, University of Wisconsin-Madison, Madison, WI 53792, USA; Biomedical Engineering Graduate Group, University of California Davis, Davis, CA 95616, USA; Department of Neurology, University of Wisconsin-Madison, Madison, WI 53792, USA; Saint Louis University School of Medicine, St. Louis, MO 63104, USA; Department of Neurology, University of Wisconsin-Madison, Madison, WI 53792, USA; Department of Neurology, University of Wisconsin-Madison, Madison, WI 53792, USA; Department of Neurology, University of Wisconsin-Madison, Madison, WI 53792, USA; Department of Philosophy, Vrije Universiteit Amsterdam, Amsterdam 1081 HV, Netherlands; Department of Neurology, University of Wisconsin-Madison, Madison, WI 53792, USA; Department of Neurology, University of Wisconsin-Madison, Madison, WI 53792, USA; Department of Neurology, University of Wisconsin-Madison, Madison, WI 53792, USA; Department of Neurology, University of Wisconsin-Madison, Madison, WI 53792, USA; Department of Neurology, University of Wisconsin-Madison, Madison, WI 53792, USA; Department of Psychiatry, University of Wisconsin-Madison, Madison, WI 53719, USA; Department of Neurology, University of Wisconsin-Madison, Madison, WI 53792, USA; Department of Neurology, University of Wisconsin-Madison, Madison, WI 53792, USA; University of South Dakota, Sanford Medical Center, Vermillion, SD 57105, USA; Department of Psychiatry, University of Wisconsin-Madison, Madison, WI 53719, USA; Department of Neurology, University of Wisconsin-Madison, Madison, WI 53792, USA; Department of Neurology, University of Wisconsin-Madison, Madison, WI 53792, USA; Department of Neurology, William S. Middleton Veterans Administration Hospital, Madison, WI 53705, USA; Department of Psychiatry, University of Wisconsin-Madison, Madison, WI 53719, USA; Department of Neurology, University of Wisconsin-Madison, Madison, WI 53792, USA

**Keywords:** sleep homeostasis, focal epilepsy, high-density EEG, slow-wave activity, epileptiform discharges

## Abstract

Given the importance of sleep for the regulation of cortical excitability, altered sleep homeostasis may contribute to the development and persistence of focal epilepsy. The goal of the present study was to quantify high-density EEG markers of sleep homeostasis in patients with focal epilepsy compared to healthy age- and gender-matched controls. In particular, we investigated the ability of sleep homeostasis markers to differentiate focal epilepsy versus healthy controls and quantified the deleterious effects of nocturnal epileptiform discharges on sleep homeostasis. Thirty-nine patients with focal epilepsy and 48 healthy controls underwent overnight 256-electrode high-density EEG recordings. Statistical analysis tested for differences in overnight slow-wave activity (i.e. delta power, 1–4 Hz, and theta power, 4–8 Hz) and average slope of slow waves between patients and healthy controls. We assessed the predictive value of sleep homeostasis parameters to identify focal epilepsy at the individual level. Finally, we investigated the relationship between the frequency of epileptiform discharges and abnormal overnight declines in sleep slow-wave activity and slow-wave slope. Patients with focal epilepsy displayed higher slow-wave activity and a steeper average slope of slow wave compared to healthy controls, exhibiting slow-wave activity values at the end of the night similar to those observed in healthy controls at the beginning of the night. Among sleep homeostasis markers, the presence of high local extremes in the topographical distribution of theta values proved to be discriminative at the single-subject level for distinguishing patients from healthy controls (area under the curve = 0.84). A significant negative association was observed between left-lateralized nocturnal epileptiform discharges and the overnight decline in slow-wave activity and slow wave slope, with the most pronounced effects over the left fronto-temporal leads. Local abnormalities in sleep homeostasis may constitute a reliable biomarker for focal epilepsy. Further studies are needed to determine whether it could also be used to identify focal epilepsy early in the disease course. Furthermore, frequent epileptiform discharges during sleep may contribute to abnormalities in sleep homeostasis and thus to the persistence of cortical hyperexcitability in focal epilepsy.

## Teaser

Ciliento *et al*. report that patients with focal epilepsy exhibit locally increased theta power during non-REM sleep compared to healthy controls. Moreover, interictal spikes negatively correlate with markers of sleep homeostasis, including slow-wave slope. These findings reveal altered local regulation of sleep and potential electrophysiological markers of epilepsy.

## Graphical Abstract

**Graphical Abstract:**
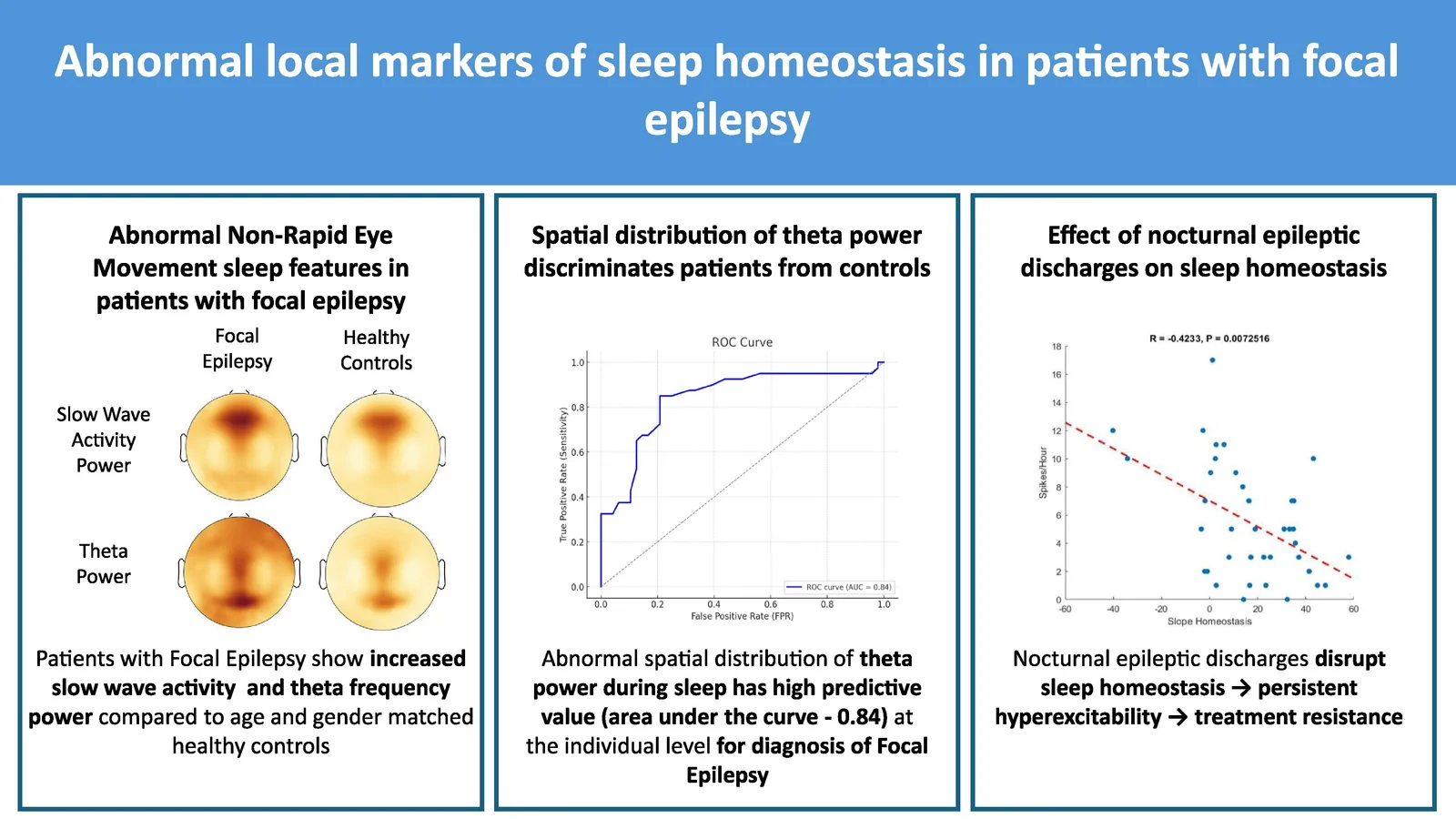

## Introduction

Epilepsy affects ∼6 cases per 1000 individuals, with an annual cumulative incidence of 67 cases per 100 000 worldwide.^1^ Notably, 30–40% of cases with focal epilepsy (FE) are drug-refractory (DRE).^2^ DRE is characterized by heightened neural excitability, as evidenced by the recurrence of seizures and frequent interictal epileptiform discharges (EDs) on scalp EEG.^3^ Animal studies suggest that both seizures and EDs induce synaptic potentiation and, in doing so, could contribute to mechanisms of epileptogenesis.^3,4^ However, the practical significance of recurrent seizures and EDs on the clinical progression of diverse forms of epilepsy in humans remains a subject of controversy. Consequently, additional research is warranted to elucidate cellular and network consequences of seizures and EDs in the human brain.^3^

The study of brain dynamics during sleep is a promising approach to investigate possible mechanisms by which epilepsy becomes refractory, assess lasting effects of ictal and interictal epileptiform activity on brain physiology and identify new epilepsy biomarkers. Slow-wave activity (SWA, i.e. delta power, 1–4 Hz, and theta power, 4–8 Hz) reflects the alternation between up states (increased firing) and down states (decreased firing) which appear as sleep slow waves on scalp EEG.^5,6^ While the largest of these slow waves correspond to K complexes, the vast majority of slow waves (both in the delta and theta frequency ranges) are small and localized: this second type of SW is homeostatically regulated and directly reflects local cortical synaptic strength.^7,8^ For example, local slow waves may increase in amplitude and slope in brain regions specifically engaged in tasks during preceding wakefulness.^9-12^ Initial results obtained using 256-electrode high-density EEG (HD-EEG) in 15 patients with epilepsy also suggested that sleep SWA may be focally increased at the epileptic focus, proportionally to the frequency of EDs present during preceding wakefulness.^13^ More widespread increases in SWA found in patients with epilepsy compared to healthy controls (HC) also correlated with the frequency of seizures with impaired awareness.^13^ While pointing to promising results, our previous small sample was insufficient to determine the potential diagnostic value of sleep markers for epilepsy at the individual level. Consistent increase in SWA during sleep in hyperconnected epileptic networks in patients with FE would be in line with recent evidence for the crucial role of sleep in synaptic plasticity. According to the synaptic homeostasis hypothesis,^14^ overall synaptic strength increases during wakefulness and decreases through off-line down-selection during NREM sleep. Recent electron microscopy research on mice has demonstrated that several markers of synaptic strength (axon-spine interface, head volume and synaptic size) decrease by about 20% during the night in a sleep-dependent manner, both in the neocortex and the hippocampus.^15^ Physiological widespread decreases in synaptic strength selectively during sleep were confirmed in a recent study in the zebrafish^16^ and shown to occur most prominently during the first part of the night. Pruning occurs most prominently among the weakest synapses, while stronger connections are preserved.^15^ This process may thus reinforce epileptic networks and favour persistent hyperexcitability towards the end of a night.^13^ In animal studies, bursting patterns of neuronal activity—which share electrophysiological features with EDs—have also been found to impede slow waves-dependent synaptic pruning.^17^ In line with this finding, we previously observed that overall SWA power decline was negatively correlated with the burden of nocturnal EDs in patients with epilepsy.^13^ Paediatric patients with encephalopathy related to electrical status epilepticus (ESES) during slow-wave sleep also display dampened decline in overnight slow waves slope, another marker of sleep homeostasis.^18^ In children with ESES, slow waves slope decline was reduced at the epileptic focus compared to contralateral cortices.^19^

The aim of the present study was to confirm and expand upon our previous findings to

- Further assess the diagnostic utility of markers of focal abnormalities in sleep homeostasis to diagnose FE
- Further investigate detrimental focal effects of EDs on overnight SWA decline, to further unveil possible mechanisms that may contribute to seizure refractoriness in many individuals with FE

## Materials and methods

### Patient population, standard protocol approvals, registrations and patient consents

Thirty-nine patients with FE were recruited from the Epilepsy Monitoring Unit (EMU) of the University of Wisconsin (mean age: 41 years, SD: 13.4, 20 females). The epileptic focus localized to either left temporal (*n* = 14), left frontal (*n* = 3), right temporal (*n* = 5), right frontal (*n* = 1), left extra- temporal (*n* = 1) right extra-temporal (*n* = 1), left fronto-temporal (*n* = 3), right fronto-temporal (*n* = 1), bilateral temporal (*n* = 7), bilateral frontal (*n* = 2) or bilateral centro-parietal (*n* = 1) regions, according to the clinical evaluations. General inclusion criteria included being potential patients with epilepsy admitted to the EMU, age of ≥18 years and ≤75 and ability to provide written consent and to understand English. General exclusion criteria includes being unable to comply with the protocol, prisoners and lacking consent capacity. Forty-eight age- and sex-matched controls free of neurological or sleep disorders and medications were recruited from two previously approved protocols conducted at the University of Wisconsin-Madison. All controls had a single night of sleep study with HD-EEG at the Wisconsin Sleep Laboratory. The Health Science Institutional Review Board of the University of Wisconsin approved all study procedures, and all subjects provided written informed consent before participating in the study.

Full details of the source studies, inclusion/exclusion criteria, and recruitment procedures are provided in the Supplementary material section.

### Data acquisition

A 256-electrode HD-EEG net was placed on participants’ heads to perform overnight recordings. Electrode impedances were kept under 50 kΩ. HD-EEG acquisitions were performed using a Geodesic System 300 [Electrical Geodesics Inc. (EGI), Eugene, OR]. 500 Hz HD-EEG recordings were referenced to Cz, with an analogue hardware high-pass filter set at 0.1 Hz. As in previous work,^13^ patient data were acquired in the EMU and control data at the Wisconsin Sleep Centre.

### EEG pre-processing

We used an EEG pre-processing pipeline similar to the one described in previous work.^20^ Sleep stages were scored according to the AASM 2018 criteria using a reduced 10–20 montage derived from the 256-channel HD-EEG, consistent with standard clinical practice. Staging was first performed on the raw EEG by a trained research assistant and subsequently reviewed case by case by a certified sleep technologist. After scoring, only epochs of non-rapid eye movement (NREM) sleep stages N2 and N3 were extracted. HD-EEG data were filtered from 1 to 40 Hz and downsampled from 500 to 200 Hz. Semi-automated artefact rejection selected clean epochs and channels.^20,21^

EDs were extracted during artefact-free periods in the HD-EEG recordings of each individual patient after visual identification on HD-EEG re-referenced with 10–20 conventional EEG display confirmed by a board-certified epileptologist (M.B.). To avoid a spurious contribution from spikes-and-waves to sleep SWA power estimation, 6-s NREM sleep epochs containing EDs were discarded from SWA and theta power computation and slow waves analyses described below. Moreover, our analyses focused on NREM (N2–N3) interictal activity; ictal activity was not analysed. When seizures occurred, the ictal periods (and a short buffer) were excluded from all spectral and topographical analyses.

Power spectrum computation was performed using MATLAB pwelch with 6-s non-overlapping time windows. Analyses were performed on average-referenced EEG. Analysis focused on *Z*-scored frequency bands of interest was computed: SWA (1–4 Hz) and theta (4–8 Hz). Computing the discrete Fourier transform for 240 frequency bins from 1 to 40 Hz for all 256 channels, power of the specific band of interest was defined as the average of power spectrum frequency bins within the specific range of interest, for example, 1–4 Hz for SWA.

As described in previous work,^12,20,21^ we used in-house software to detect individual NREM sleep slow waves on average-referenced EEG data and compute their negative down-going slope. Topographical analyses were performed on whole-night SWA, defined as the average SWA across all NREM epochs (stages N2 and N3) of the night. To estimate their overnight decline, we measured delta and theta power and slow-wave slope during the first and last hour of the artefact-free N2-N3 recordings and calculated the difference between these periods. If the pre-processed N2-N3 segments were shorter than 2 h, we divided the data into two equal parts and measured the features in the initial and final halves, as done in Boly *et al*.^13^

### Statistical analysis

Statistical analyses were computed using both statistical parametric mapping (SPM, http://www.fil.ion.ucl.ac.uk/spm) and statistical non-parametric mapping (SNPM, https://www.nisox.org/Software/SnPM13/).^22^ Topographical values of absolute and relative delta and theta power as well as their overnight decline and the average slow-wave slope and its overnight decrease were converted to 64×64 2D images. All statistical analyses used random-effects models. Given our *a priori* hypothesis, separate one-tailed two-sample *t*-tests assessed for increased overnight SWA power and average slow-wave slope and decreased overnight decline in SWA and slow-wave slope in patients with FE compared to HC. As in previous work, results were thresholded at *P* < 0.05 corrected for multiple comparisons using family-wise error at the voxel- or cluster-level (with uncorrected parametric *P* = 0.05 as cluster forming threshold).^13^ To compare the local differences in sleep-related features between FE and HC, we manually selected three standard coordinates representing distinct brain areas: midline (*x* = 0, *y* = 18.25), left temporal (*x* = −51, *y* = 18.25) and right temporal (*x* = 51, *y* = 18.25). To assess whether focal sleep homeostasis markers could discriminate patients with FE from HC at the individual level, we adopted a subject-wise case-versus-reference approach based on spatial theta power maps.^23^ For each subject, theta power was first represented as a spatial map with one value per electrode. The theta power map of a given subject was then compared one by one against the theta power maps of all HC, yielding a set of spatial difference maps. These difference maps were entered into an electrode-wise *t*-test implemented in SPM, resulting in a subject-specific t-map that quantified spatial deviations of theta power relative to the control group. From each subject-specific t-map, a single summary statistic was extracted, namely, the maximum positive *t*-value, reflecting the strongest focal increase in theta power compared to controls. Repeating this procedure for all subjects resulted in one *t*-value per individual. An analogous leave-one-out procedure was applied to HC to obtain a corresponding distribution of control *t*-values. These subject-level *t*-values were subsequently standardized (*Z*-scored) to place all individuals on a common scale and to reduce the influence of extreme values. Because theta power yielded clearer group-level separation than delta power, we only report the latter analysis in the present manuscript. Signal detection theory^24^ was applied to the distributions of *t*-values from controls and patients to analyse differences in distributions between the groups. As the classification threshold, we used a value by which the likelihood of detecting epilepsy and the likelihood of falsely identifying it as present was equal (identified 50% of the time as HC and 50% of the time as DRE). We also constructed a receiver operating characteristic (ROC) curve to evaluate the predictive capability of our diagnostic measure concerning epilepsy. This maximum *t*-value was the diagnostic measure used for ROC analysis. This curve delineates the balance between sensitivity (the true positive rate) and 1 − specificity (the false positive rate) across a spectrum of threshold values.^25^ Sensitivity measures the ability to accurately detect FE, while 1 − specificity indicates the probability of mistakenly classifying HC cases as FE. Our ROC curve was generated using MATLAB's built-in functions, facilitating systematic computation of sensitivity and 1 − specificity across the threshold range. Each point on the ROC curve represents the diagnostic measure's performance at distinct threshold settings. The area under the curve (AUC) offers a quantitative assessment of diagnostic accuracy, with an AUC of 0.5 denoting chance performance and an AUC of 1.0 indicating perfect discrimination. To further assess classification performance and ensure generalizability, we implemented 5-fold cross-validation using logistic regression. The dataset was split into five subsets, with each subset serving as an independent test set while the remaining four were used for training. Model performance was evaluated using accuracy, AUC, sensitivity and specificity across all folds. To explore potential non-linearity in classification, we also trained a support vector machine with an radial basis function (RBF) kernel, using the same cross-validation procedure.

Finally, multiple regression random-effect models were employed to examine group-level negative correlations between delta power, theta power, slow-wave slope and their decline with total ED frequency (in spikes/h), which was included as a single covariate or with left versus right EDs included as different covariates. The ED frequency in our population varied from 0 to 2082 spikes/h. To mitigate the influence of outliers, a square root transform was applied to ED frequency when using it in the analysis.

## Results

### Group-level results: abnormal local markers of non-rapid eye movement sleep homeostasis in patients with focal epilepsy

**Figure 1:**
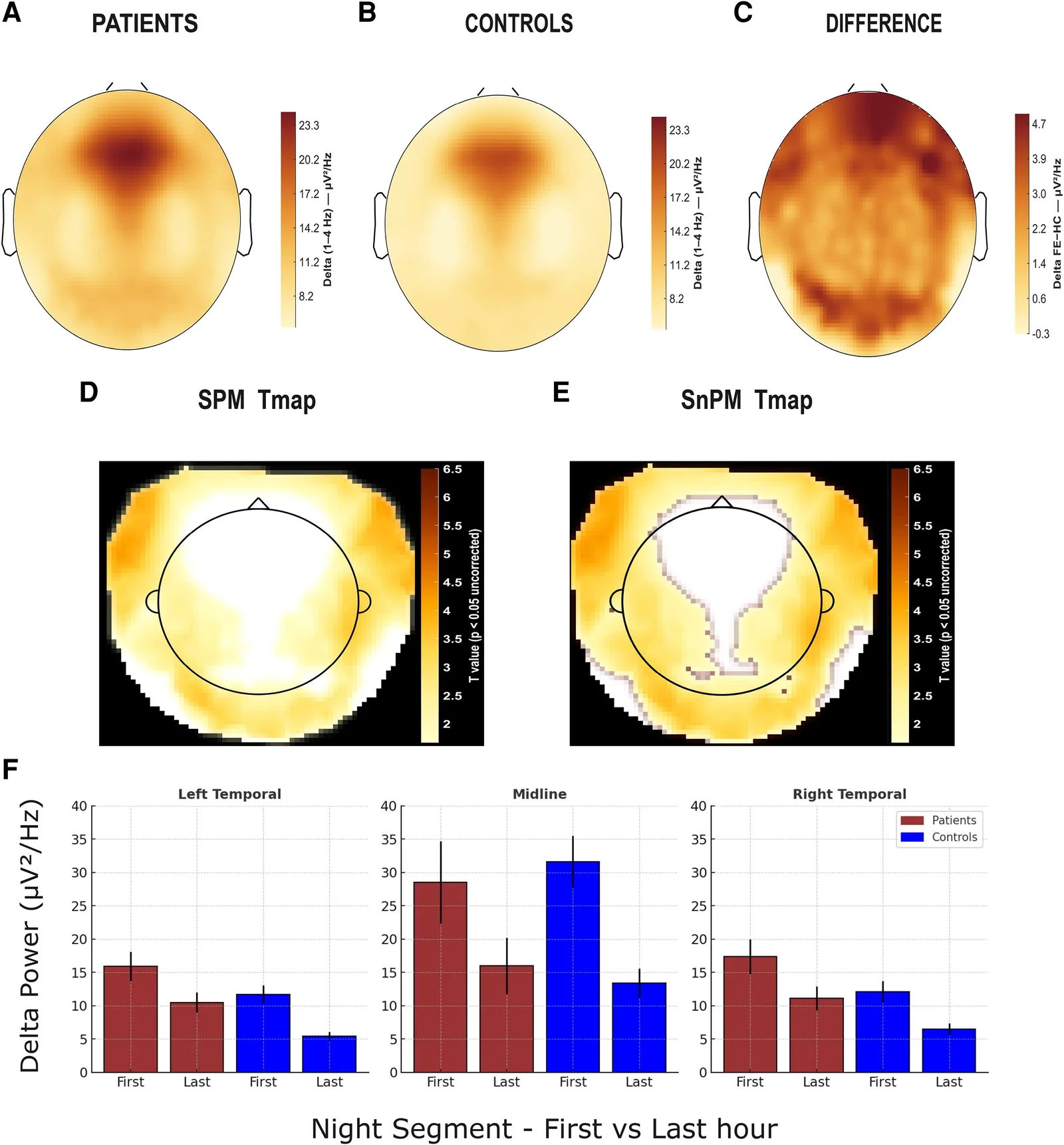
**Delta power topographies and group-level comparison for average-referenced EEG in controls and patients.** (**A**) Mean delta power in patients with FE. (**B**) Mean delta power in HC. For transparency, representative topographical maps from all individual subjects are provided in the Supplementary material, showing delta power distributions for both patients and controls. (**C**) Difference in delta power between patients and controls. (**D**) SPM T map for this difference, thresholded for display at uncorrected *P* < 0.05. (**E**) SnPM T map for this difference, thresholded for display at uncorrected *P* < 0.05. Statistical analyses: group-level differences were assessed using SPM12 univariate *t*-tests (*N* = 39 FE, *N* = 48 HC) with SnPM permutation tests for non-parametric validation. (**F**) Bar plots showing NREM sleep delta power values (mean ± SEM) in the first and last part of the night in patients and controls over different regions of interest. Left to right: left temporal region, midline, right temporal region. In each plot, patients are shown in brown and correspond to the first two bars from the left; healthy controls are shown in blue and correspond to the last two bars. Patients with FE *N* = 39, HC *N* = 48. EEG, electroencephalography; FE, focal epilepsy; HC, healthy controls; NREM, non-rapid eye movement; SEM, standard error of the mean; SPM, statistical parametric mapping; SnPM, statistical non-parametric mapping.

**Figure 2:**
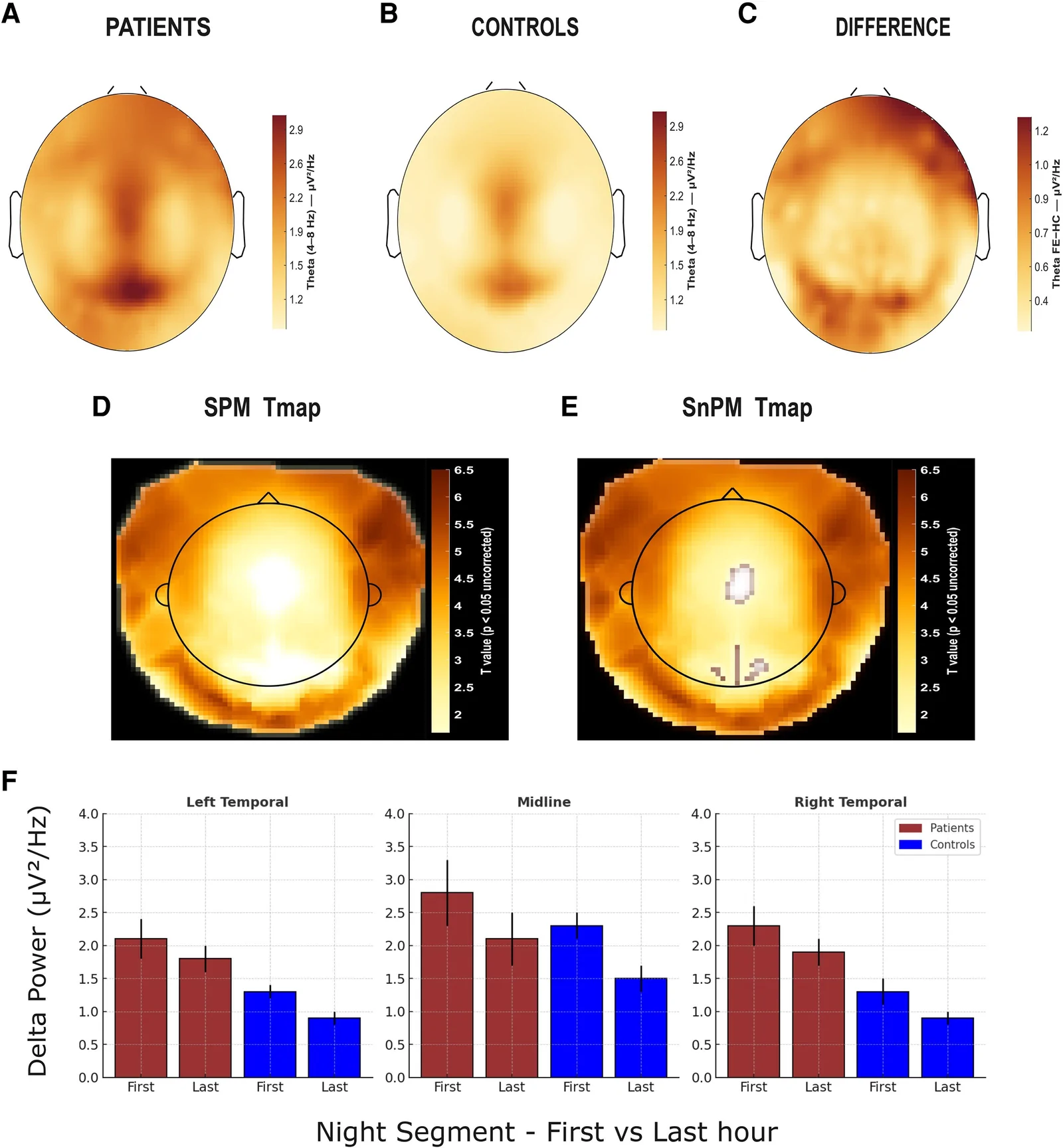
**Theta power topographies and group-level comparison for average-referenced EEG in controls and patients.** (**A**) Mean theta power in patients with FE. (**B**) Mean theta power in HC. For transparency, representative topographical maps from all individual subjects are provided in the Supplementary material, showing theta power distributions for both patients and controls. (**C**) Difference in theta power between patients and controls. (**D**) SPM T map for this difference, thresholded for display at uncorrected *P* < 0.05. (**E**) SnPM T map for this difference, thresholded for display at uncorrected *P* < 0.05. Statistical analyses: group-level differences were assessed using SPM12 univariate *t*-tests (*N* = 39 FE, *N* = 48 HC) with SnPM permutation tests for non-parametric validation. (**F**) Bar plots showing NREM sleep theta power values (mean ± SEM) in the first and last part of the night in patients and controls, over different regions of interest. Left to right: left temporal region, midline, right temporal region. In each plot, patients are shown in brown and correspond to the first two bars from the left; healthy controls (HCs) are shown in blue and correspond to the last two bars. Patients with FE *N* = 39, HC *N* = 48. EEG, electroencephalography; FE, focal epilepsy; HC, healthy controls; NREM, non-rapid eye movement; SEM, standard error of the mean; SPM, statistical parametric mapping; SnPM, statistical non-parametric mapping.

Compared to HC, patients with FE showed significantly increased delta and theta power during NREM sleep over a widespread set of fronto-temporal and parietal-occipital areas bilaterally (Figs. 1 and 2 top rows; for transparency, representative topographical maps from all individual subjects are provided in Supplementary Figs 2–175, showing delta and theta power distributions for both patients and controls.) However, no significant differences between HC and FE were found over the midline. On average, in patients with FE, EEG delta power increased by 37% over left temporal areas, 36% over right temporal areas and 14% at the midline. Theta power showed even larger average increases: 45% over left temporal areas, 48% over right temporal areas and 16% increase at the midline. No regions showed decreased SWA or theta power in patients with FE compared to HC. Peaks of significant differences in SWA, theta and slow-wave slope between HC and FE are reported in the Supplementary material (Supplementary Table 1).

Both patients with FE and HC showed a significant overnight decline of EEG SWA and slow-wave slope. On average, delta power declined 34% versus 48% over the left temporal areas, 37% versus 47% over the right temporal areas and 44% versus 60% over the midline. Patients also showed a decline in EEG theta power overnight, although to a lesser degree than HC: theta power declined by 15% versus 31% over the left temporal areas, 17% versus 31% over right temporal areas and 25% versus 35% at the midline. Overall, patients with FE showed a smaller mean overnight decline in sleep markers compared to HC, especially pronounced in bilateral temporal areas: EEG delta and theta power in patients with FE reached values at the end of the night that were comparable or higher than those found in HC at the beginning of their night (Figs. 1 and 2, bottom rows; Supplementary Table 2). However, this difference did not reach statistical significance Similar findings were observed for overnight SW slope decline.

### Diagnostic value of overnight theta power topography

**Figure 3:**
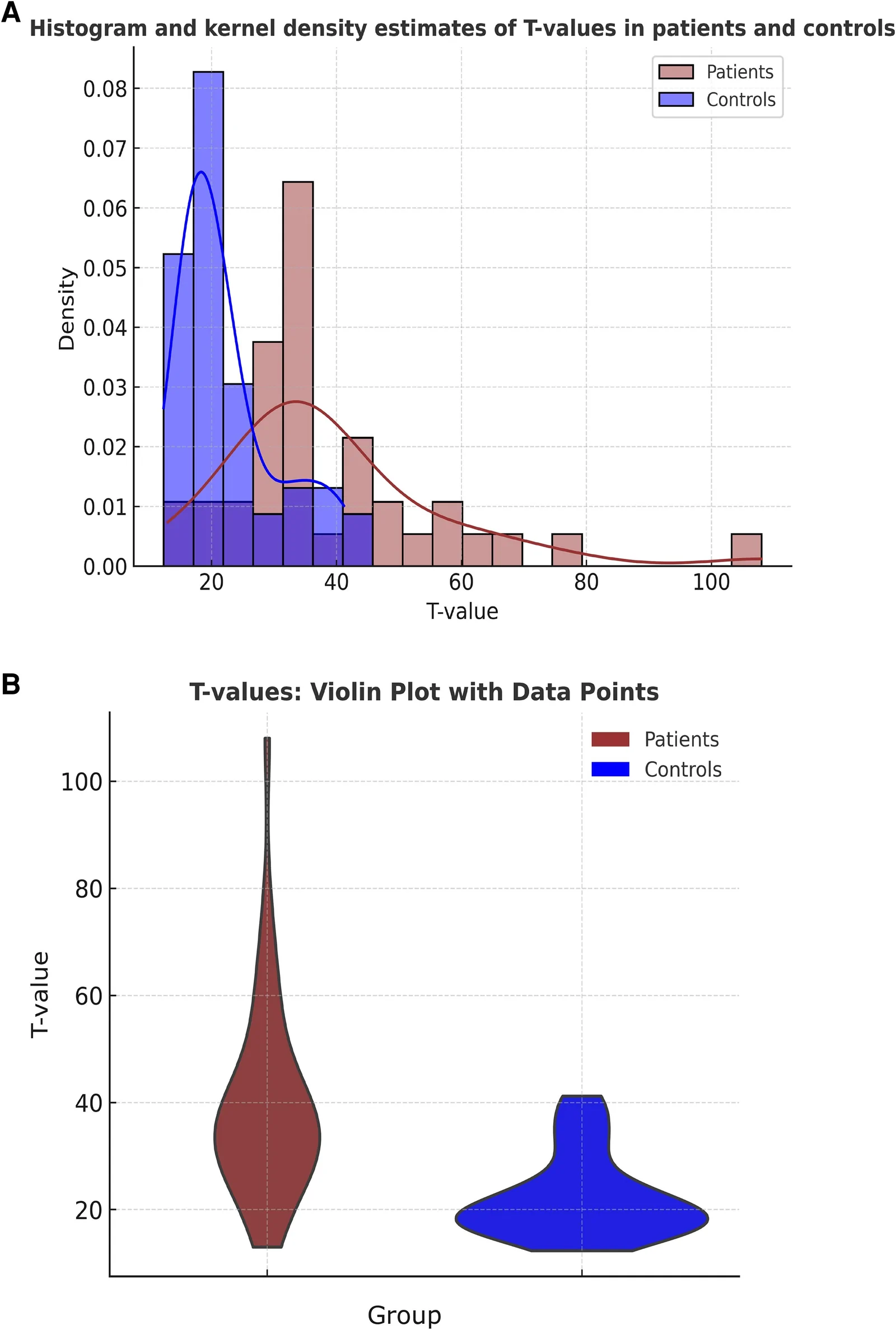
**Normalized overnight theta power. Distributions of *t*-values at the peaks for every single subject and every single controls.** Once we calculated theta power overnight in patients and controls, we subtracted those values for every single subject and all the controls, obtaining 40 different distributions of value and then we applied one-sample *t*-test for all of them. The shape and the values show a natural bimodal distribution that allows us to apply signal detection theory in order to assess the prediction power for diagnosis of epilepsy of the *t*-values for individual overnight theta power. (**A**) Histogram overlaid with KDE. It shows the distribution of *t*-values in patients with (maroon) and HC (blue). The distribution appears distinct but with some overlap, suggesting a potential classification boundary. Statistical analyses: subject-level distributions of *Z*-scored theta maxima were compared to controls using one-sample *t*-tests (*N* = 39 FE, *N* = 48 HC). (**B**) Violin plot of *t*-values for patients (on the left) and healthy controls (on the right). FE, focal epilepsy; HC, healthy controls; KDE, kernel density estimation.

**Figure 4:**
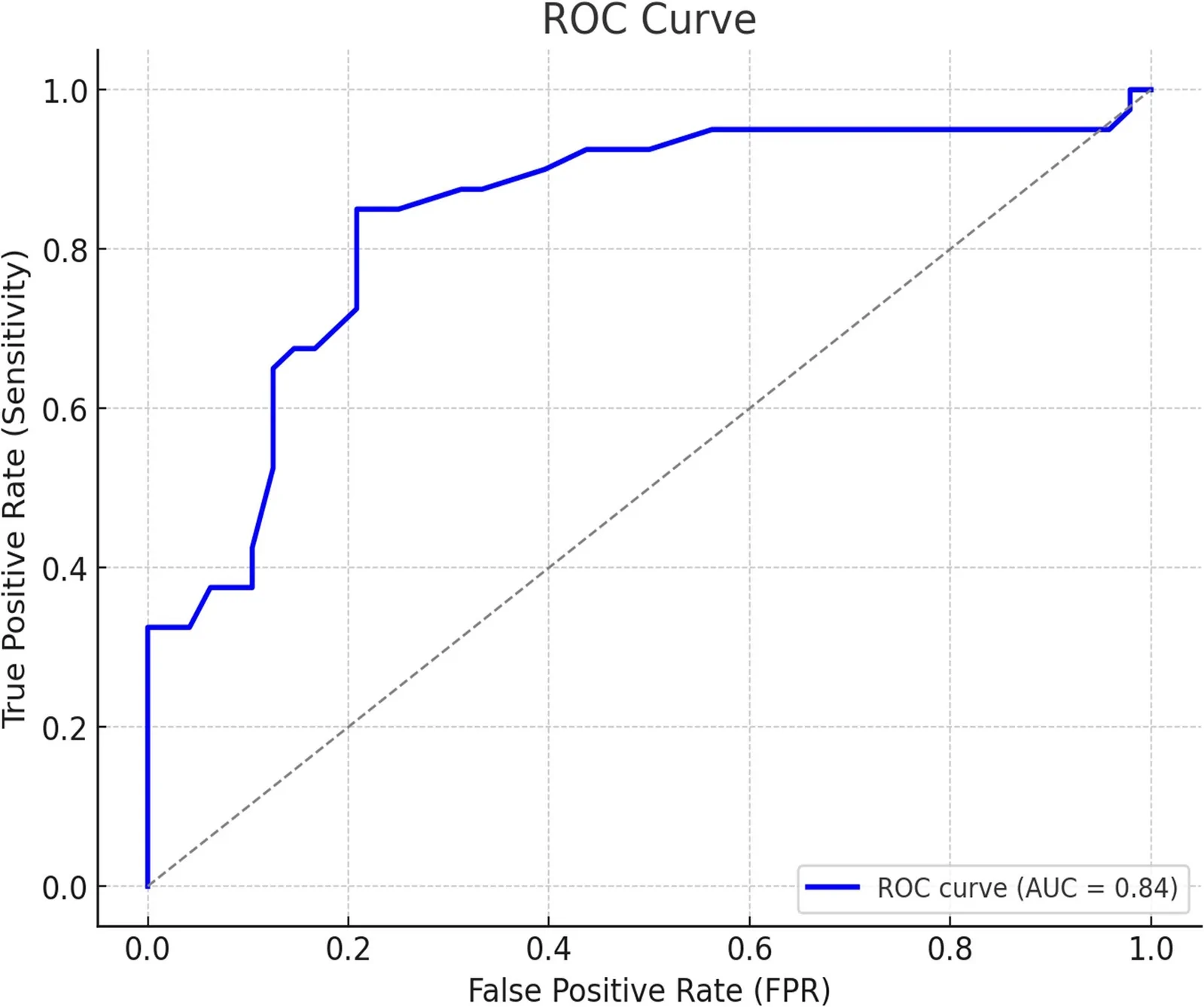
**ROC curve analysis of classification performance.** Classification performance was assessed using ROC analysis with permutation testing. Model generalizability was tested with 5-fold cross-validation using logistic regression and SVM classifiers (*N* = 39 FE, *N* = 48 HC). AUC, area under the curve; FE, focal epilepsy; HC, healthy controls; RBF, radial basis function; ROC, receiver operating characteristic; SVM, support vector machine.

At the individual level, patients with FE demonstrated significantly elevated *Z*-scored theta power values in brain regions corresponding to the peaks of theta power during NREM sleep, when compared to the respective regions of maximum *Z*-scored values observed in HC (mean T FE = 39.19 ± 17.8 versus mean T HC = 22.4 ± 7.7, *P* < 0.001). Normalized overnight theta values are provided in the Supplementary material (Supplementary Table 3). Examining Fig. 2, one can further see that the scalp distribution of *t*-values for sleep theta power in HC appears Gaussian, while among the participants with FE demonstrates some discrete higher values that are only found in this group. Finally, low theta power values were not specific to either patients with FE or HC. Applying signal detection theory, we observed a different distribution of peak *Z*-scored theta power values in FE versus HC. A *t*-value threshold of 32.7 separating the two distributions led to a measured *d*-prime value of 1.51, with response bias of −0.30 (specificity = 0.85, sensitivity 0.67, false alarm rate 0.14). The AUC for this distribution was calculated at 0.84 (Figs. 3 and 4).

To assess classification performance and generalizability, we further trained a logistic regression model and evaluated it using 5-fold cross-validation. The model achieved a mean accuracy of 77.19% ± 8.43%, with an AUC of 0.8157 ± 0.01394. The AUC values across the 5-folds were 0.944, 0.7375, 0.7531, 0.9857 and 0.6667, showing some variability but overall good performance. To determine whether a non-linear approach could improve classification, we also trained a support vector machine with an RBF kernel using the same cross-validation approach. The support vector machine resulted in a mean AUC of 0.8078 ± 0.1200, with fold-wise AUC values of 0.8750, 0.7250, 0.7531, 0.9857 and 0.7000. While the results were comparable, logistic regression performed slightly better on average and exhibited lower variability across folds, suggesting that a linear model is sufficient for classifying FE versus HC.

### Effect of nocturnal epileptiform discharge on sleep homeostasis

**Figure 5:**
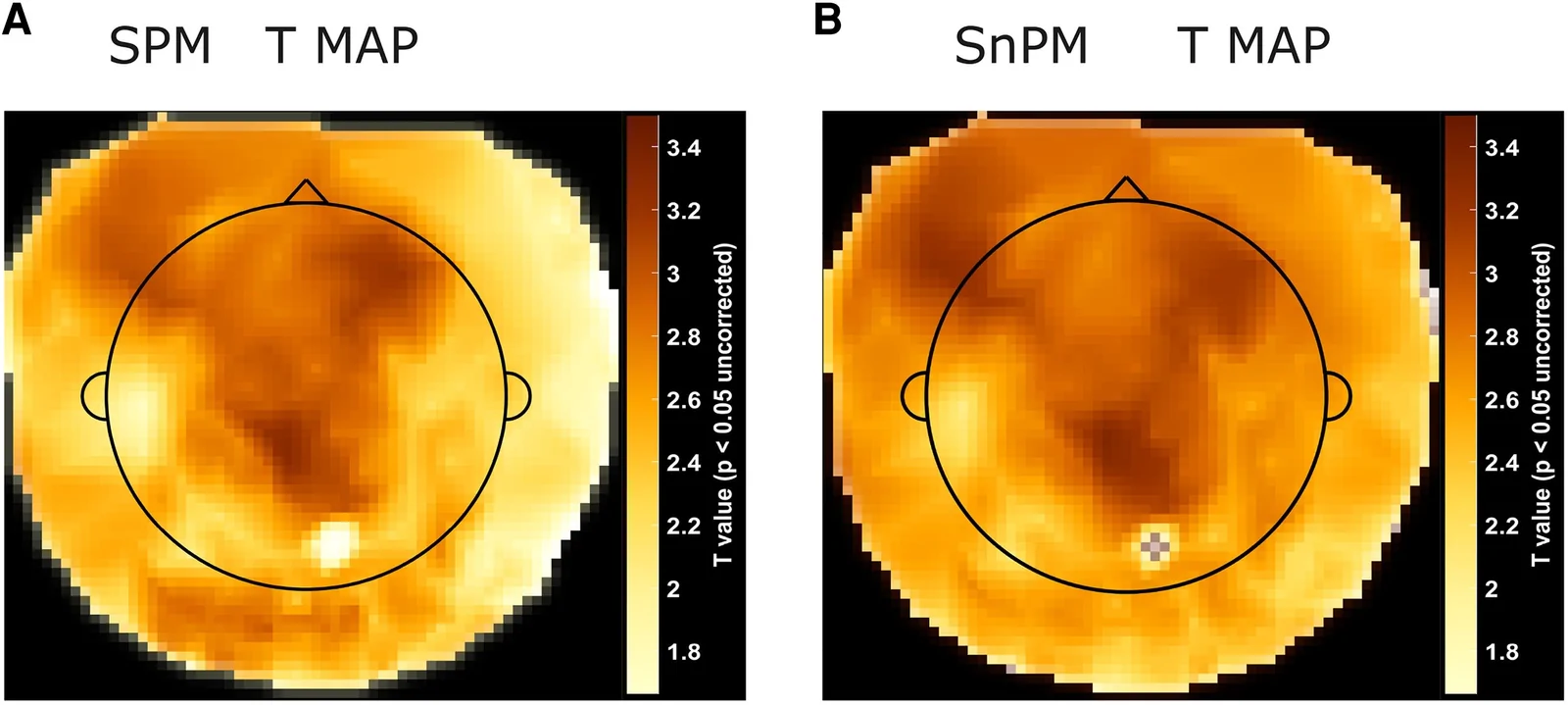
**Negative association between overnight average slow-wave slope decline and nocturnal spikes frequency.** (**A**) SPM T map of negative association between overnight average slow waves slope decline and nocturnal left side spikes frequency, thresholded for display at uncorrected *P* < 0.05. Correlations between slow-wave slope decline and epileptiform discharges’ rate were tested using random-effects multiple regression in SPM12 (*N* = 39 FE) (**B**) SNPM T map of negative association between overnight average slow-wave slope decline and nocturnal left side spikes frequency, thresholded for display at uncorrected *P* < 0.05. SnPM permutation tests for non-parametric validation (*N* = 39 FE). FE, focal epilepsy; SPM, statistical parametric mapping; SnPM, statistical non-parametric mapping.

**Figure 6:**
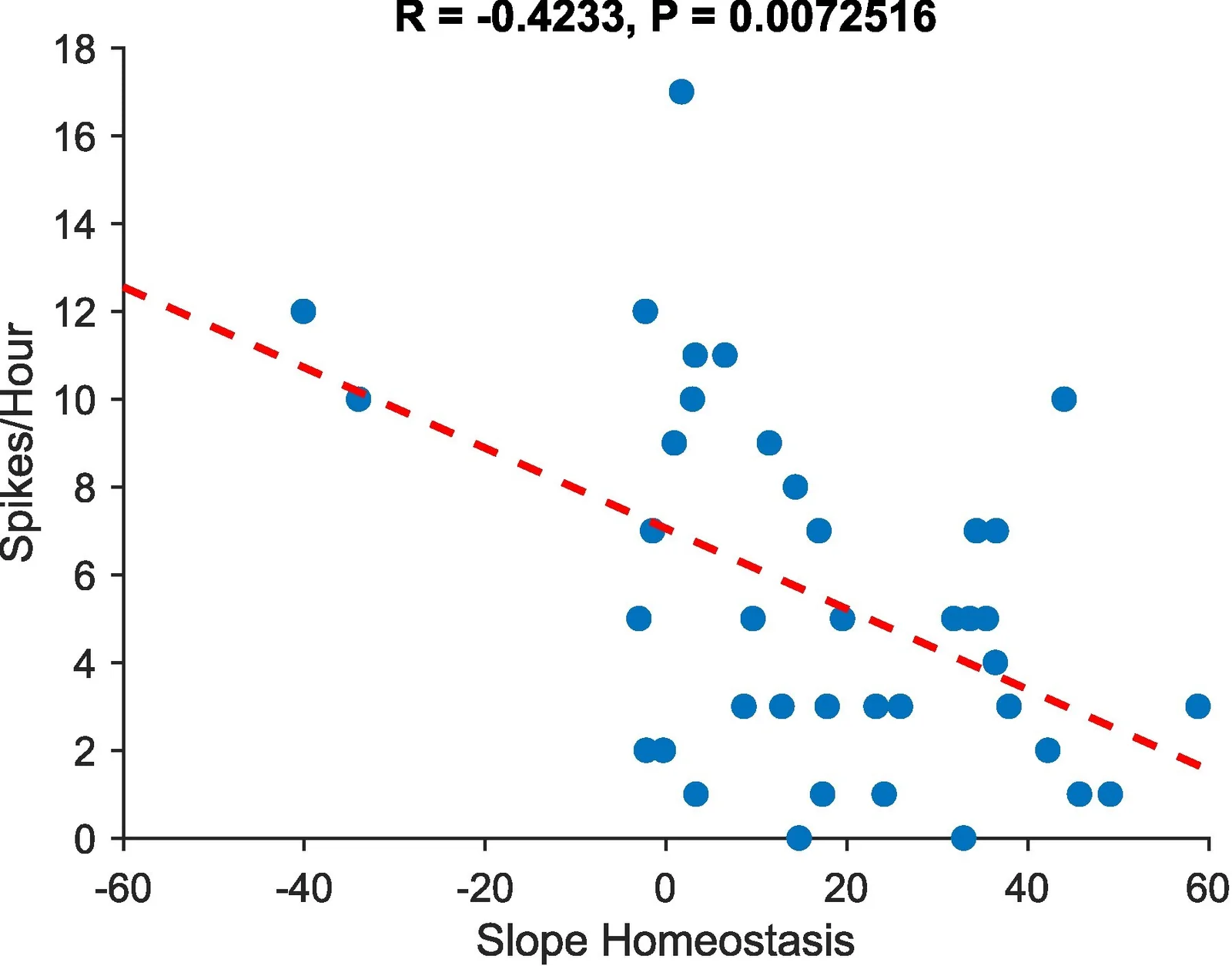
**Correlation between interictal spike rate and slope decline.** Scatterplot showing the relationship between interictal spike rate in the focus and slope decline in the corresponding region. The plot includes the linear fit and Pearson's correlation coefficient. Each data point represents one patient with FE (*N* = 39). Reported statistics: *r* = −0.4233, *P* = 0.0072516.

Topographical statistics showed a significant negative association between the overall frequency of EDs during NREM sleep and overnight slow-wave slope decline (Fig. 5), involving a widespread set of scalp areas bilaterally (*P* < 0.05). Similar findings were observed for overnight delta power decline, although less pronounced. We repeated this statistical analysis while further separating left and right EDs into different covariates SPM. As a result, only left-lateralized EDs (which were most prevalent in our cohort) showed statistically significant effects, with a significant widespread negative association with the average slope of slow waves involving bilateral scalp area, and maximum effect over left fronto-temporal regions. At the peak of significance, located on the left fronto-temporal area, we found a correlation coefficient of *r* = −0.422 with a *P*-value of 0.007 (Fig. 6). A negative association between left-lateralized EDs and slow-wave slope decline in left fronto-temporal was statistically significant also when assessed non-parametrically using SNPM (Fig. 5). A negative association was also found between left-lateralized ED frequency and overnight SWA decline in left fronto-central and fronto-temporal regions.

## Discussion

Using overnight HD-EEG recordings in 39 FE patients and 48 HC, we observed that FE is associated with widespread abnormalities of sleep homeostasis, with sleep SWA elevated at the beginning of the night and remaining abnormally high at the end of the night. At the regional level, patients showed temporal SWA values at the end of the night that were comparable to those seen in HC at sleep onset, underscoring both the global elevation and the insufficient overnight decline. Interestingly, focal abnormalities in SWA distribution (extremes in local maxima compared to the whole scalp) proved highly discriminative between patients with FE and controls, with an AUC of 0.84. Increased ED frequency during sleep predicted more dysfunctional sleep homeostasis with greater dampening of SWA decline, especially ipsilaterally. These results confirm and extend our initial findings of marked abnormal sleep homeostasis in patients with FE and open new avenues for validating biomarkers to assess and monitor FE severity based on interictal sleep SWA. Frequent EDs during nocturnal sleep may contribute to pharmacoresistance in patients with DRE by preventing decreases in cortical excitability enabled by normal sleep homeostasis. Finally, although more than one-third of our patients had a left temporal epileptogenic focus, our findings were not confined to this subgroup, but instead reflected bilateral temporal abnormalities of sleep homeostasis in FE.

### Quantitative impairment of sleep homeostasis in patients with focal epilepsy

In patients with FE compared to HC, group-level analyses revealed widespread increases in delta and theta power during NREM sleep. Such changes were especially marked across bilateral temporal and occipital regions, while no significant changes were observed in midline areas. Similar findings were observed when comparing the slope of sleep slow waves, shown to be a high reliable scalp marker of cortical excitability.^20,21^ While both patients with FE and HC exhibited a decline in sleep SWA and slow-wave slope over the course of the night, in patients such decline was insufficient to compensate for their overall increase at the beginning of the night. Indeed, in patients with FE, sleep delta power, theta power and average slow-wave slope were as high at the end of the night as they were at the beginning of the night in HC. Such findings confirm and extend our previous initial findings in 15 patients with FE in a larger cohort and continue to suggest the presence of widespread, bilateral increases in cortical excitability in the epileptic brain, extending beyond the seizure focus in patients with FE. In animal studies, sleep SWA has been shown to be a reliable marker of synaptic strength and to decrease in proportion to synaptic downscaling occurring during physiological sleep both in the hippocampus and neocortex.^15,26^ In humans, sleep SWA has been shown to track plastic changes related to learning occurring during preceding wakefulness.^10,27^ Our results suggest that increased sleep SWA in patients with FE reflects the hijacking of normal synaptic plasticity mechanisms by epileptic activity.^13,28^ Indeed, widespread increases in sleep SWA in patients with FE are in line with previous human slice work showing a 2.6-fold increase in synaptic density accompanied by elevated CREB expression (a marker of long-term potentiation) in layer two-third of epileptic neocortex compared to neighbouring cortex.^28-31^ Bilateral abnormalities in sleep SWA in our patient cohort may reflect potential multifocality for EDs and/or seizures frequently found in FE patient populations after a prolonged disease course.^32^ Insufficient synaptic downscaling during sleep, reflected by persistently higher SWA at the end of the night in patients with FE, may favour the persistence of widespread increases in cortical hyperexcitability and contribute to pharmacoresistance.

### Hyper-local increases in sleep SWA as candidate diagnostic biomarker for focal epilepsy

Studies of physiological sleep regulation suggest that SWA is prominently regulated at a local level.^33^ Recent HD EEG studies of human sleep SWA also suggest that smaller slow waves (‘type 2’) are especially subject to overnight decline.^8,9^ Given that increases in synaptic density in epileptic cortex most consistently localize to layers two-third, which are the source of most local connectivity in the neocortex,^30^ we hypothesized that sleep SWA increases in patients with FE compared to HC would be most marked in the theta frequency, capturing smaller sleep slow waves.^8,9^ Indeed, our results show that local peaks in the spatial distribution of HD-EEG sleep theta power were able to discriminate patients with FE from HC, achieving an AUC of 0.84 in an exploratory individual-level classification analysis. This accuracy is similar to that of other gold-standard imaging modalities for FE such as PET, SPECT or electrical source imaging of EDs.^34^ Because only individuals with FE exhibited highly focal overnight elevations in theta power, this feature may serve as a quantitative EEG marker with discriminative value at the individual level. However, this result should be interpreted as an exploratory, proof-of-concept finding. In particular, subject-level features were derived using the available control reference set rather than controls restricted to each training fold, which may have increased apparent separability and limits conclusions regarding out-of-sample generalizability. In addition, the classification threshold was derived from the same dataset and should therefore be considered an optimistic, retrospective estimate. Replication in independent datasets, implementation of fully training-set-restricted normalization procedures and direct comparison with ED-based diagnostic approaches will be essential before clinical application. Finally, while these results are promising, the classification performance of quantitative sleep SWA markers requires further evaluation in independent cohorts, including populations with other causes of EEG slowing (such as stroke or dementia, where lower rather than higher SWA overnight^35,36^) and in patients with the absence of classical EEG diagnostic findings for epileptic disease, such as interictal EDs.

### Deleterious effects of nocturnal epileptiform discharges on sleep homeostasis

Patients with FE exhibited a negative association between spike frequency and overnight SWA. Left-lateralized spikes, which were most frequent in our cohort, also showed focal deleterious effects, with a maximum negative correlation between spike frequency and the average slope of sleep slow waves localized to the left side (Figs. 5 and 6). Increased spike frequency was also associated with a lesser decline in the average slope of SWs overnight. These results suggest that the presence of EDs during sleep may worsen sleep homeostasis abnormalities by preventing normal sleep homeostasis processes that decrease local cortical excitability, further favouring seizure risk. These results are in line with our initial results obtained in a smaller cohort, where interictal spikes during NREM sleep were correlated with decreased overnight decline in slow-wave slope.^13^ They also fit with results obtained in children with electrical status epilepticus during sleep, in whom a correlation between the frequency of overnight EDs and dampened sleep slow-wave slope homeostasis was observed, which was most pronounced at the epileptic focus, while normal sleep homeostasis patterns re-emerged upon remission of the disease^18,19^ A negative effect of EDs on sleep homeostasis may be related to recent findings in animal studies that supra-threshold pre- and post-synaptic pairing during neuronal firing up-states, expressed as burst firing during sleep, prevents normal weakening of the synapses and may even strengthen them.^17,37^ Therefore, the occurrence of abnormal neural firing during NREM sleep may have detrimental effects on sleep homeostasis beyond the abnormal strengthening of epileptic networks that may occur due to seizures and/or EDs occurring during wake.^13,28,38^

## Limitations

All patients were receiving anti-seizure medications at the time of recording. Prior work has shown that medications can modulate EEG rhythms, for instance by enhancing slow activity or reducing the amplitude of NREM oscillations, while tapering is associated with reduced delta/theta power (Roebber *et al*., 2022; Besné *et al*., 2024; Limotai *et al*., 2020).^39-41^ Therefore, their effects may have contributed to the increased power observed in FE. Importantly, the retrospective nature of this EMU-based dataset precludes the availability of an ASM-free baseline. Nevertheless, given the heterogeneity of drug regimens and the bilateral distribution of our findings, it is unlikely that ASM effects alone fully account for the results. Future studies in medication-stratified cohorts or longitudinal designs will be required to more precisely disentangle disease-related alterations in sleep homeostasis from medication-related effects. We provide a detailed table of ASM regimens for each patient (Supplementary Table 4).

The classification analyses should also be viewed as exploratory. We used the maximum theta power as a summary feature, which provides a simple subject-level index of focal deviation. However, subject-level features were derived using the full control reference set rather than controls restricted to each training fold. As a result, the feature construction step is not fully independent from the test data in a strict machine learning sense and may influence estimates of separability. Replication in independent patient cohorts and implementation of training-set-restricted normalization procedures will be required before assessing clinical applicability.

## Conclusions and future directions

We used overnight HD-EEG in patients with FE undergoing EMU presurgical evaluation and age- and gender-matched HC to quantify abnormalities in sleep homeostasis. Sleep SWA (in delta and theta power) and average slope of slow waves were found to be consistently increased in patients with FE, with insufficient overnight decline to compensate for such increase. Hyper-local increases in sleep SWA (in the theta range) showed discriminative value for FE with an AUC of 0.84. Additionally, frequent EDs during sleep predicted further reduction of sleep overnight SWA and slow-wave slope decline. Altogether, these results strongly support a marked dysfunction in sleep homeostasis in patients with FE, which may both reflect maladaptive synaptic potentiation due to epileptic activity and contribute to some of the mechanisms underlying seizure refractoriness.

Our results may have diagnostic implications for identifying patients with FE in cases where EDs are absent or seizures are not captured during EMU monitoring. They may also help to localize candidate regions for the seizure focus.^42^ Further studies are needed to determine if sleep SWA patterns can be used to identify FE early in the disease course. Future clinical trials should further evaluate the effect of interventions that improve sleep quality (sleep hygiene, treatment of OSA and/or medications suppressing spikes) on sleep homeostasis and correlate sleep EEG changes to clinical outcomes. In the future, clinical trials may also attempt to improve local sleep homeostasis in patients with FE through neuromodulation techniques enhancing sleep rhythms.^43^ Further studies in patients with FE should also assess if focal sleep homeostasis findings predict surgical outcomes. Intracranial EEG studies may be used to further differentiate abnormalities in sleep homeostasis present in the seizure onset zone versus the neighbouring cortex involved in ED generation. It will also be important to investigate the temporal relationship between SWA dynamics and ED occurrence across the night, including their modulation by ultradian cycles and the possibility of phase-locking between interictal discharges and SWA. Finally, future studies may further assess changes in clinical outcomes after pharmacotherapy suppressing EDs in adult patients with frequent IEDs during night-time sleep.^44^

## Supplementary Material

fcag294_Supplementary_Data

## Data Availability

The de-identified EEG datasets analysed in this study are available from the corresponding author upon reasonable request.
