# Supplementary material for: Abnormal local markers of sleep homeostasis in patients with focal epilepsy
Source: Brain Commun. 2026 Jul 31;8(4):fcag294. doi: 10.1093/braincomms/fcag294 (PMC13490870; doi:10.1093/braincomms/fcag294)
Supplement: Supplementary file 1 [file fcag294_supplementary_data.pdf]

Source and Characteristics of Control Participants

Control subjects were recruited from two independent research protocols conducted at the University of Wisconsin–Madison.

1. **Changes in Functional and Effective Connectivity in Sleep and Waking: A Unique Approach to Investigating Brain Disorders (July 2007 – March 2013).**
   This single-site, controlled, unblinded observational study aimed to characterize high-density EEG (hd-EEG) activity during sleep and wakefulness and to investigate local regulation of sleep using hd-EEG, transcranial magnetic stimulation (TMS), and MRI. The project established a normative database of sleep recordings from healthy adults and clinical populations. The study was not registered on *ClinicalTrials.gov* as it was purely observational and non-therapeutic.

Exclusion criteria included insulin-treated diabetes, recent myocardial infarction, active neoplastic disease, significant medical abnormalities, psychiatric or substance-use disorders, pregnancy, shift work, and use of medications with psychotropic or sleep-modifying effects. Comparison subjects were required to be good sleepers (7–9 h/night, consistent schedule, low caffeine consumption) with no history of neurological, psychiatric, or sleep disorders.

2. **The Effects of Well-Being Interventions on Affect, Attention, Sleep, Social Stress and Pain Regulation (November 2009 – March 2012; *ClinicalTrials.gov* ID NCT01057368).**
   This study investigated behavioral and neural mechanisms of mindfulness-based and compassion/loving-kindness meditation across 150 participants (120 meditation-naïve and 30 long-term practitioners). Data collection included hd-EEG, fMRI, and TMS assessments of affect, attention, pain, and sleep physiology.

Inclusion criteria required fluency in English to ensure comprehension of instructions, task stimuli, and questionnaires. Exclusion criteria covered major medical and psychiatric conditions (including chronic pain, inflammatory, or respiratory diseases), BMI > 35, shift work, and current use of psychotropic or

sleep-affecting medications.

All parent studies were approved by the University of Wisconsin–Madison Institutional Review Board, and written informed consent was obtained from all participants.

Supplementary Table 1. Peaks of significance for differences in SWA, theta and slow wave slope between HC and FE

**Statistics (df/N)**	**Side**	**Scalp coordinates (x, y)**	**T value**	**P value corrected**	**P value uncorrected**
**SWA power: average referenced EEG, patients > controls**					
SPM (85/87)	Right	59.50, 45.12	4.35	0.002	<0.001
	Left	-61.62, 15.56	4.2	0.019	<0.001
SNPM (85/87)	Right	59.5, 45.12	4.07	0.006[v]	<0.001
	Left	-59.5 20.93	4.07	0.006[v] 0.045[c]	<0.001
**Theta power: average referenced EEG, patients > controls**					
SPM (85/87)	Right	44.62, 23.62	6.13	<0.001	<0.001
	Left	-59.50, 15.56	5.47	<0.001	<0.001
SNPM (85/87)	Right	44.62, 23.62	5.82	<0.001[v]	<0.001
	Left	-60 16	5.70	<0.001[v]	<0.001
**Average slope of sleep slow waves, patients > controls**					

SPM (85/87)	Right	No suprathreshold clusters	-	-	-
	Midline	8.50, 10.19	4.30	0.008	<0.001
	Left	No suprathreshold clusters	-	-	-

SNPM (85/87)	Right	No suprathreshold clusters	-	-	-
	Left	No suprathreshold clusters	-	-	-

Coordinates are expressed as distance from a midline reference point for scalp analyses, and distance from anterior commissure in MNI space for source space analysis. df: degrees of freedom; N: number of subjects included in the model; [v] voxel-level corrected; [c] cluster-level corrected.

Supplementary Table 2. Delta and theta power overnight decline in FE and HC over different brain areas

Group	Region	SWA Power First Part (mV²/Hz)	SWA Power Last Part (mV²/Hz)	Theta Power First Part (mV²/Hz)	Theta Power Last Part (mV²/Hz)
FE	Midline	28.5 ± 6.2	16 ± 4.2	2.8 ± 0.5	2.1 ± 0.4
	Left temporal	15.9 ± 2.2	10.5 ± 1.5	2.1 ± 0.3	1.8 ± 0.2
	Right Temporal	17.4 ± 2.6	11.1 ± 1.8	2.3 ± 0.3	1.9 ± 0.2
HC	Midline	31.6 ± 3.9	13.4 ± 2.2	2.3 ± 0.2	1.5 ± 0.2
	Left Temporal	11.7 ± 1.4	5.4 ± 0.7	1.3 ± 0.1	0.9 ± 0.1
	Right Temporal	12.1 ± 1.6	6.5 ± 0.9	1.3 ± 0.2	0.9 ± 0.1

In detail, in patients, average referenced EEG SWA power means were 28.5 ± 6.2 mV2 /Hz in the

first part and 16 ± 4.2 mV2 /Hz in the last part of the night at the midline, 15.9 ± 2.2 mV2 /Hz in the first part and 10.5 ± 1.5 mV2 /Hz in the last part of the night on the left temporal areas and 17.4 ± 2.6 mV2 /Hz in the first part and 11.1 ± 1.8 mV2 /Hz in the last part of the night on the right temporal areas **(Figure 1).**

In HC, average referenced EEG SWA power means were 31.6 ± 3.9 mV2 /Hz in the first part and 13.4 ± 2.2 mV2 /Hz in the last part of the night at the midline, 11.7 ± 1.4 mV2 /Hz in the first part and 5.4 ± 0.7 mV2 /Hz in the last part of the night on the left temporal areas and 12.1 ± 1.6 mV2 /Hz in the first part and 6.5 ± 0.9 mV2 /Hz in the last part of the night on the right temporal areas **(Figure 1).**

In patients, average referenced EEG theta power means were 2.8 ± 0.5 mV2 /Hz in the first part and 2.1 ± 0.4 mV2 /Hz in the last part of the night at the midline, 2.1 ± 0.3 mV2 /Hz in the first part and 1.8 ± 0.2 mV2 /Hz in the last part of the night on the left temporal areas and 2.3 ± 0.3 mV2 /Hz in the first part and 1.9 ± 0.2 mV2 /Hz in the last part of the night on the right temporal areas **(Figure 2).**

In HC, average referenced EEG theta power means were 2.3 ± 0.2 mV2 /Hz in the first part and 1.5 ± 0.2 mV2 /Hz in the last part of the night at the midline, 1.3 ± 0.1 mV2 /Hz in the first part and 0.9 ± 0.1 mV2 /Hz in the last part of the night on the left temporal areas and 1.3 ± 0.2 mV2 /Hz in the first part and 0.9 ± 0.1 mV2 /Hz in the last part of the night on the right temporal areas **(Figure 2).**

Supplementary Table 3. Normalized overnight theta values

FE	Maxima	Minima	HC	Maxima	Minima
P02	22,42078	13,79599	C01	19,20634	14,56439
P03	77,28442	21,84433	C02	18,37673	12,06182
P04	43,75028	32,26235	C03	41,19458	17,82169
P05	13,01281	21,52407	C04	17,69755	11,36137
P06	68,26711	16,12662	C05	20,32256	24,05036
P07	34,25357	17,74998	C06	16,6828	14,87156
P08	47,19333	15,00572	C07	24,10931	12,55234

**P09**	63,77069	18,15674	**C08**	16,10137	16,11527
**P10**	59,69778	17,02497	**C09**	24,05982	17,91417
**P11**	35,47229	20,0739	**C10**	15,83817	12,54771
**P12**	21,15609	12,04121	**C11**	22,85052	15,80486
**P13**	19,34191	17,26556	**C12**	21,60729	16,8682
**P14**	108,0087	17,45877	**C13**	29,95508	12,43408
**P15**	29,04798	17,44561	**C14**	32,42431	20,79888
**P18**	34,374	39,96122	**C15**	21,18751	19,31775
**P19**	33,32248	24,17002	**C16**	23,94696	22,76084
**P23**	54,50832	23,89981	**C17**	18,24952	9,727445
**P24**	57,57264	25,70845	**C18**	17,31022	12,58301
**P25**	28,48908	25,17808	**C19**	18,83436	12,09518
**P26**	41,81482	23,48975	**C20**	30,69162	20,4856
**S01**	28,36229	21,59268	**C21**	36,52625	15,22752
**S02**	43,78112	24,42439	**C22**	25,80825	13,05295
**S04**	28,17985	22,09482	**C23**	17,04516	11,55375
**S05**	33,82034	21,39334	**CS01**	19,93203	11,07144
**S06**	34,40616	21,47394	**CS02**	39,39768	17,17333
**S07**	36,19762	14,78054	**CS03**	18,39741	11,75388
**S09**	34,56535	40,23601	**CS04**	17,62725	12,65261
**S10**	47,33489	28,34924	**CS05**	33,07337	20,13932
**S11**	35,19561	22,50823	**CS06**	37,74384	18,95788
**S13**	43,61399	15,62504	**CS07**	41,13538	11,63243
**S14**	14,40067	13,71631	**CS08**	22,31452	18,96228
**S16**	35,50126	40,61452	**CS09**	16,77249	21,06133
**S17**	23,97087	19,48052	**CS10**	12,33412	11,60925
**S19**	31,19845	23,30267	**CS11**	15,60128	15,50101
**S20**	33,61053	25,76892	**CS12**	16,8498	11,48169
**S21**	30,12243	23,29524	**CS13**	15,73178	18,07307
**S22**	34,52515	16,04646	**CS14**	21,58845	18,67232

**S24**	39,13844	20,38992	**CS15**	18,84248	10,70562
**S26**	28,2423	16,92737	**CS16**	15,24856	10,60837
			**CS17**	21,42291	16,85864
			**CS18**	17,39333	12,18604
			**CS19**	13,86228	25,78265
			**CS20**	35,15908	19,92523
			**CS21**	17,16135	22,35414
			**CS23**	24,65561	26,72486
			**CS24**	17,13759	21,35386
			**CS25**	16,06685	18,04544
			**CS26**	20,32354	16,74084

Supplementary Table 4. Anti-seizure medications (ASMs) in patients with focal epilepsy

Subject	Anti-seizure medications (total daily dose)
S01	Carbamazepine 400 mg/day; Levetiracetam 500 mg/day
S02	Divalproex 500 mg/day; Levetiracetam 500 mg/day
S03	Divalproex 500 mg/day; Levetiracetam 500 mg/day
S04	Carbamazepine 200 mg/day; Zonisamide 100 mg/day
S05	Levetiracetam 1500 mg/day; Topiramate 100 mg/day
S06	Lamotrigine 100 mg/day; Levetiracetam 1500 mg/day; Lorazepam 1 mg/day
S07	Phenobarbital 32.4 mg/day; Zonisamide 100 mg/day
S08	Carbamazepine 200 mg/day; Zonisamide 100 mg/day
S09	Pregabalin 150 mg/day; Topiramate 100 mg/day
S10	Divalproex 500 mg/day; Levetiracetam 250 mg/day
S11	Lamotrigine 200 mg/day; Levetiracetam 750 mg/day
S12	Clonazepam 0.5 mg/day; Lamotrigine 200 mg/day; Levetiracetam 500 mg/day; Lorazepam 0.5 mg/day
S13	Clonazepam 0.25 mg/day; Lamotrigine 200 mg/day; Zonisamide 100 mg/day
S14	Diazepam 5 mg/day; Levetiracetam 1000 mg/day; Oxcarbazepine 600 mg/day
S15	Lacosamide 200 mg/day; Lamotrigine 200 mg/day; Zonisamide 100 mg/day
S16	Lamotrigine 100 mg/day
S17	Lacosamide 200 mg/day; Levetiracetam 500 mg/day
S18	Carbamazepine 400 mg/day; Levetiracetam 500 mg/day; Lorazepam 0.5 mg/day; Phenytoin 100 mg/day
S19	Oxcarbazepine 600 mg/day; Perampanel 6 mg/day
S20	Lacosamide 150 mg/day; Lamotrigine 100 mg/day; Levetiracetam 1750 mg/day; Lorazepam 1 mg/day; Midazolam 10 mg (rescue medication)

Subject	Anti-seizure medications (total daily dose)
S21	Levetiracetam 1250 mg/day; Carbamazepine 400 mg/day
S22	Lamotrigine 700 mg/day
S23	Divalproex 1250 mg/day; Lacosamide 400 mg/day
S24	Lamotrigine 400 mg/day; Topiramate 200 mg/day
S25	Lamotrigine 700 mg/day; Levetiracetam 3000 mg/day; Zonisamide 200 mg/day
S26	Divalproex 2250 mg/day
S27	Lamotrigine 400 mg/day; Levetiracetam 750 mg/day
S28	Levetiracetam 3000 mg/day; Divalproex 1500 mg/day
S29	Lamotrigine 500 mg/day
S30	Levetiracetam 3750 mg/day; Phenobarbital 145.8 mg/day; Zonisamide 300 mg/day; Clonazepam PRN
S31	No ASM at time of recording
S32	Clonazepam 0.5 mg/day; Lacosamide 400 mg/day; Perampanel 12 mg/day; Topiramate 800 mg/day
S33	Lamotrigine 600 mg/day; Zonisamide 500 mg/day; Lorazepam PRN
S34	Lamotrigine 300 mg/day; Levetiracetam 4000 mg/day
S35	Lacosamide 300 mg/day; Lamotrigine 800 mg/day; Levetiracetam 3500 mg/day; Gabapentin 600 mg/day; additional 500 mg PRN
S36	Carbamazepine 700 mg/day; Lamotrigine 650 mg/day
S37	Lamotrigine 500 mg/day (Zonisamide discontinued 4 weeks prior to recording)
S38	Lamotrigine 500 mg/day; Zonisamide 100 mg/day
S39	Lamotrigine 450 mg/day; Levetiracetam 3000 mg/day

Supplementary Table 4. The table reports the anti-seizure medications taken by each patient at the time of HD-EEG recording. Patients were recorded under their usual clinical treatment. Treatment regimens were heterogeneous, and most patients were receiving polytherapy. Drug names are reported in generic form. Medications prescribed on an as-needed basis are indicated as PRN

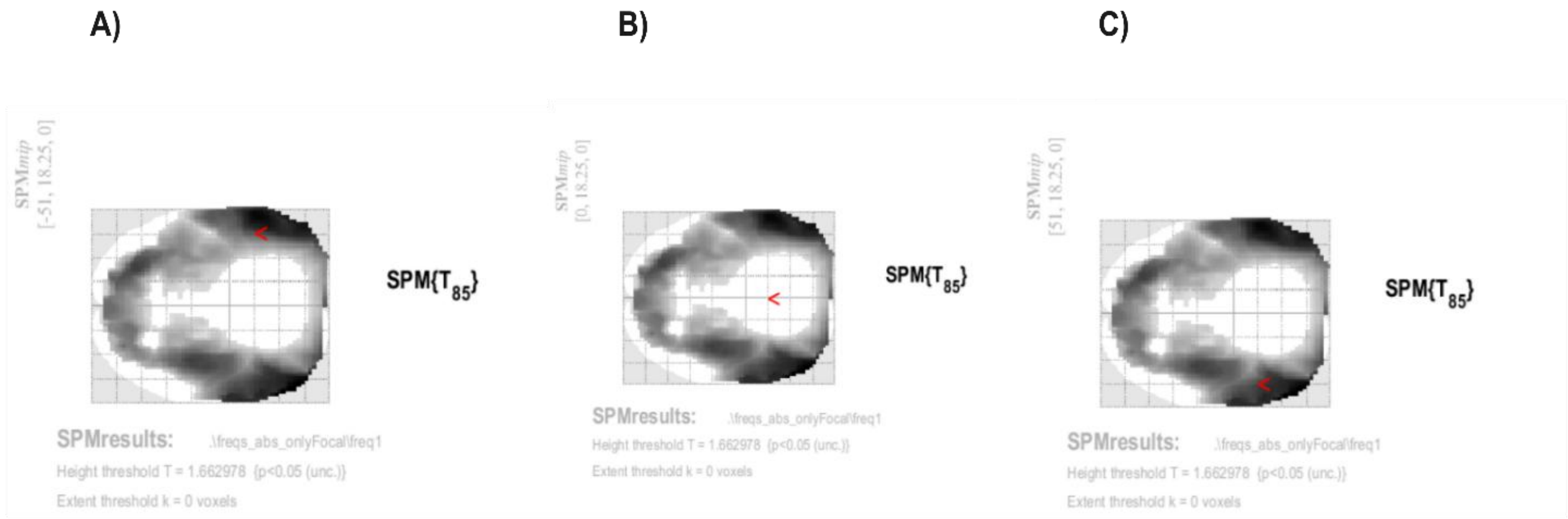

Supplementary Figure 1. ***Schematic illustration of the three standard electrode locations used for group-level analyses.*** A). Left temporal. B) Midline. C) Right Temporal.

The midline site (between Fz and Cz) was selected for its known maximal expression of NREM sleep features, including slow waves and K-complexes. Left and right temporal sites were chosen symmetrically over the temporal lobes to capture potential focal alterations in sleep markers in patients with focal epilepsy. The highlighted positions indicate the approximate scalp locations employed across all subjects.

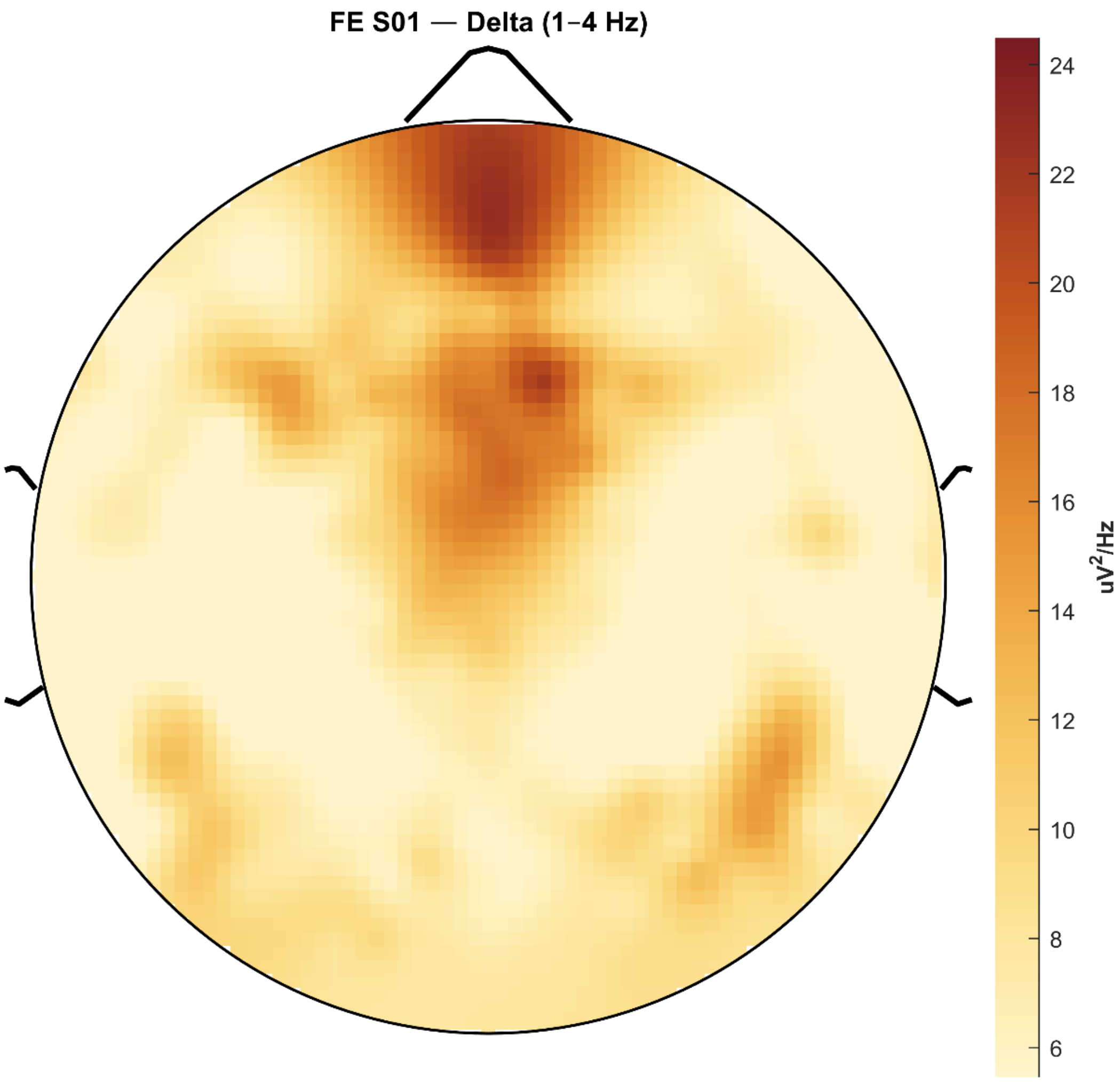

Supplementary Figure 2

Delta (1–4 Hz) power map for FE subject S01. The color scale represents delta power ($\mu V^2/Hz$).

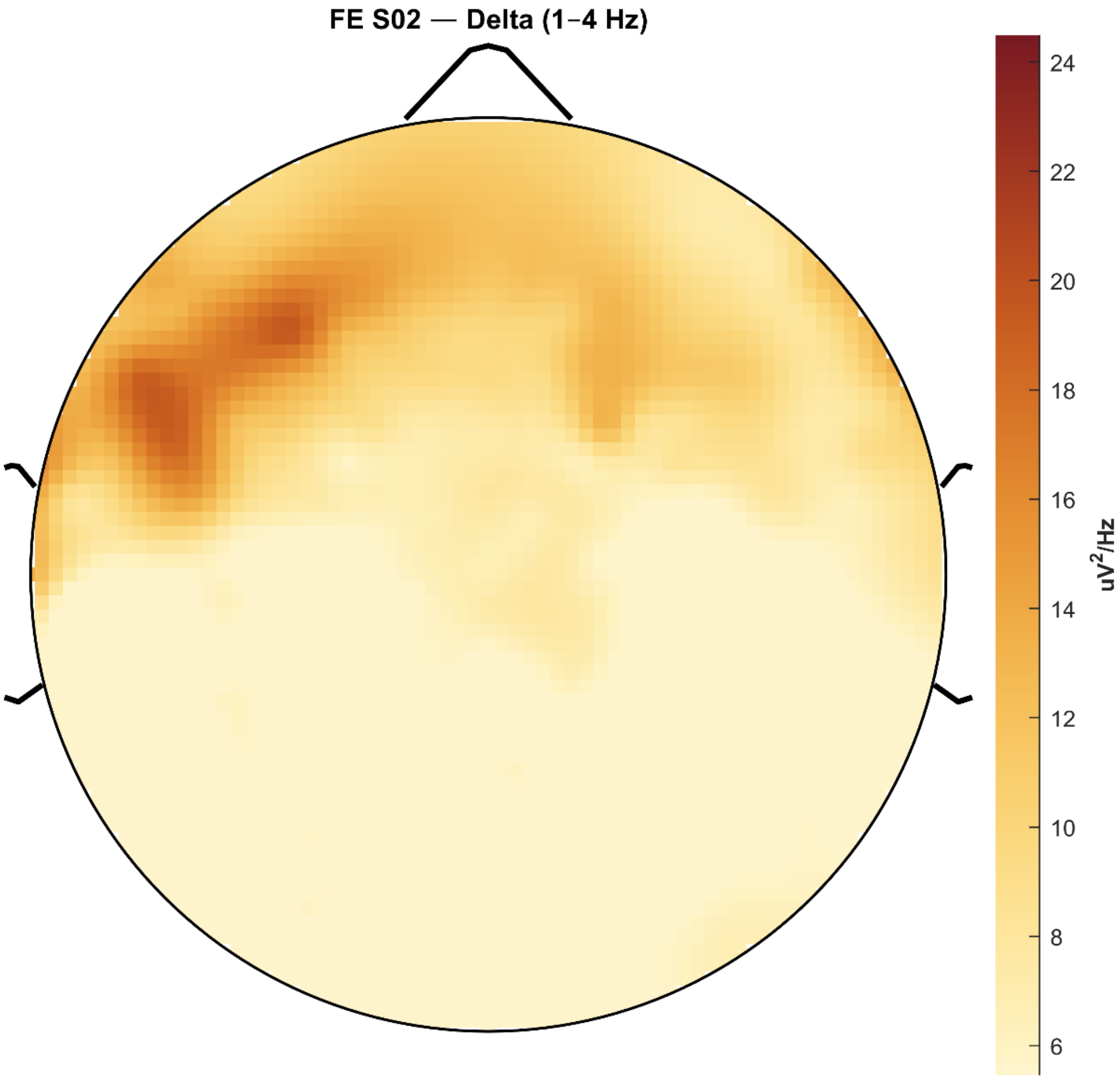

Supplementary Figure 3

Delta (1–4 Hz) power map for FE subject S02. The color scale represents delta power (µV²/Hz).

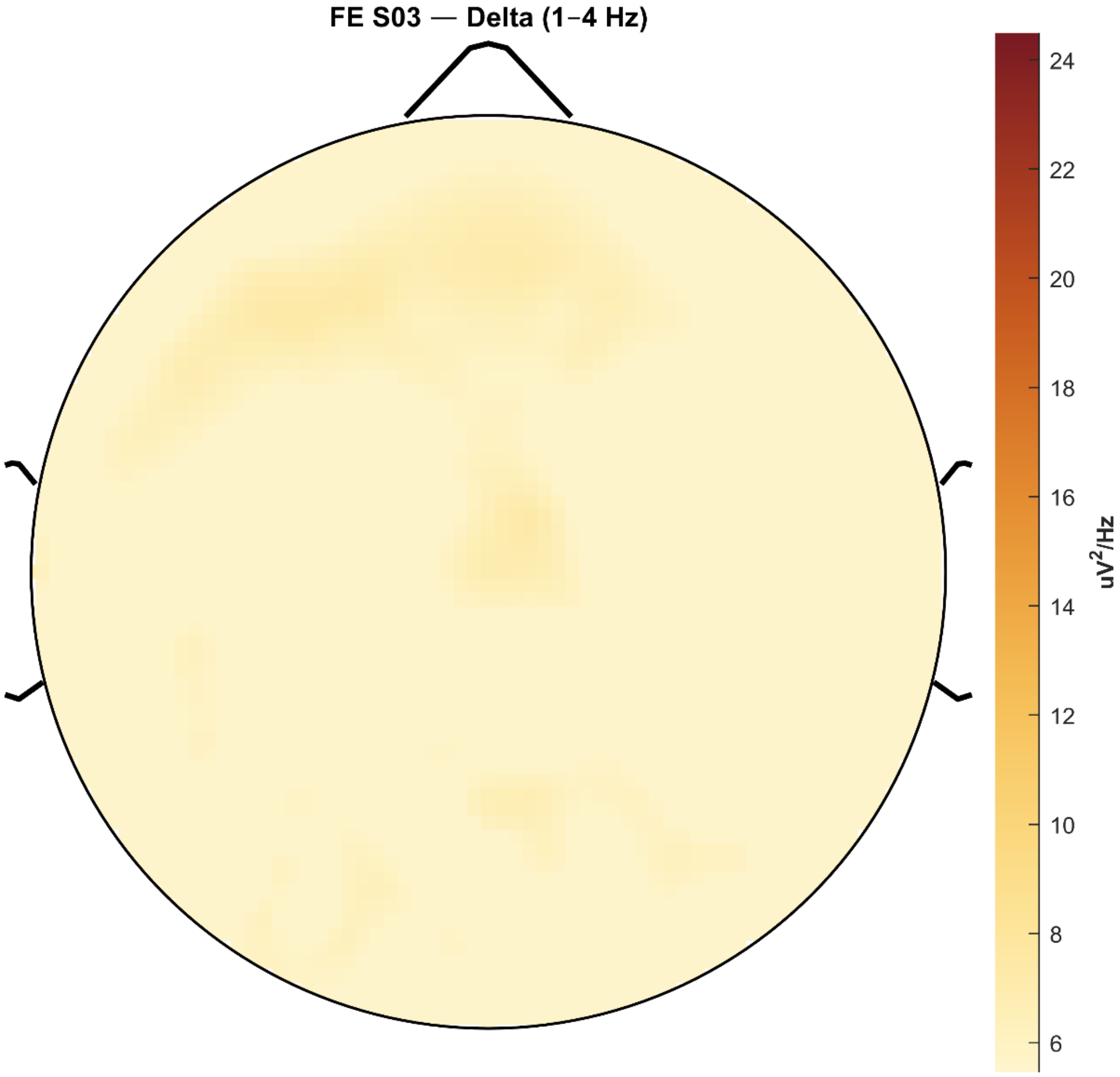

Supplementary Figure 4

Delta (1–4 Hz) power map for FE subject S03. The color scale represents delta power (µV²/Hz).

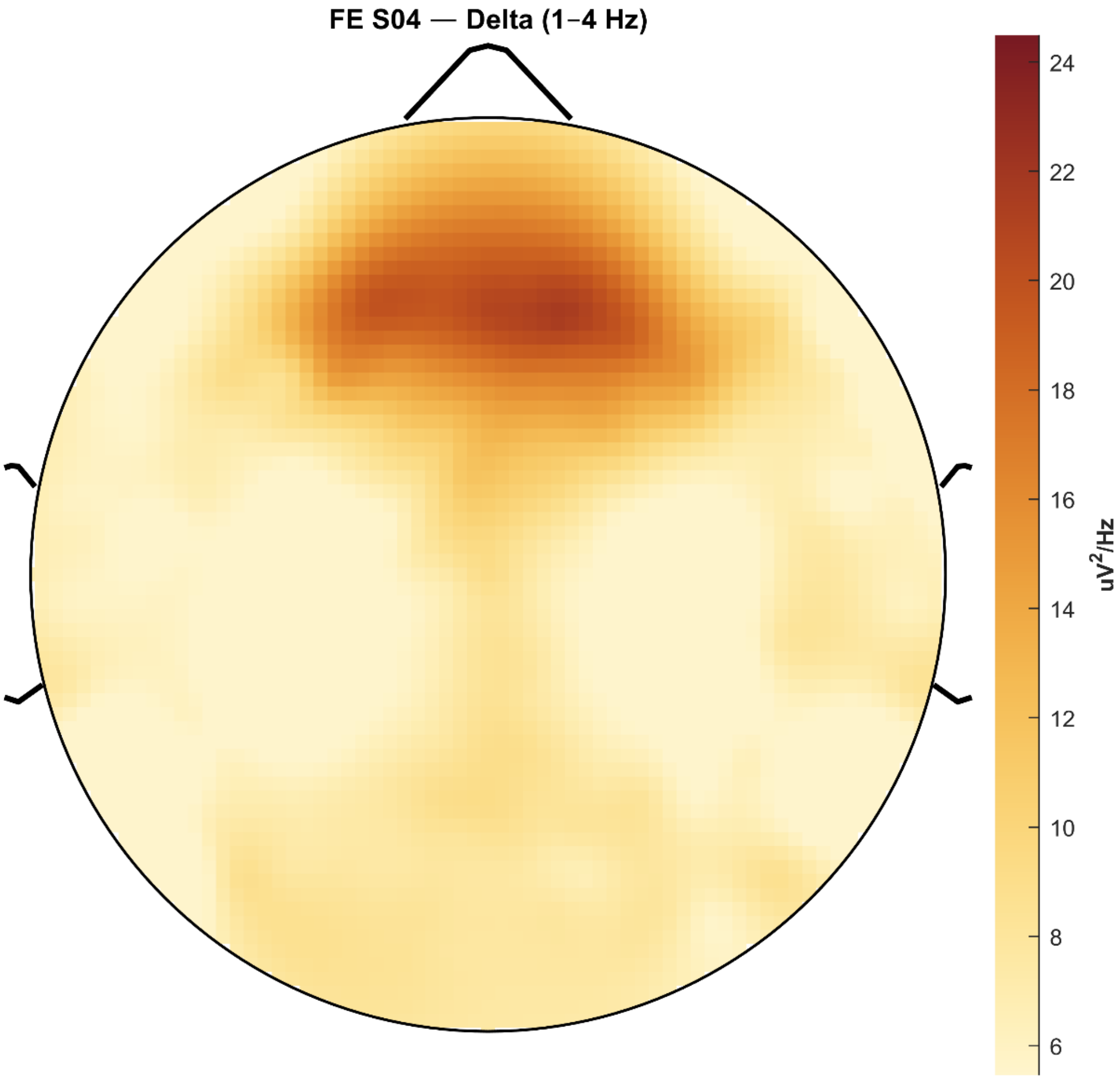

Supplementary Figure 5

Delta (1–4 Hz) power map for FE subject S04. The color scale represents delta power (µV²/Hz).

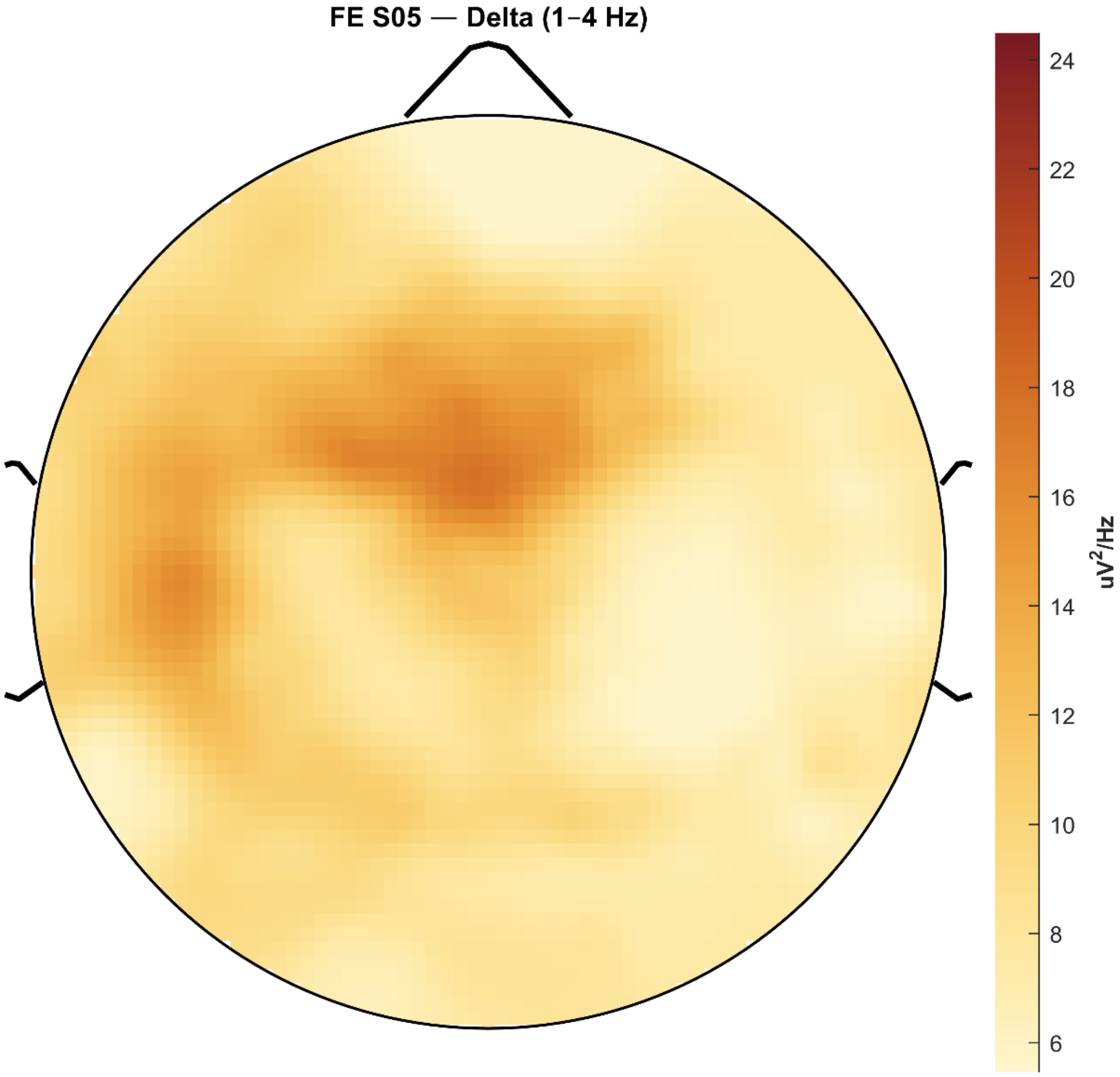

Supplementary Figure 6

Delta (1–4 Hz) power map for FE subject S05. The color scale represents delta power (µV²/Hz).

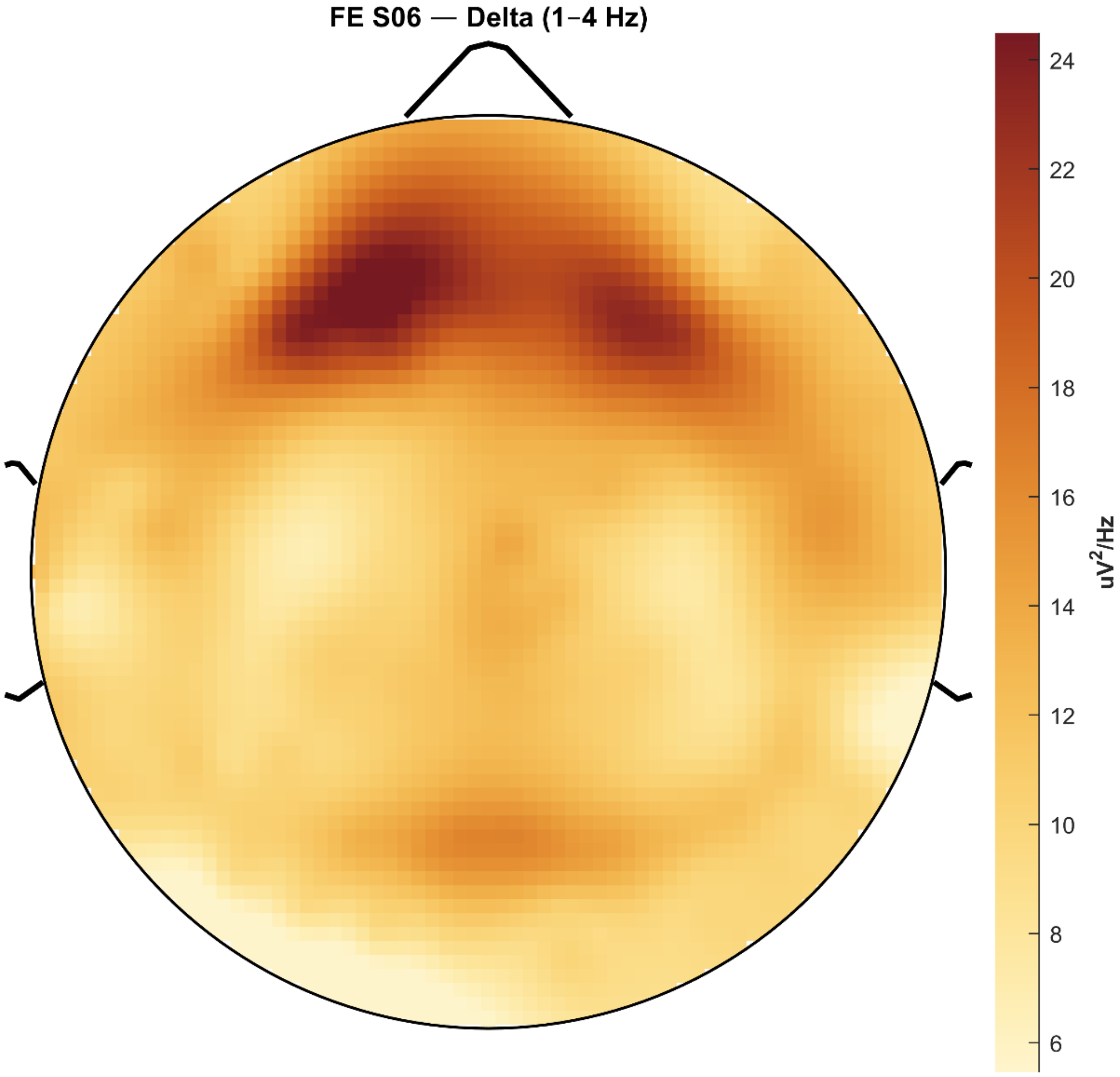

Supplementary Figure 7

Delta (1–4 Hz) power map for FE subject S06. The color scale represents delta power (µV²/Hz).

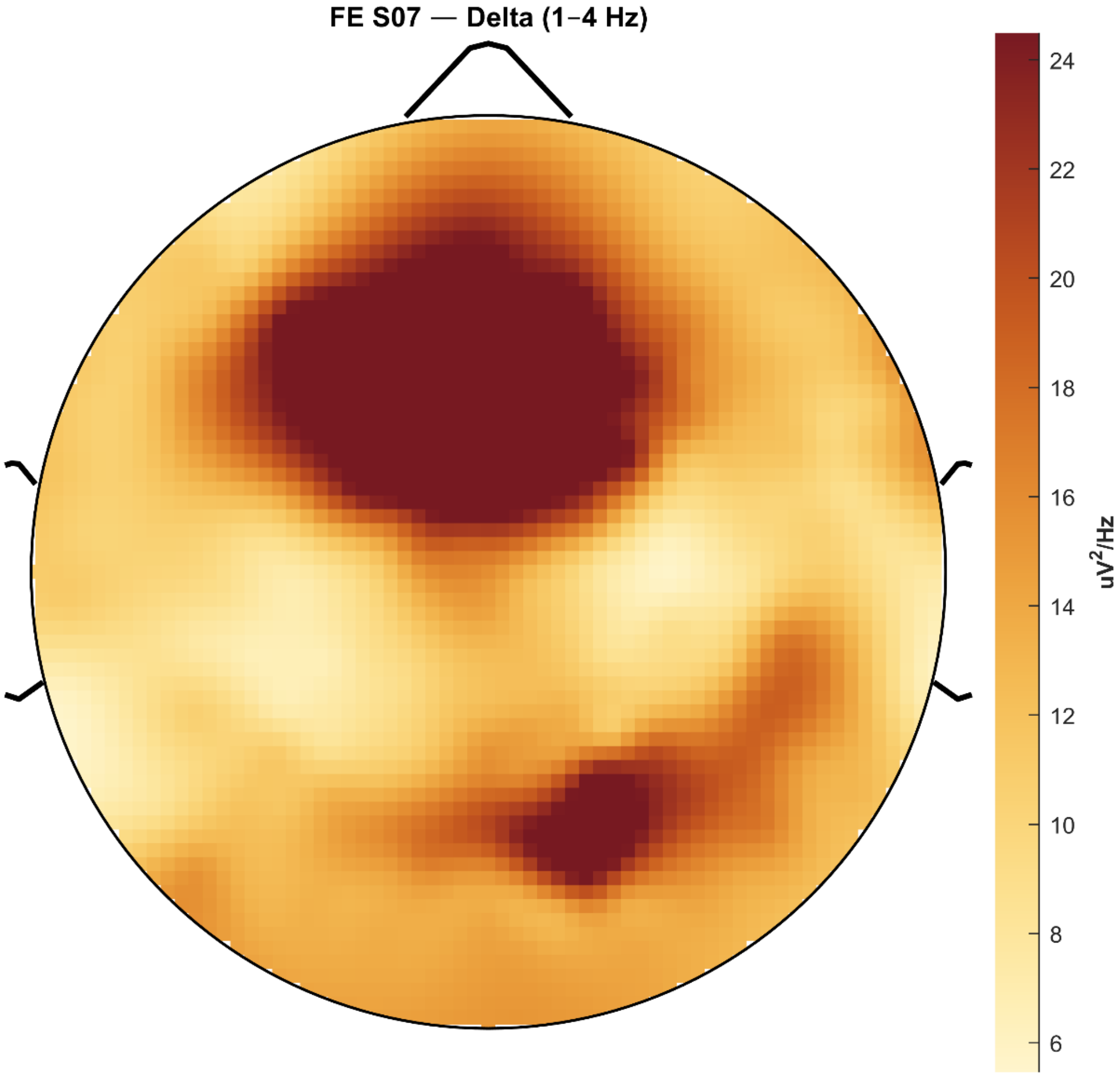

Supplementary Figure 8

Delta (1–4 Hz) power map for FE subject S07. The color scale represents delta power (µV²/Hz).

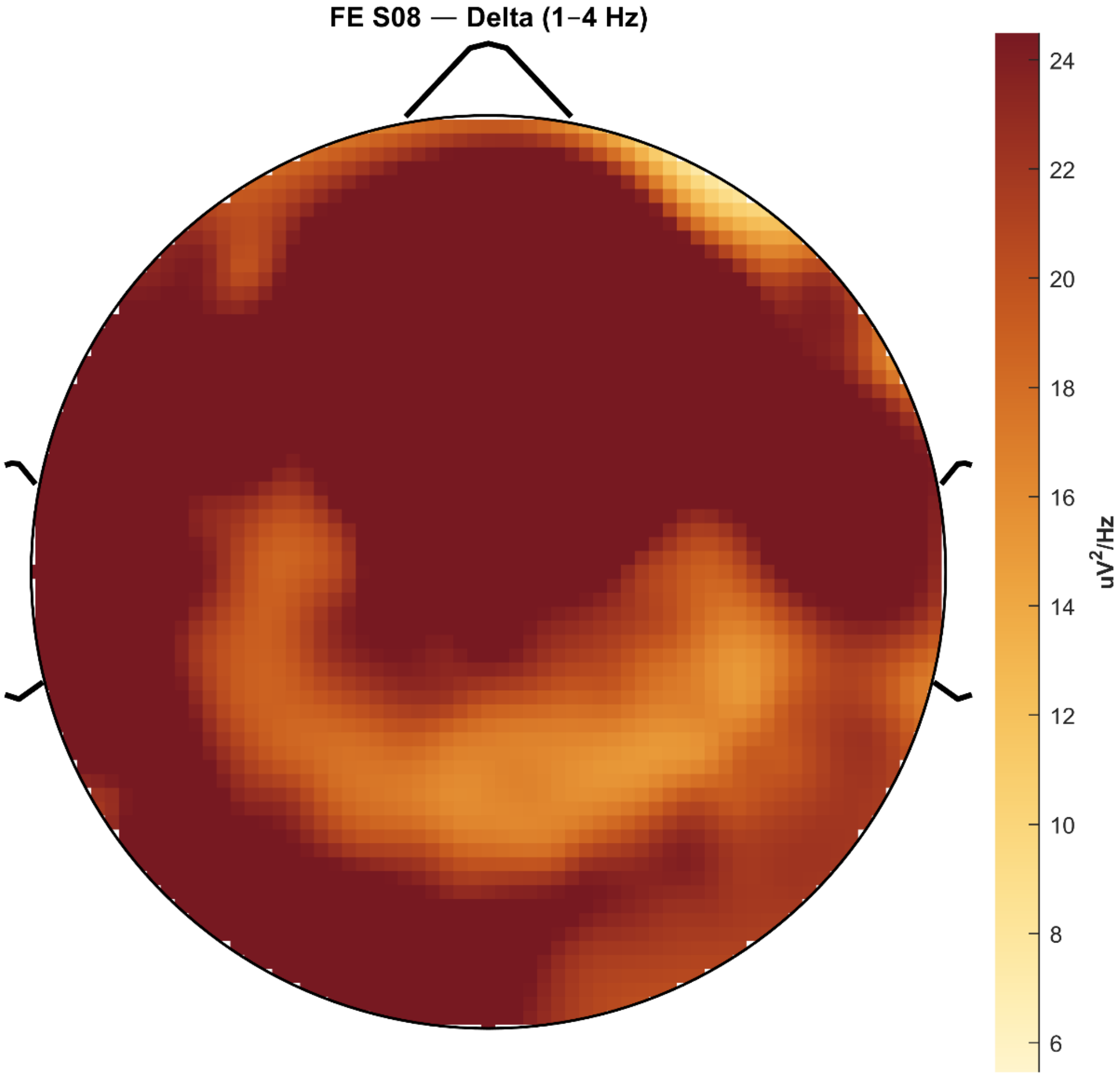

Supplementary Figure 9

Delta (1–4 Hz) power map for FE subject S08. The color scale represents delta power (µV²/Hz).

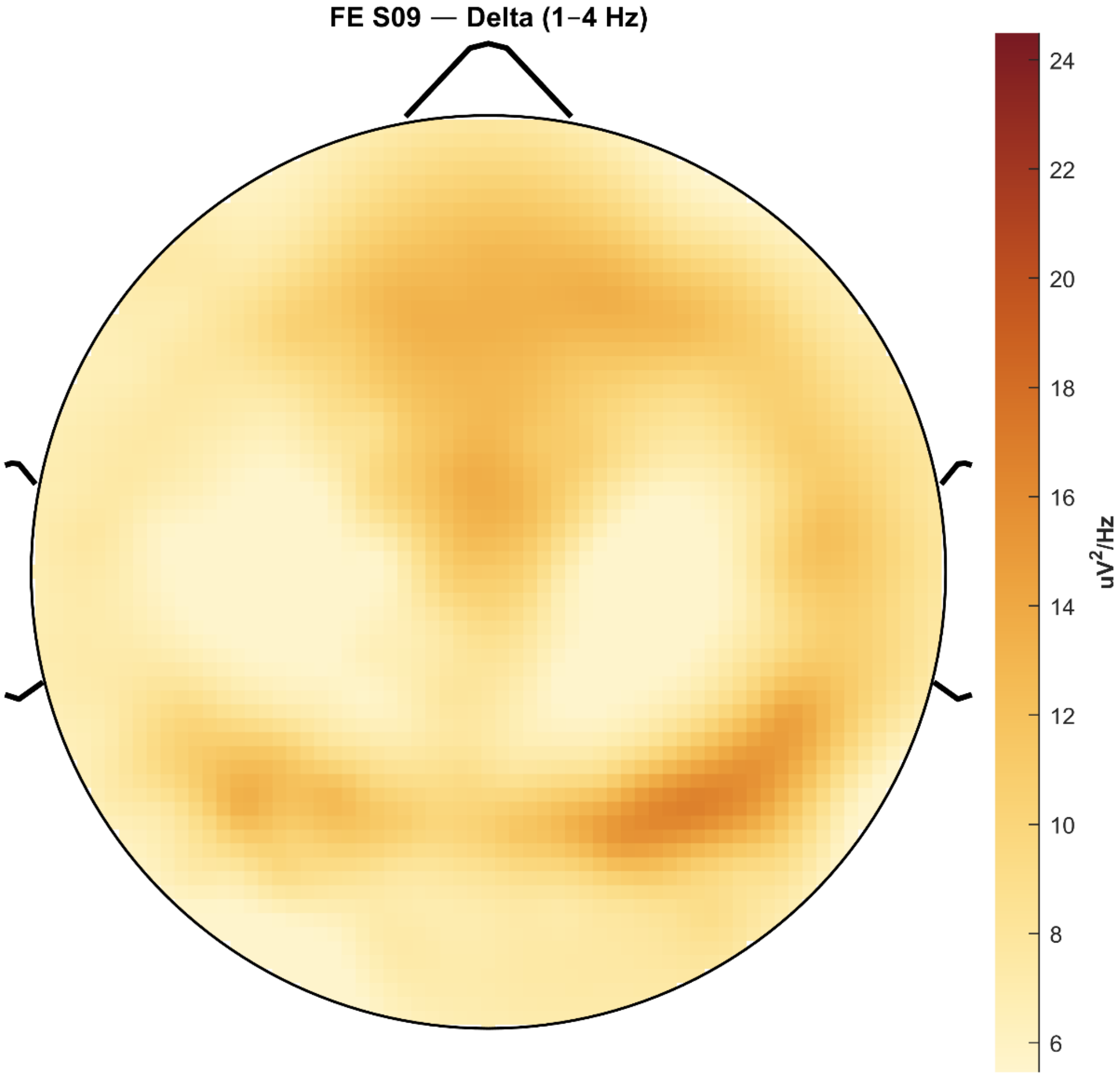

Supplementary Figure 10

Delta (1–4 Hz) power map for FE subject S09. The color scale represents delta power (µV²/Hz).

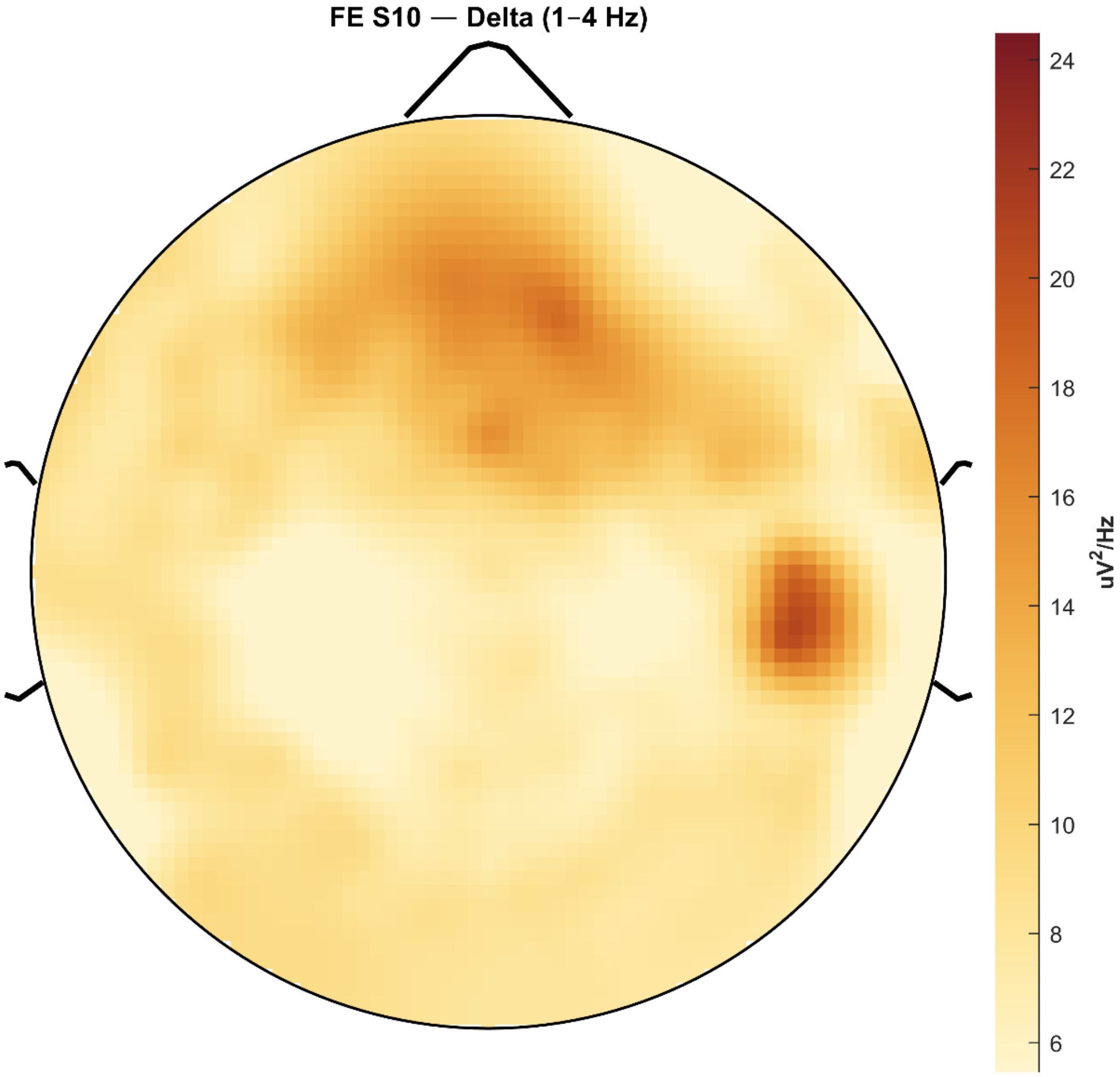

Supplementary Figure 11

Delta (1–4 Hz) power map for FE subject S10. The color scale represents delta power (µV²/Hz).

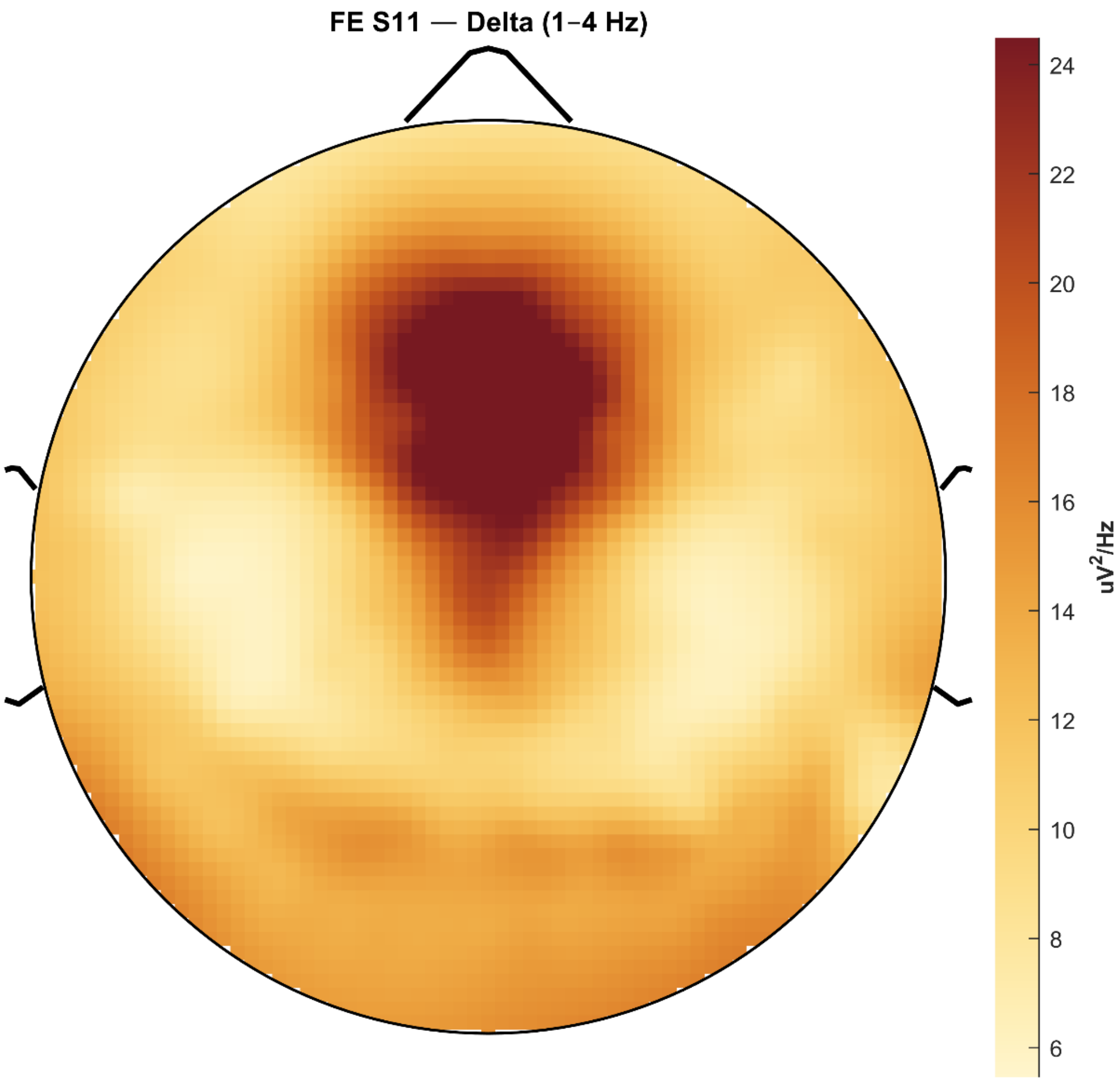

Supplementary Figure 12

Delta (1–4 Hz) power map for FE subject S11. The color scale represents delta power (μV²/Hz).

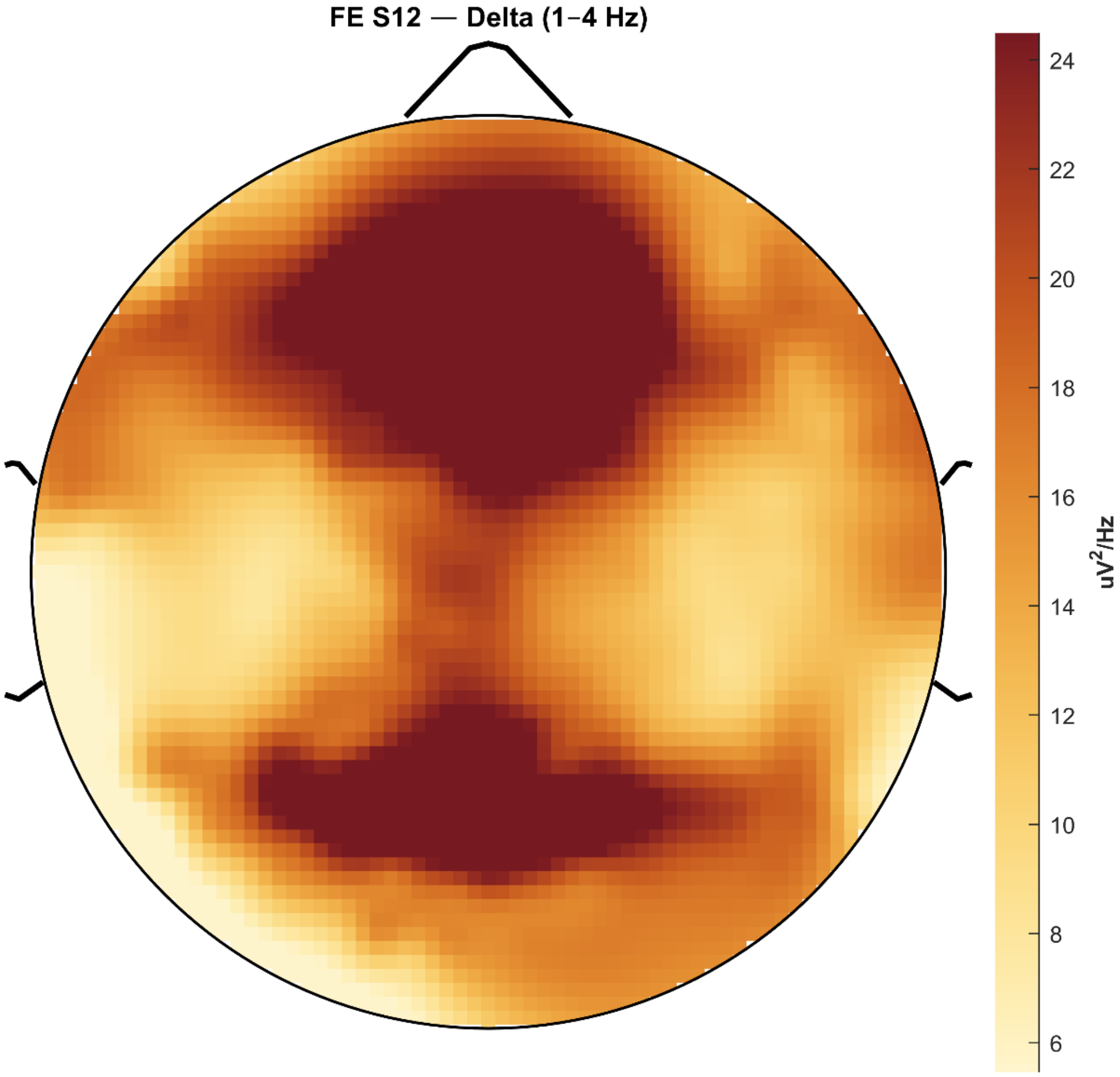

Supplementary Figure 13

Delta (1–4 Hz) power map for FE subject S12. The color scale represents delta power (µV²/Hz).

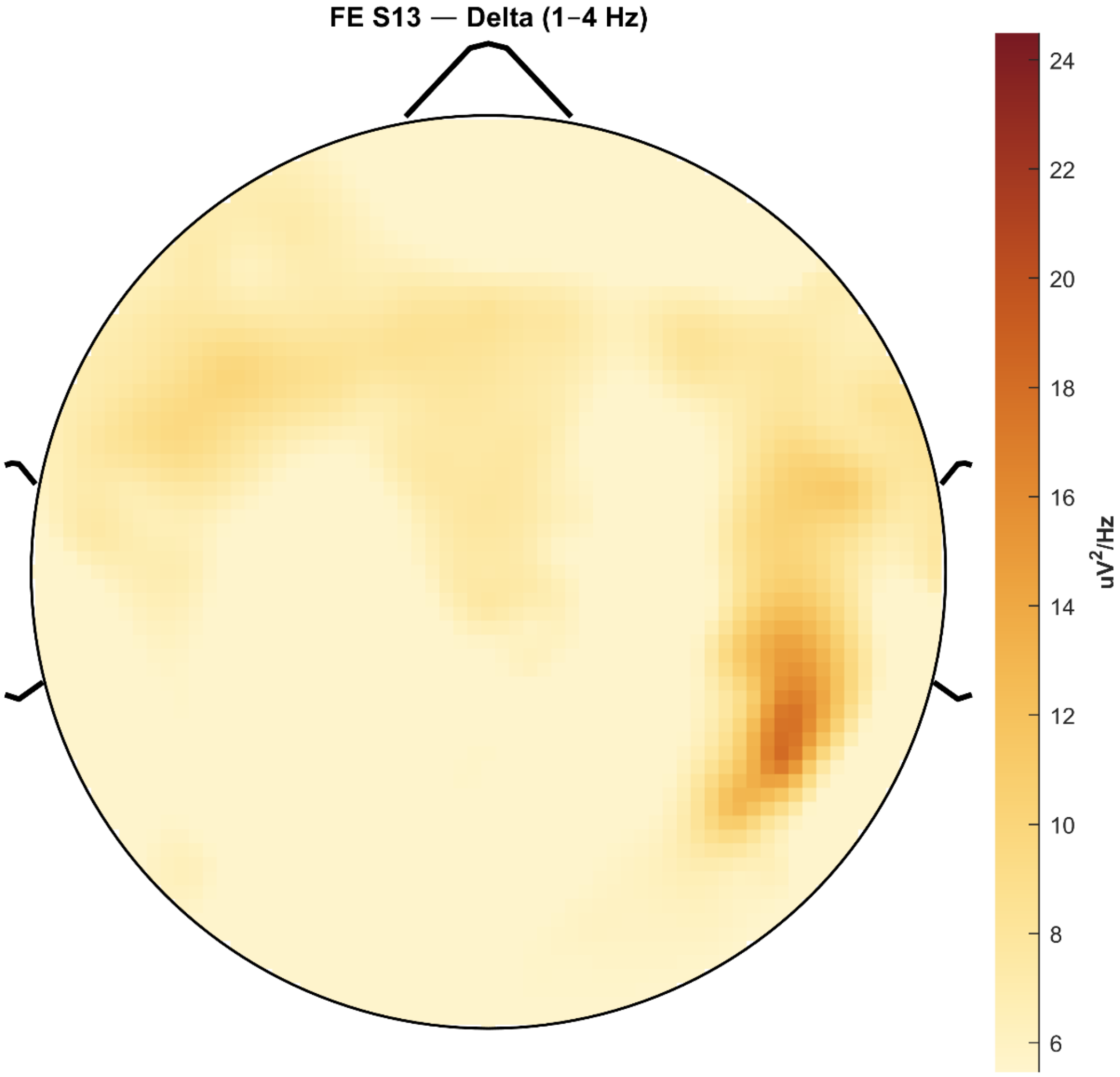

Supplementary Figure 14

Delta (1–4 Hz) power map for FE subject S13. The color scale represents delta power (µV²/Hz).

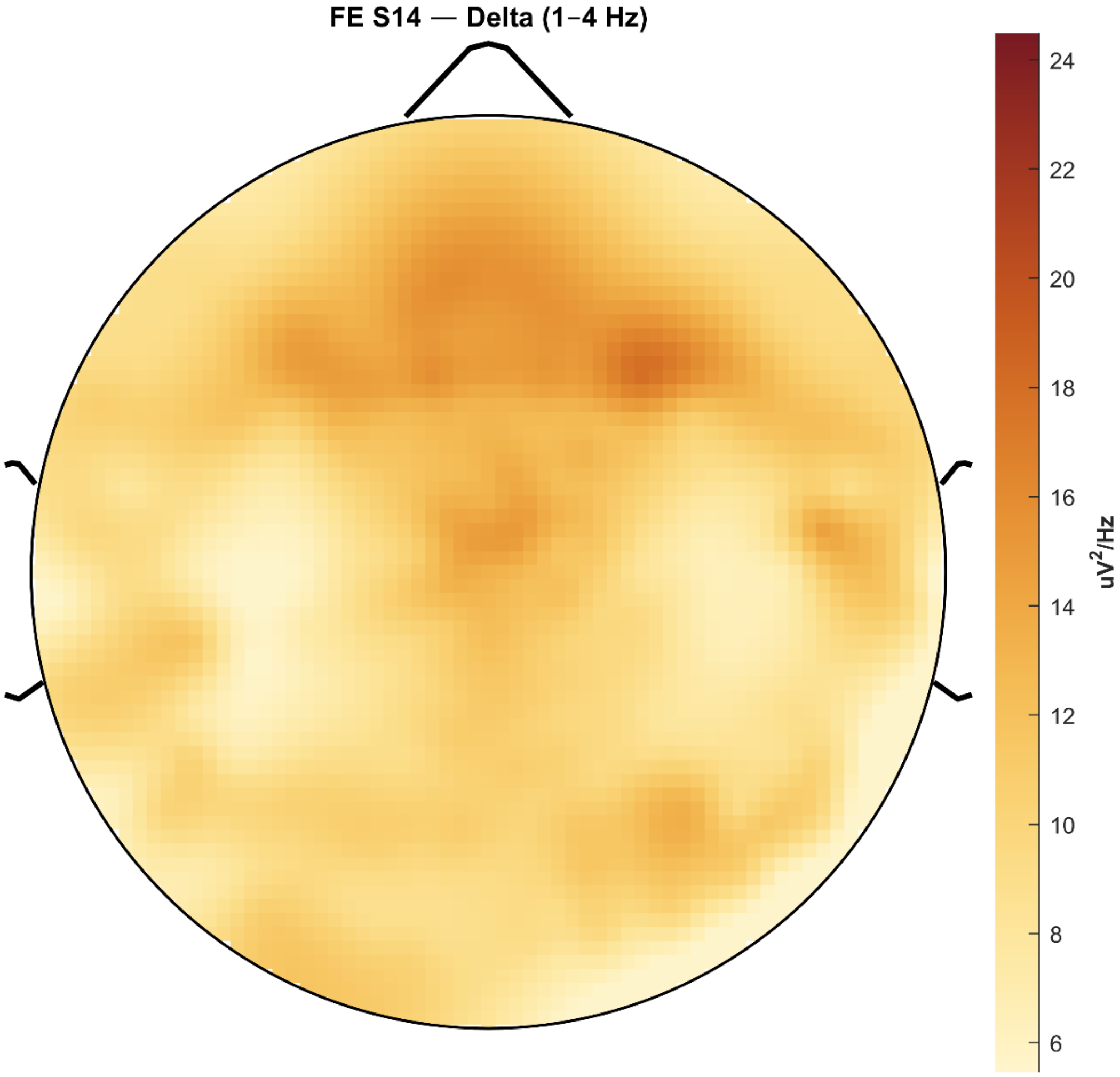

Supplementary Figure 15

Delta (1–4 Hz) power map for FE subject S14. The color scale represents delta power (µV²/Hz).

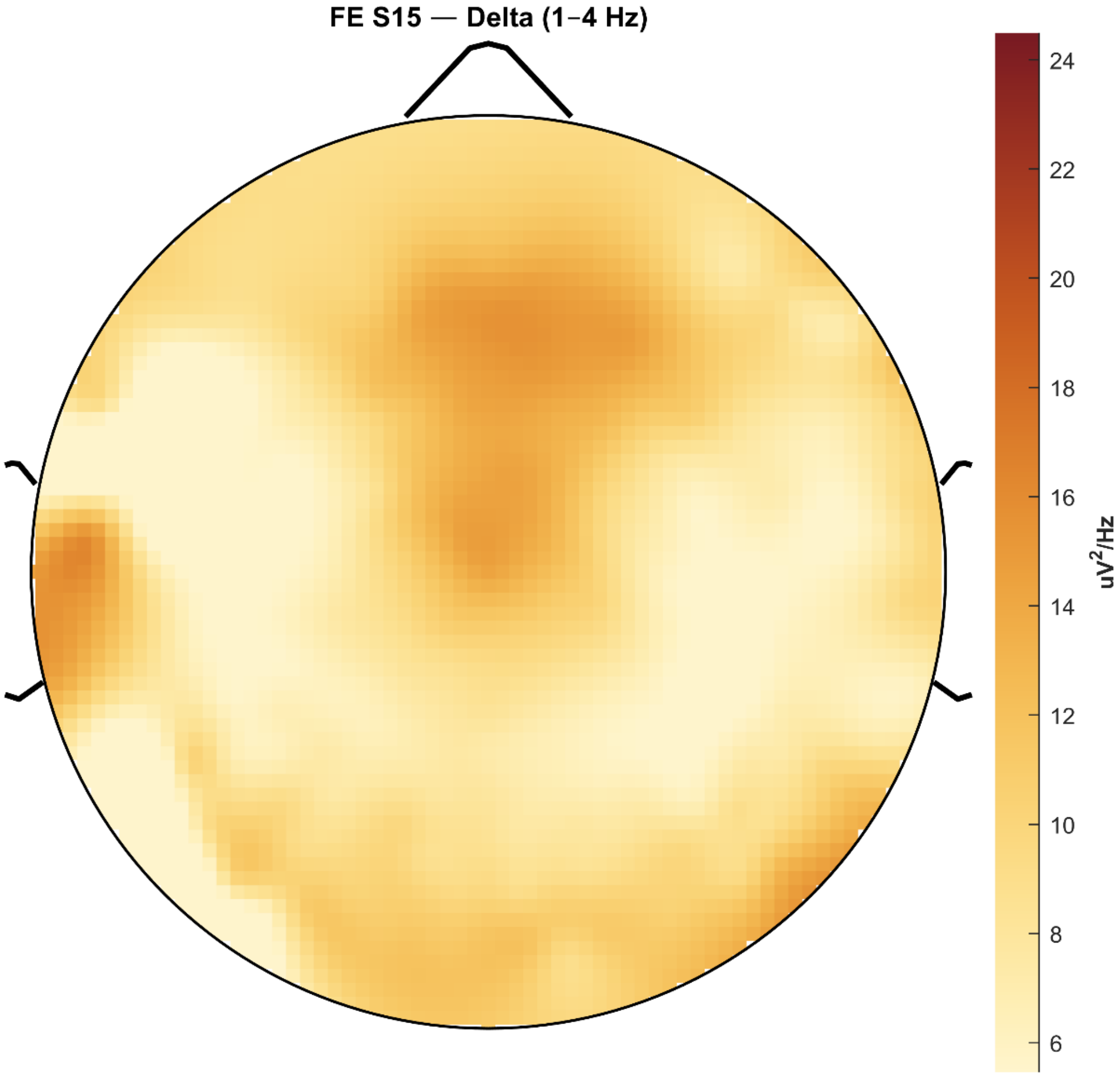

Supplementary Figure 16

Delta (1–4 Hz) power map for FE subject S15. The color scale represents delta power (µV²/Hz).

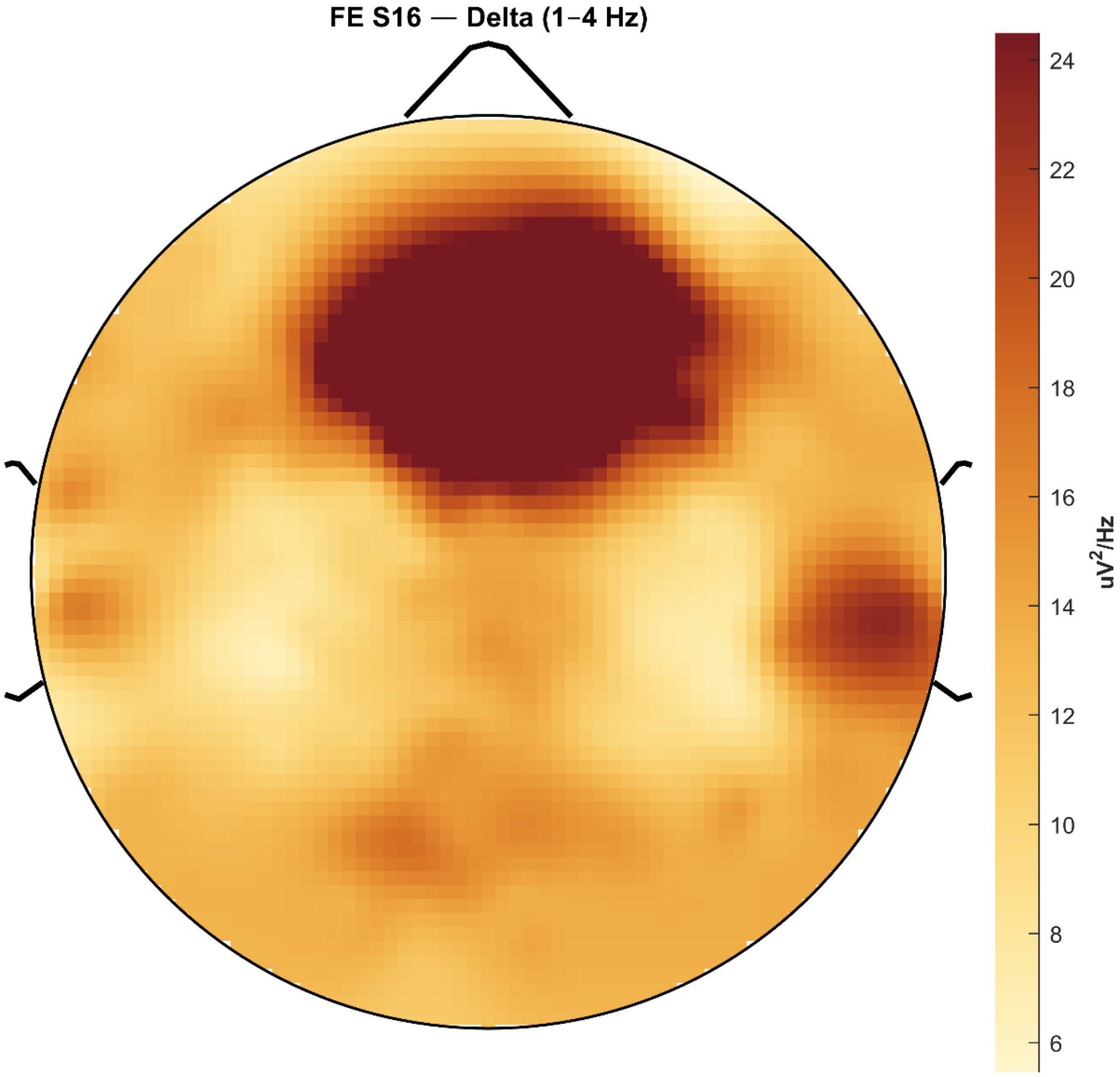

Supplementary Figure 17

Delta (1–4 Hz) power map for FE subject S16. The color scale represents delta power (µV²/Hz).

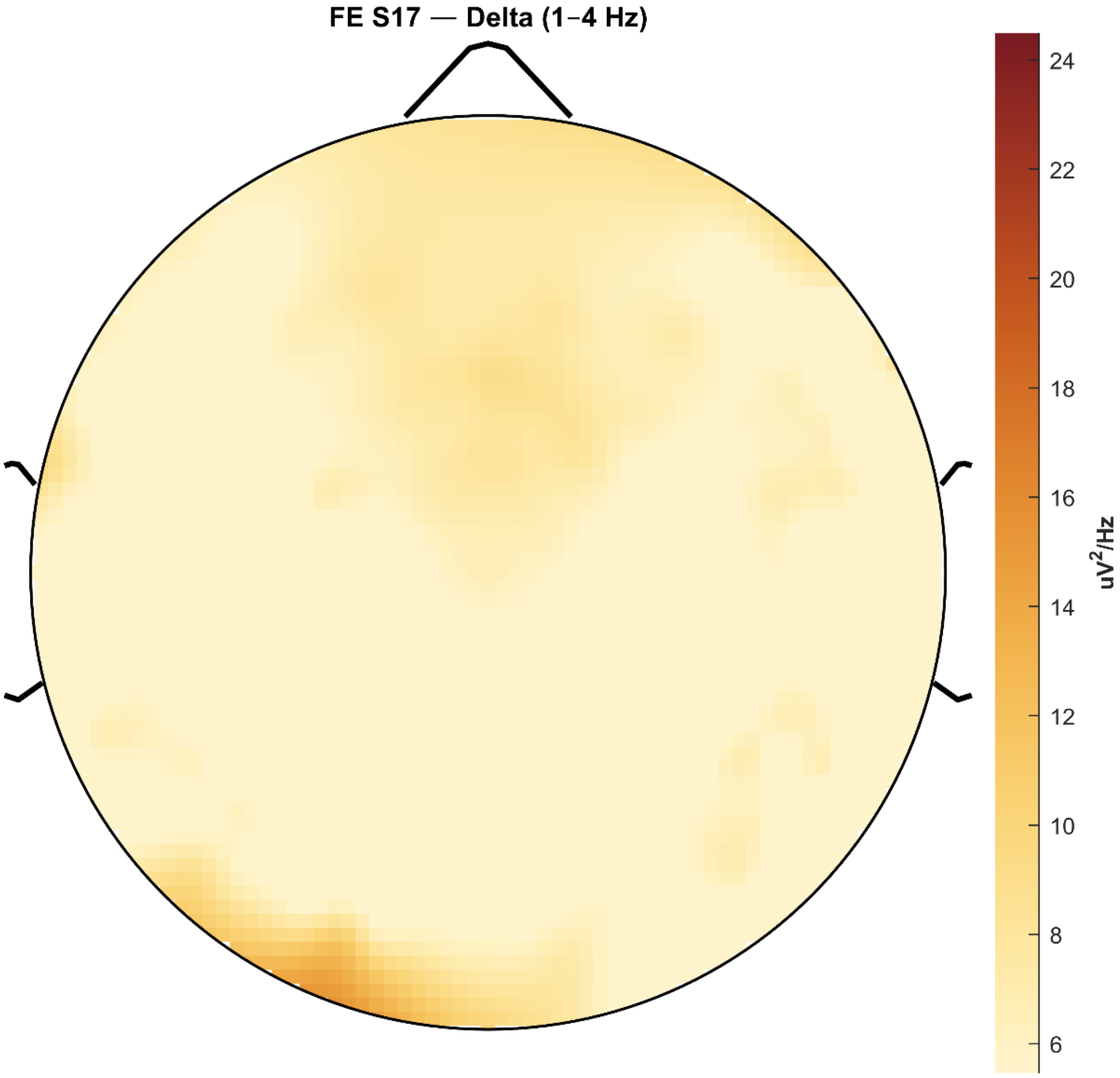

Supplementary Figure 18

Delta (1–4 Hz) power map for FE subject S17. The color scale represents delta power (µV²/Hz).

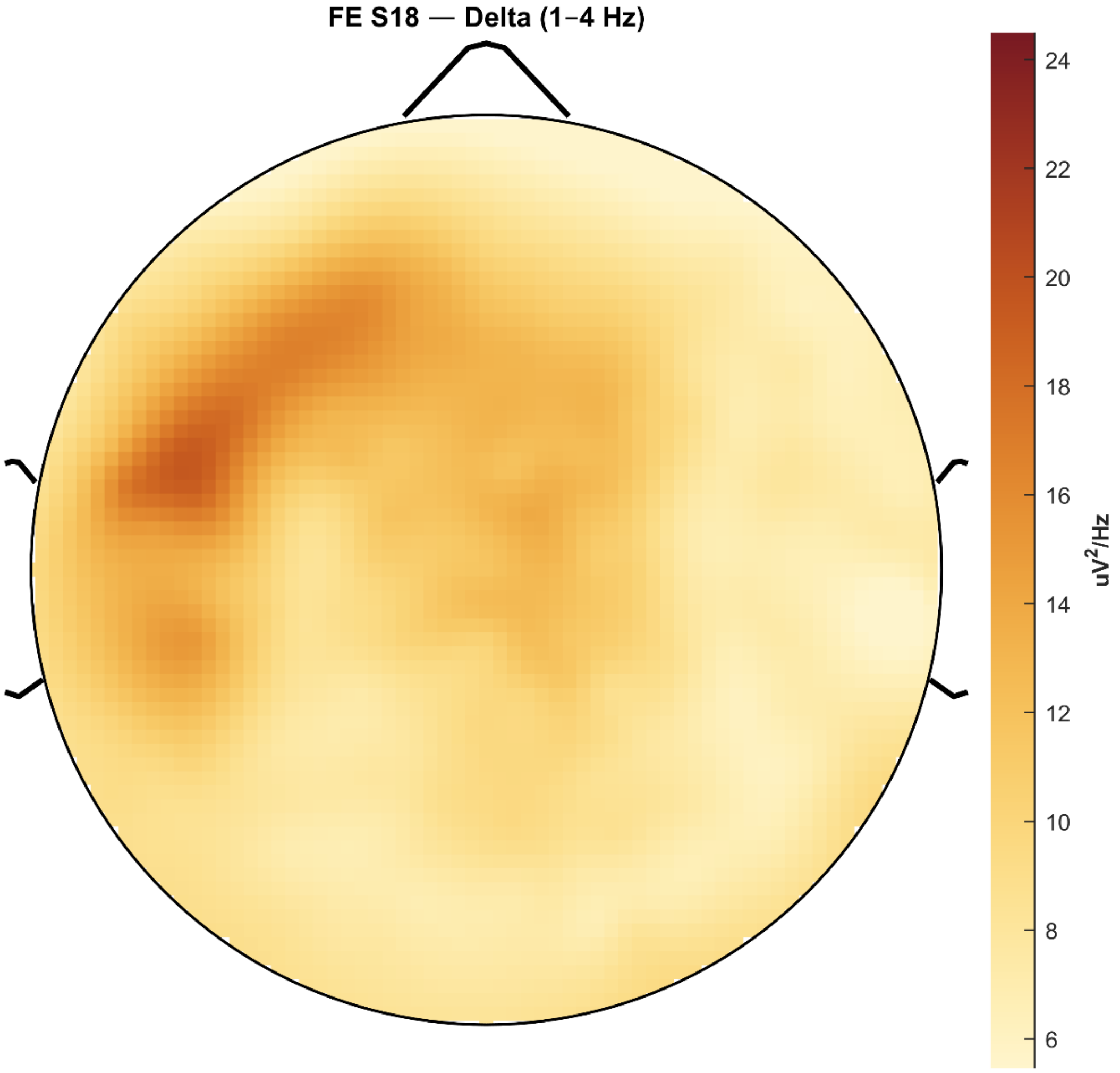

Supplementary Figure 19

Delta (1–4 Hz) power map for FE subject S18. The color scale represents delta power (μV²/Hz).

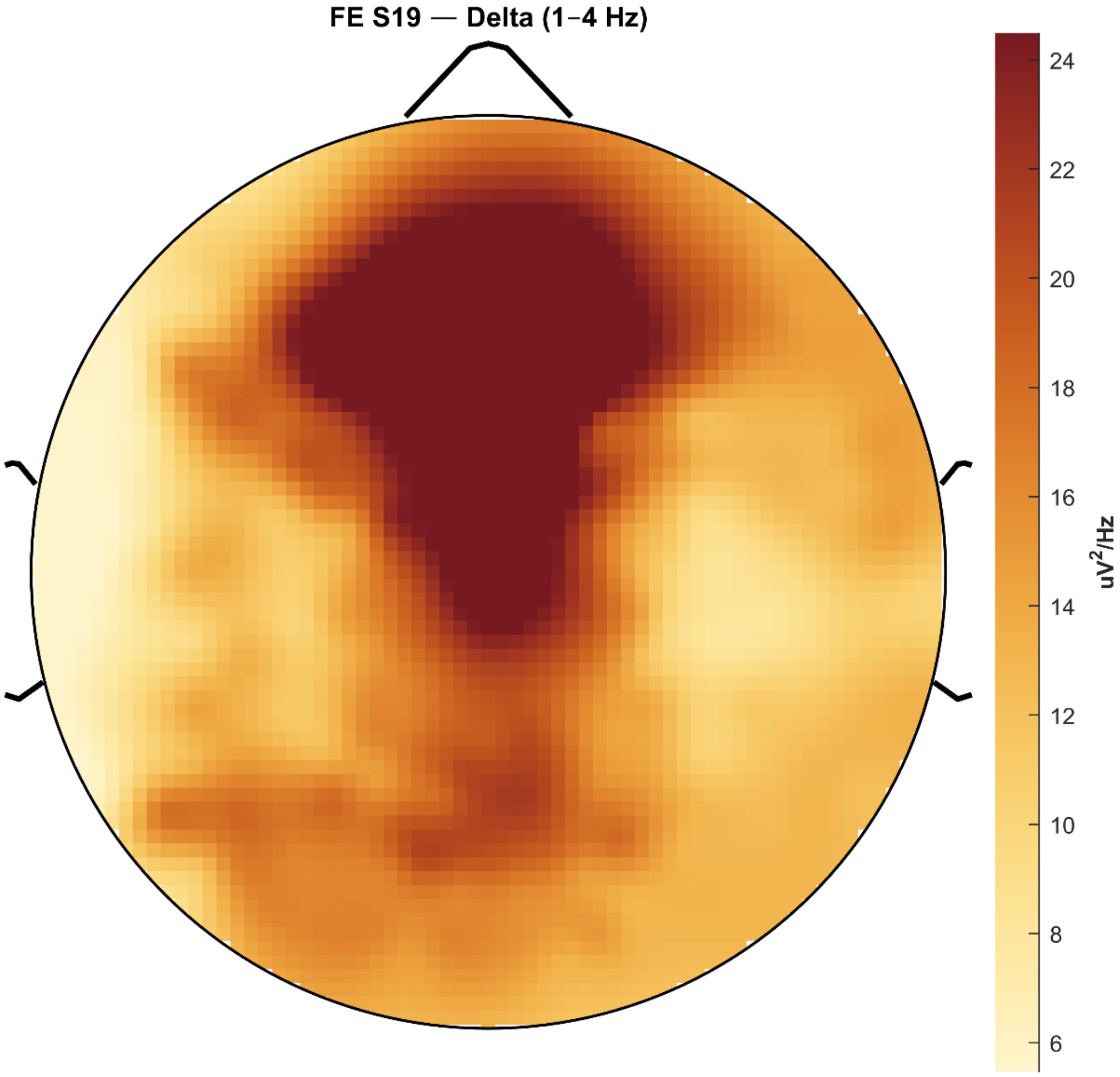

Supplementary Figure 20

Delta (1–4 Hz) power map for FE subject S19. The color scale represents delta power (µV²/Hz).

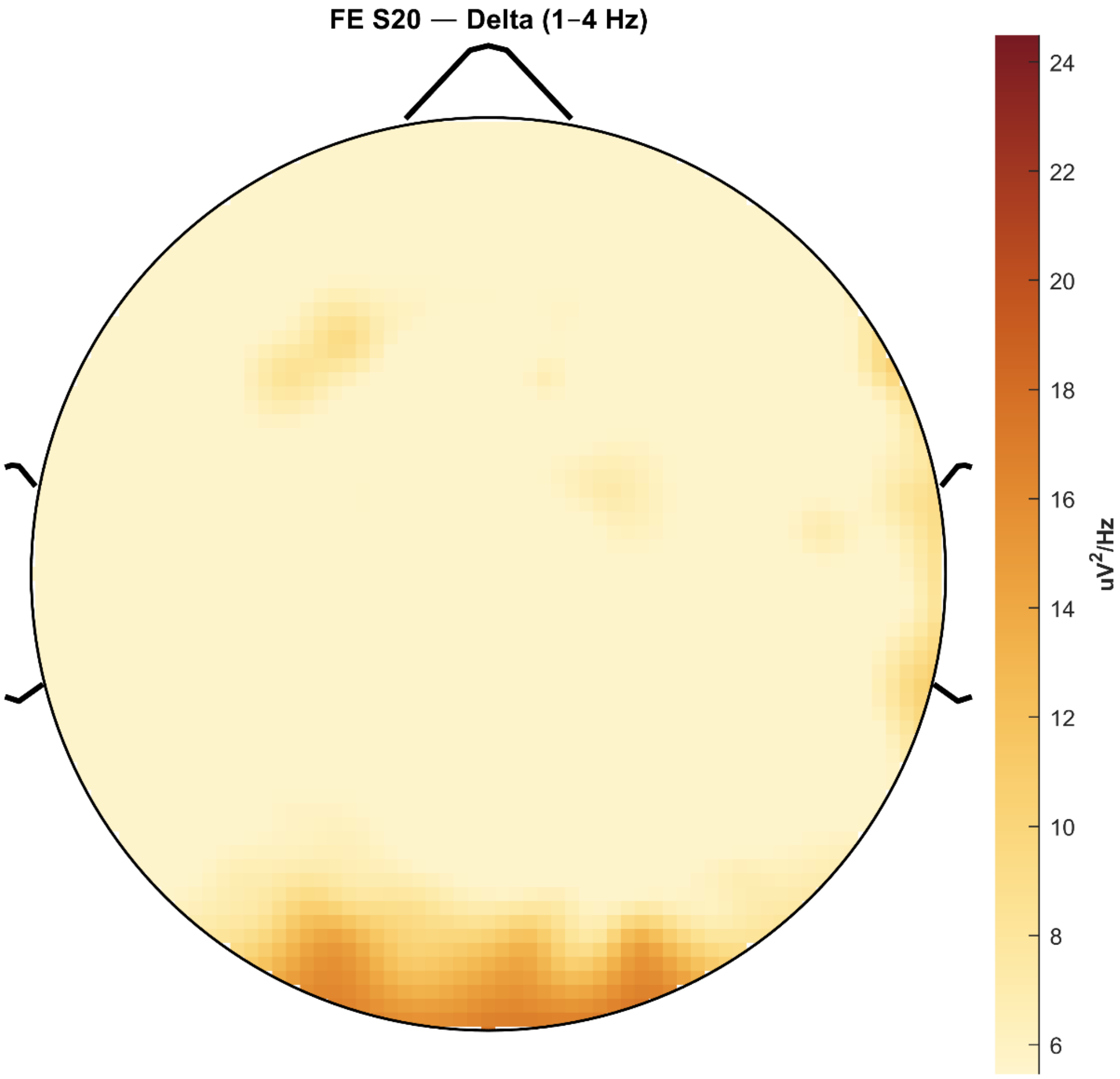

Supplementary Figure 21

Delta (1–4 Hz) power map for FE subject S20. The color scale represents delta power (µV²/Hz).

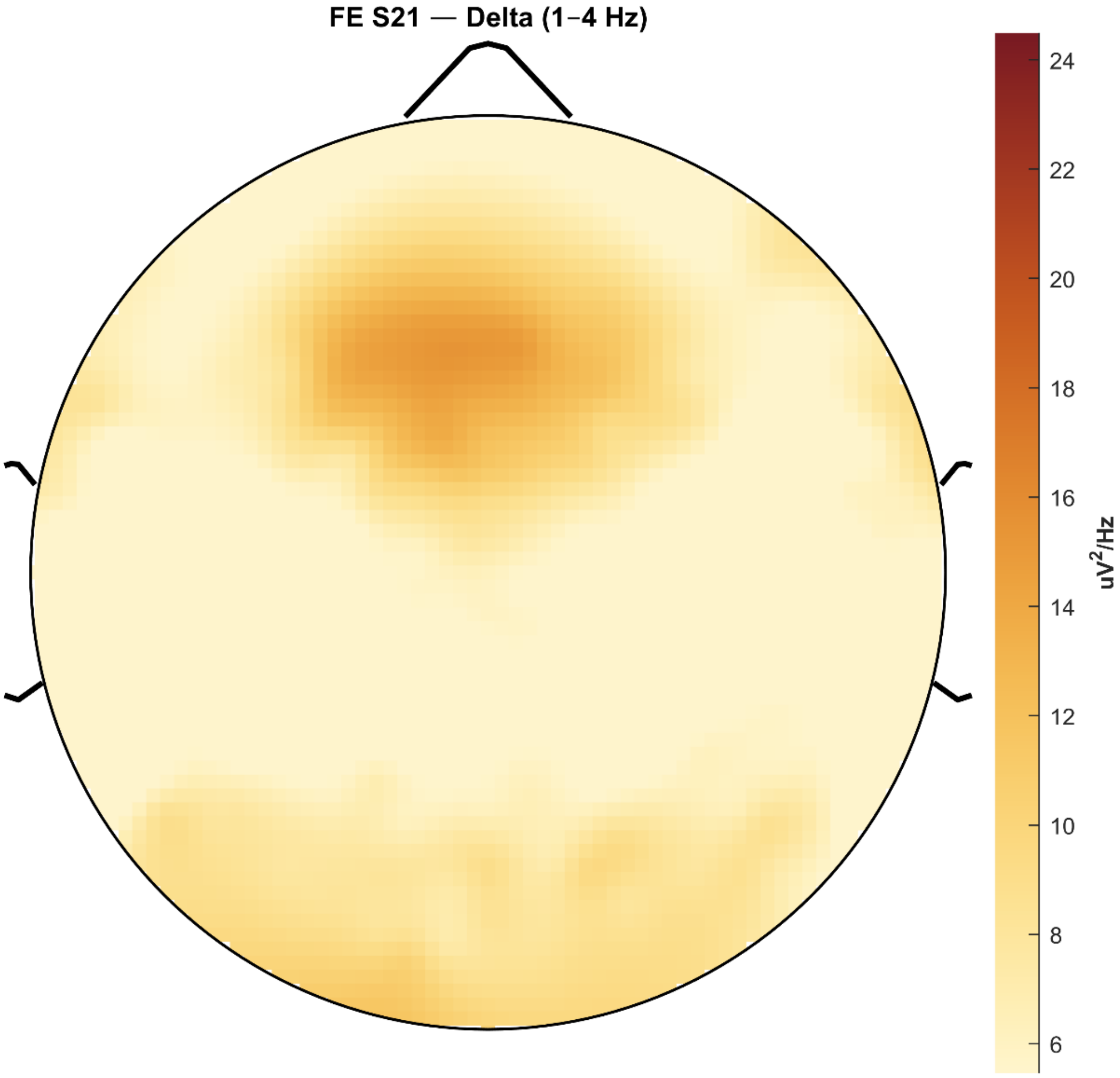

Supplementary Figure 22

Delta (1–4 Hz) power map for FE subject S21. The color scale represents delta power (µV²/Hz).

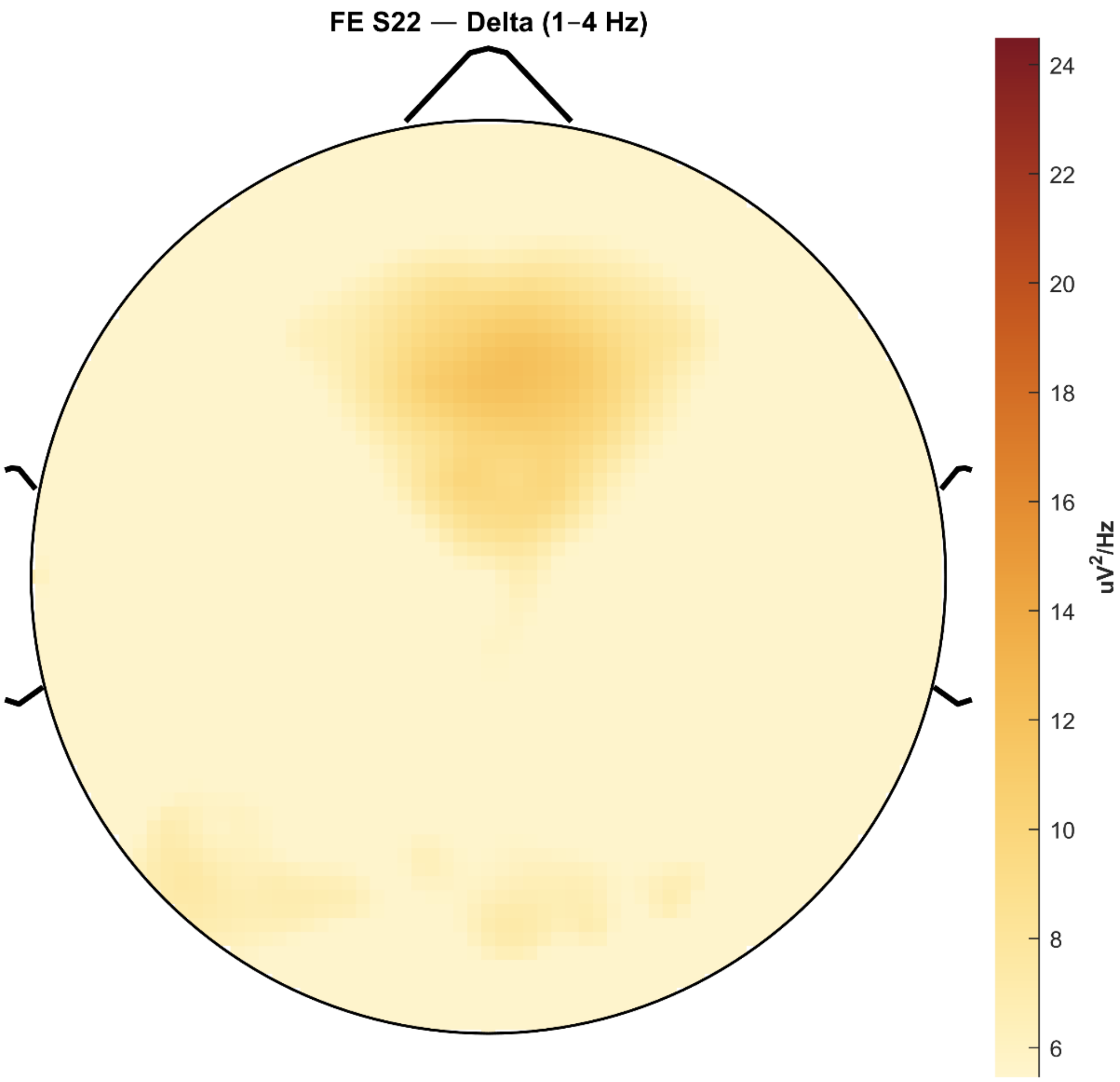

Supplementary Figure 23

Delta (1–4 Hz) power map for FE subject S22. The color scale represents delta power (µV²/Hz).

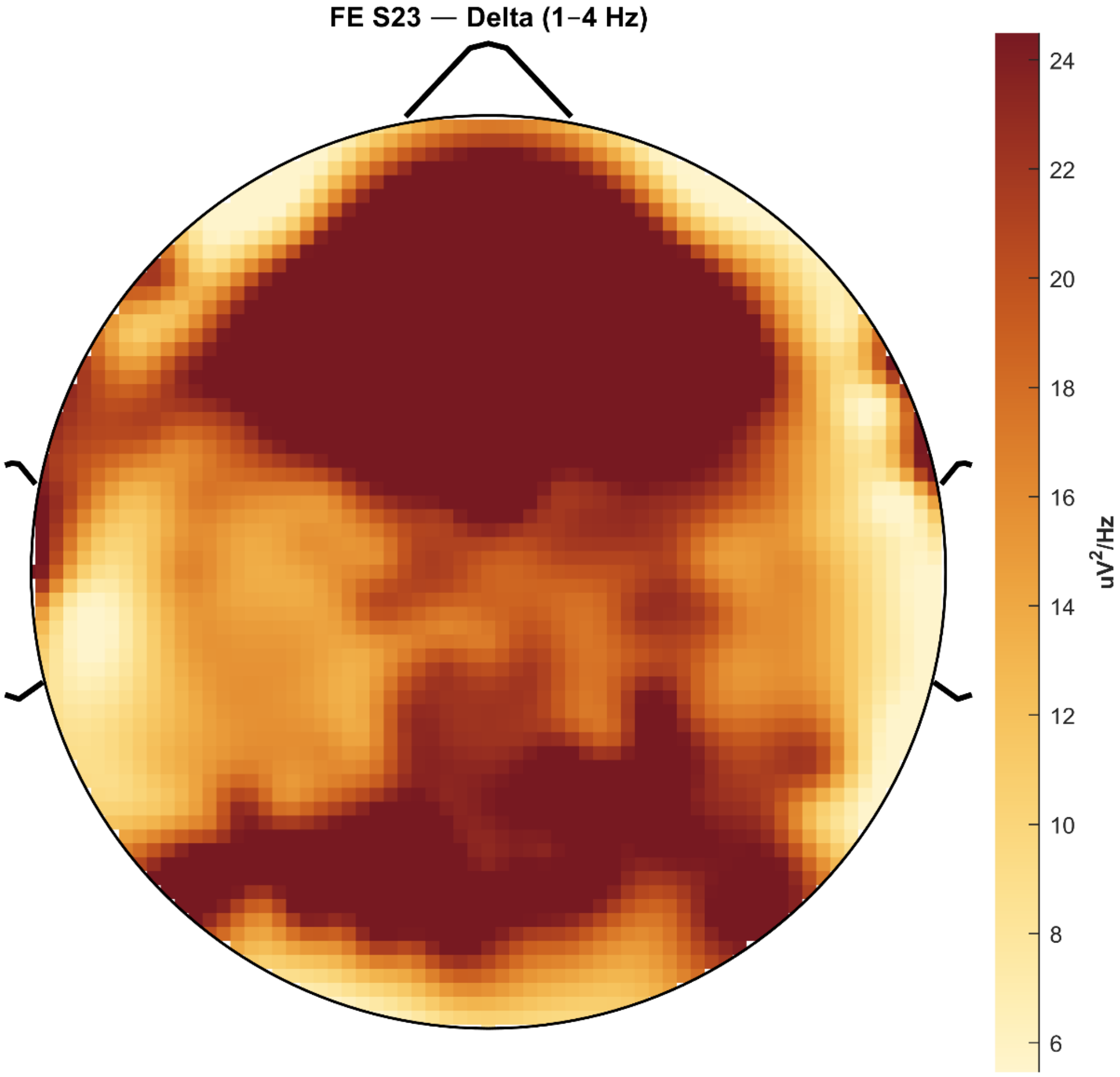

Supplementary Figure 24

Delta (1–4 Hz) power map for FE subject S23. The color scale represents delta power ($\mu V^2/Hz$).

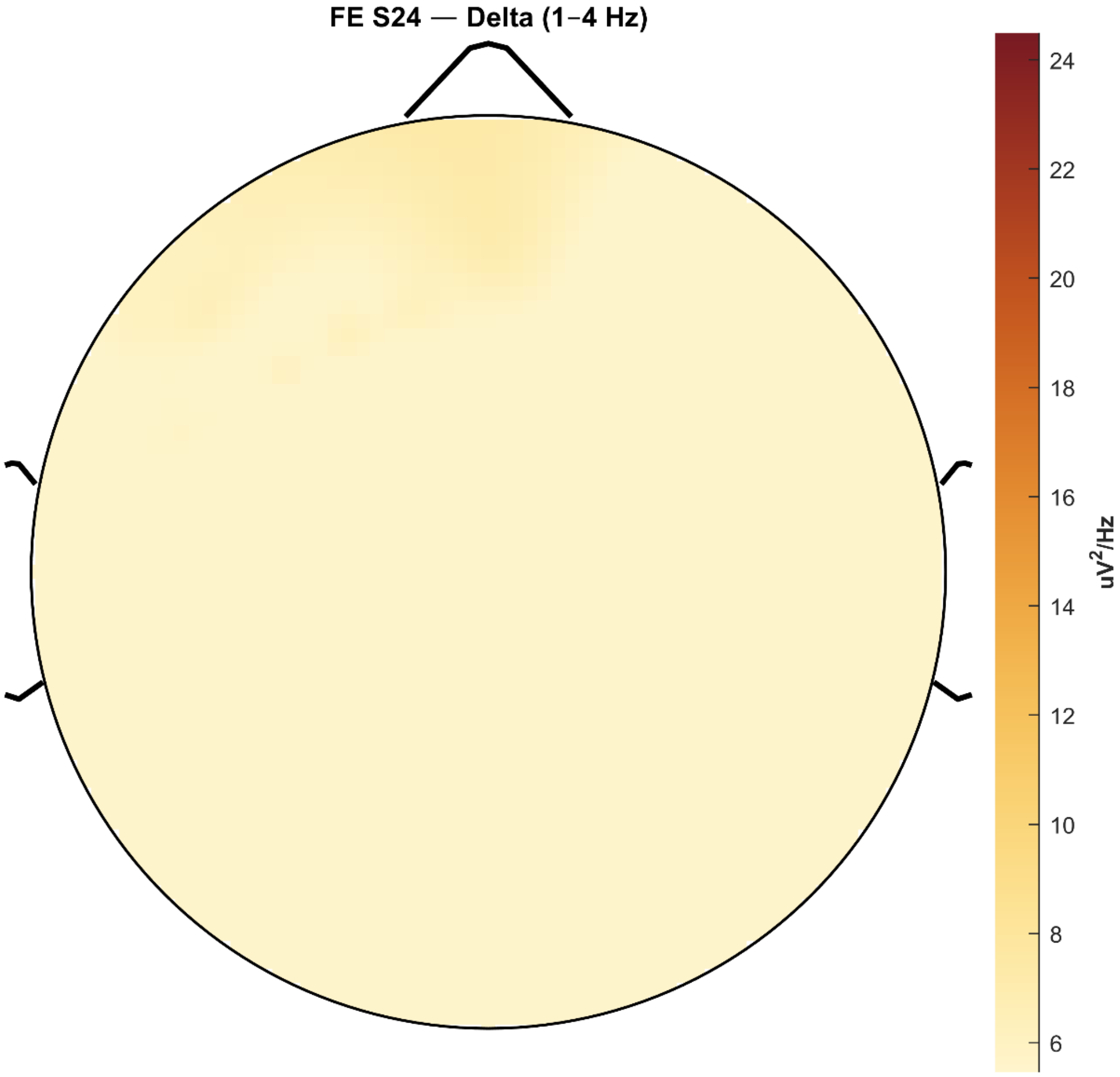

Supplementary Figure 25

Delta (1–4 Hz) power map for FE subject S24. The color scale represents delta power (μV²/Hz).

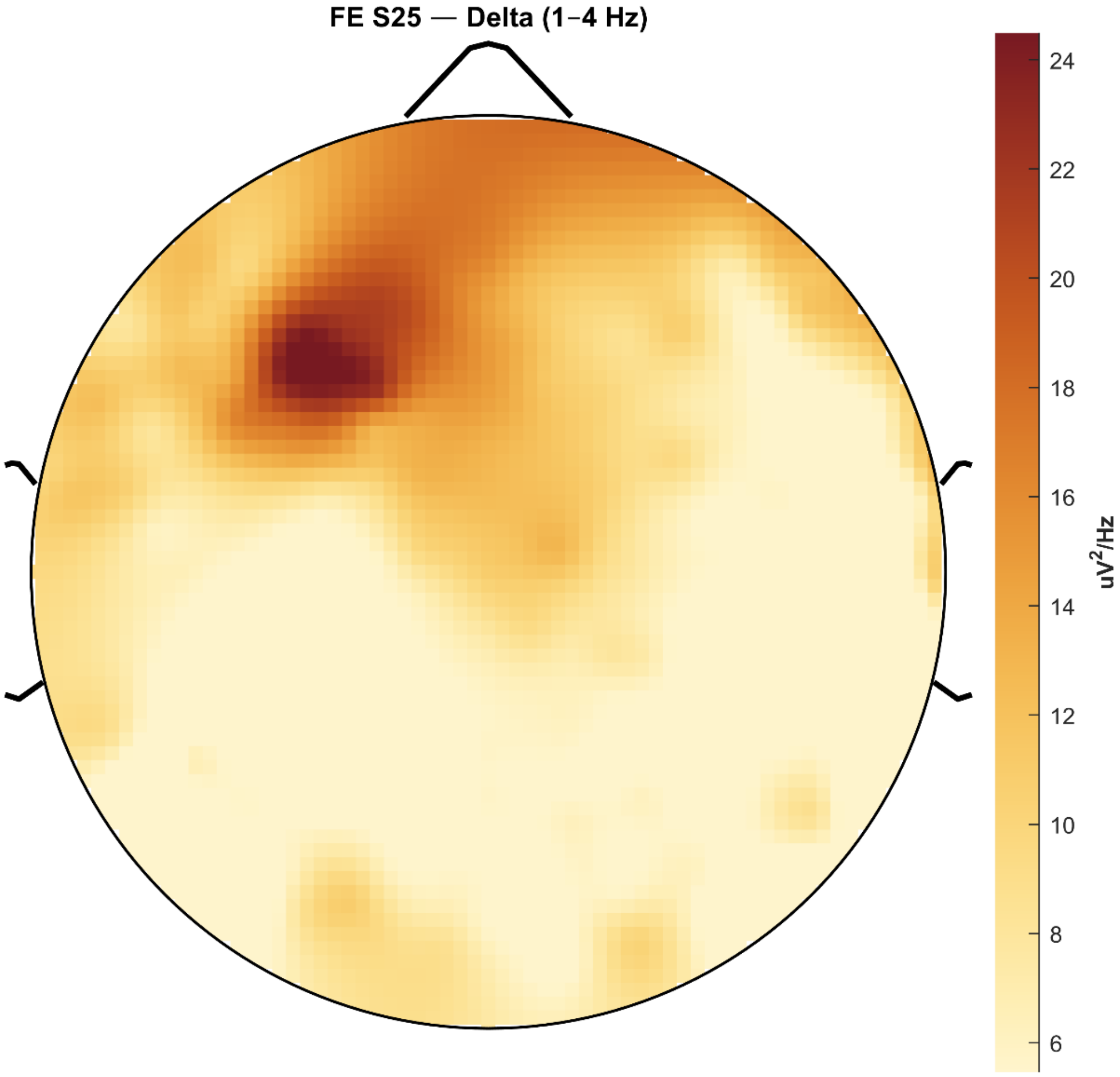

Supplementary Figure 26

Delta (1–4 Hz) power map for FE subject S25. The color scale represents delta power (µV²/Hz).

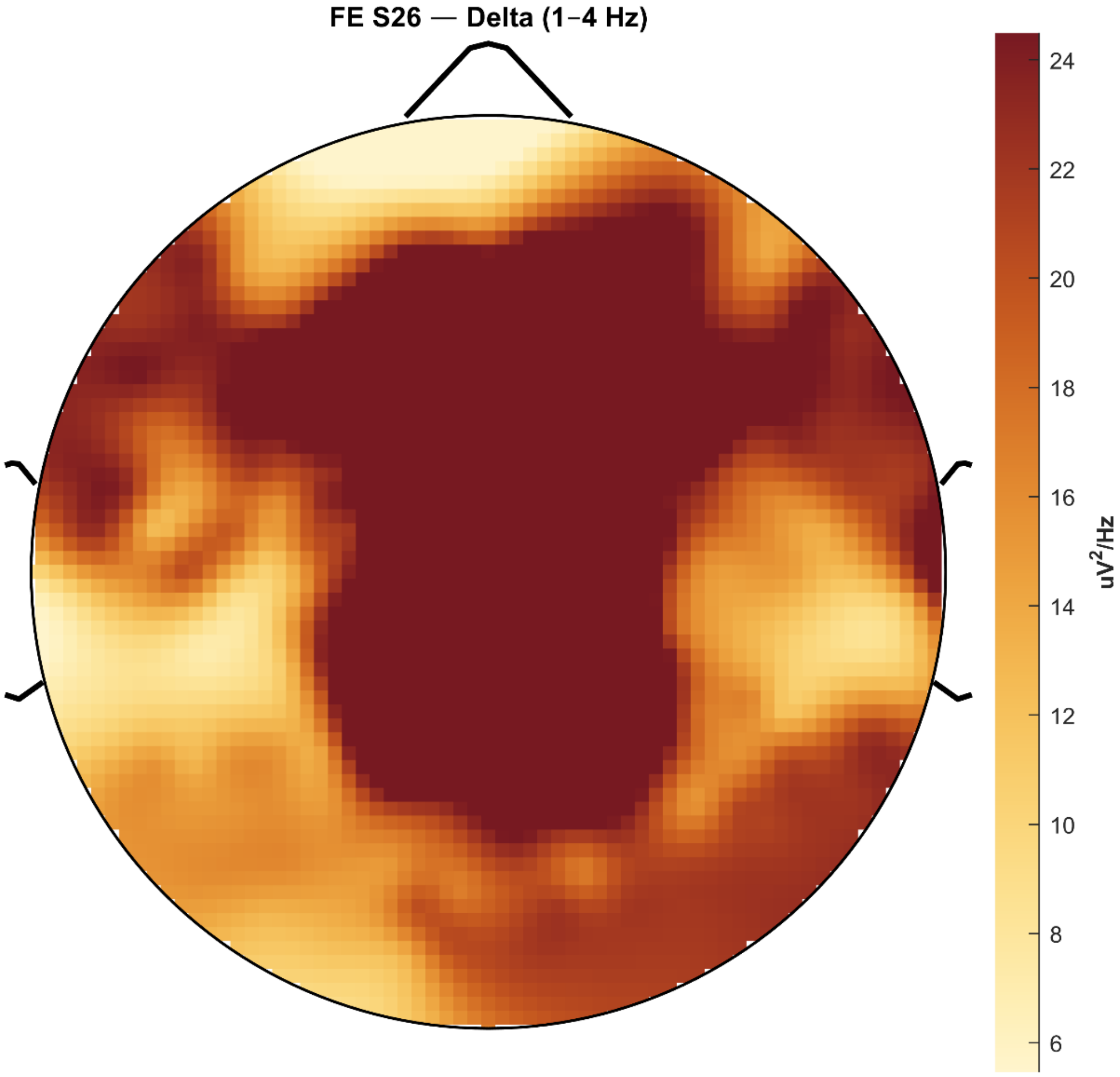

Supplementary Figure 27

Delta (1–4 Hz) power map for FE subject S26. The color scale represents delta power (μV²/Hz).

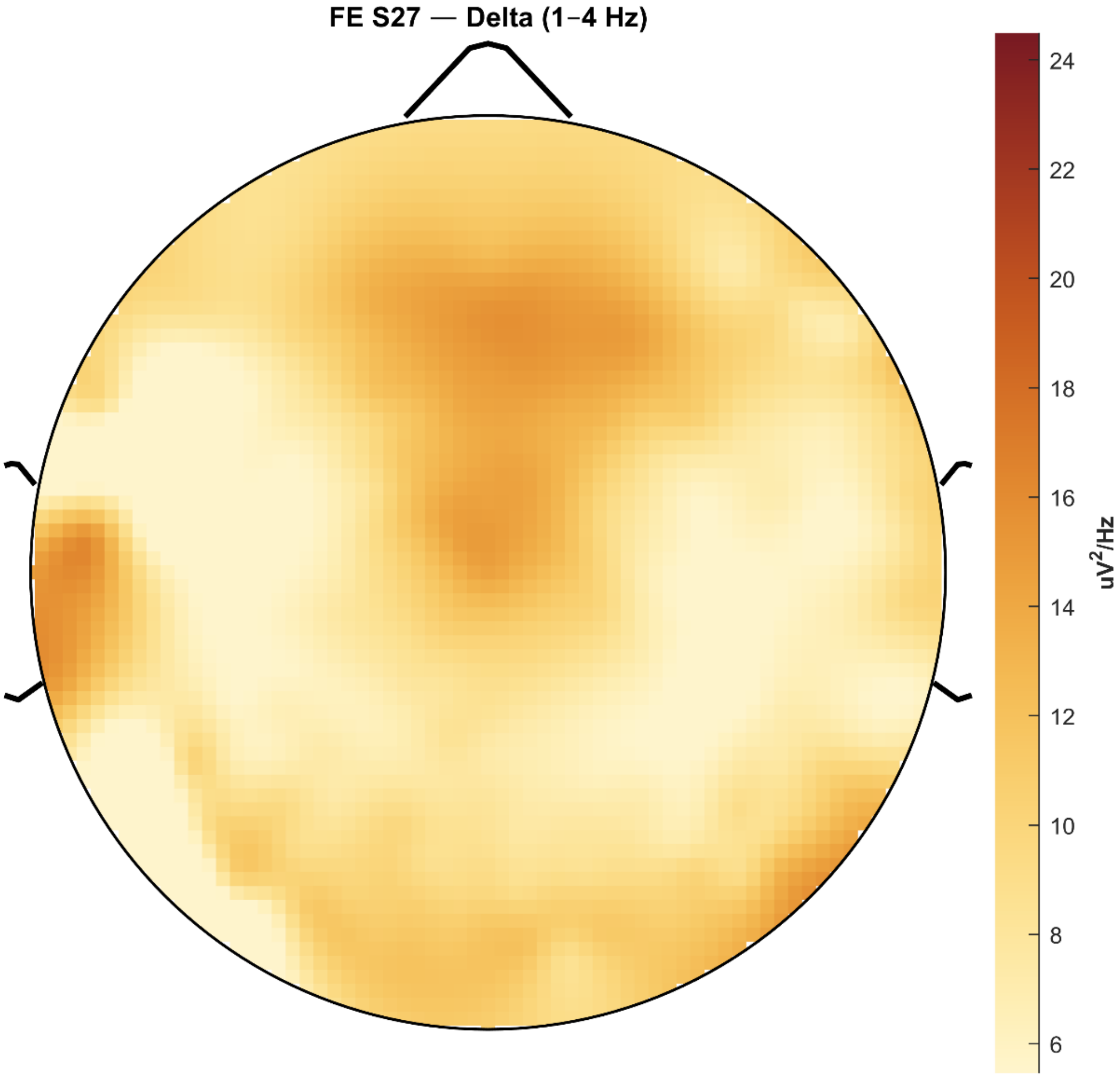

Supplementary Figure 28

Delta (1–4 Hz) power map for FE subject S27. The color scale represents delta power (μV²/Hz).

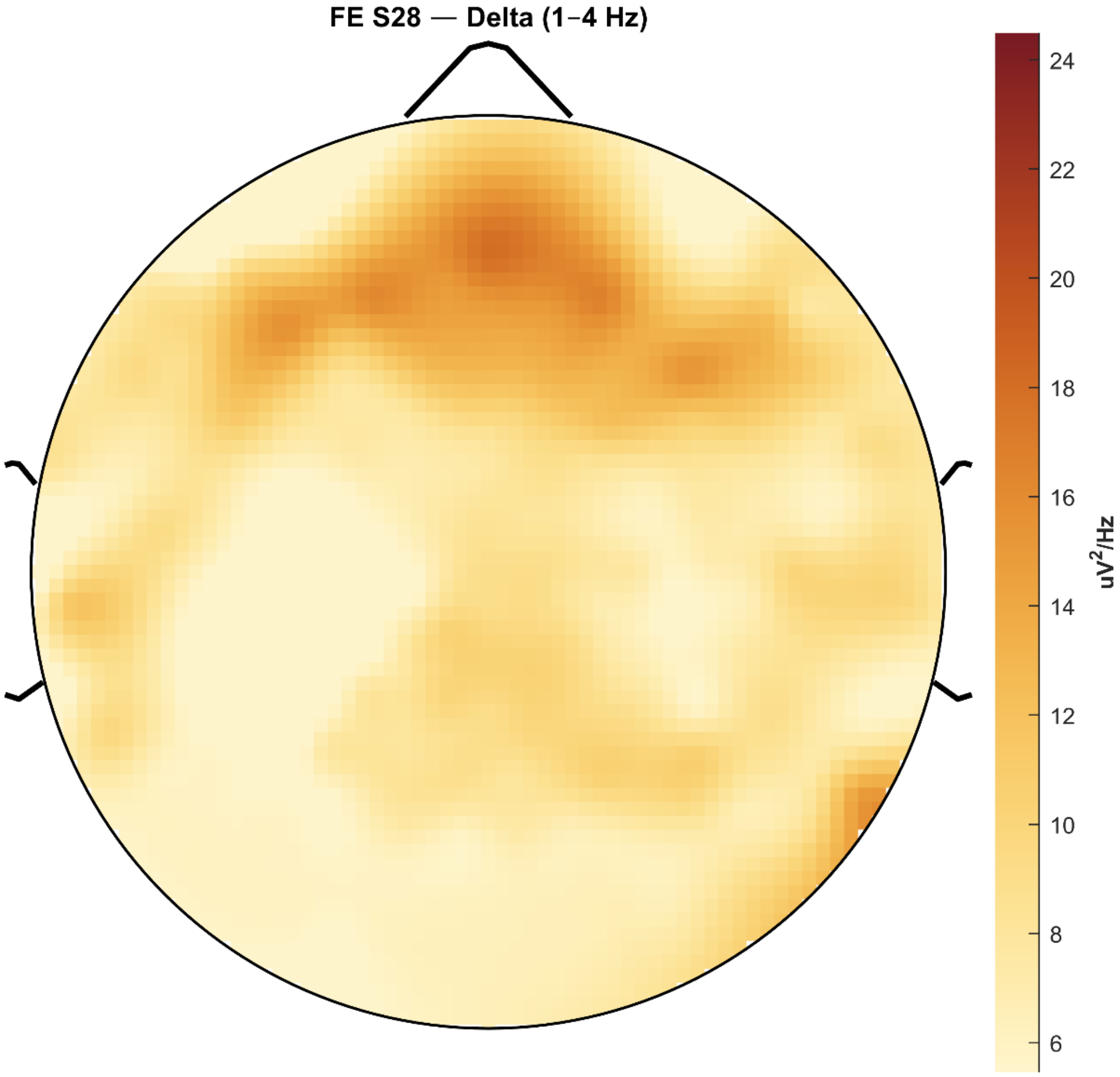

Supplementary Figure 29

Delta (1–4 Hz) power map for FE subject S28. The color scale represents delta power (µV²/Hz).

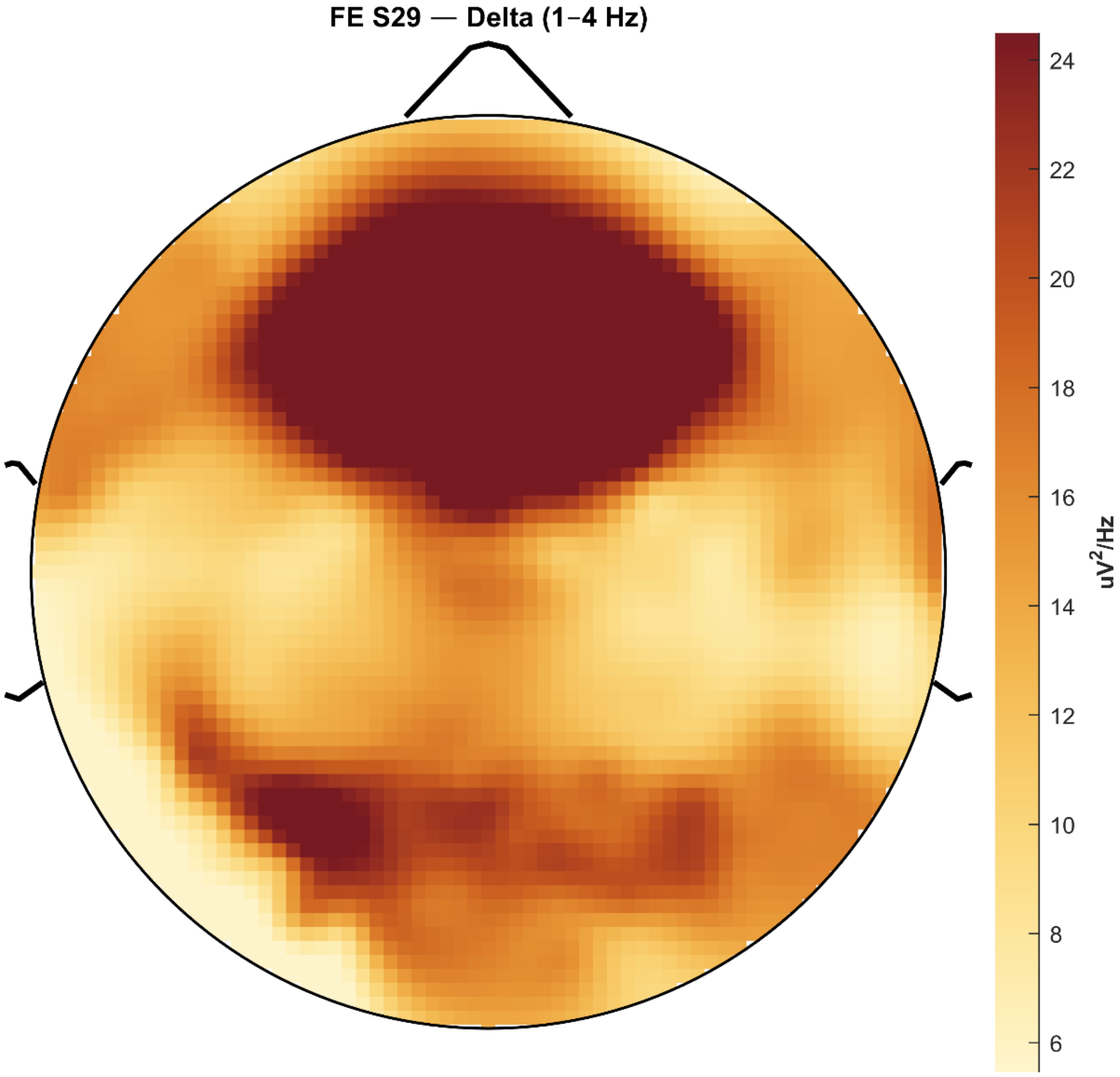

Supplementary Figure 30

Delta (1–4 Hz) power map for FE subject S29. The color scale represents delta power (µV²/Hz).

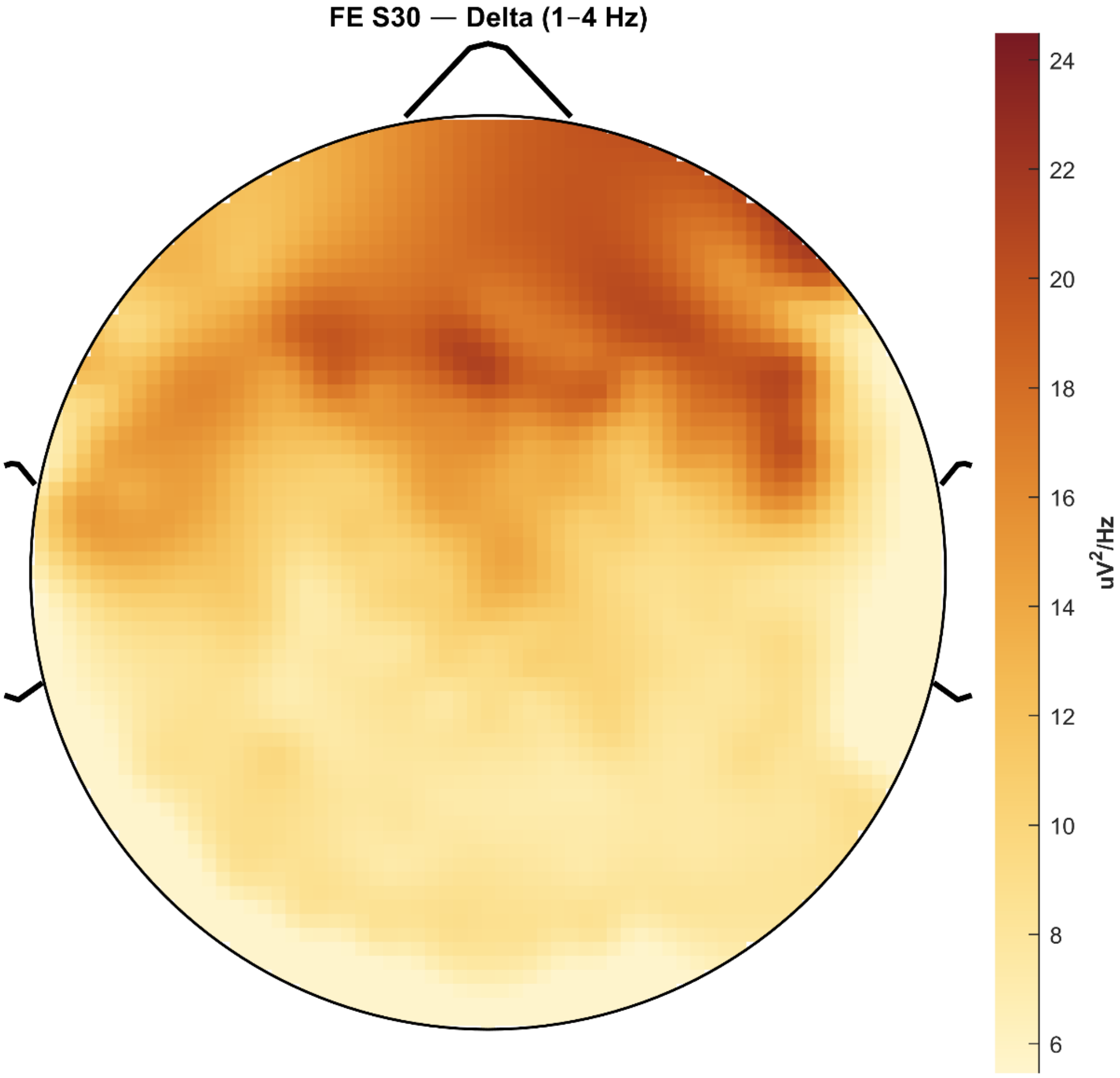

Supplementary Figure 31

Delta (1–4 Hz) power map for FE subject S30. The color scale represents delta power (µV²/Hz).

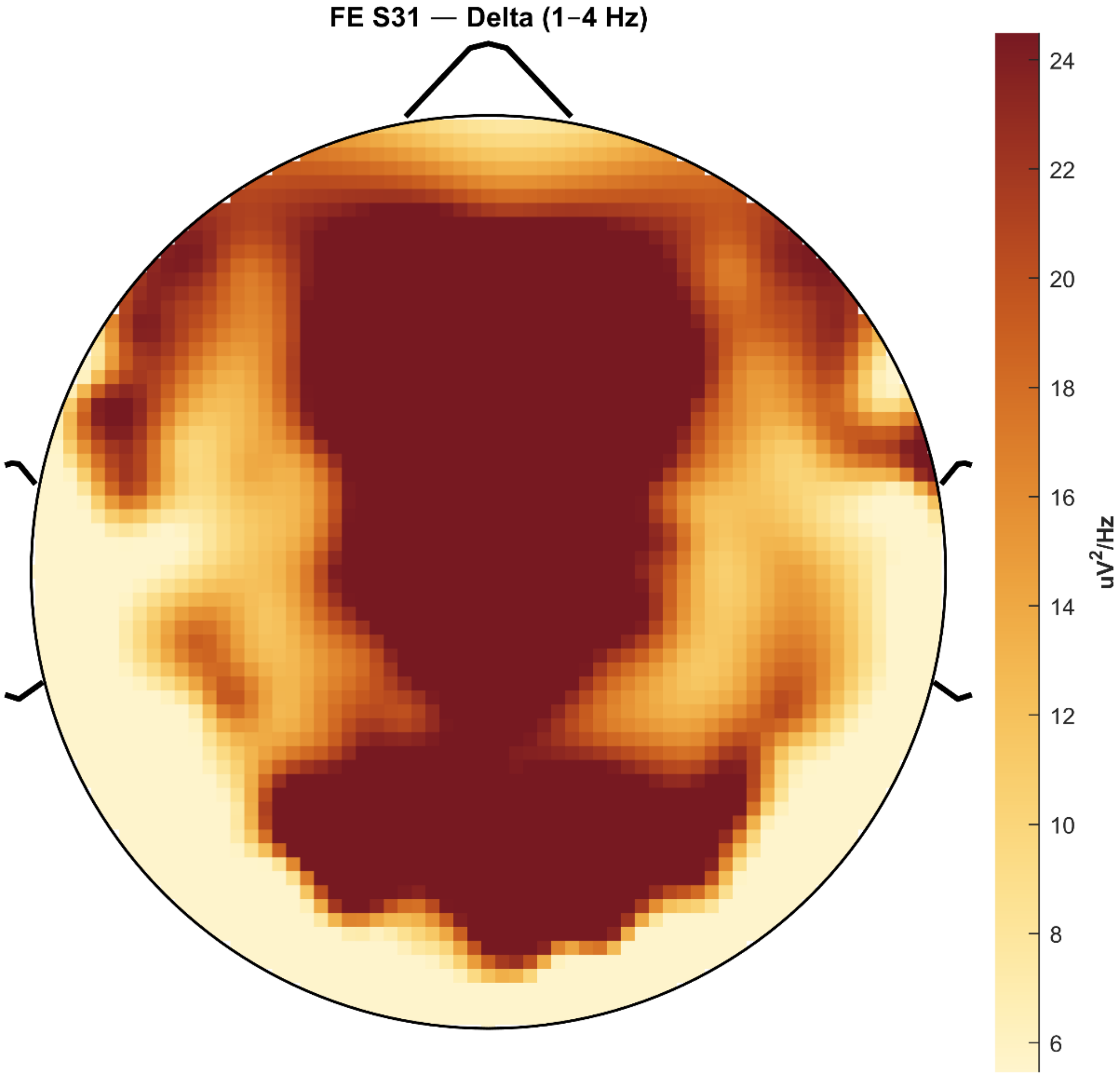

Supplementary Figure 32

Delta (1–4 Hz) power map for FE subject S31. The color scale represents delta power (µV²/Hz).

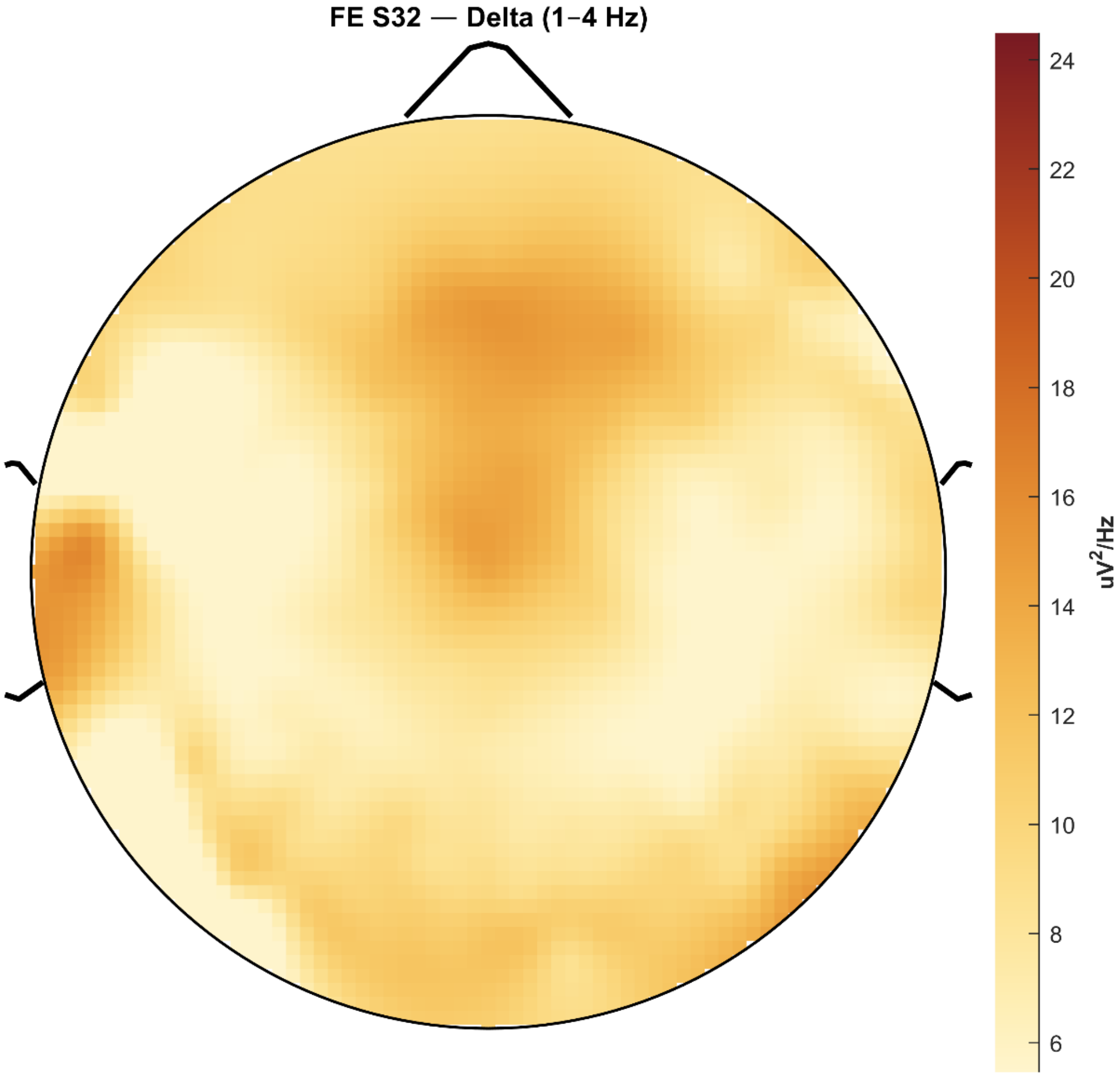

Supplementary Figure 33

Delta (1–4 Hz) power map for FE subject S32. The color scale represents delta power (µV²/Hz).

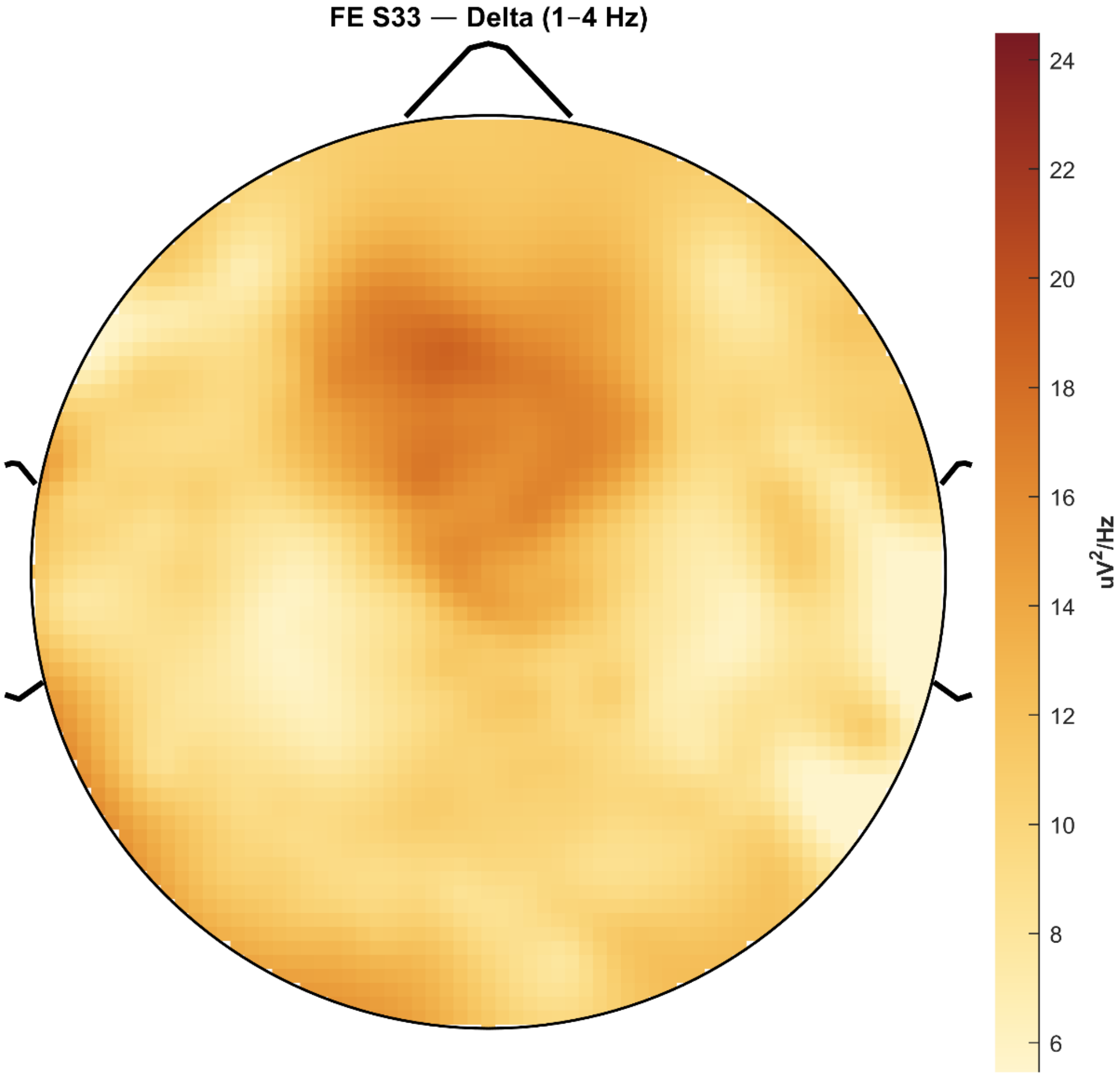

Supplementary Figure 34

Delta (1–4 Hz) power map for FE subject S33. The color scale represents delta power (μV²/Hz).

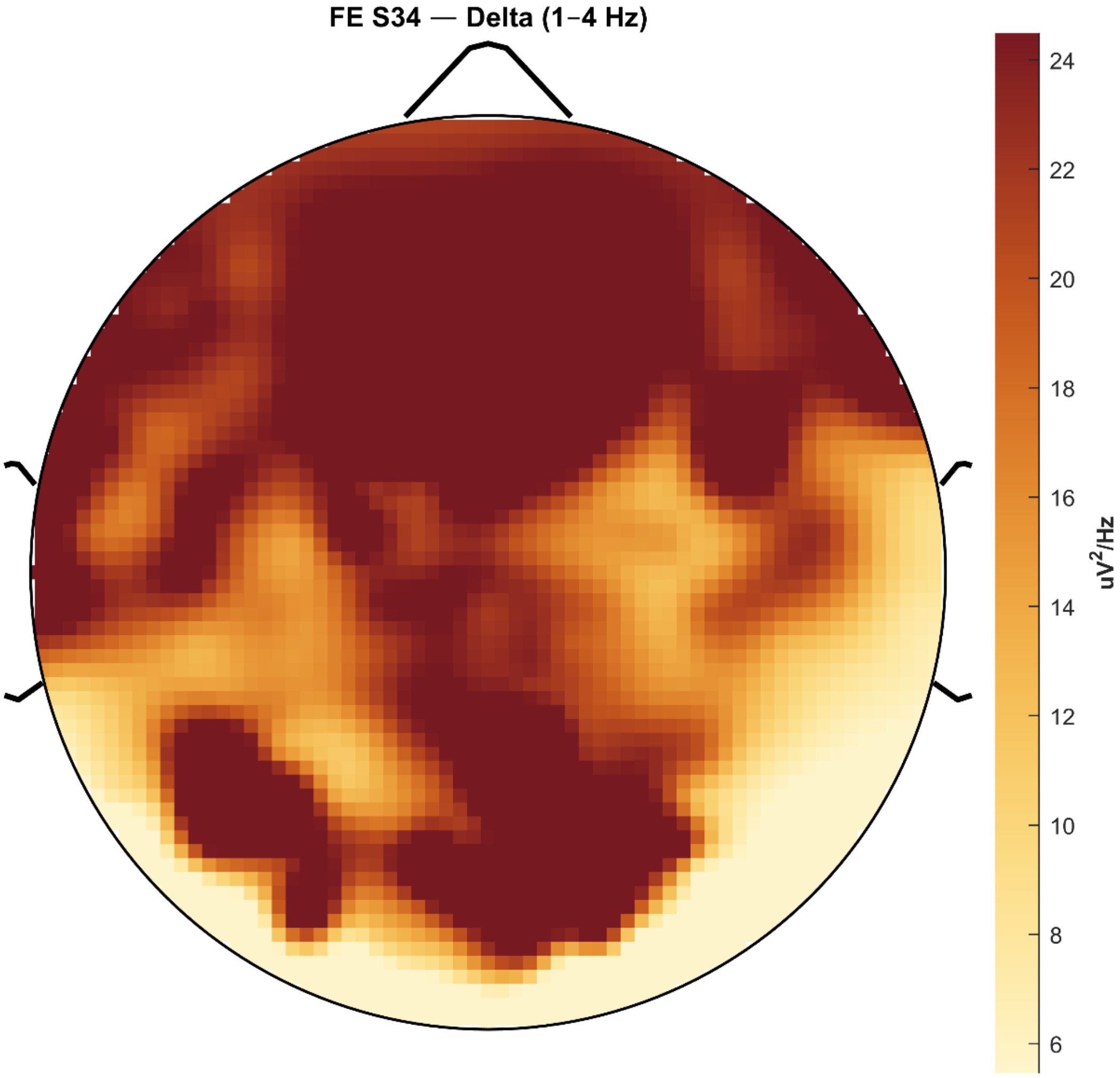

Supplementary Figure 35

Delta (1–4 Hz) power map for FE subject S34. The color scale represents delta power (µV²/Hz).

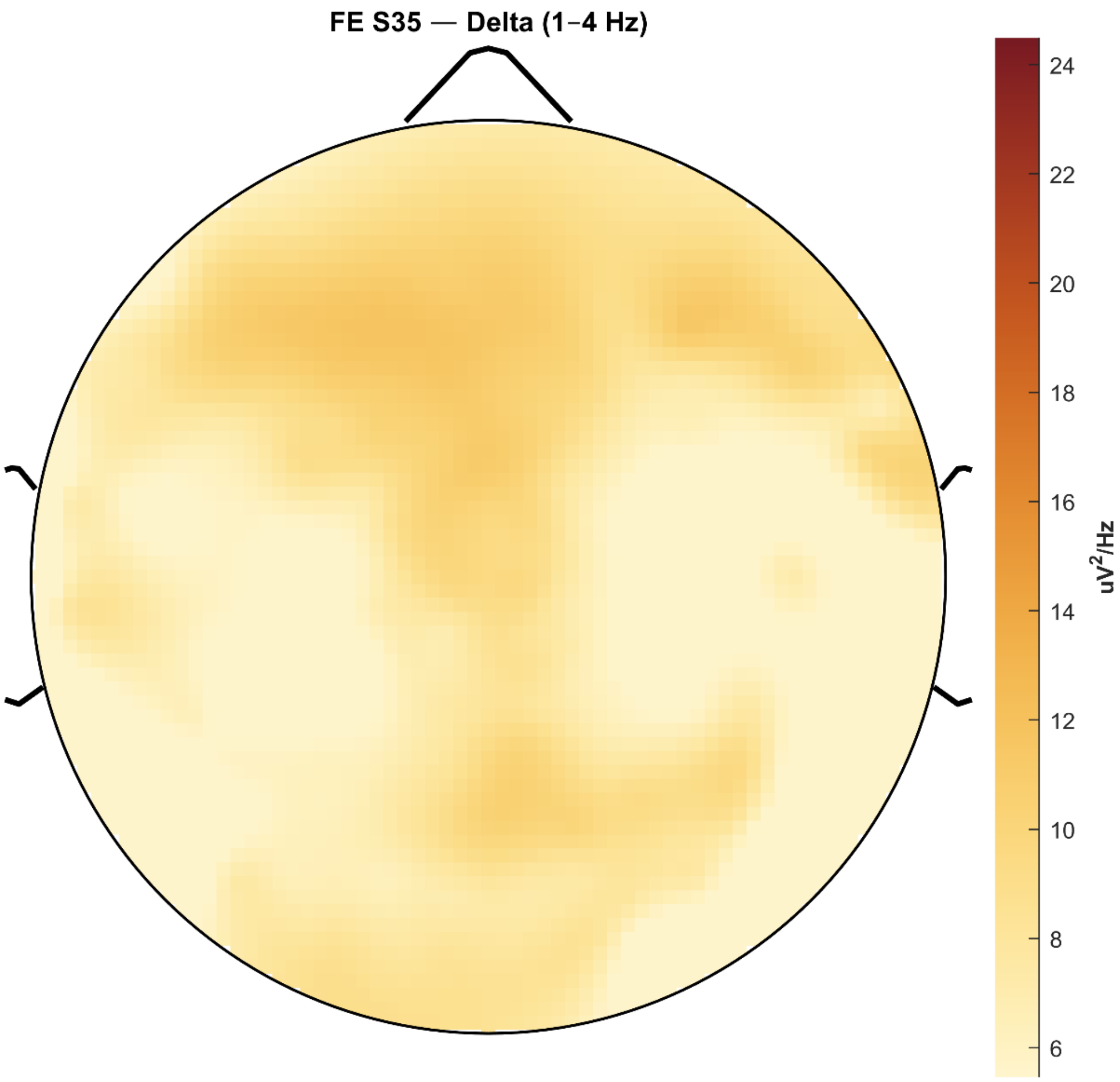

Supplementary Figure 36

Delta (1–4 Hz) power map for FE subject S35. The color scale represents delta power (µV²/Hz).

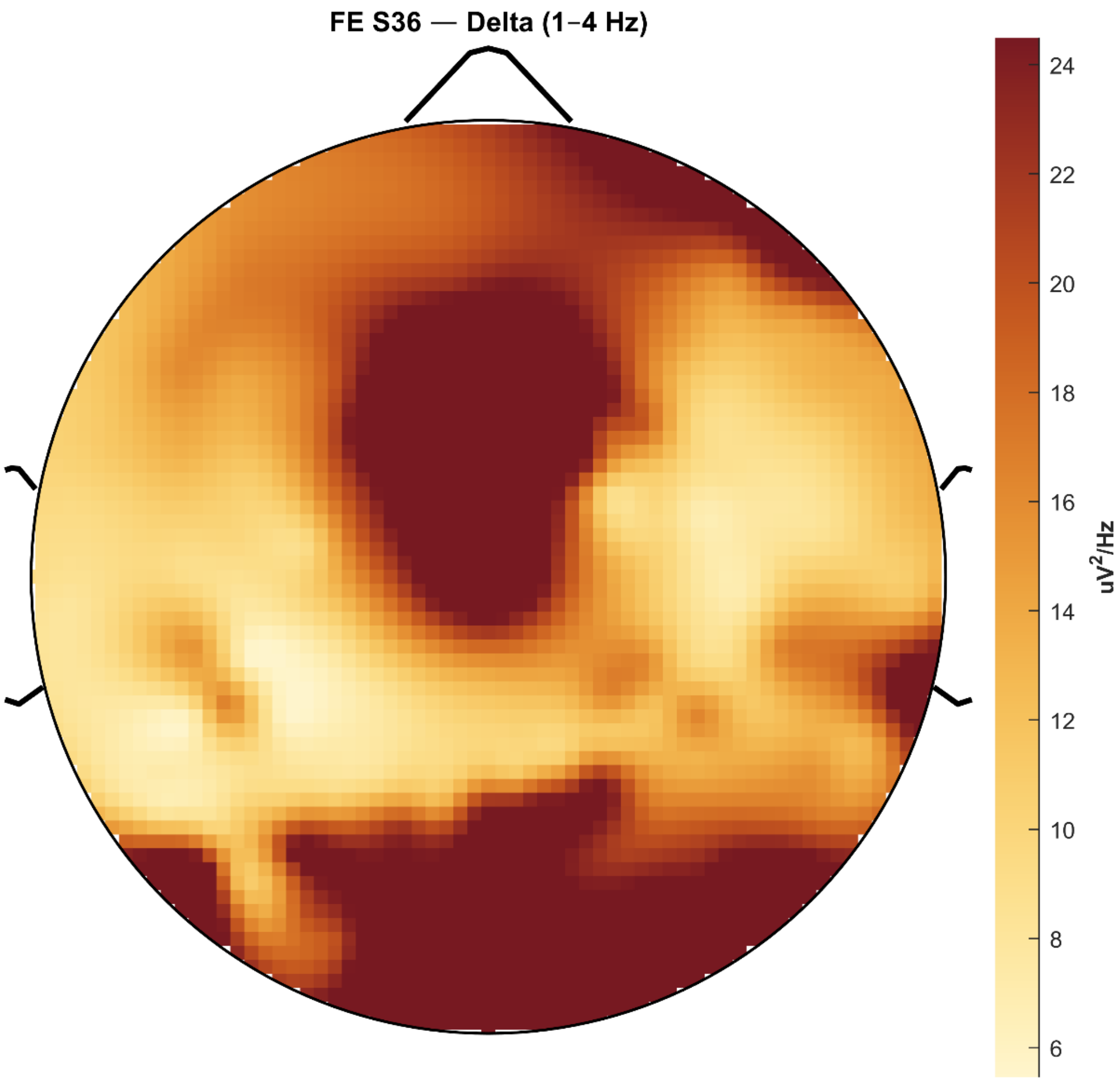

Supplementary Figure 37

Delta (1–4 Hz) power map for FE subject S36. The color scale represents delta power (µV²/Hz).

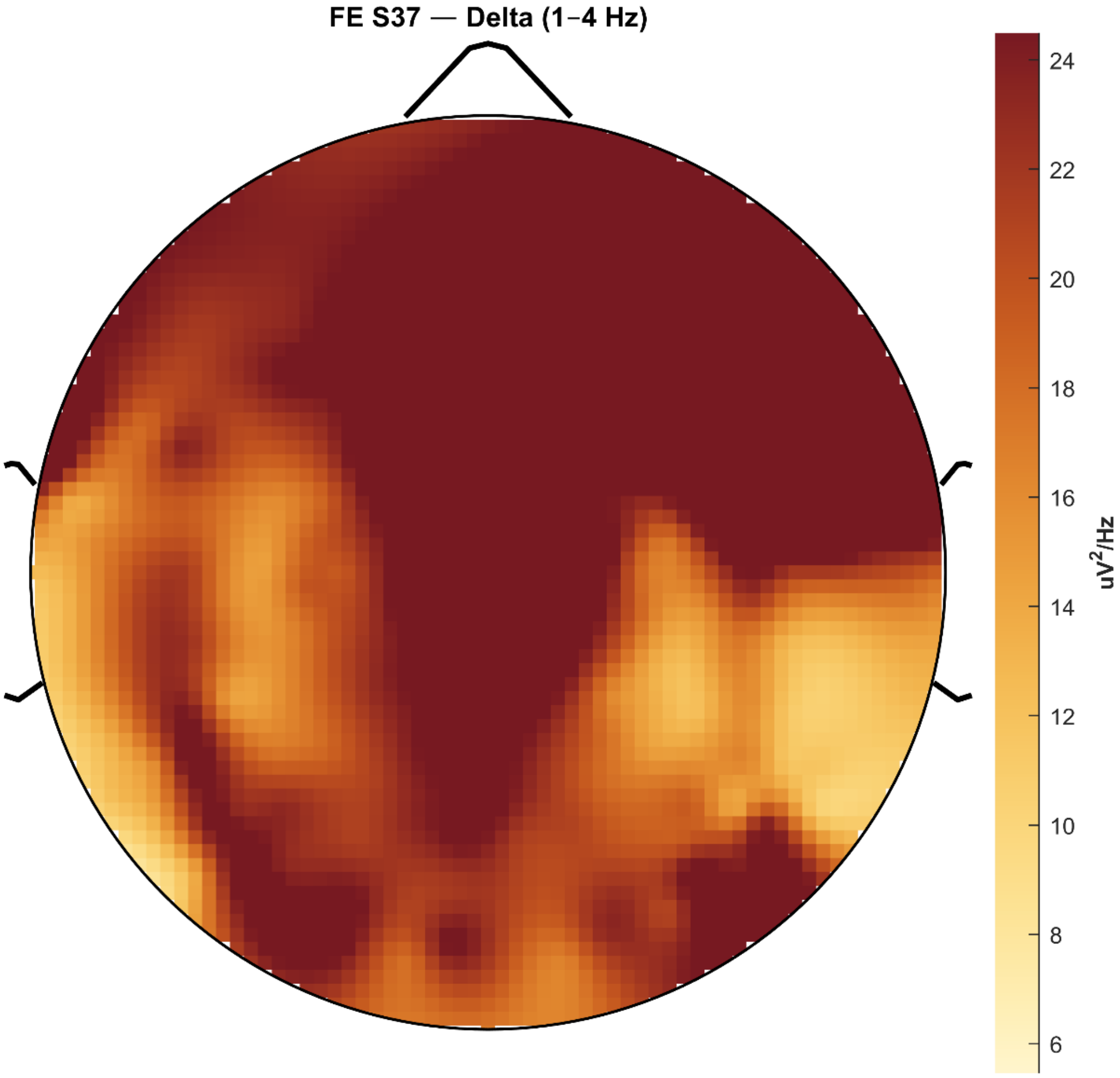

Supplementary Figure 38

Delta (1–4 Hz) power map for FE subject S37. The color scale represents delta power (µV²/Hz).

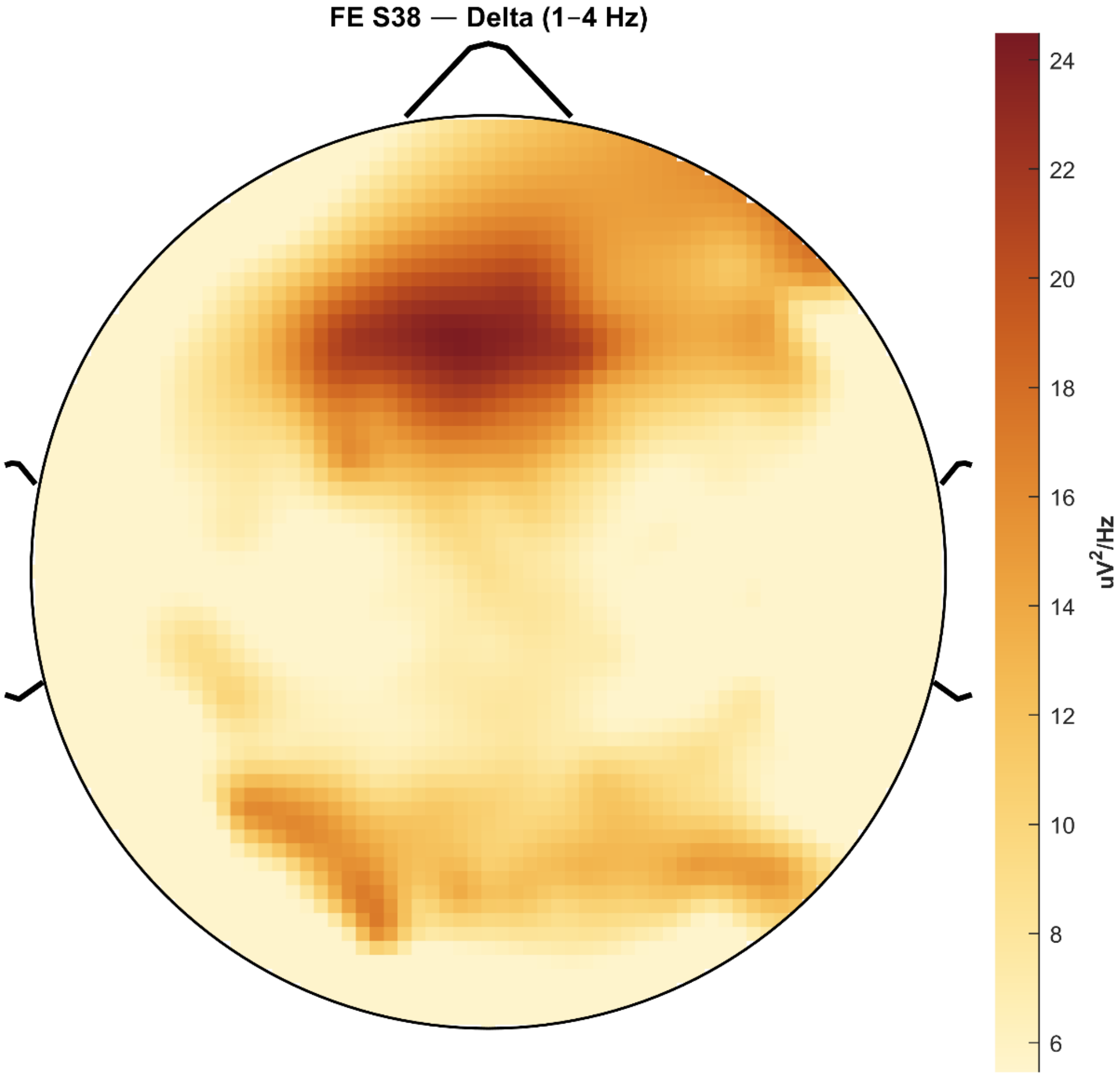

Supplementary Figure 39

Delta (1–4 Hz) power map for FE subject S38. The color scale represents delta power (µV²/Hz).

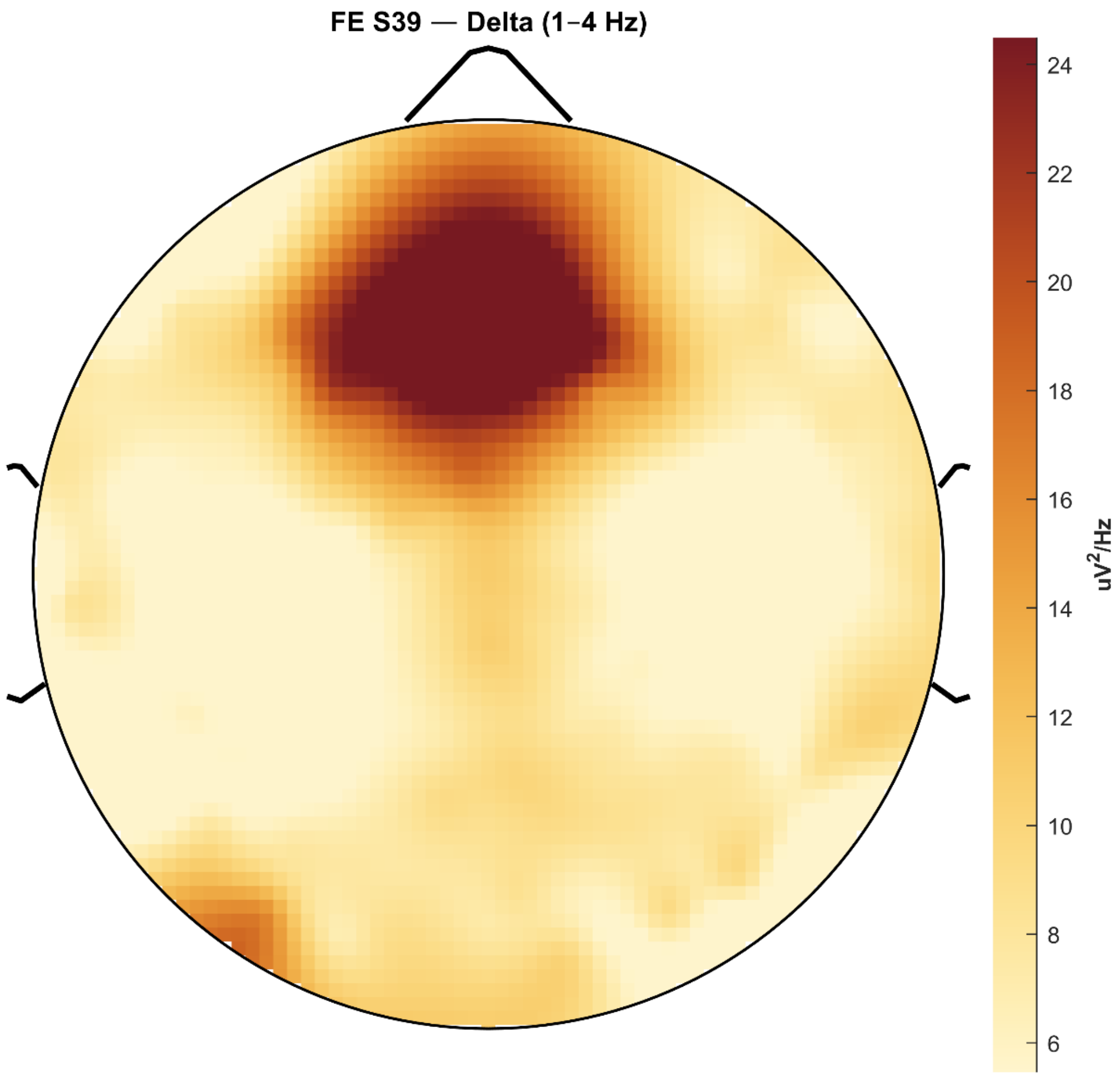

Supplementary Figure 40

Delta (1–4 Hz) power map for FE subject S39. The color scale represents delta power (μV²/Hz).

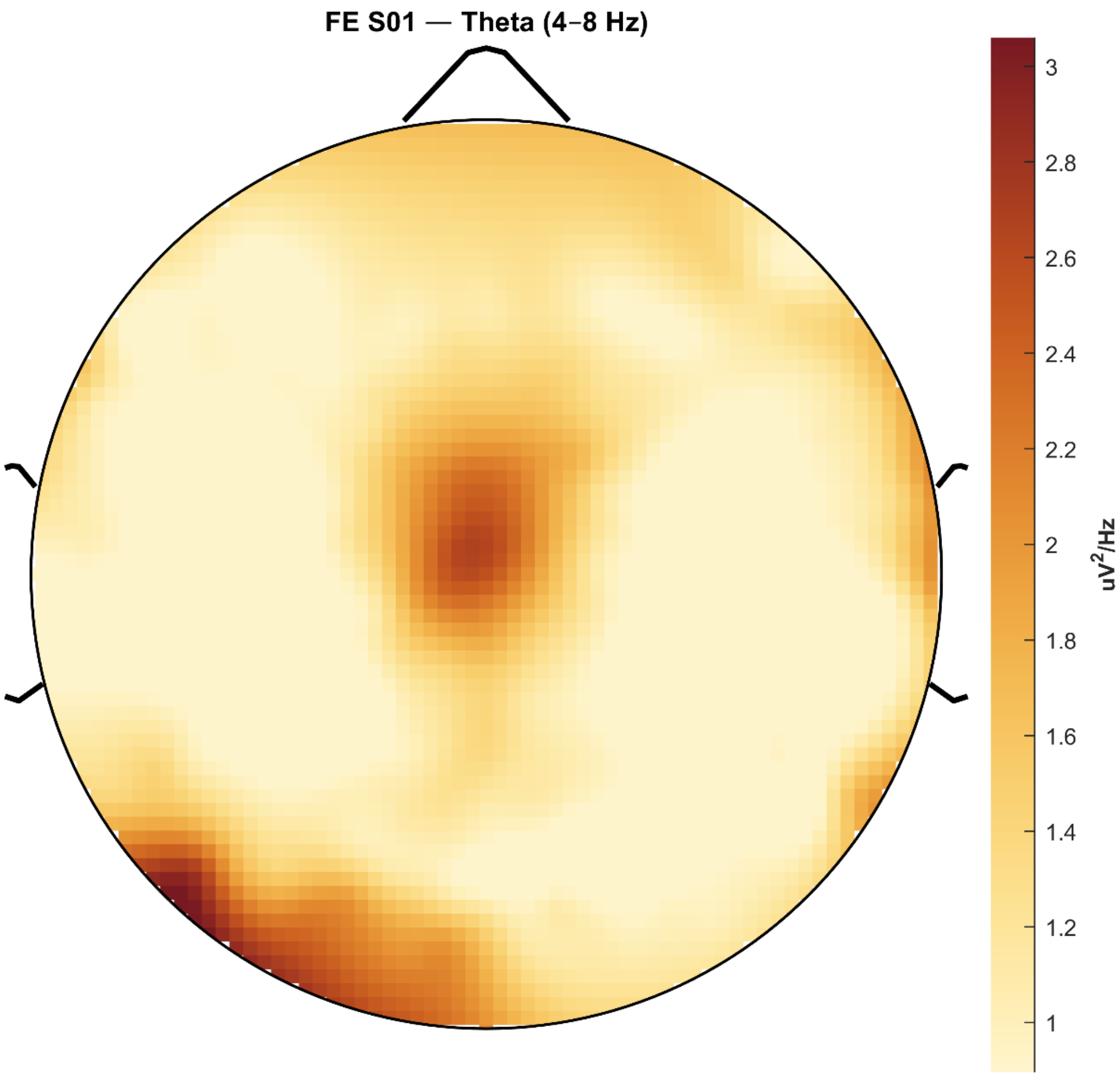

Supplementary Figure 41

Theta (4–8 Hz) power map for FE subject S01. The color scale represents theta power (μV²/Hz).

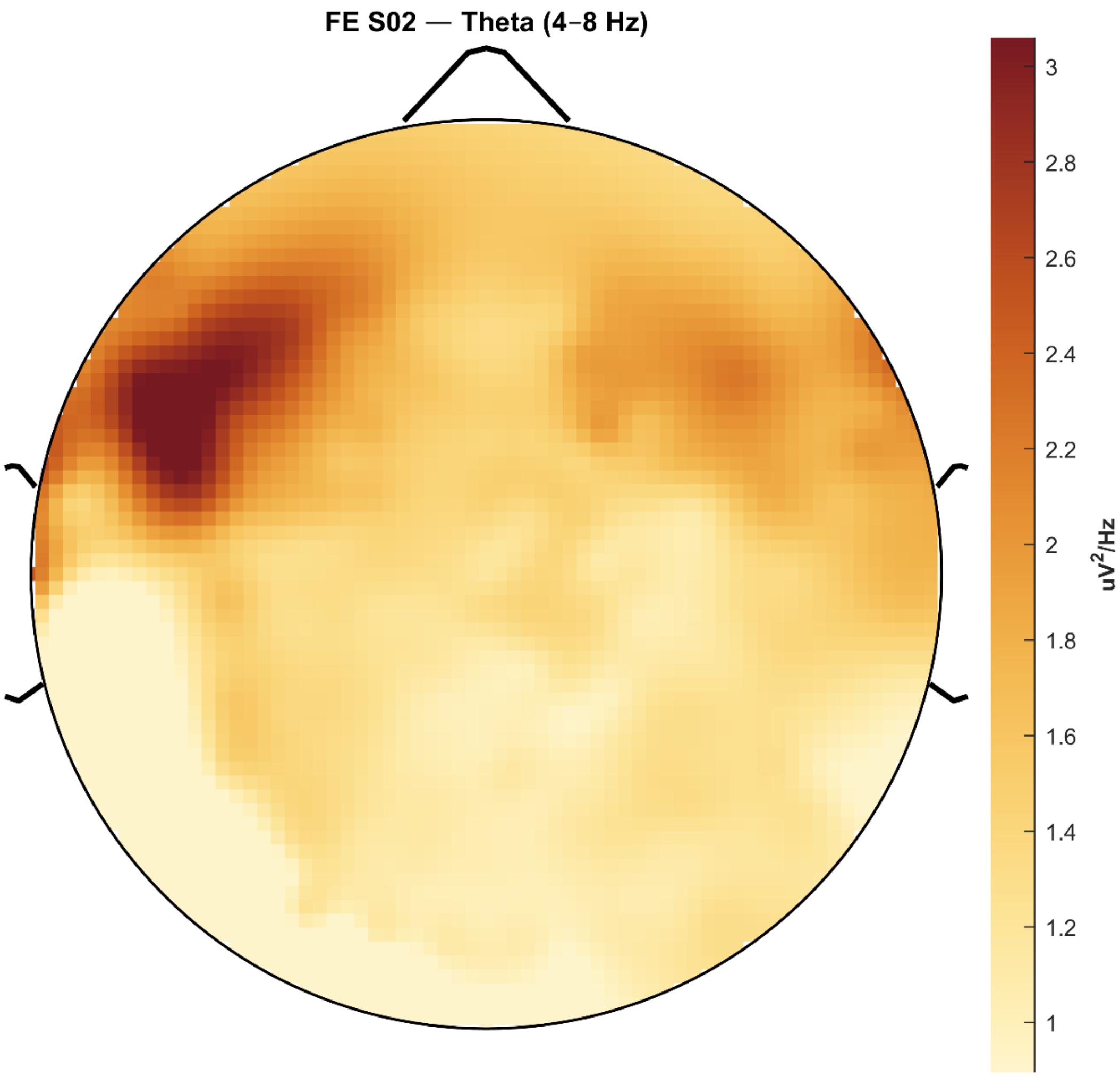

Supplementary Figure 42

Theta (4–8 Hz) power map for FE subject S02. The color scale represents theta power (µV²/Hz).

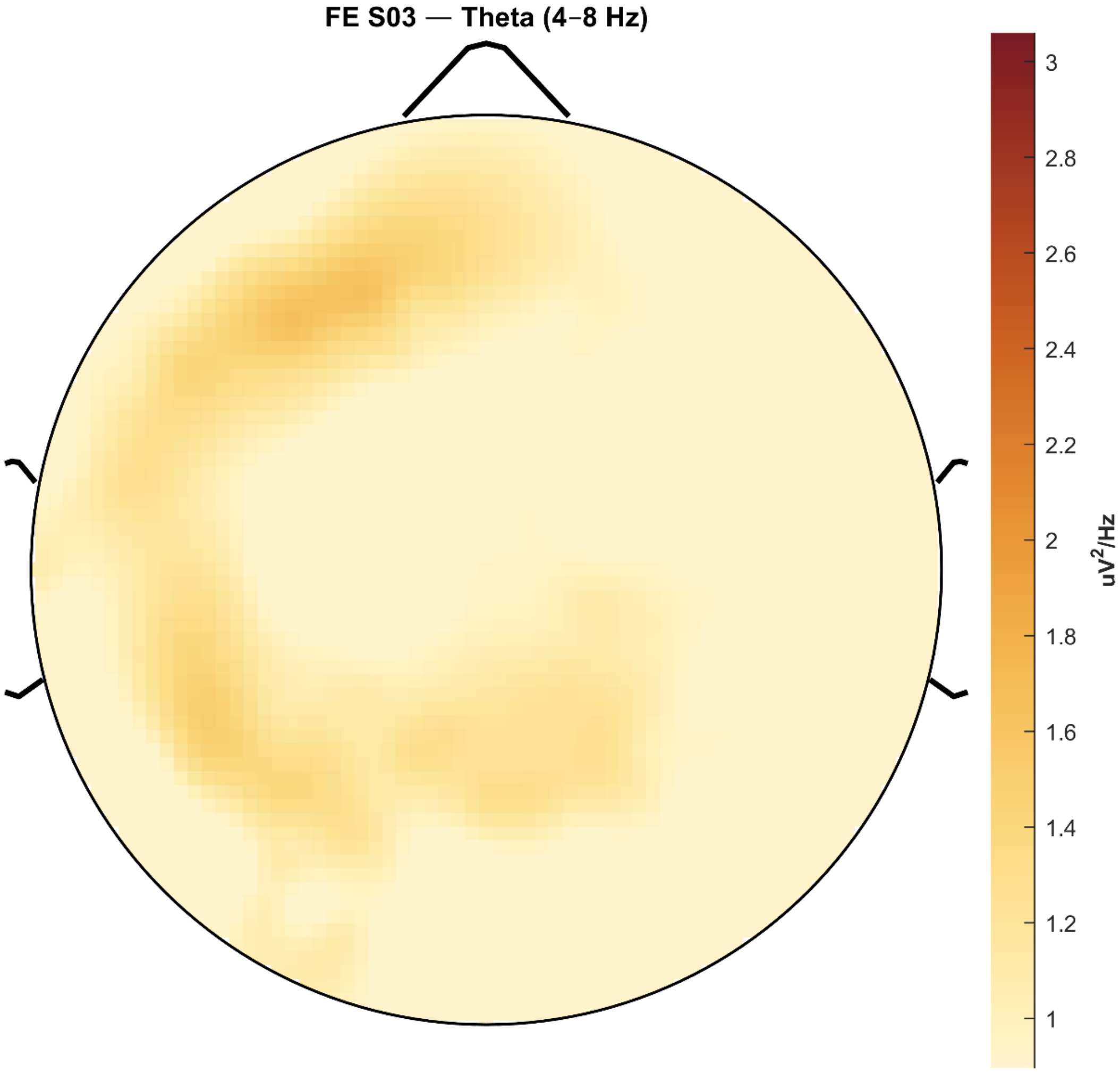

Supplementary Figure 43

Theta (4–8 Hz) power map for FE subject S03. The color scale represents theta power (µV²/Hz).

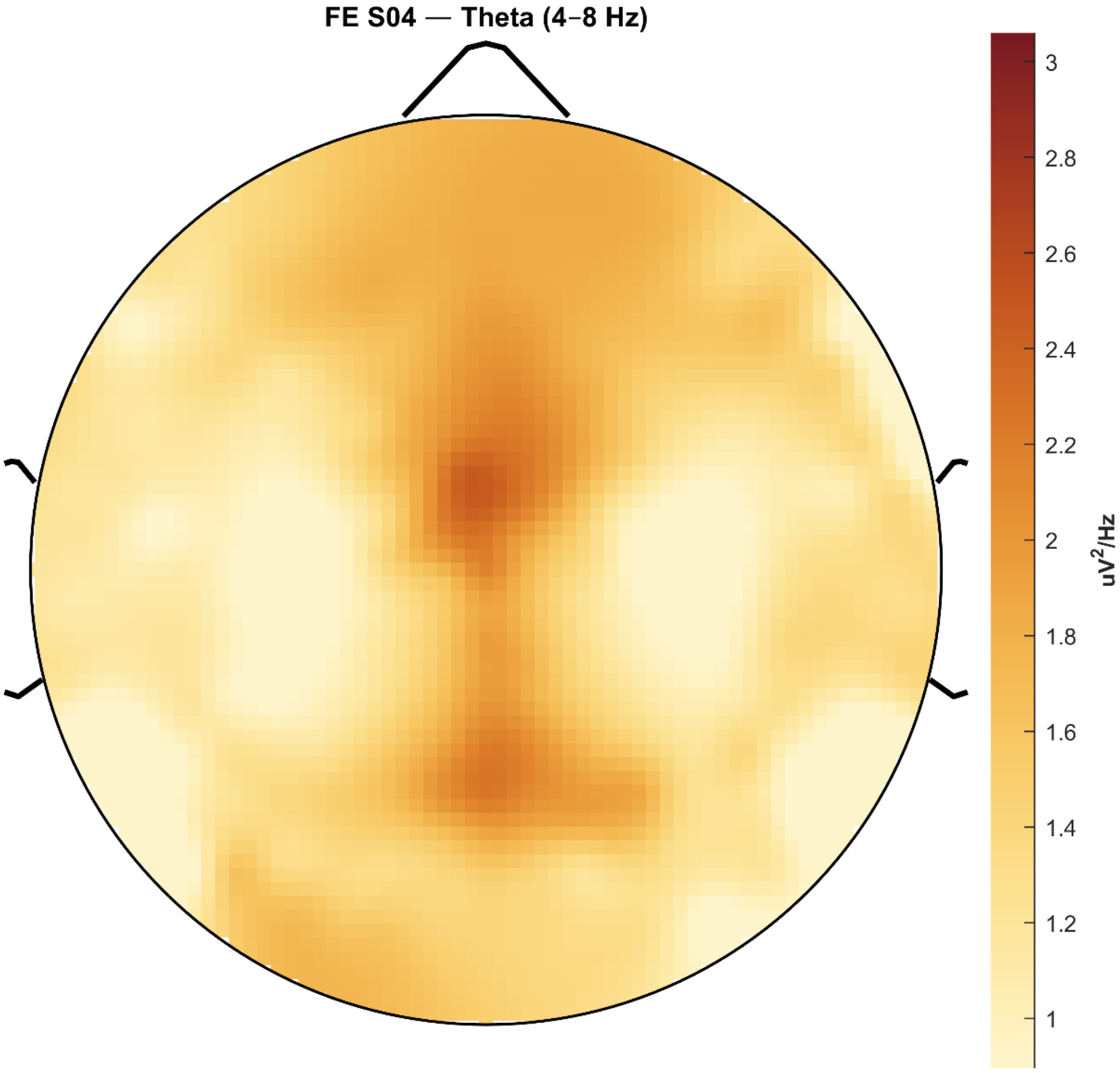

Supplementary Figure 44

Theta (4–8 Hz) power map for FE subject S04. The color scale represents theta power (μV²/Hz).

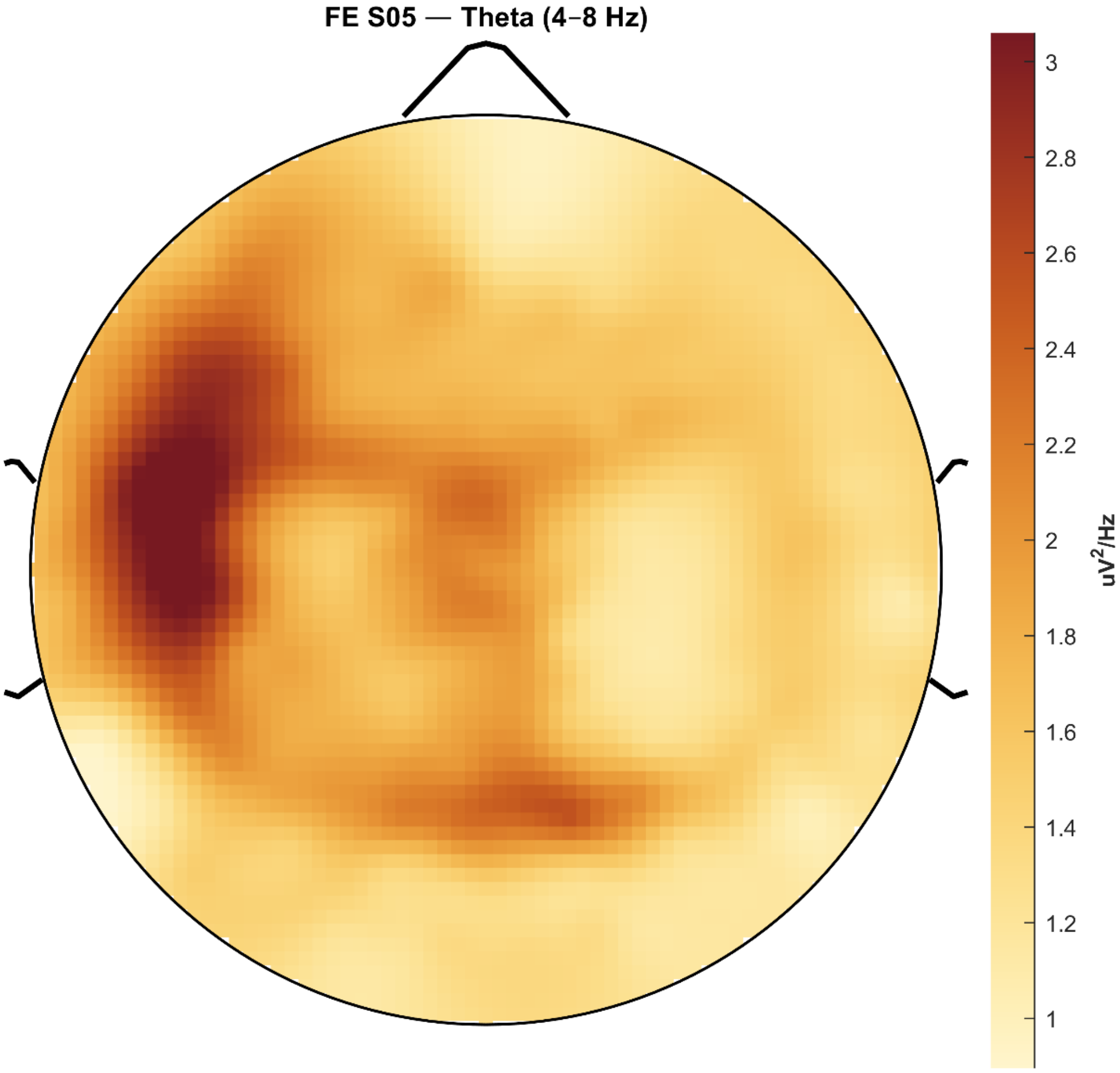

Supplementary Figure 45

Theta (4–8 Hz) power map for FE subject S05. The color scale represents theta power (µV²/Hz).

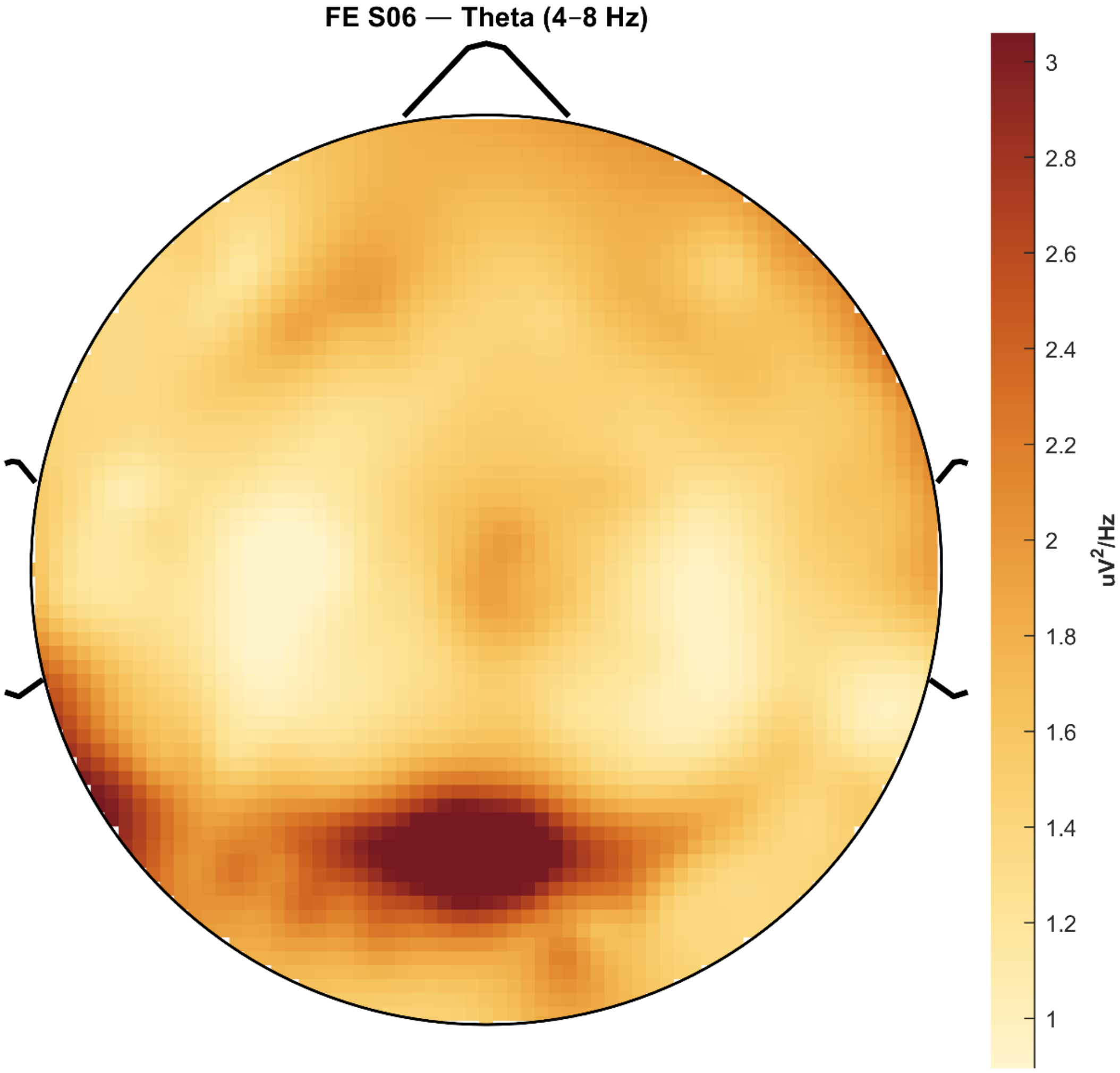

Supplementary Figure 46

Theta (4–8 Hz) power map for FE subject S06. The color scale represents theta power (µV²/Hz).

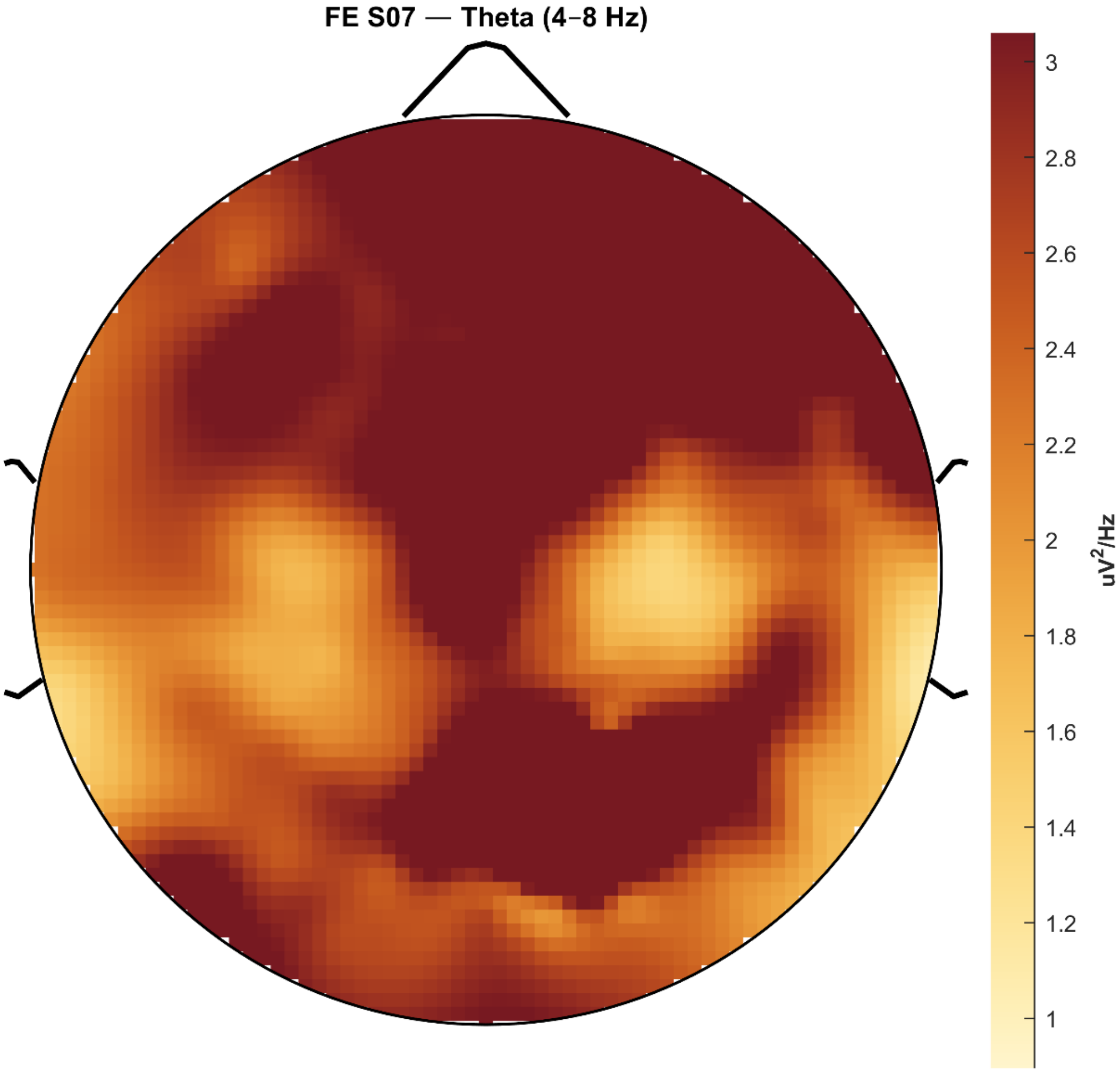

Supplementary Figure 47

Theta (4–8 Hz) power map for FE subject S07. The color scale represents theta power (μV²/Hz).

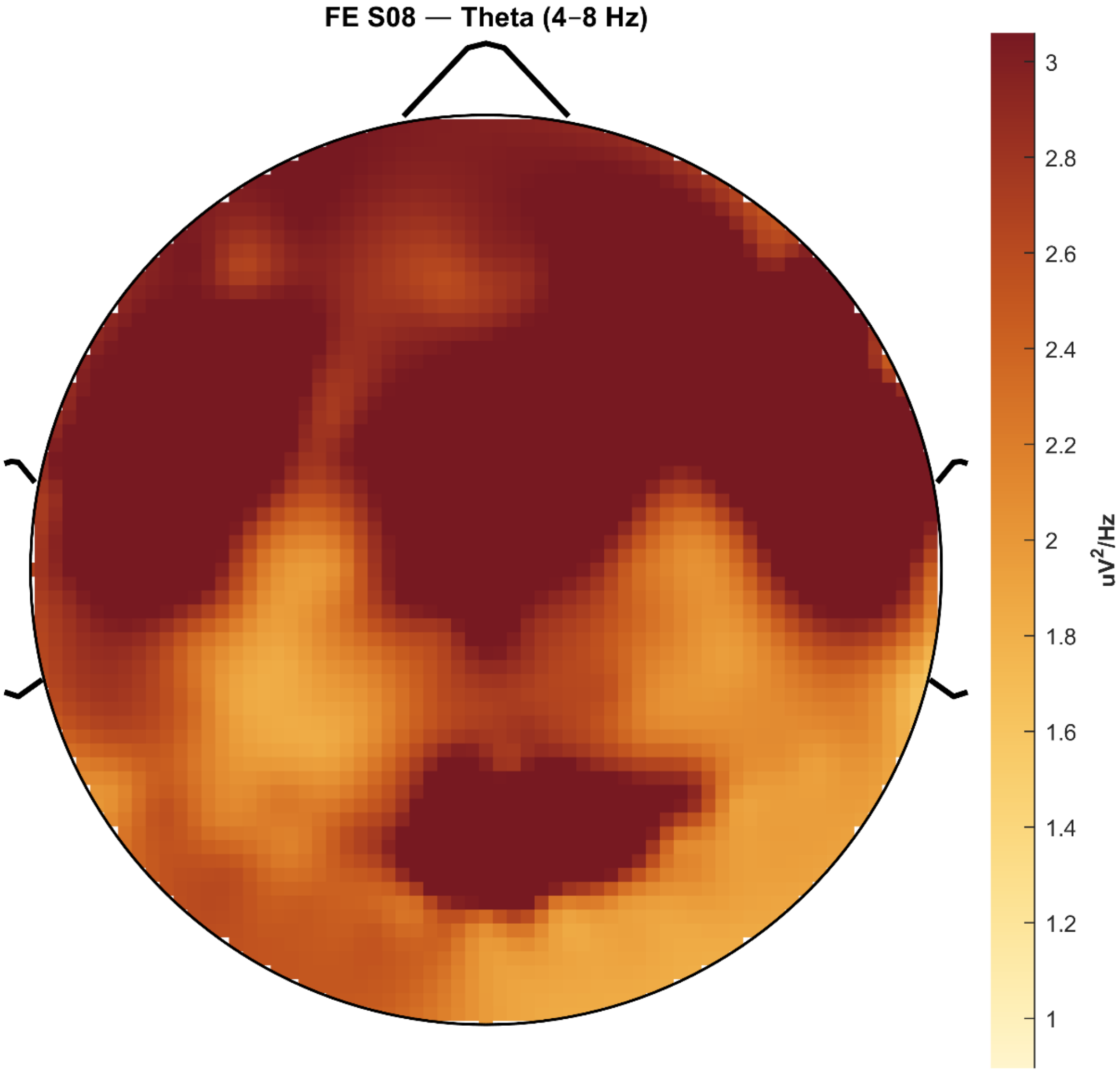

Supplementary Figure 48

Theta (4–8 Hz) power map for FE subject S08. The color scale represents theta power (μV²/Hz).

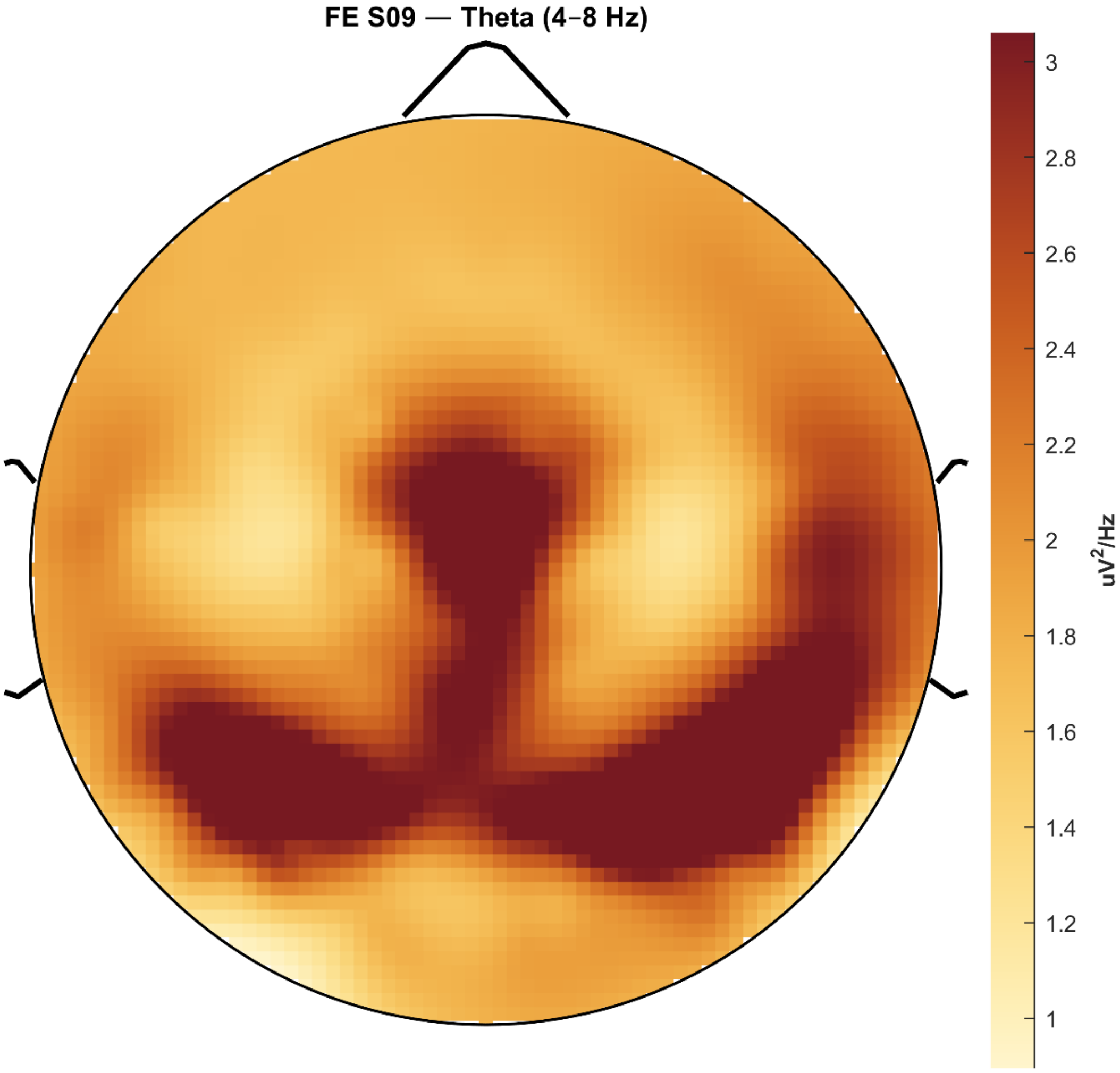

Supplementary Figure 49

Theta (4–8 Hz) power map for FE subject S09. The color scale represents theta power (µV²/Hz).

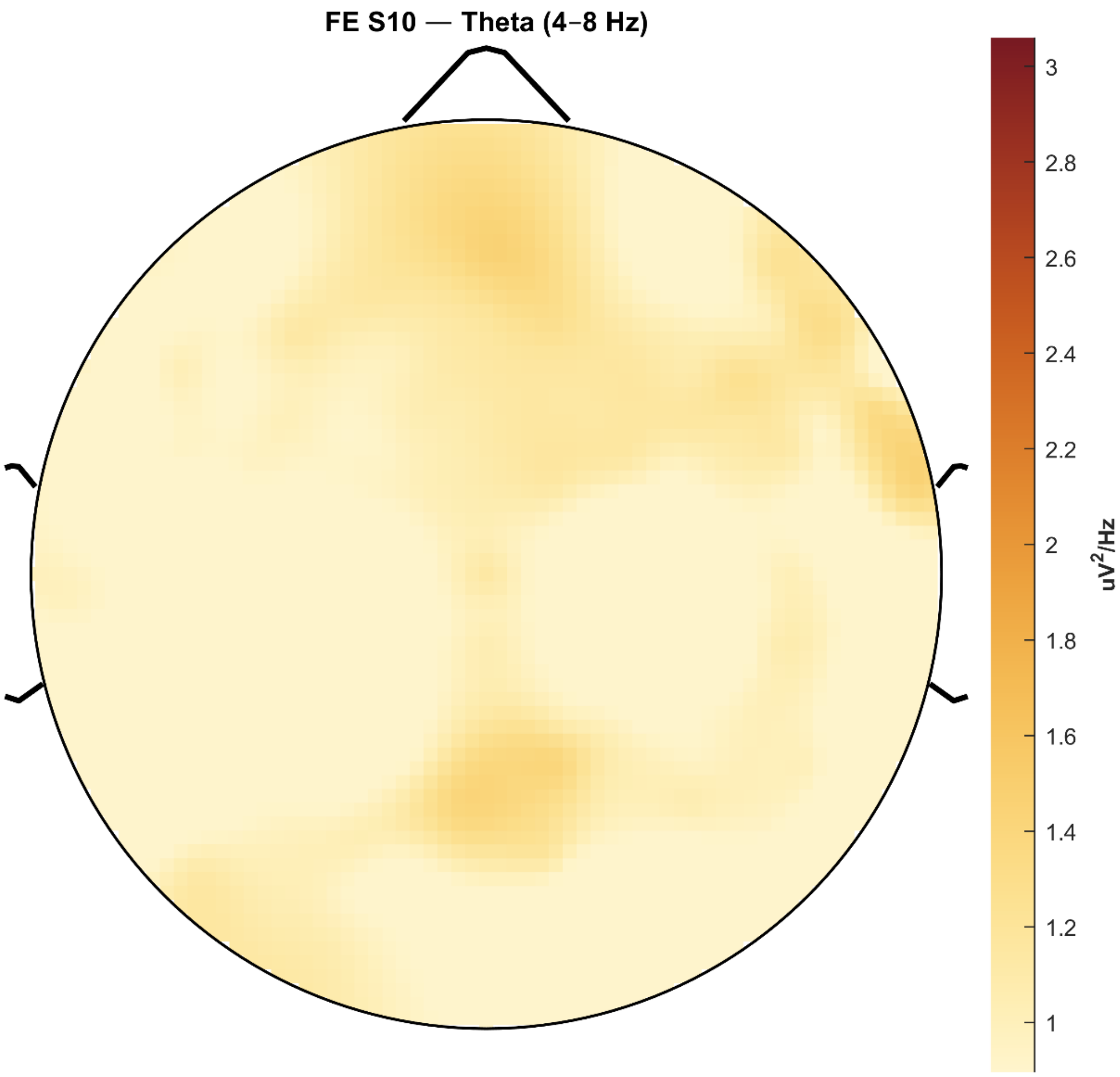

Supplementary Figure 50

Theta (4–8 Hz) power map for FE subject S10. The color scale represents theta power (μV²/Hz).

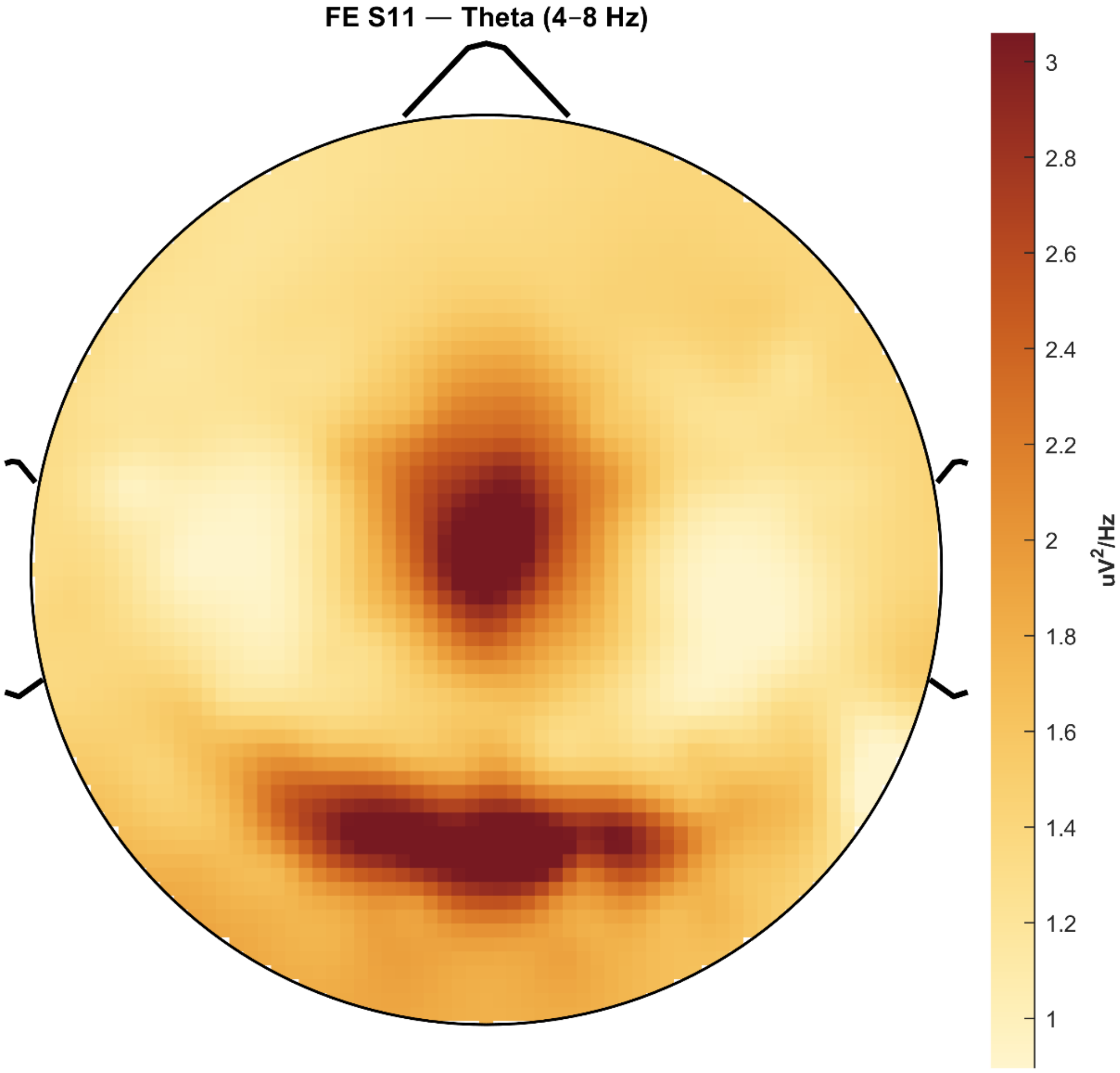

Supplementary Figure 51

Theta (4–8 Hz) power map for FE subject S11. The color scale represents theta power (µV²/Hz).

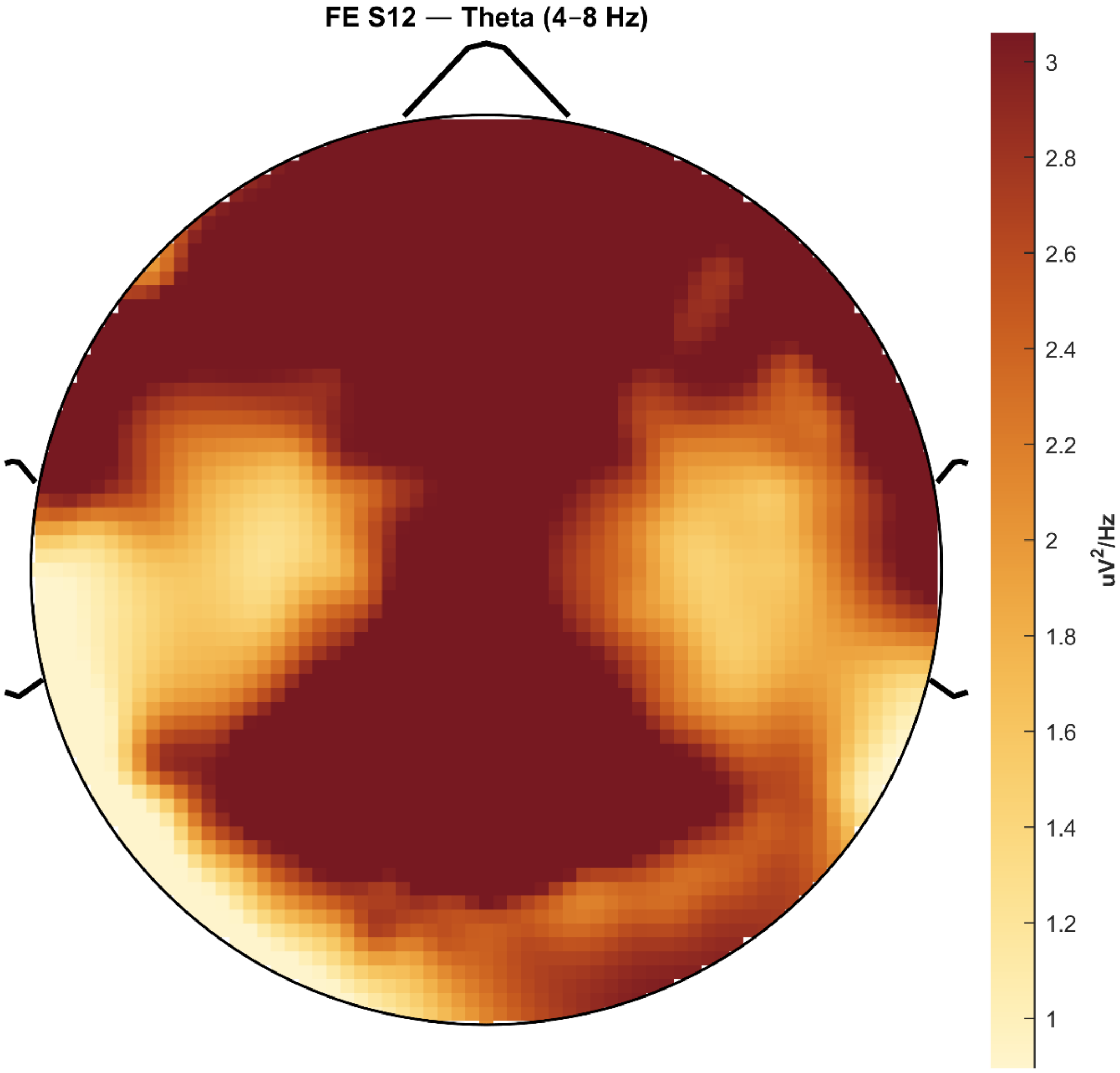

Supplementary Figure 52

Theta (4–8 Hz) power map for FE subject S12. The color scale represents theta power (µV²/Hz).

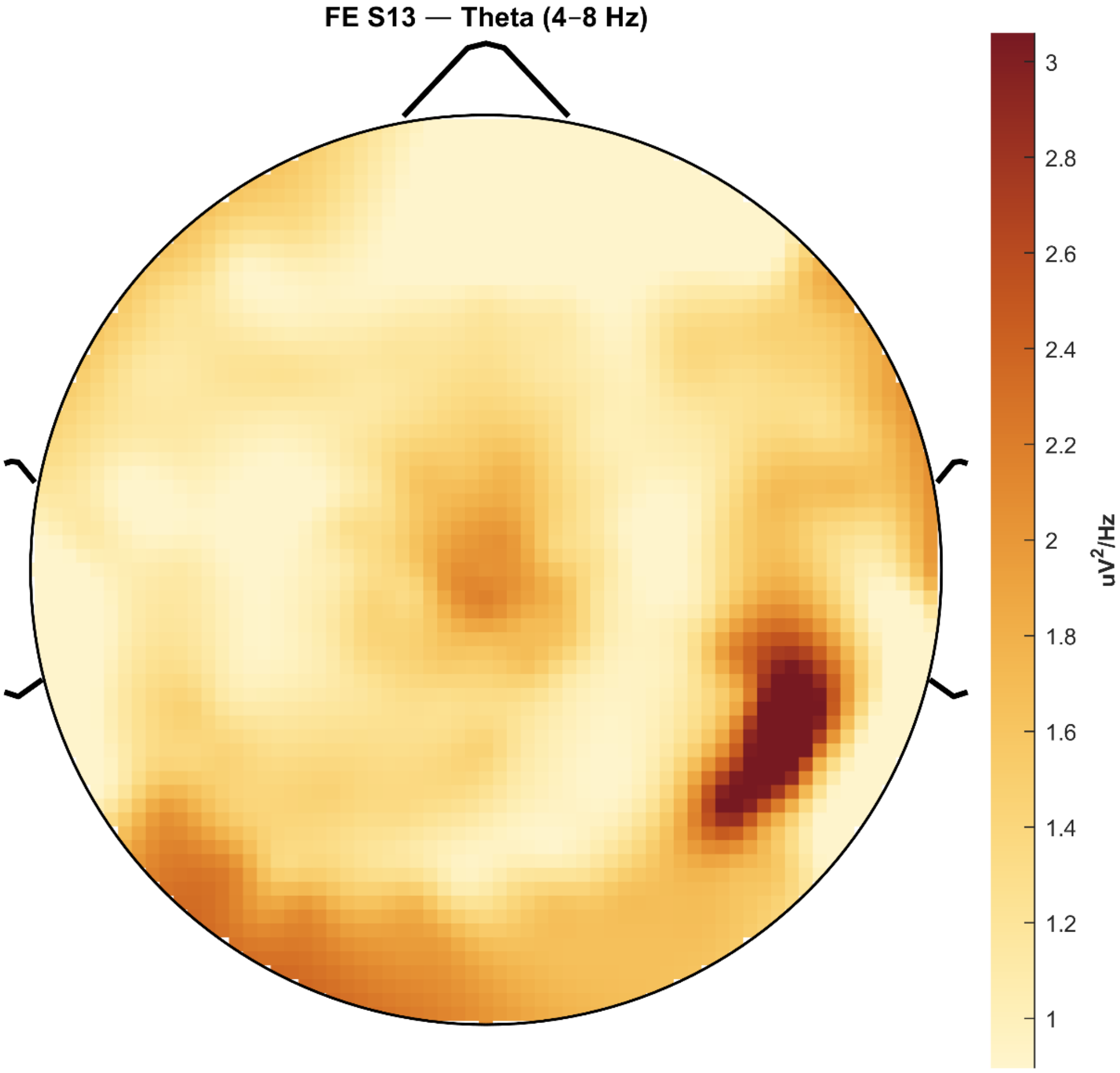

Supplementary Figure 53

Theta (4–8 Hz) power map for FE subject S13. The color scale represents theta power (µV²/Hz).

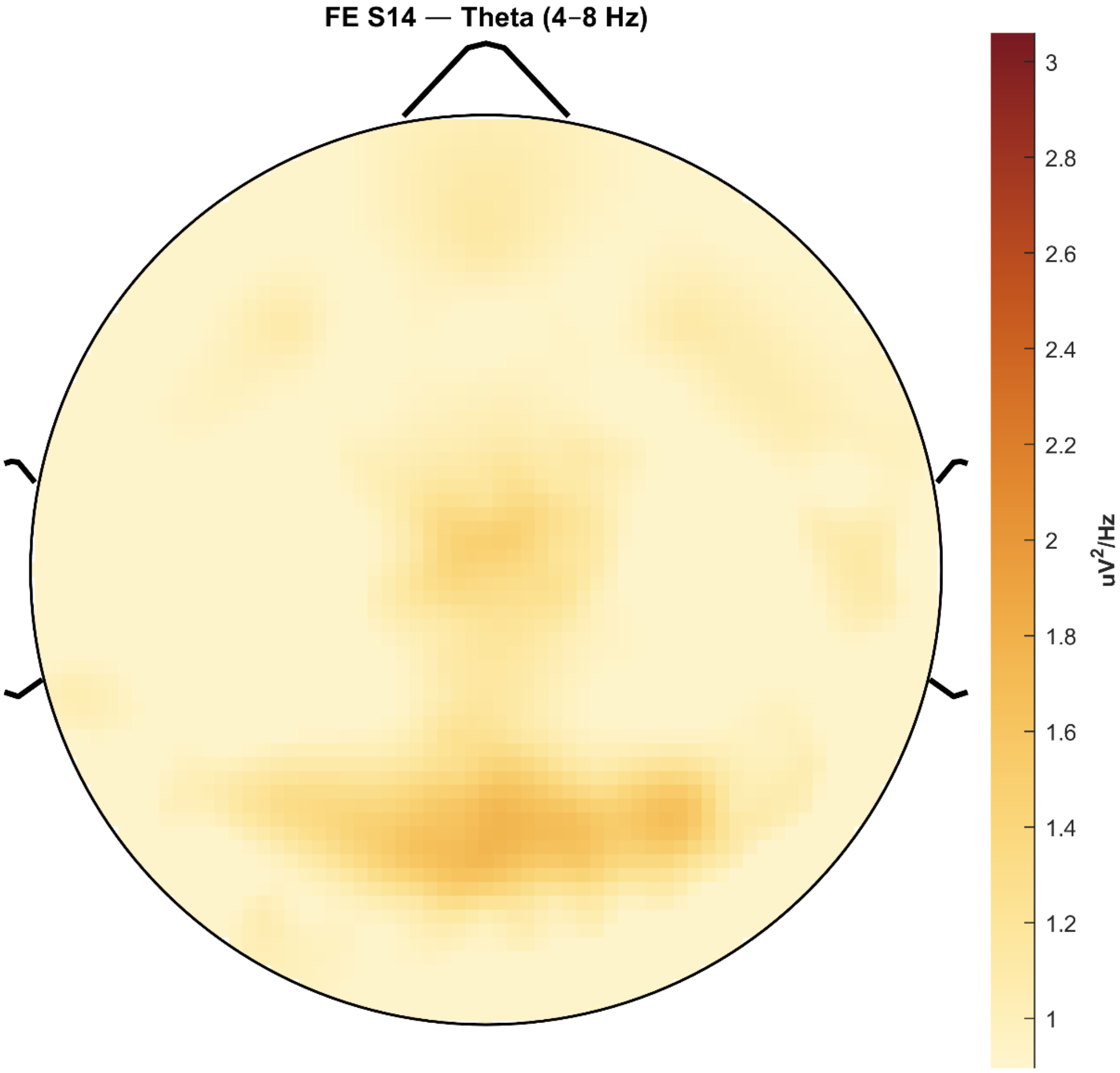

Supplementary Figure 54

Theta (4–8 Hz) power map for FE subject S14. The color scale represents theta power (μV²/Hz).

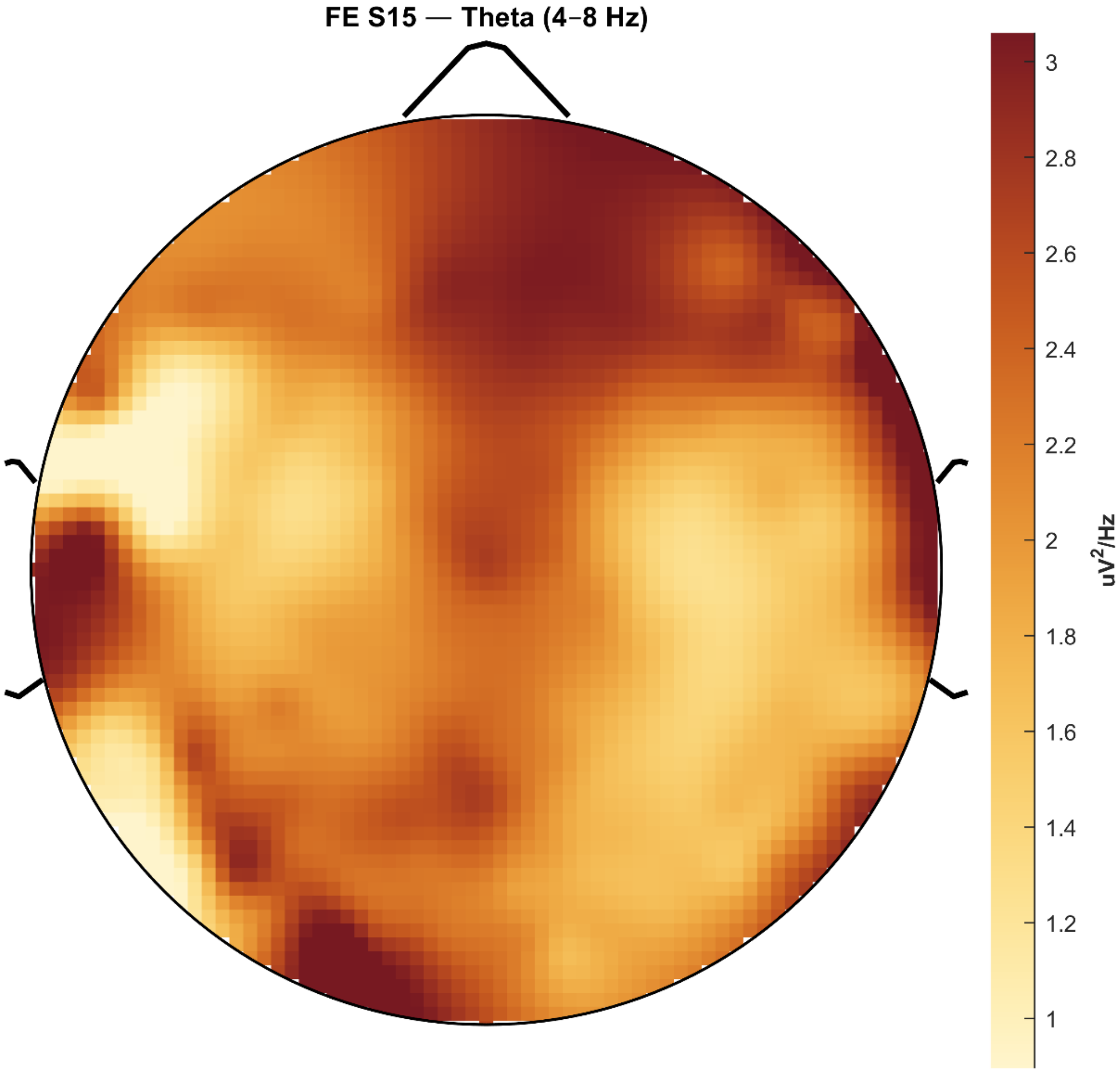

Supplementary Figure 55

Theta (4–8 Hz) power map for FE subject S15. The color scale represents theta power (µV²/Hz).

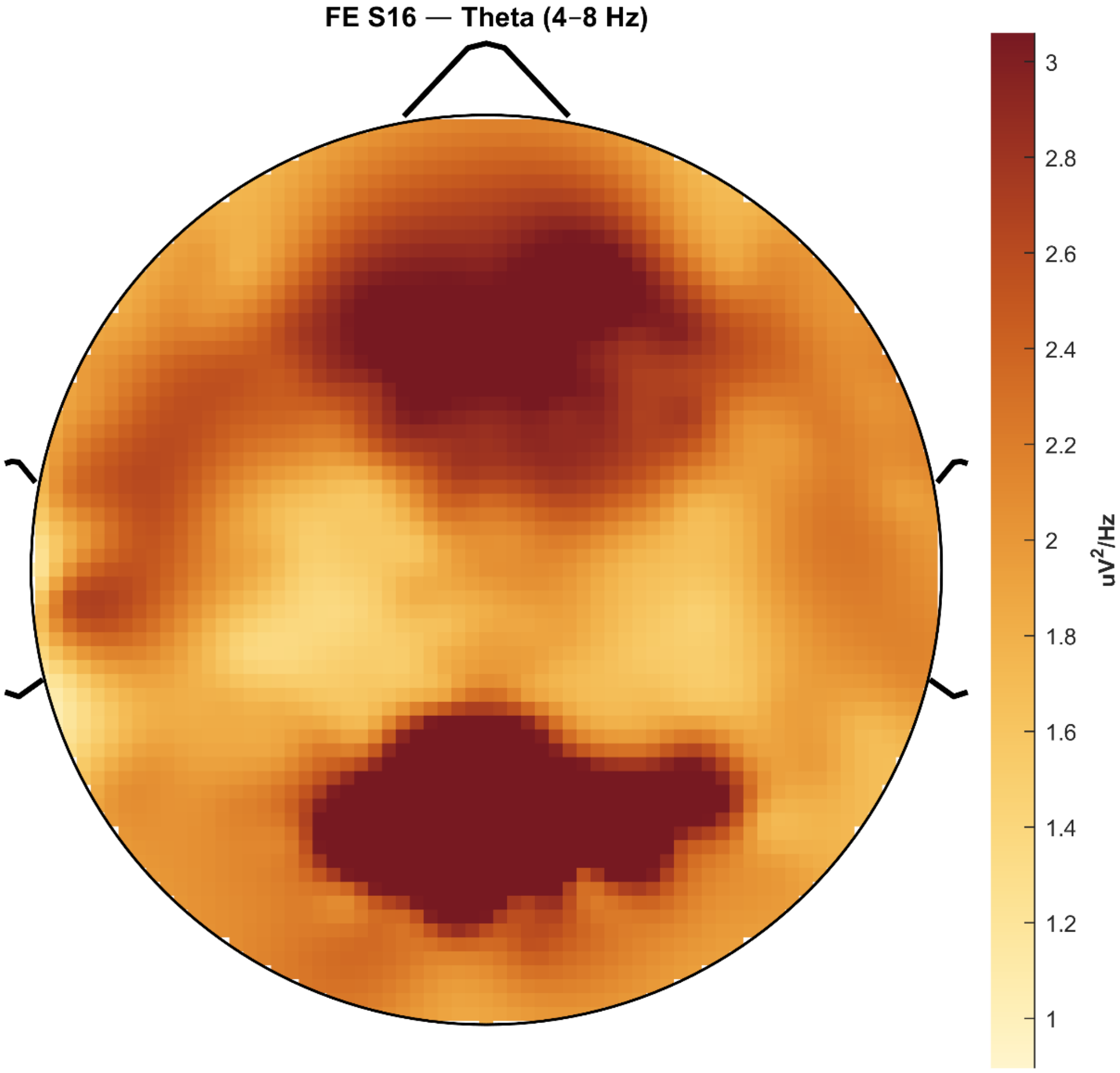

Supplementary Figure 56

Theta (4–8 Hz) power map for FE subject S16. The color scale represents theta power (µV²/Hz).

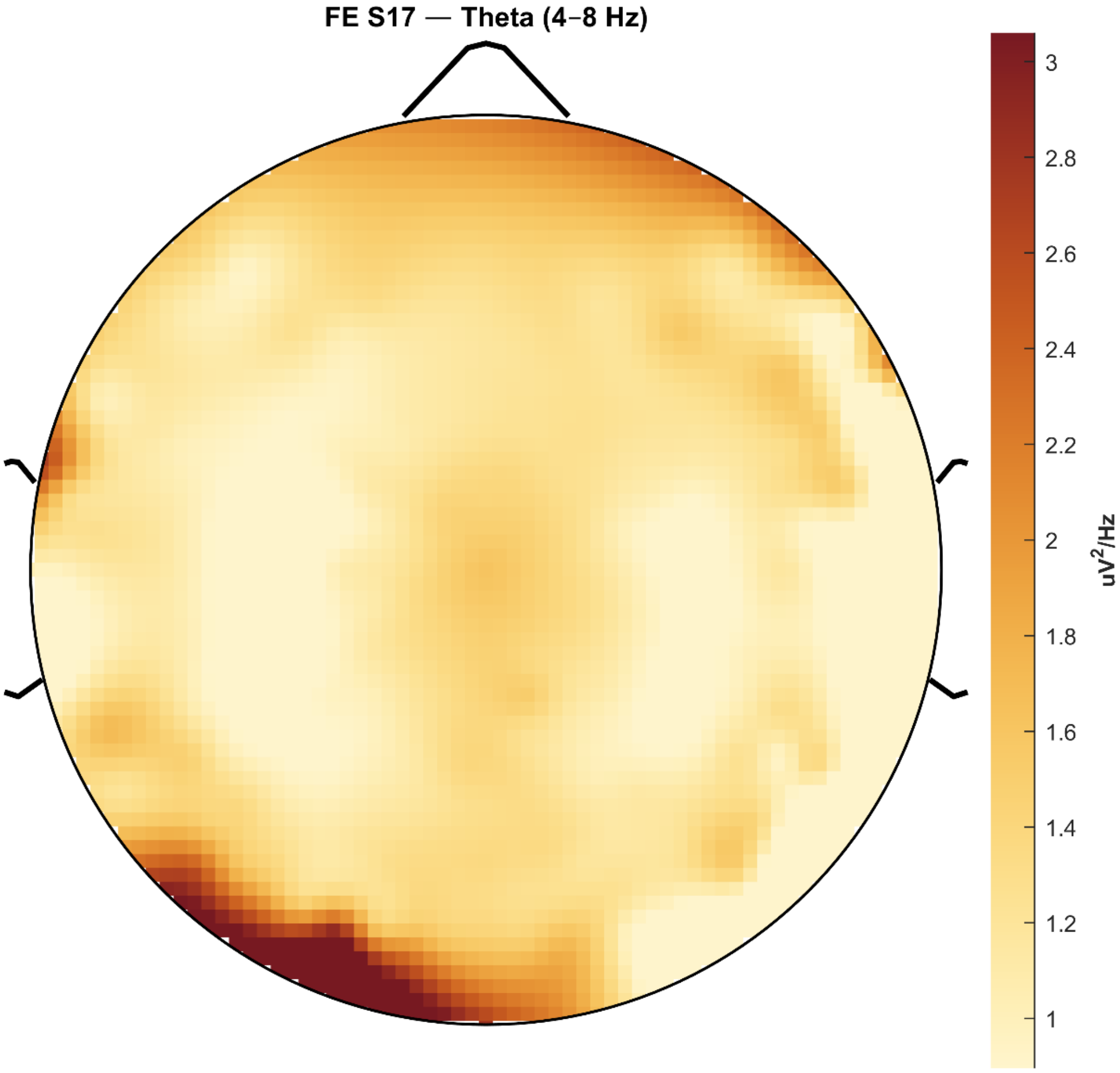

Supplementary Figure 57

Theta (4–8 Hz) power map for FE subject S17. The color scale represents theta power (μV²/Hz).

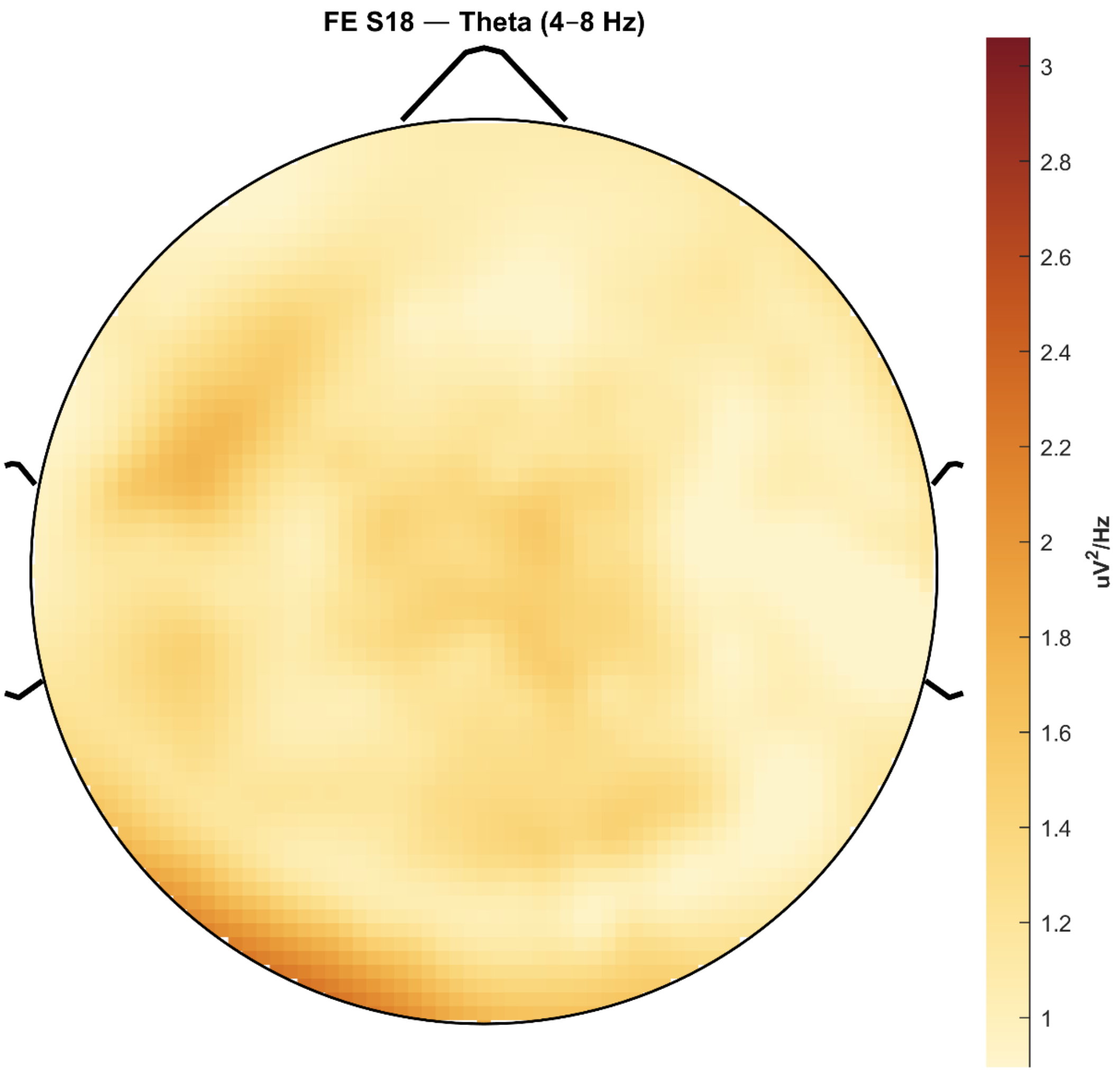

Supplementary Figure 58

Theta (4–8 Hz) power map for FE subject S18. The color scale represents theta power (μV²/Hz).

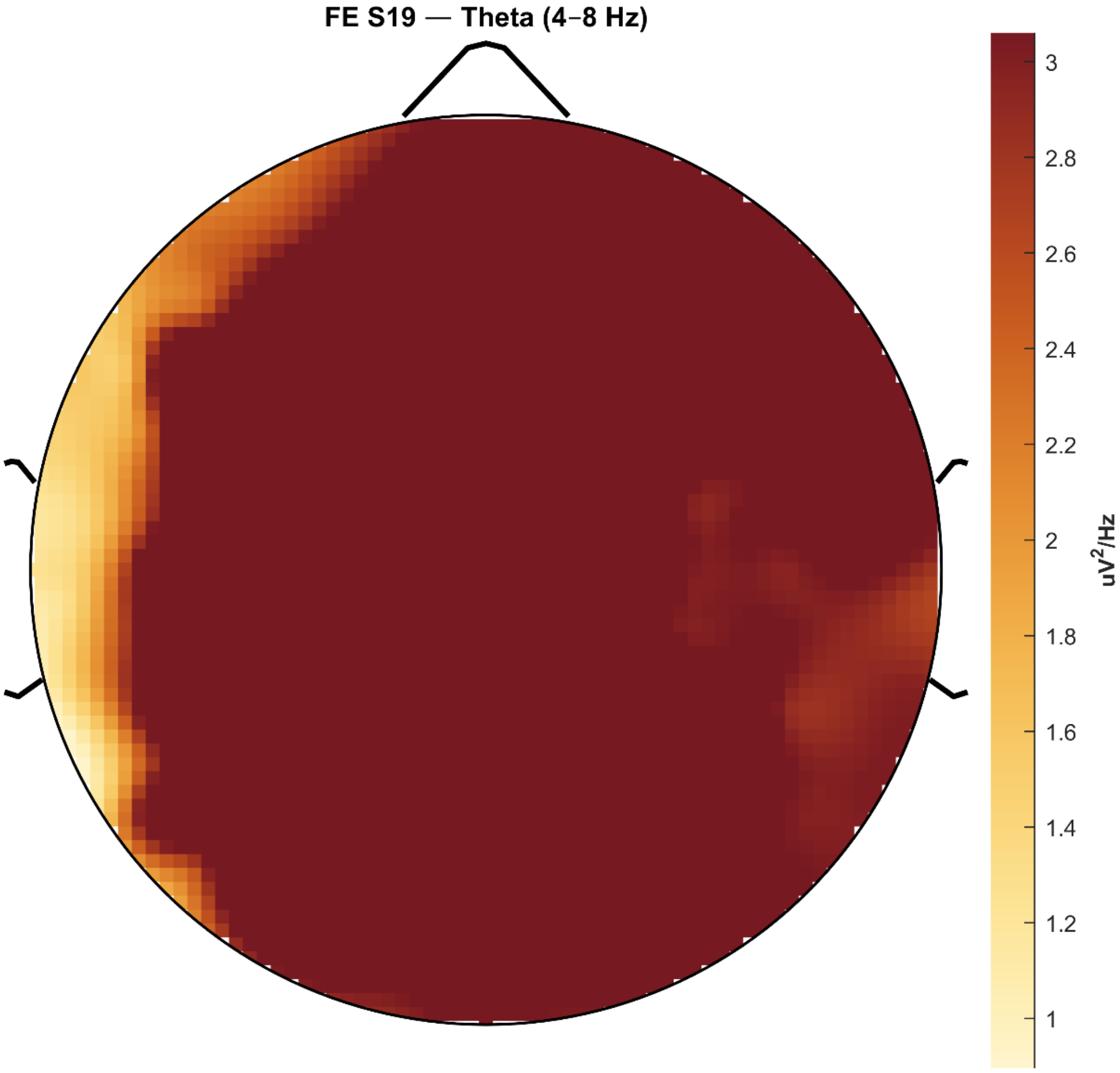

Supplementary Figure 59

Theta (4–8 Hz) power map for FE subject S19. The color scale represents theta power (µV²/Hz).

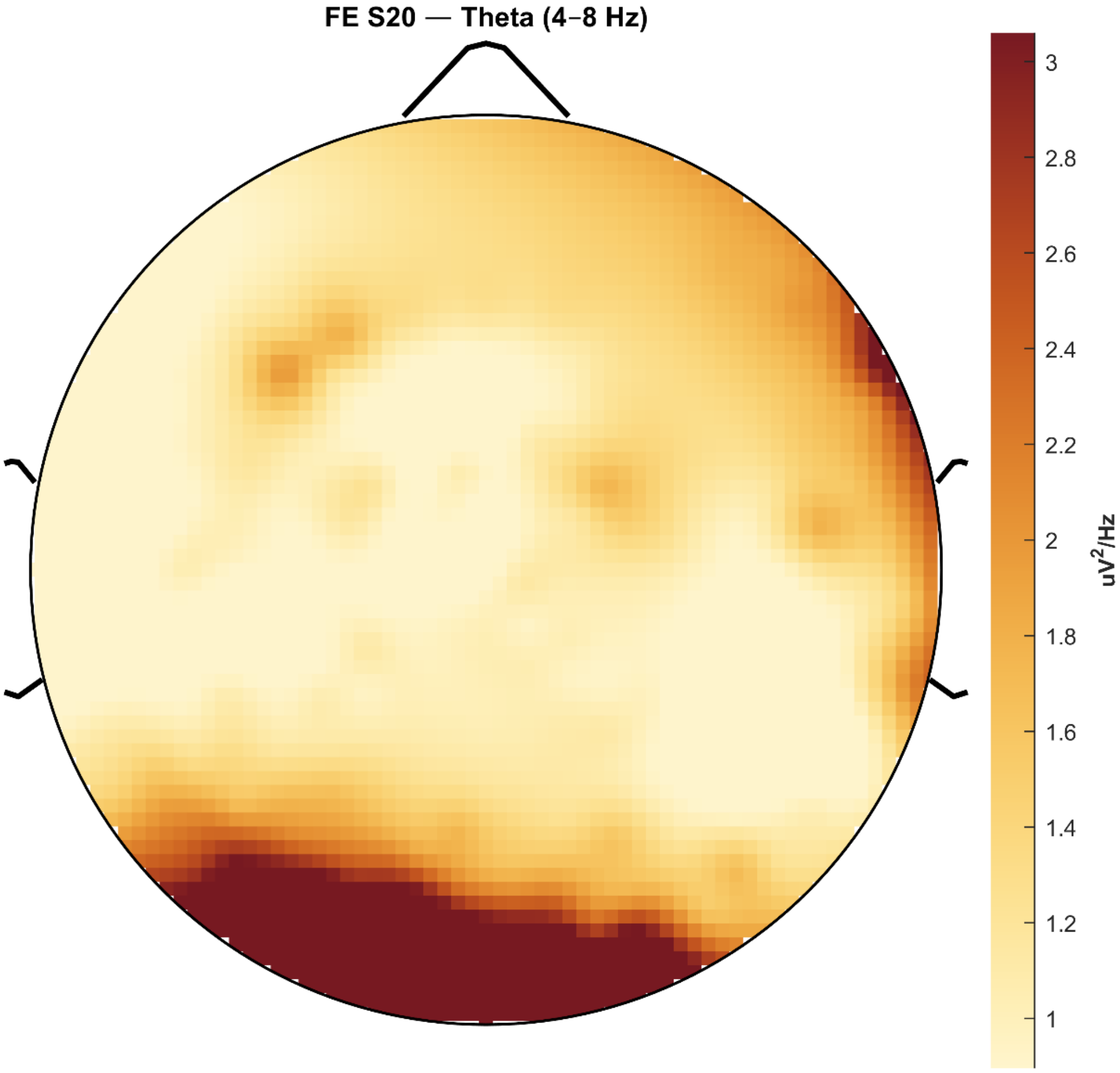

Supplementary Figure 60

Theta (4–8 Hz) power map for FE subject S20. The color scale represents theta power (µV²/Hz).

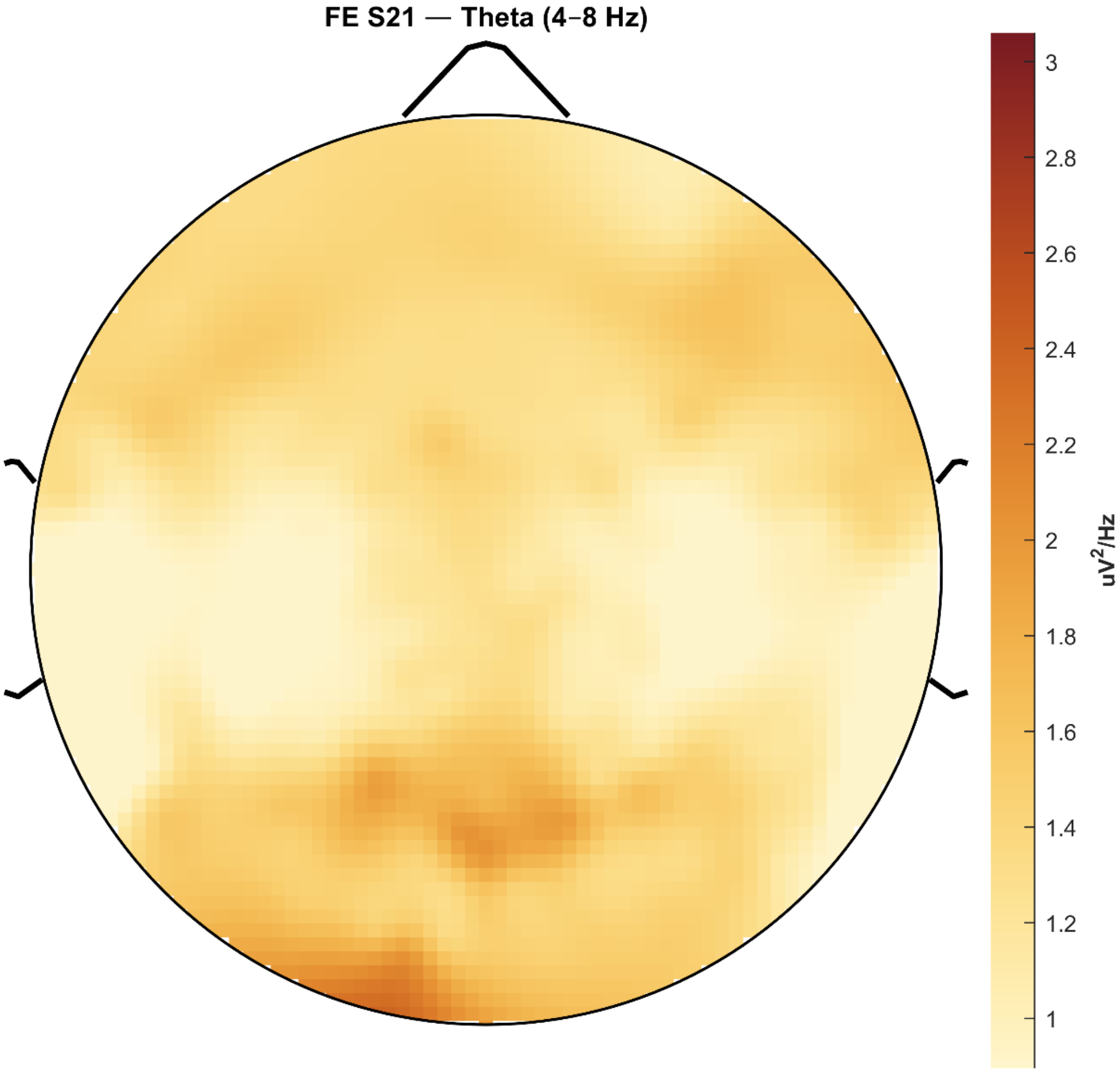

Supplementary Figure 61

Theta (4–8 Hz) power map for FE subject S21. The color scale represents theta power (µV²/Hz).

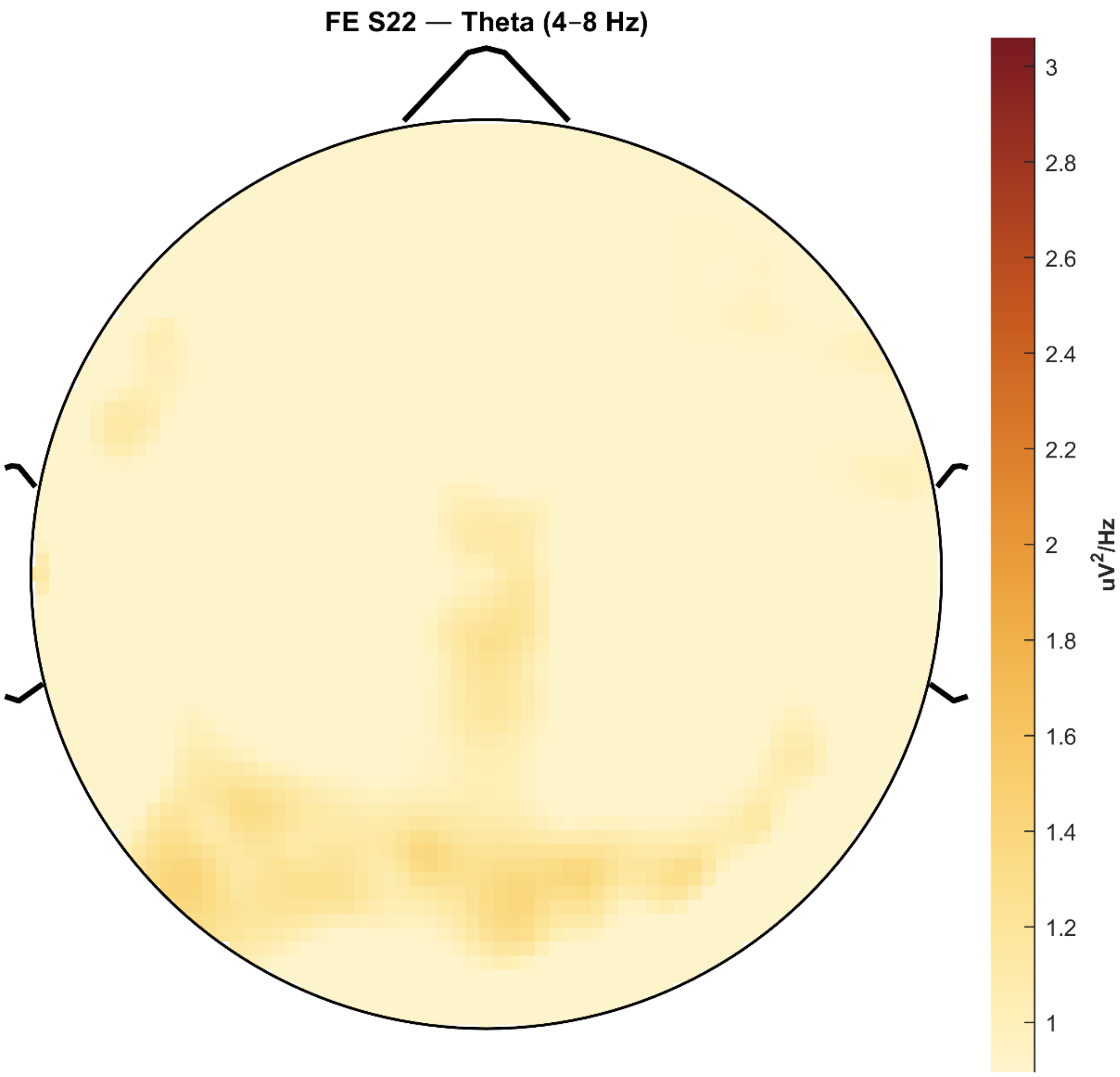

Supplementary Figure 62

Theta (4–8 Hz) power map for FE subject S22. The color scale represents theta power (μV²/Hz).

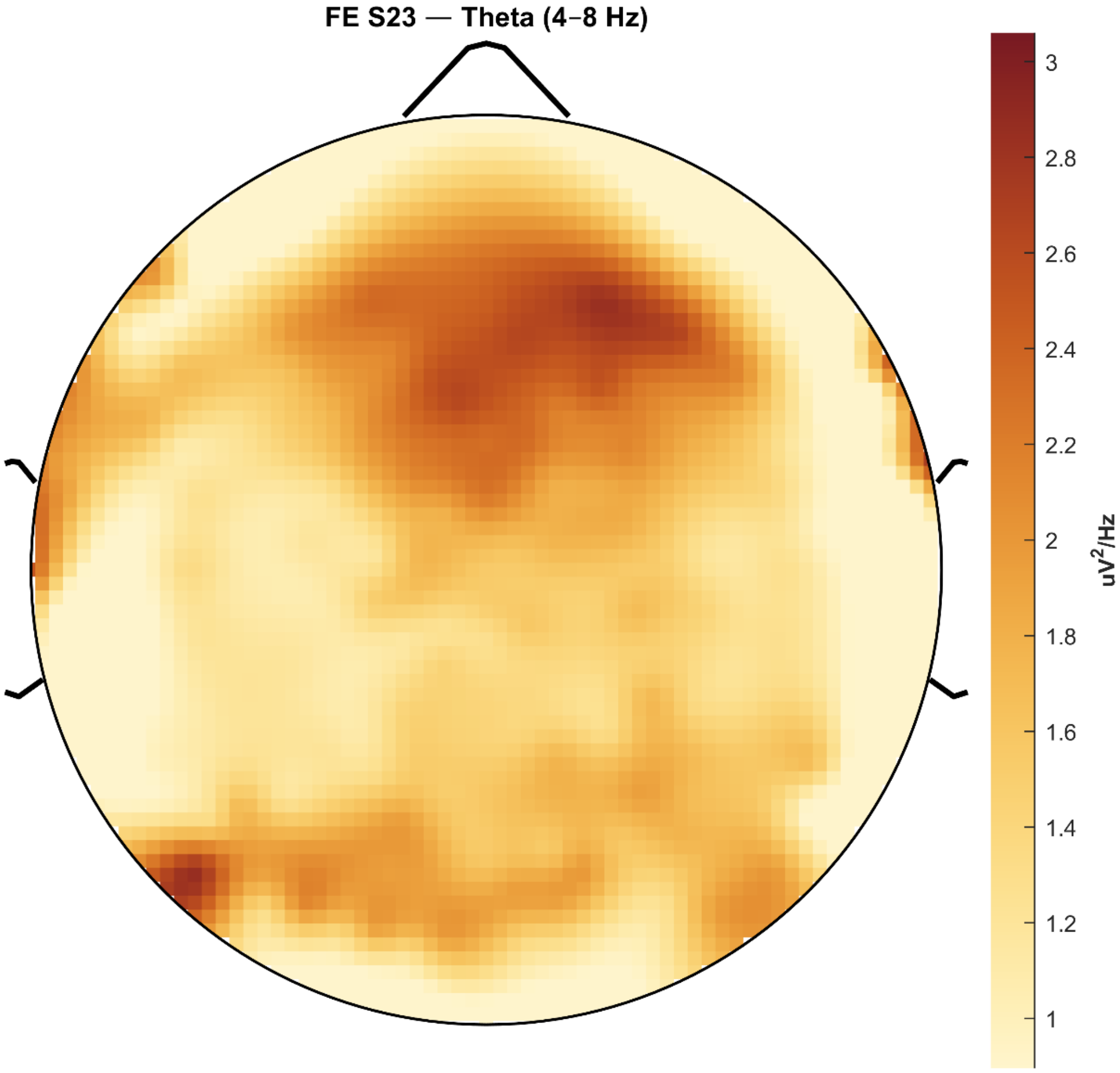

Supplementary Figure 63

Theta (4–8 Hz) power map for FE subject S23. The color scale represents theta power (µV²/Hz).

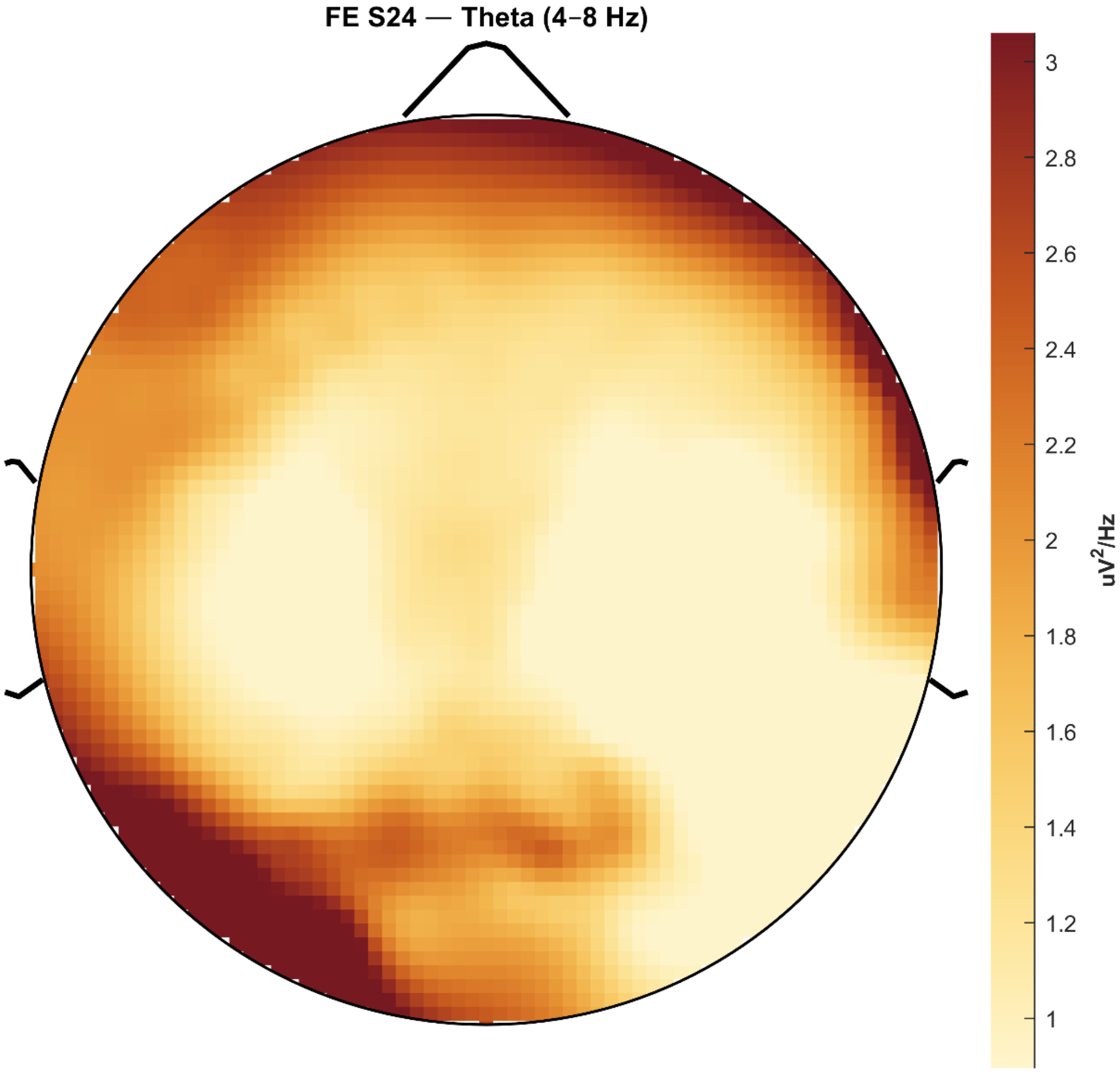

Supplementary Figure 64

Theta (4–8 Hz) power map for FE subject S24. The color scale represents theta power (μV²/Hz).

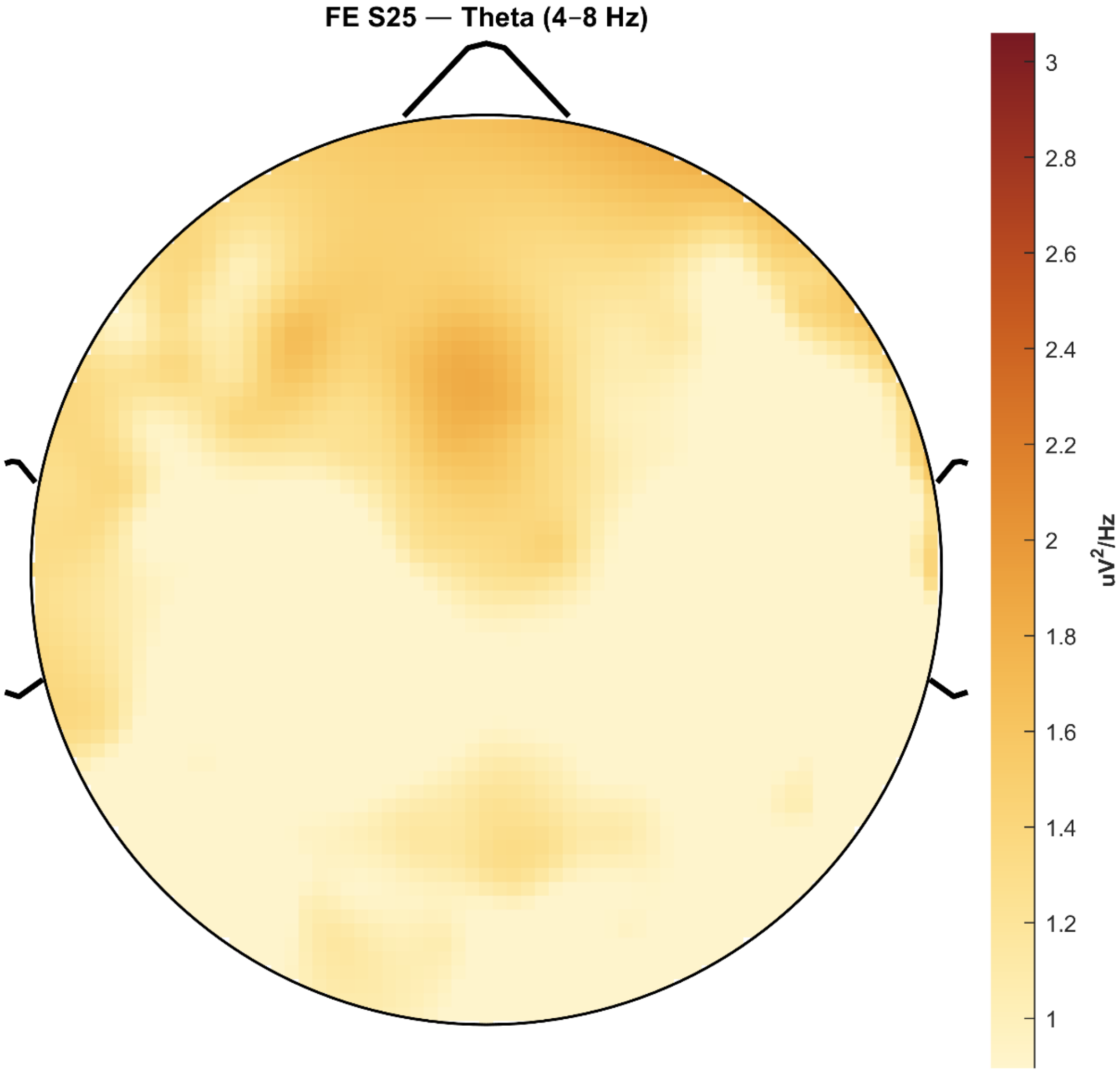

Supplementary Figure 65

Theta (4–8 Hz) power map for FE subject S25. The color scale represents theta power (µV²/Hz).

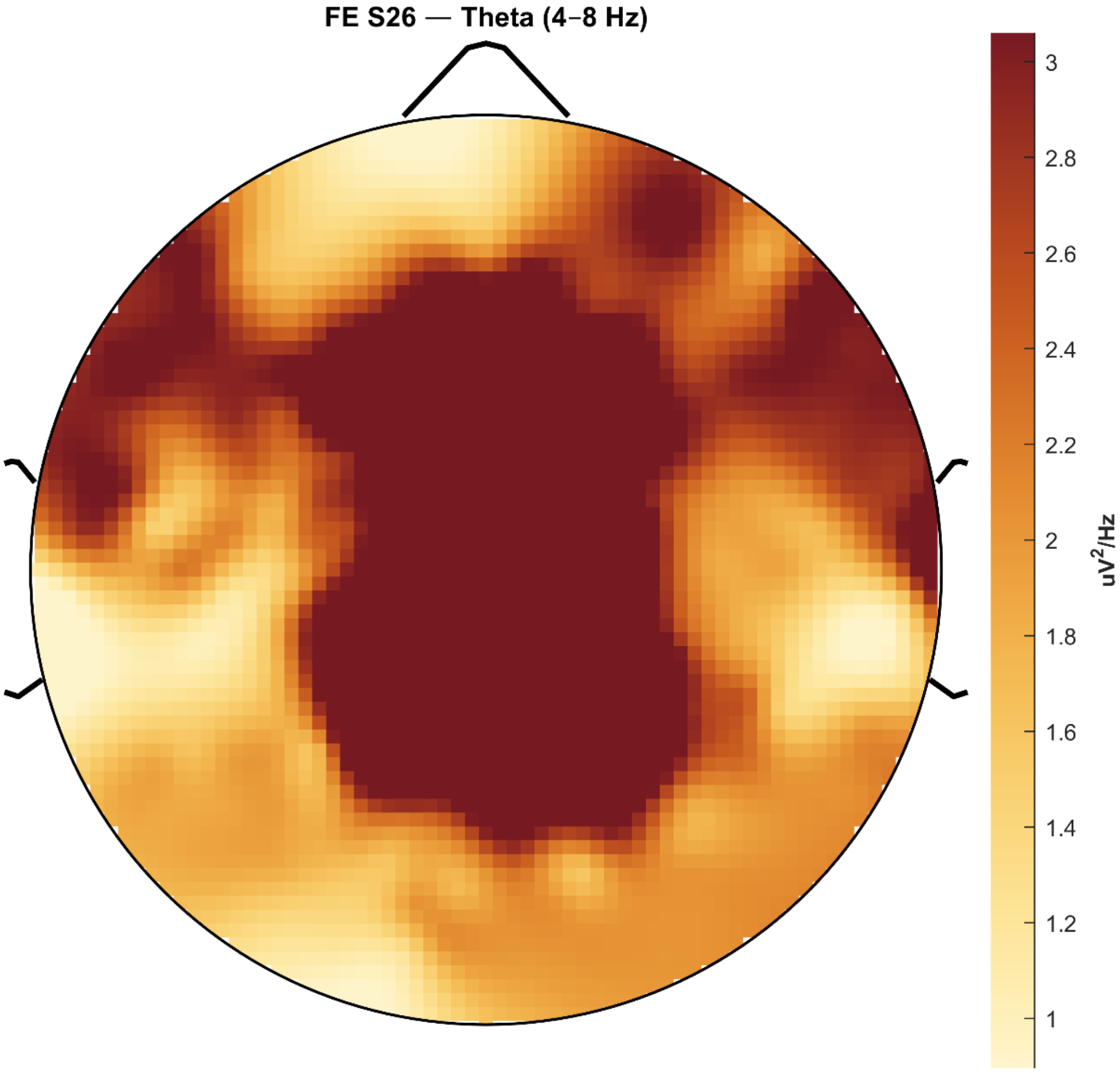

Supplementary Figure 66

Theta (4–8 Hz) power map for FE subject S26. The color scale represents theta power (μV²/Hz).

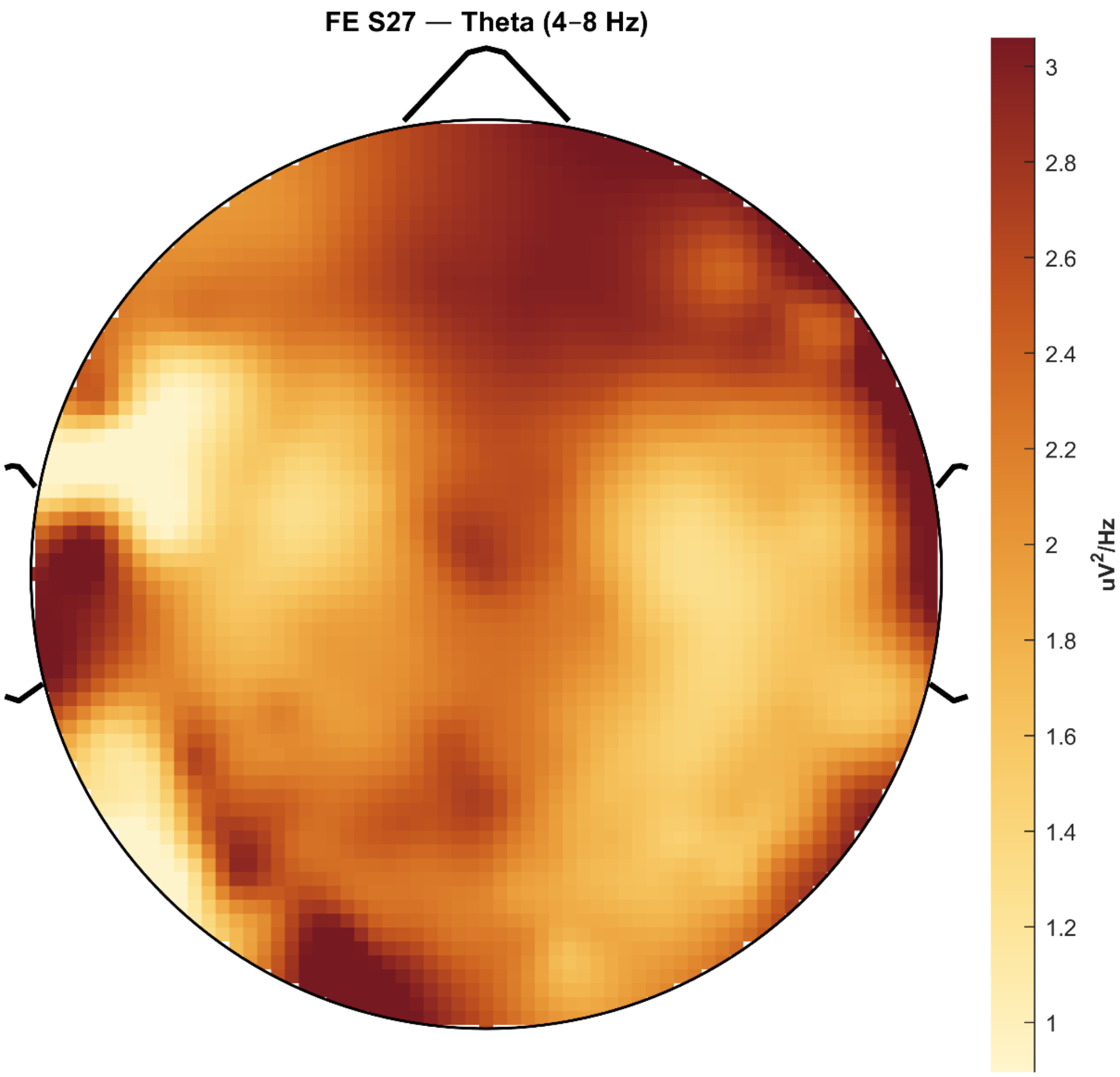

Supplementary Figure 67

Theta (4–8 Hz) power map for FE subject S27. The color scale represents theta power (µV²/Hz).

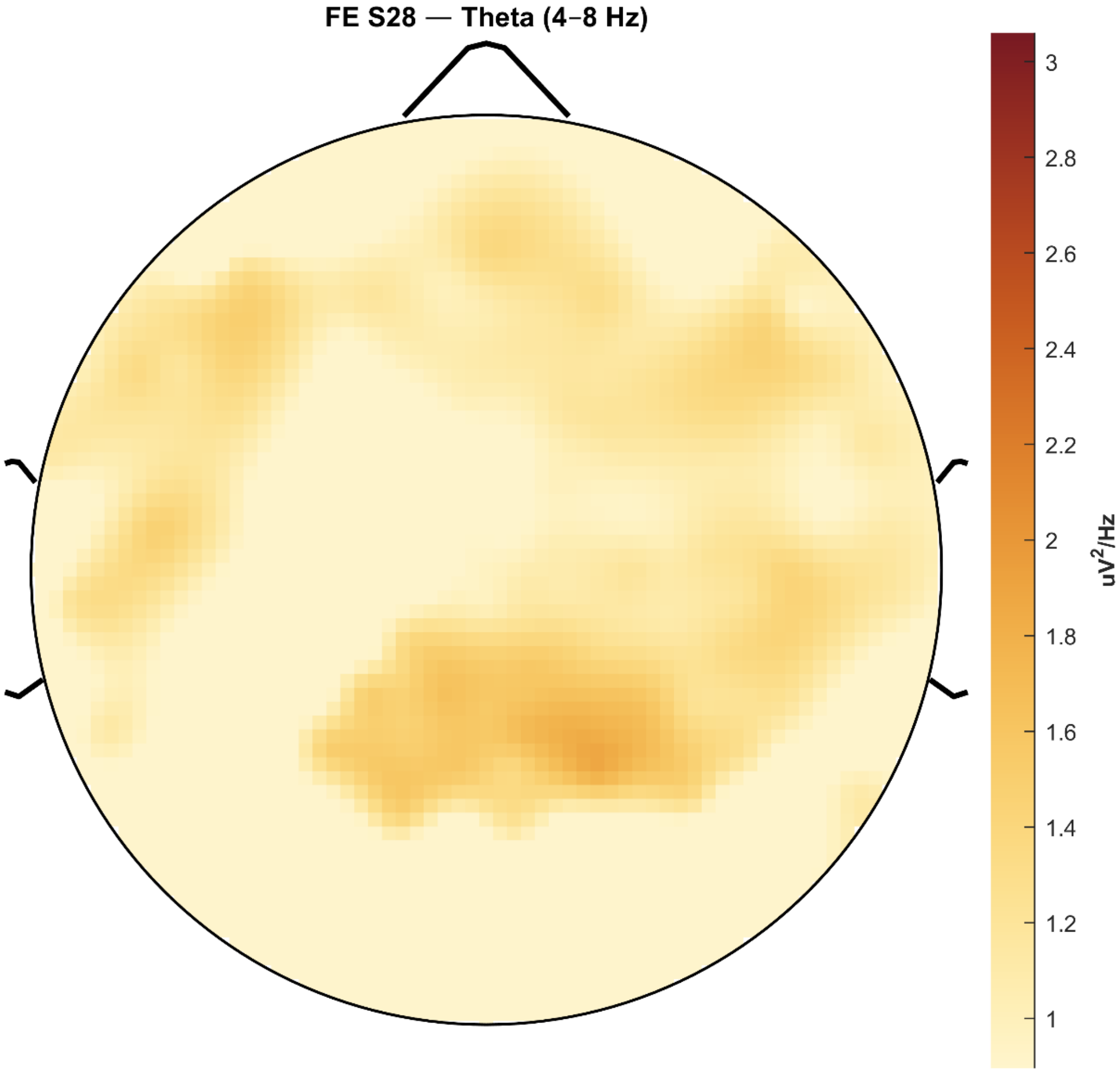

Supplementary Figure 68

Theta (4–8 Hz) power map for FE subject S28. The color scale represents theta power (µV²/Hz).

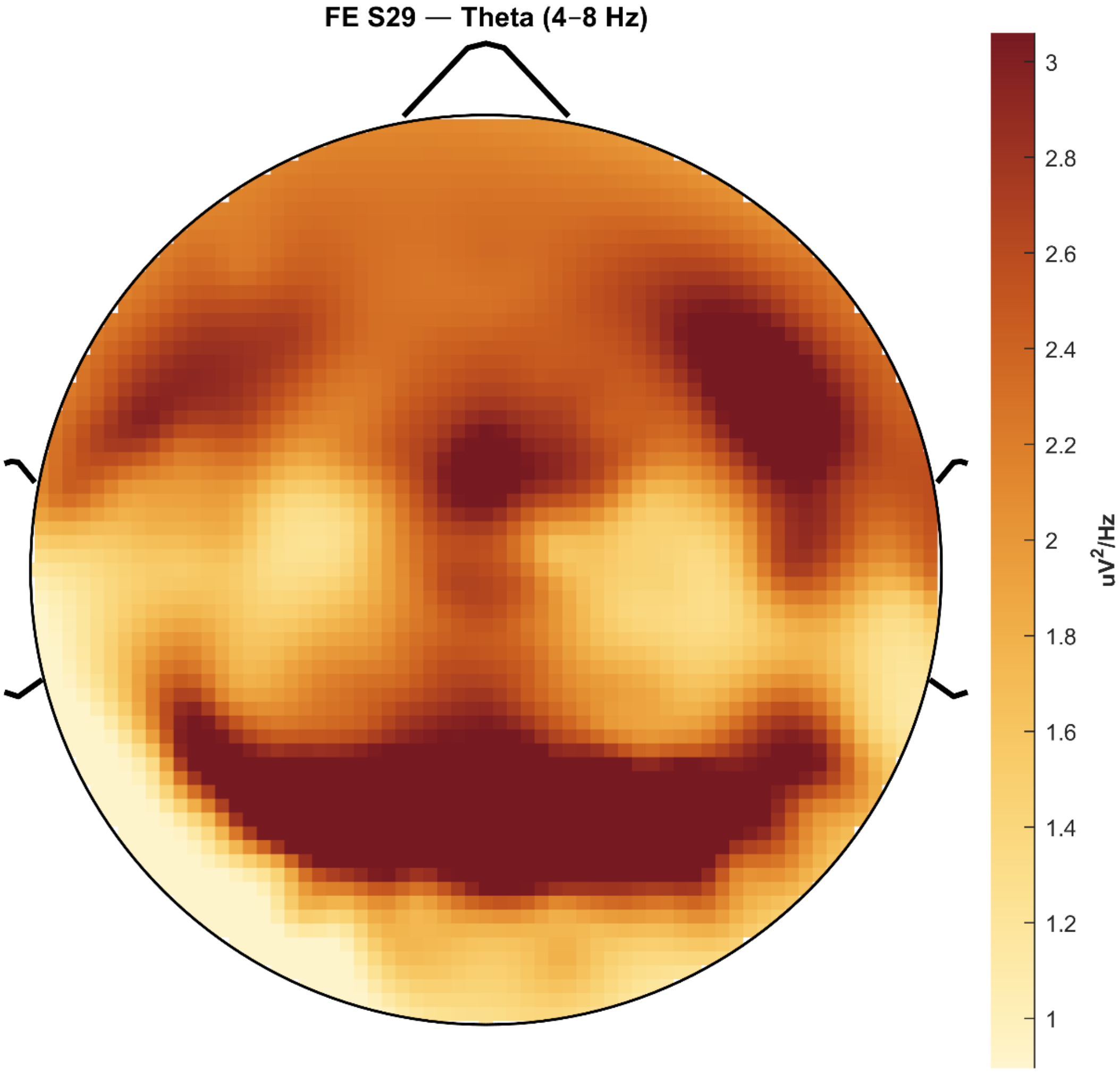

Supplementary Figure 69

Theta (4–8 Hz) power map for FE subject S29. The color scale represents theta power (μV²/Hz).

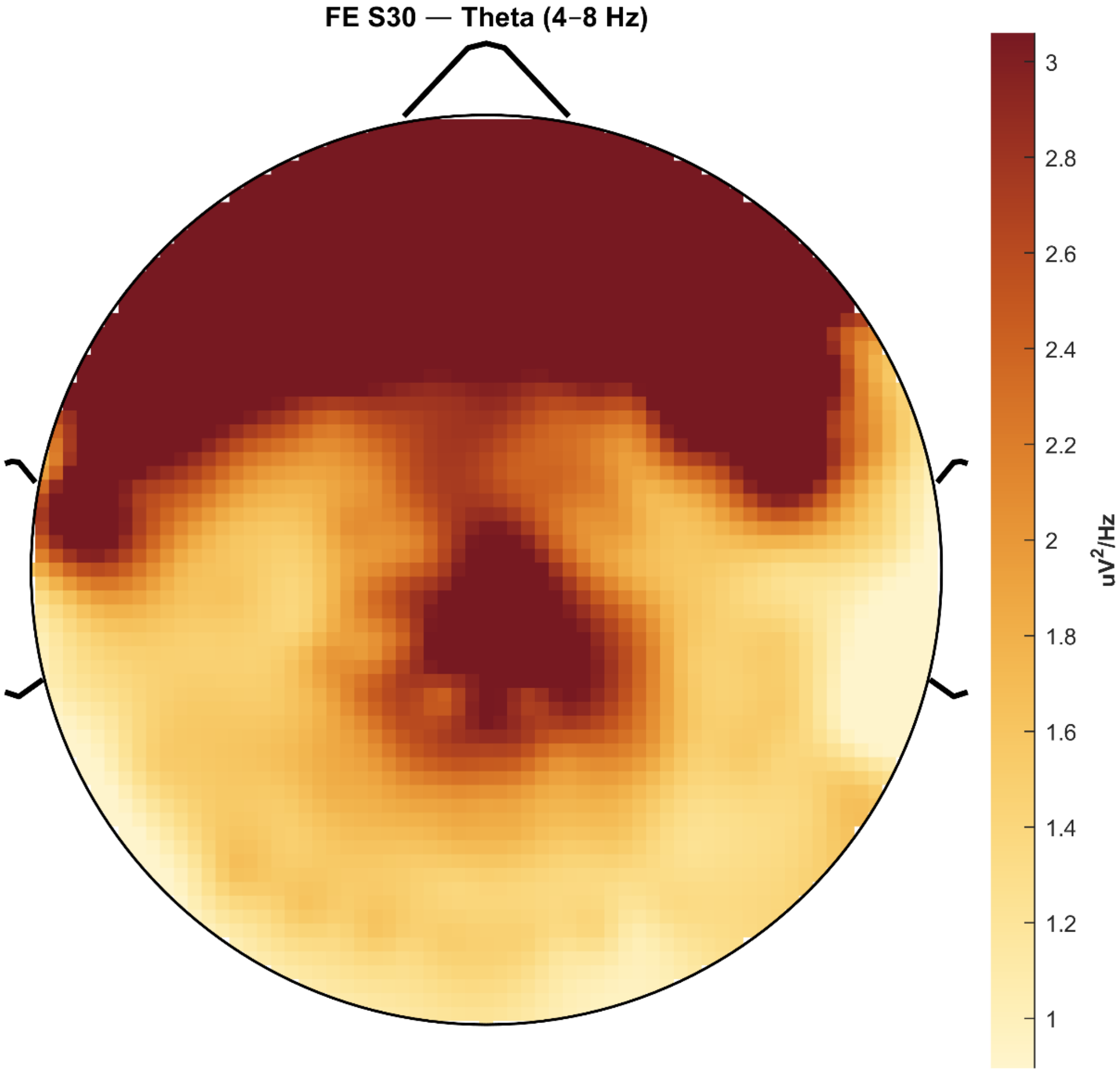

Supplementary Figure 70

Theta (4–8 Hz) power map for FE subject S30. The color scale represents theta power (µV²/Hz).

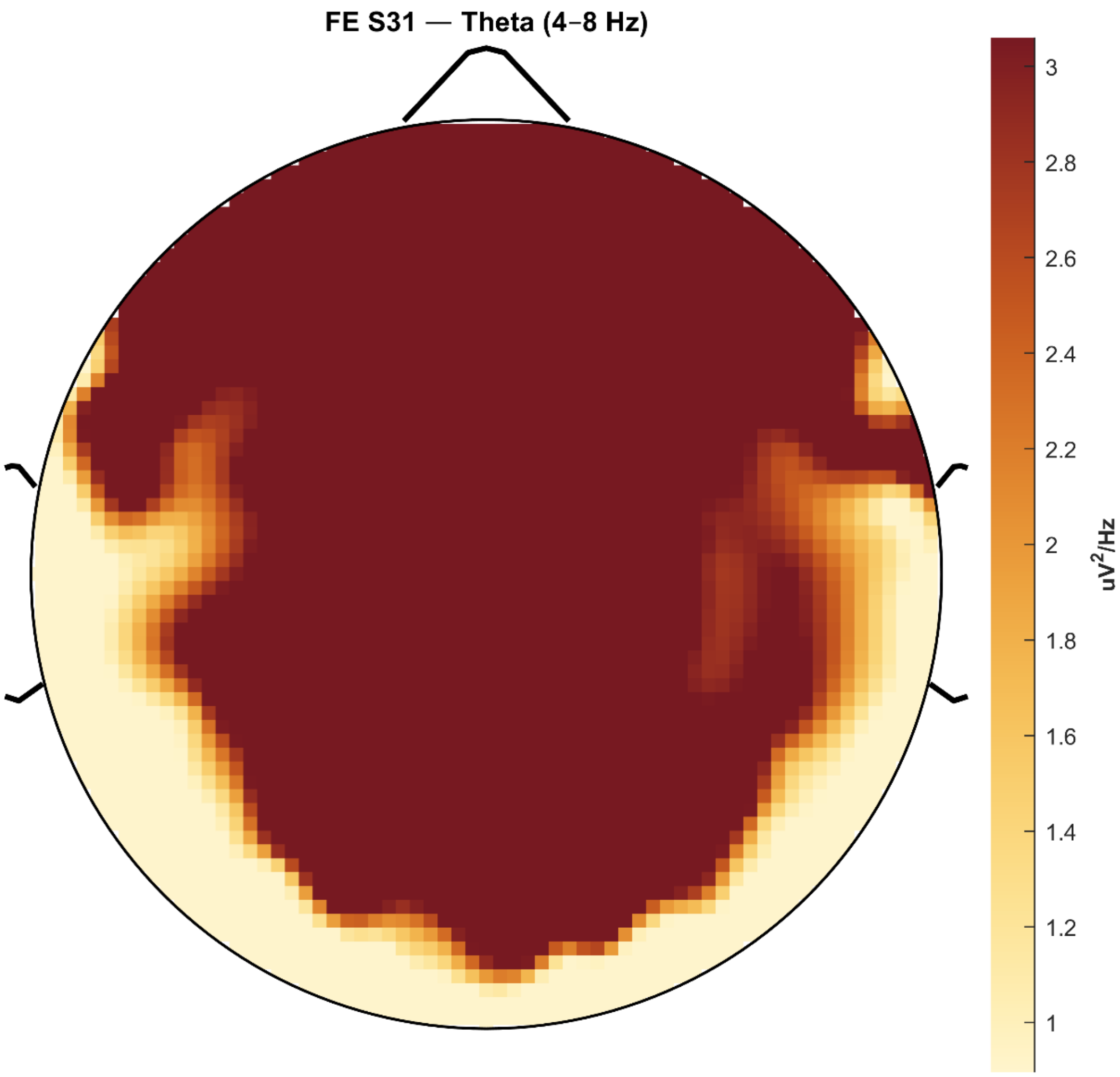

Supplementary Figure 71

Theta (4–8 Hz) power map for FE subject S31. The color scale represents theta power (μV²/Hz).

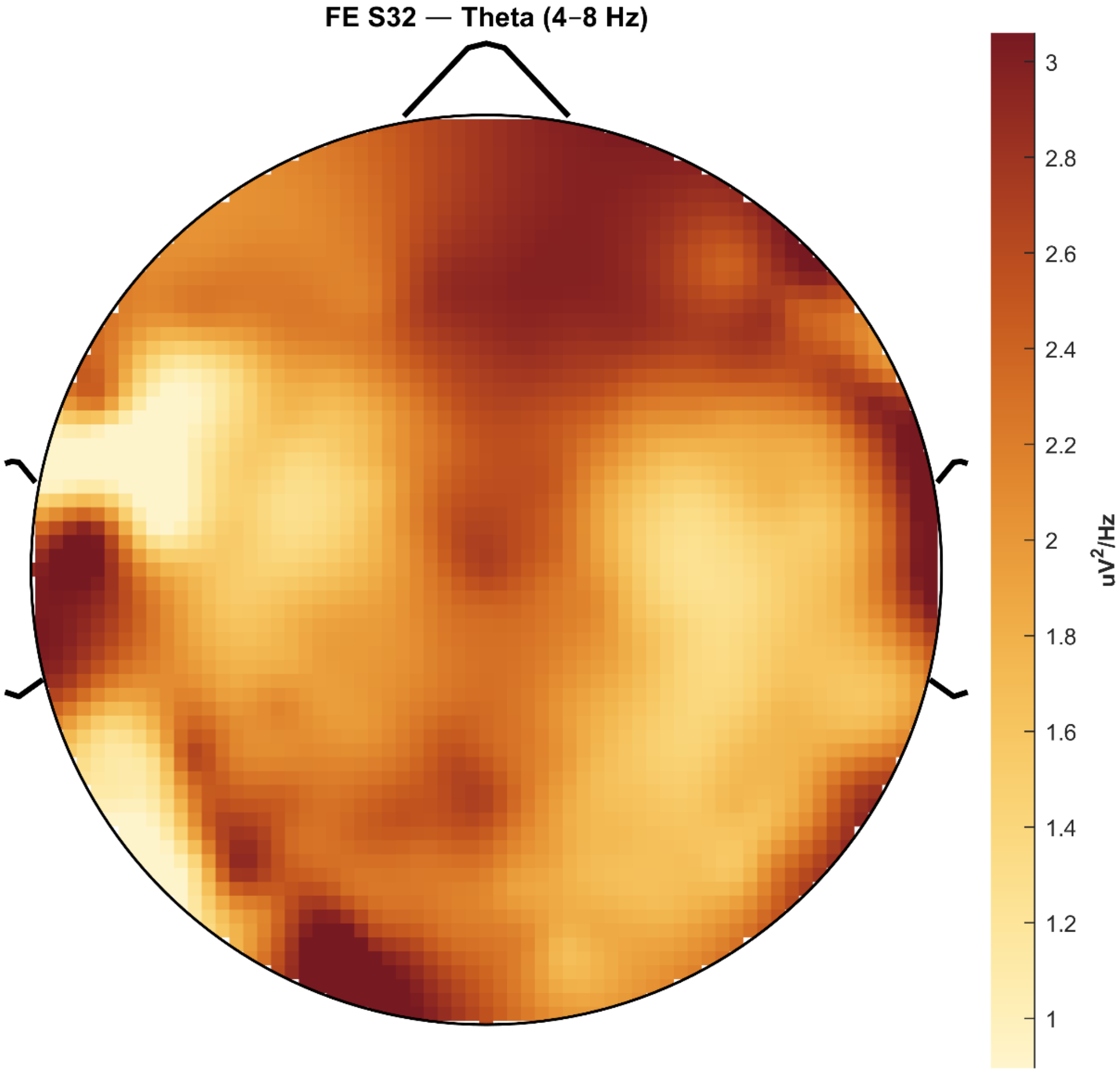

Supplementary Figure 72

Theta (4–8 Hz) power map for FE subject S32. The color scale represents theta power (μV²/Hz).

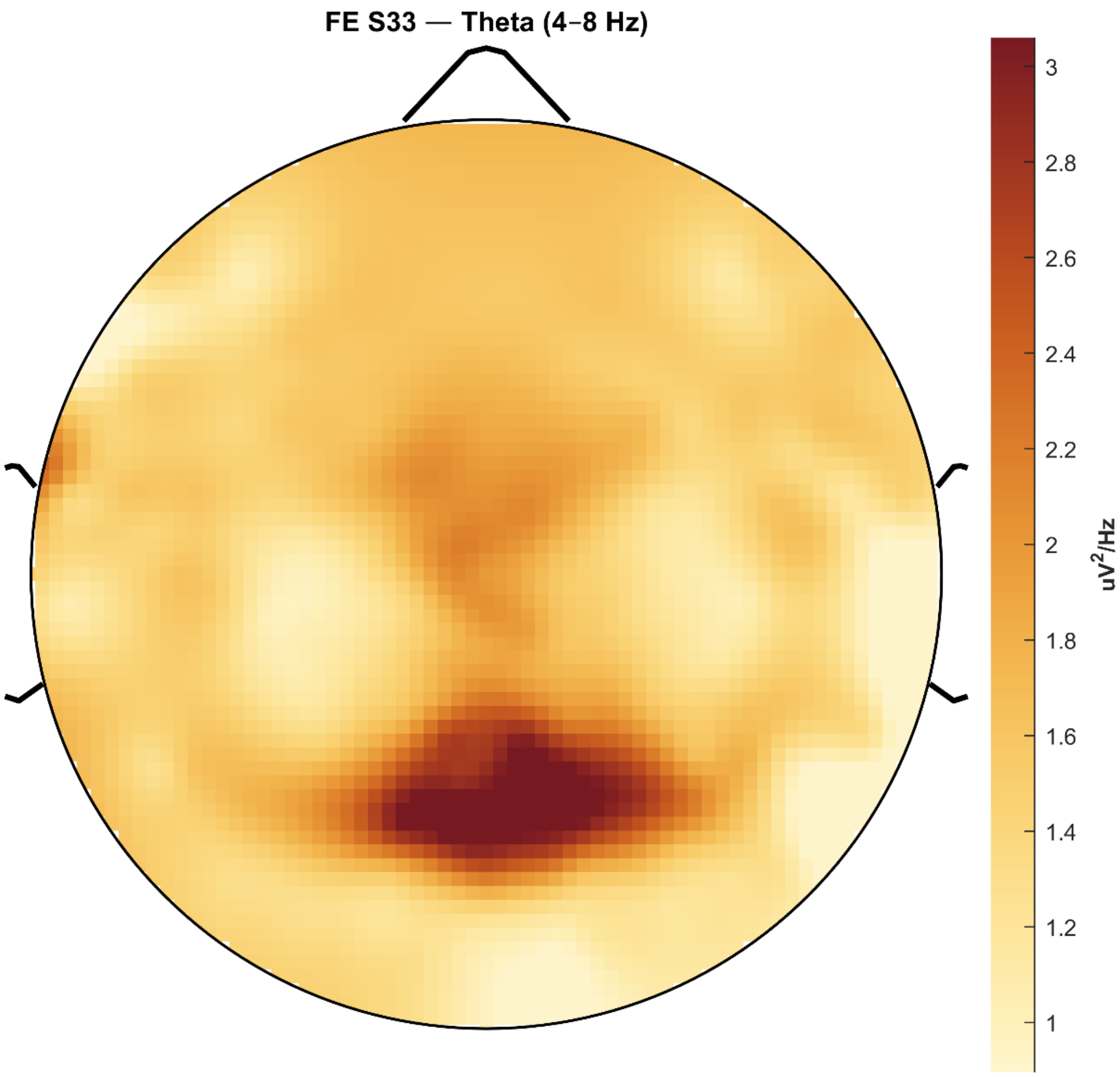

Supplementary Figure 73

Theta (4–8 Hz) power map for FE subject S33. The color scale represents theta power (μV²/Hz).

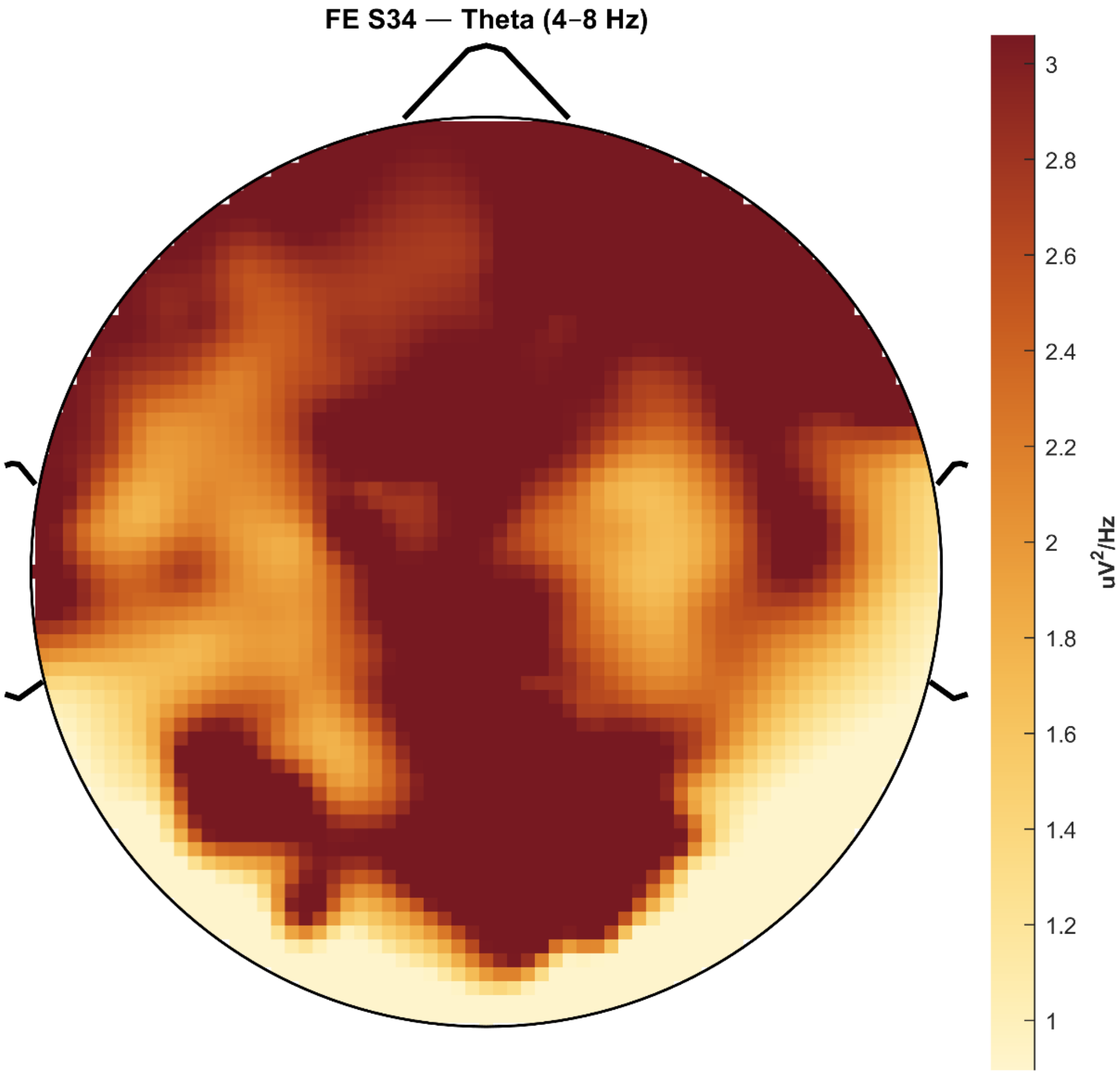

Supplementary Figure 74

Theta (4–8 Hz) power map for FE subject S34. The color scale represents theta power (µV²/Hz).

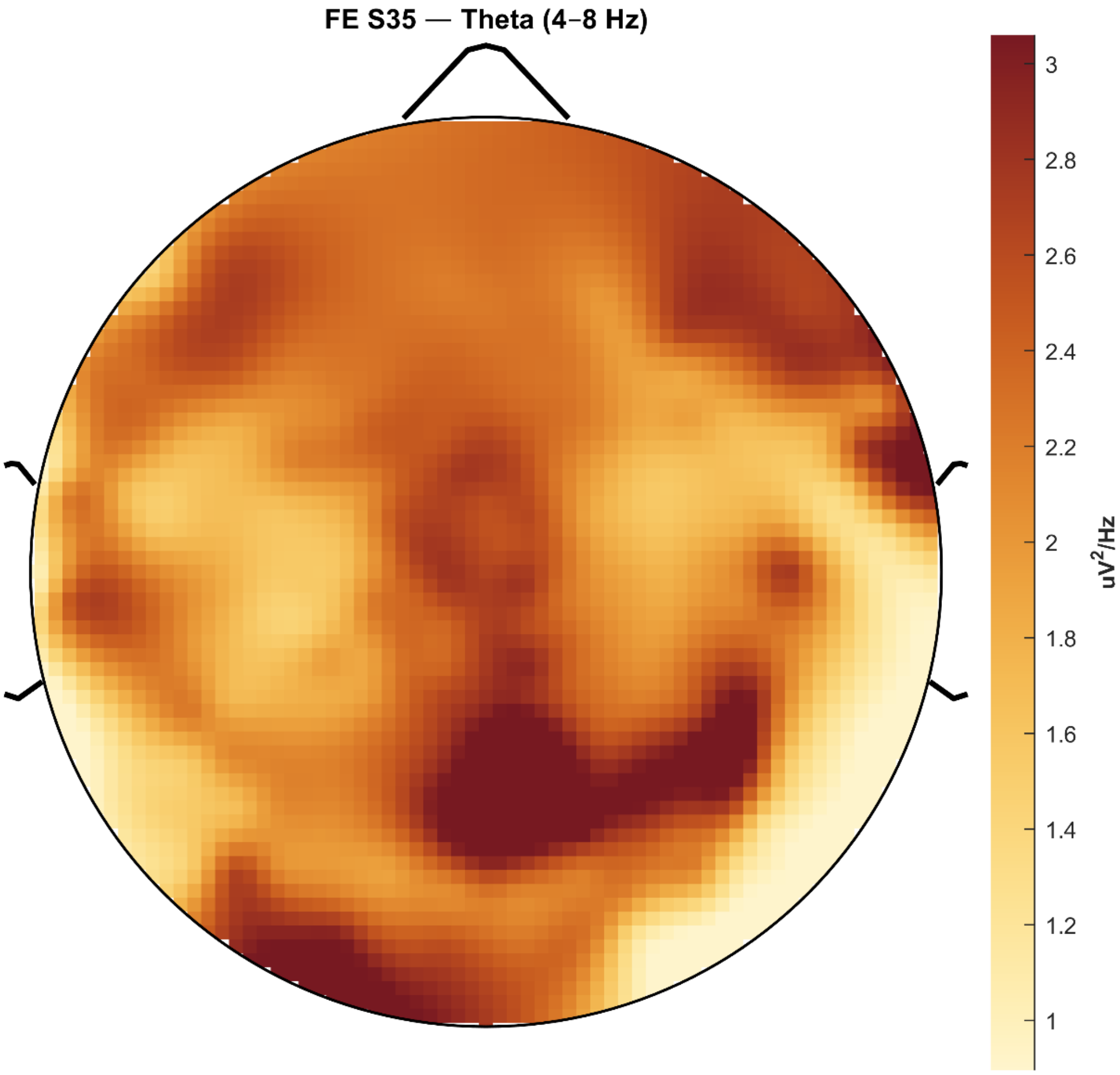

Supplementary Figure 75

Theta (4–8 Hz) power map for FE subject S35. The color scale represents theta power (µV²/Hz).

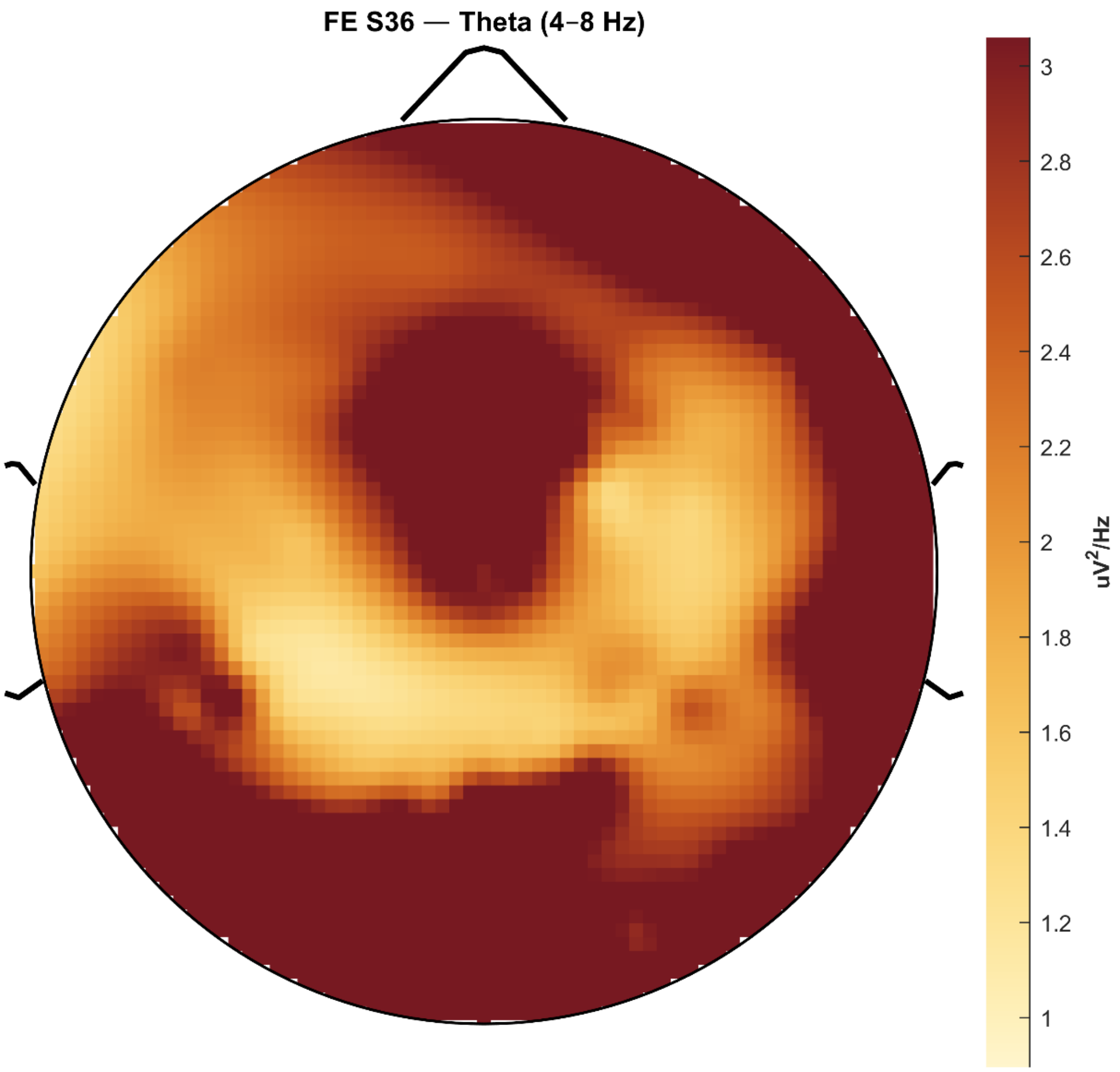

Supplementary Figure 76

Theta (4–8 Hz) power map for FE subject S36. The color scale represents theta power (µV²/Hz).

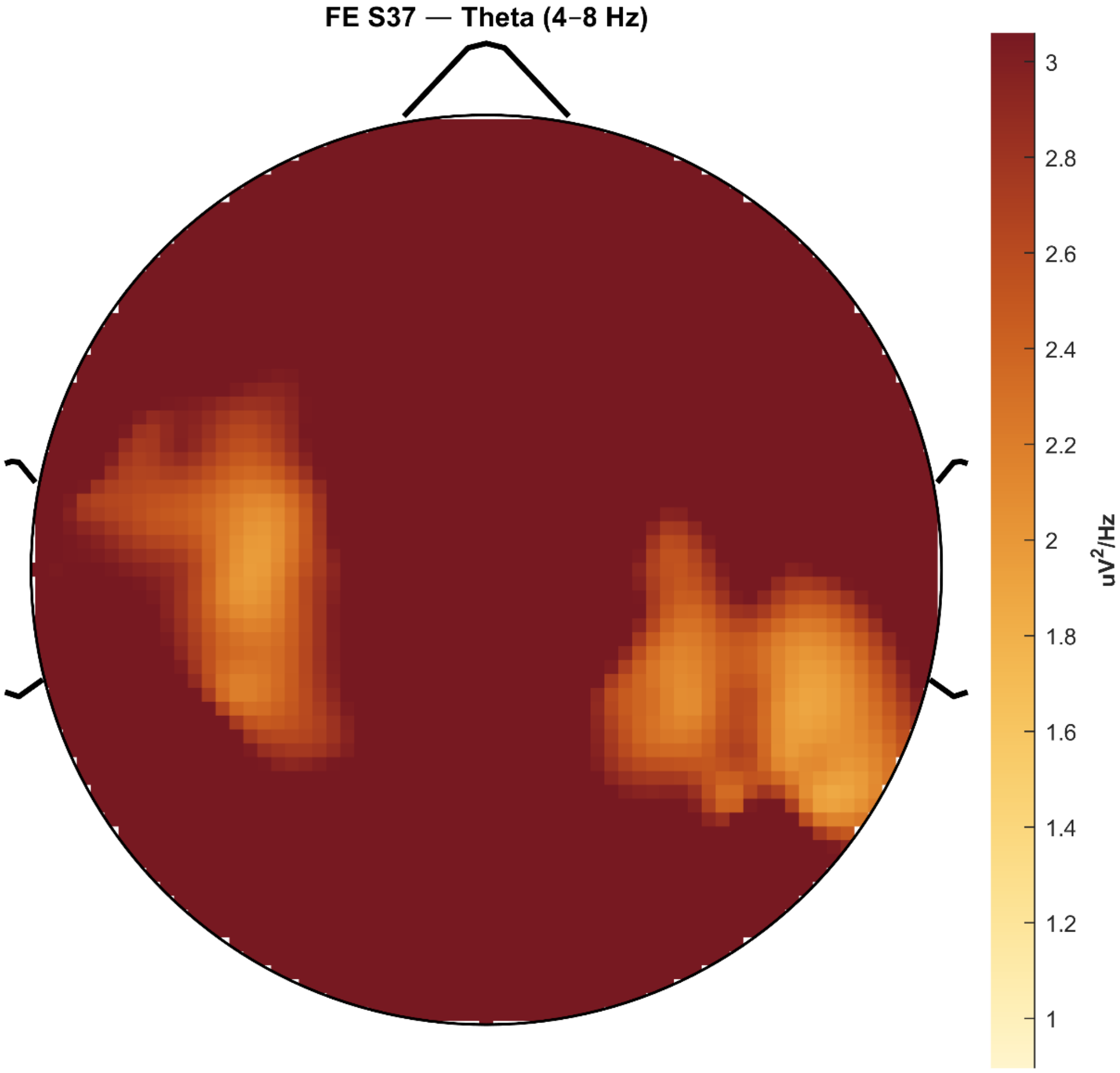

Supplementary Figure 77

Theta (4–8 Hz) power map for FE subject S37. The color scale represents theta power (µV²/Hz).

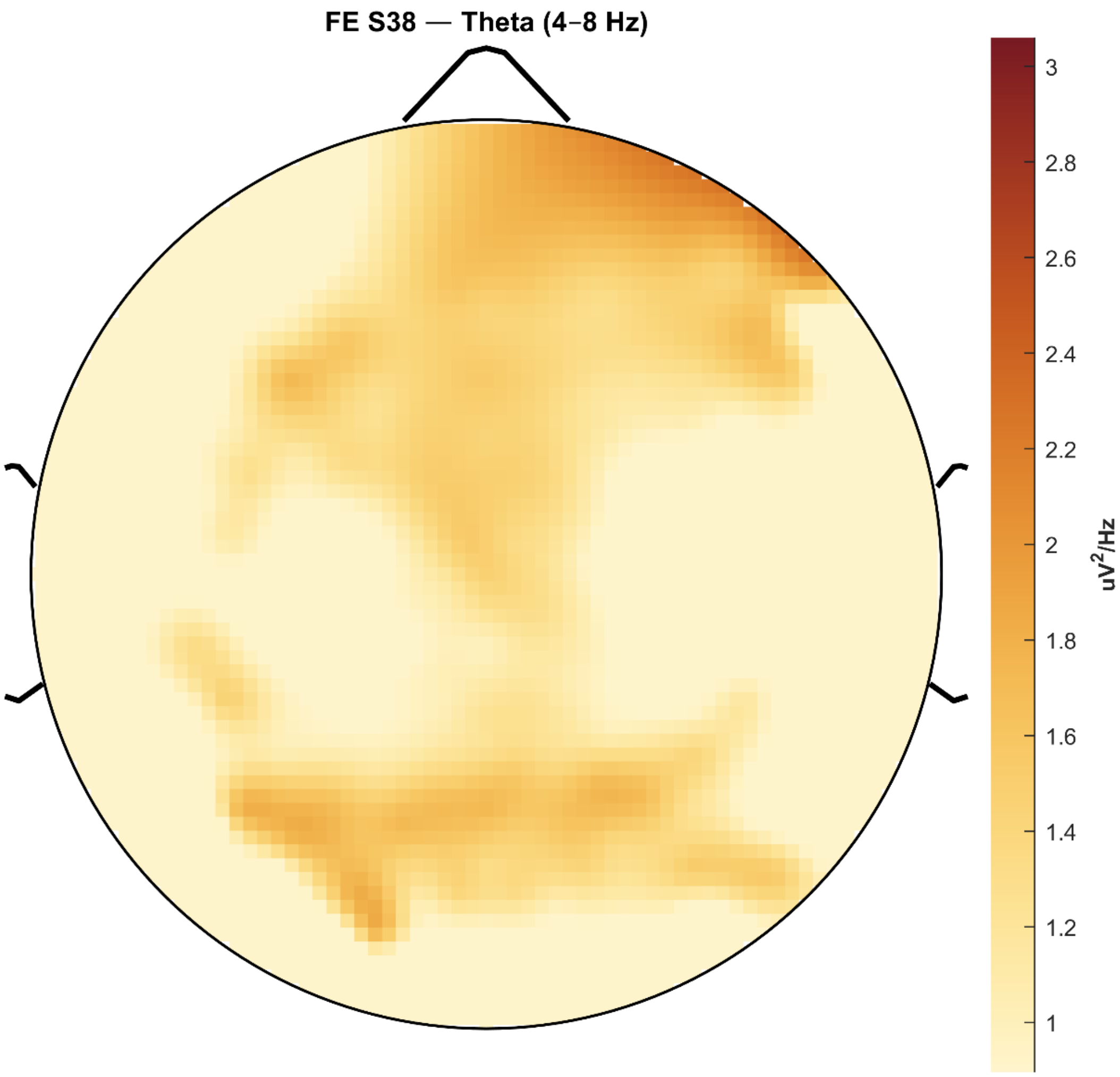

Supplementary Figure 78

Theta (4–8 Hz) power map for FE subject S38. The color scale represents theta power (μV²/Hz).

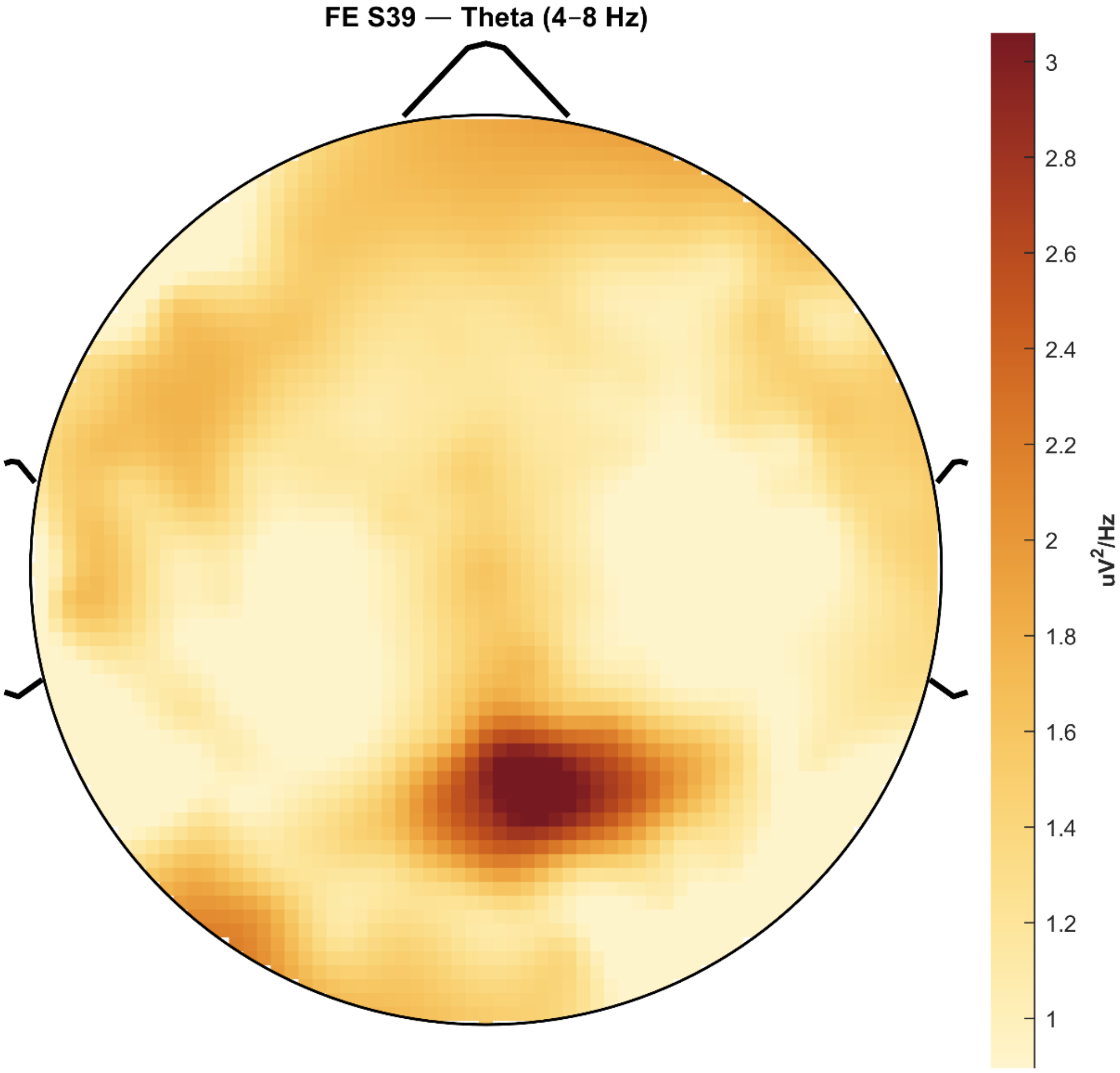

Supplementary Figure 79

Theta (4–8 Hz) power map for FE subject S39. The color scale represents theta power (μV²/Hz).

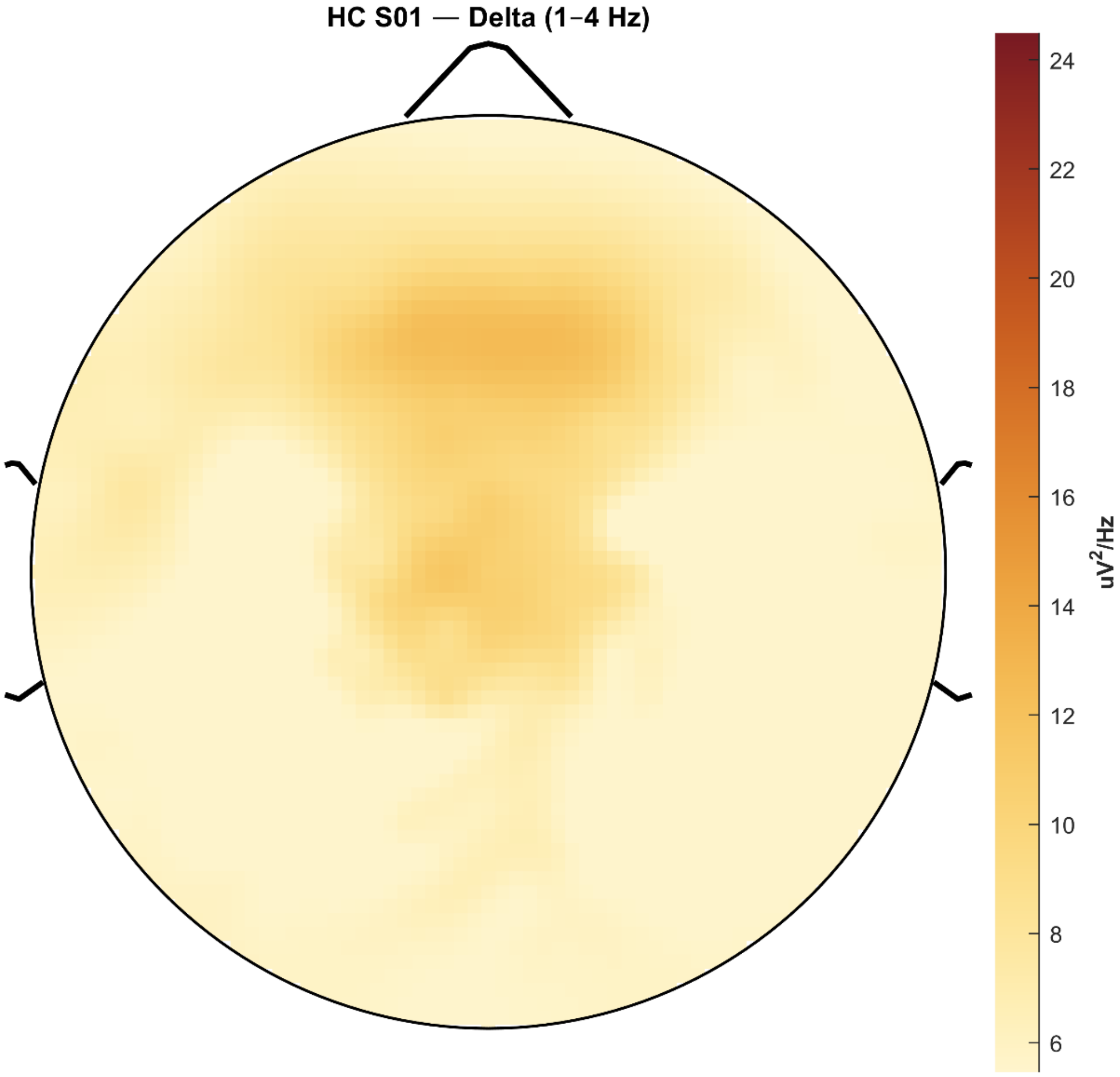

Supplementary Figure 80

Delta (1-4 Hz) power map for HC subject S01. The color scale represents delta power (μV²/Hz).

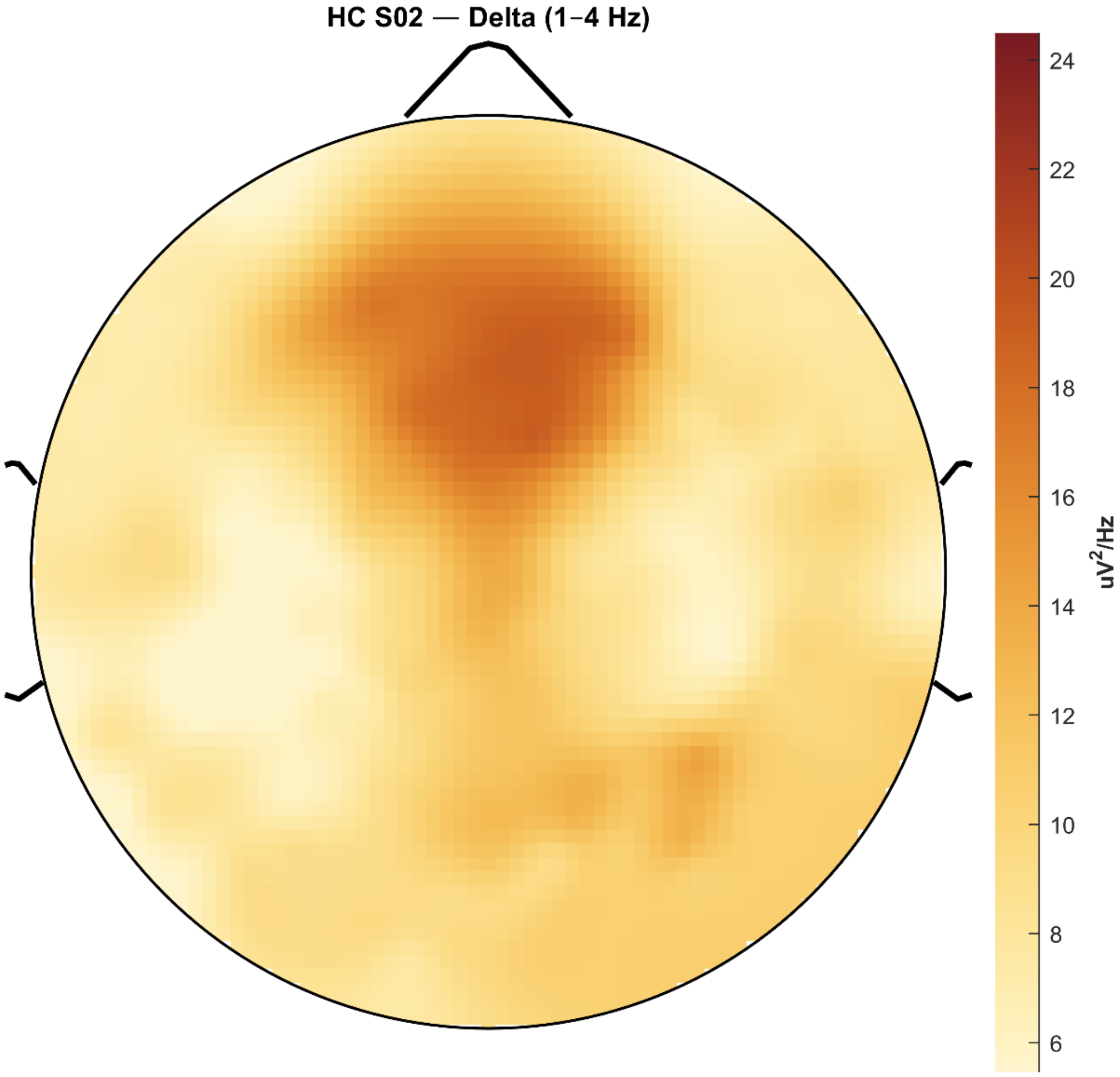

Supplementary Figure 81

Delta (1-4 Hz) power map for HC subject S02. The color scale represents delta power (μV²/Hz).

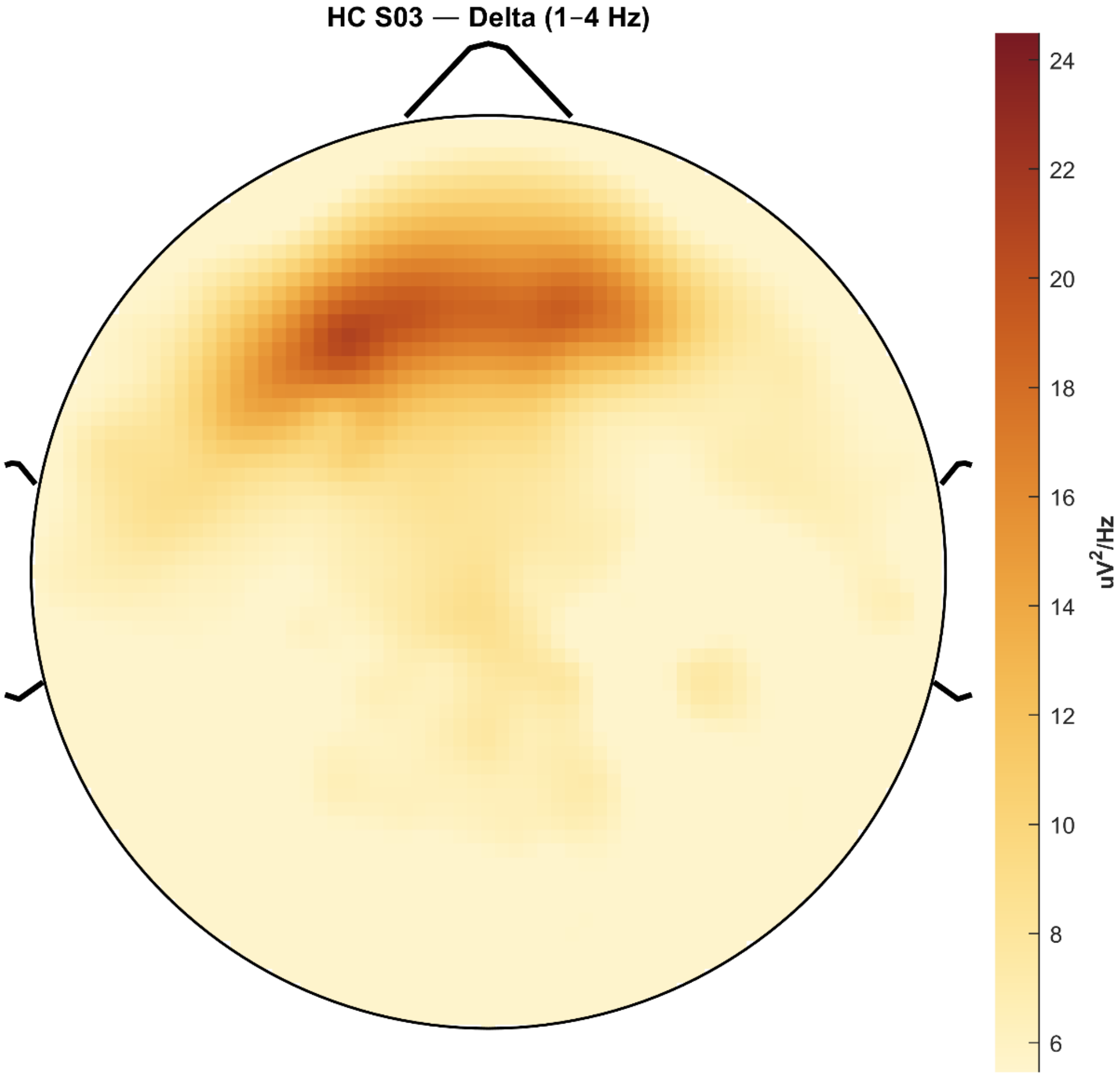

Supplementary Figure 82

Delta (1-4 Hz) power map for HC subject S03. The color scale represents delta power (μV²/Hz).

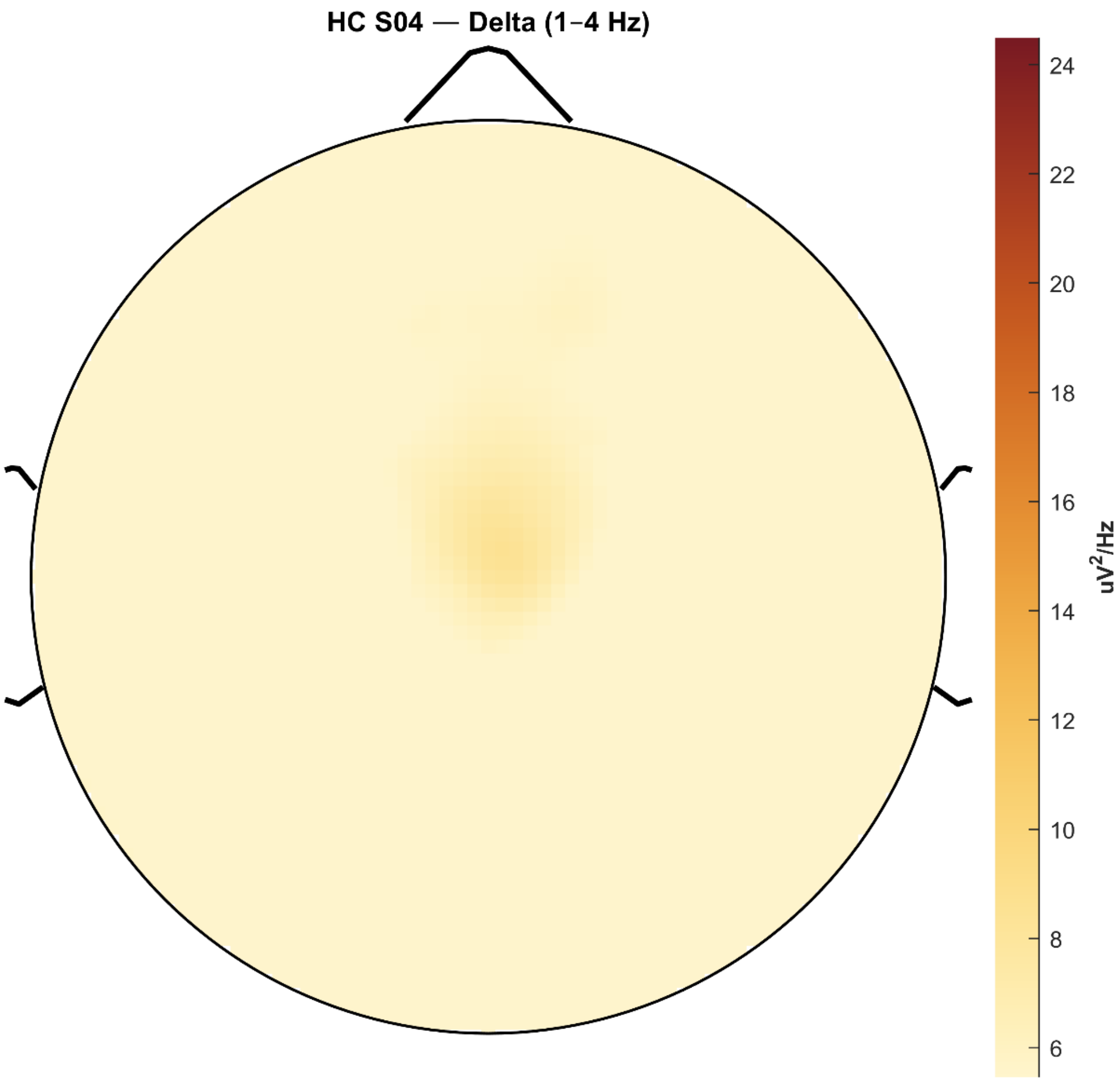

Supplementary Figure 83

Delta (1-4 Hz) power map for HC subject S04. The color scale represents delta power (μV²/Hz).

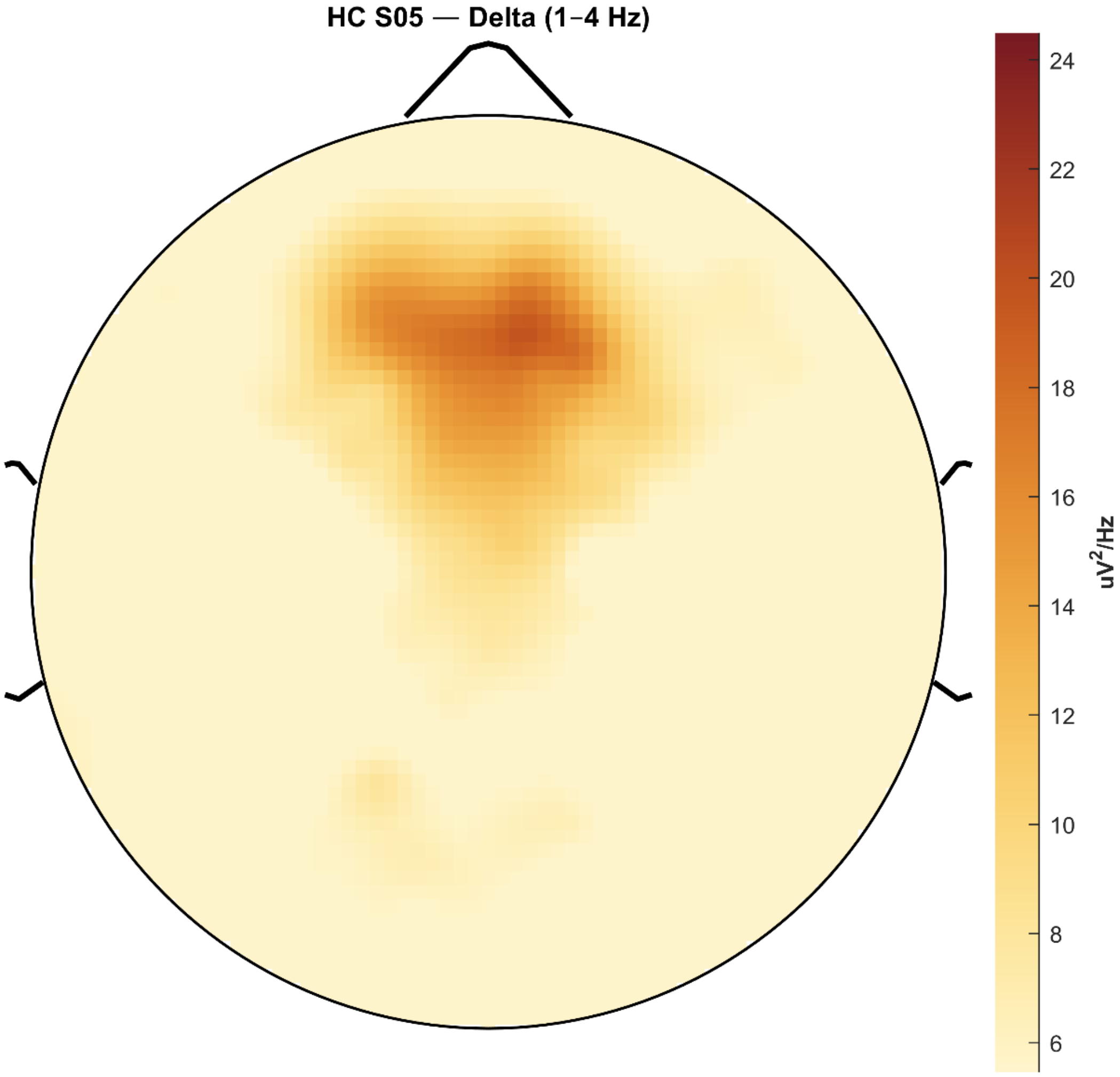

Supplementary Figure 84

Delta (1-4 Hz) power map for HC subject S05. The color scale represents delta power (μV²/Hz).

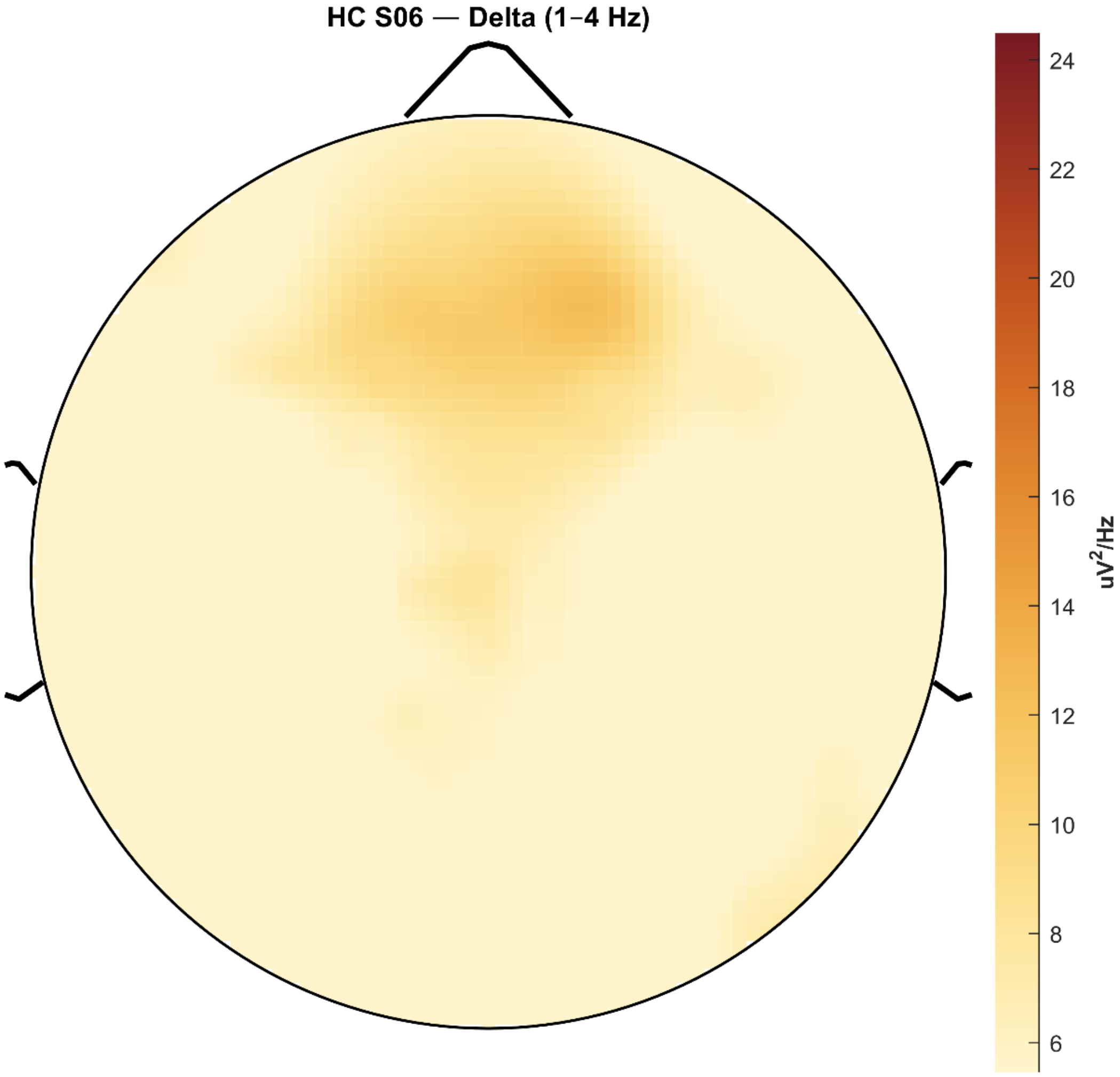

Supplementary Figure 85

Delta (1-4 Hz) power map for HC subject S06. The color scale represents delta power (μV²/Hz).

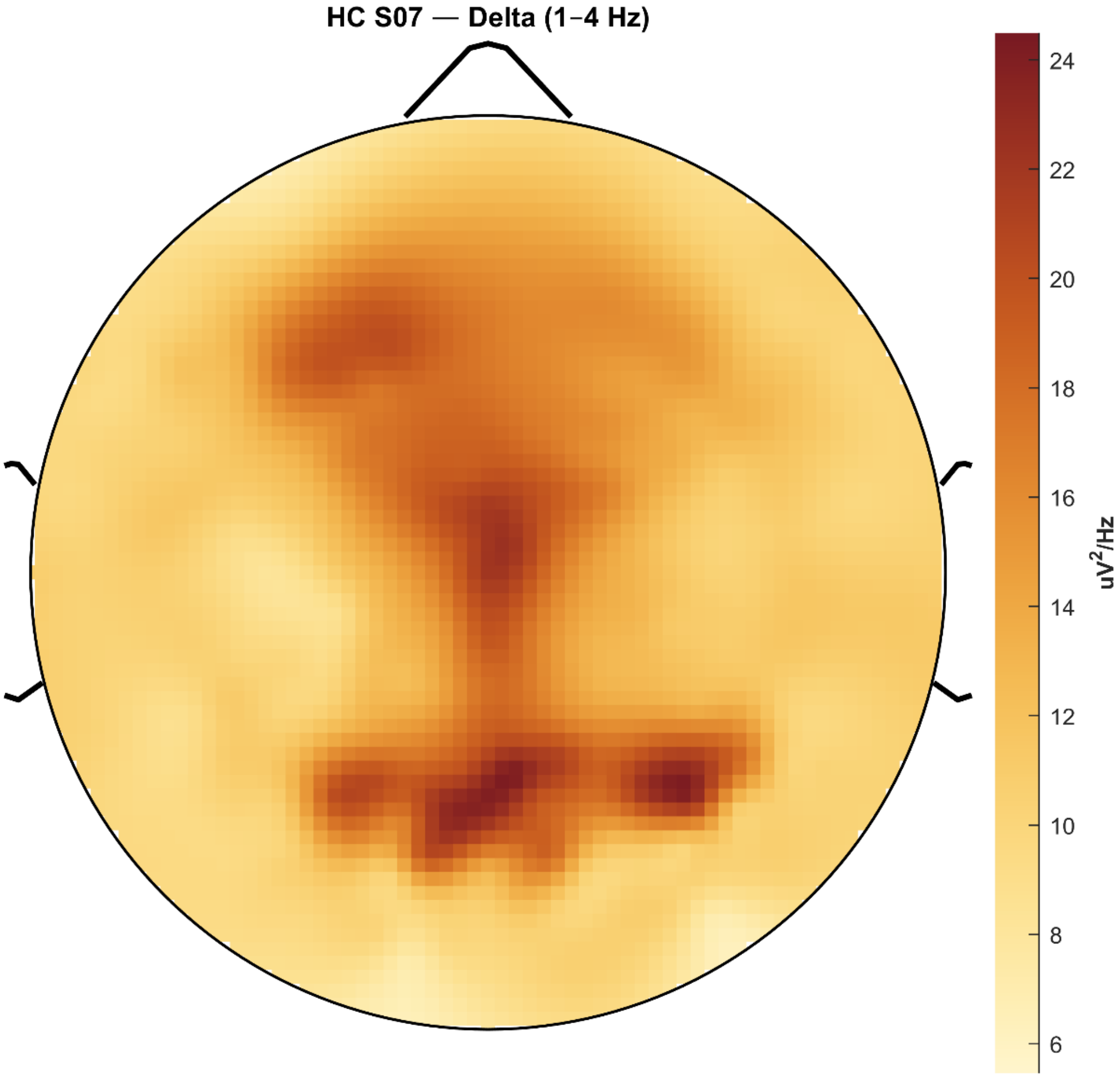

Supplementary Figure 86

Delta (1-4 Hz) power map for HC subject S07. The color scale represents delta power ($\mu V^2/Hz$).

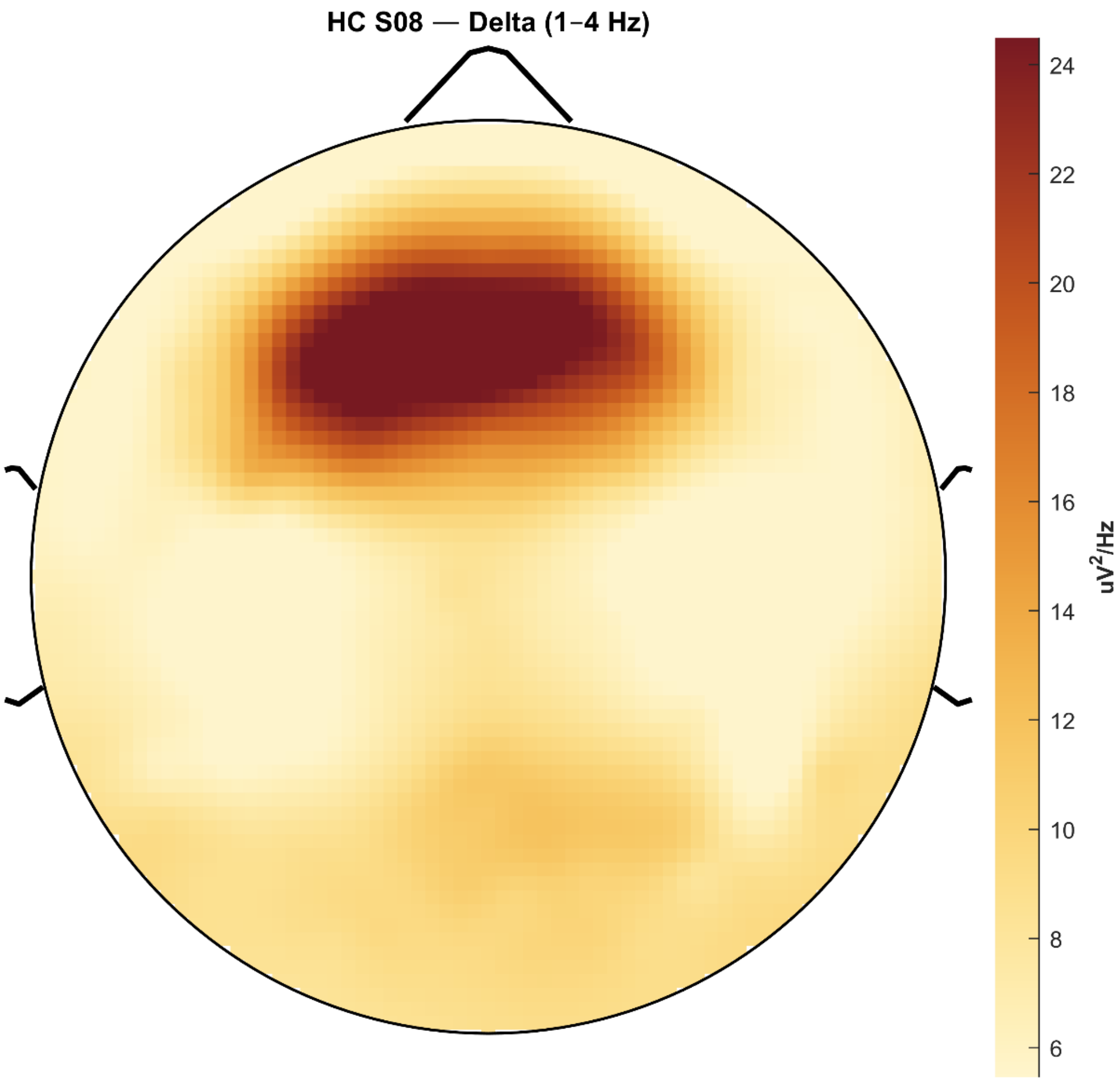

Supplementary Figure 87

Delta (1-4 Hz) power map for HC subject S08. The color scale represents delta power (μV²/Hz).

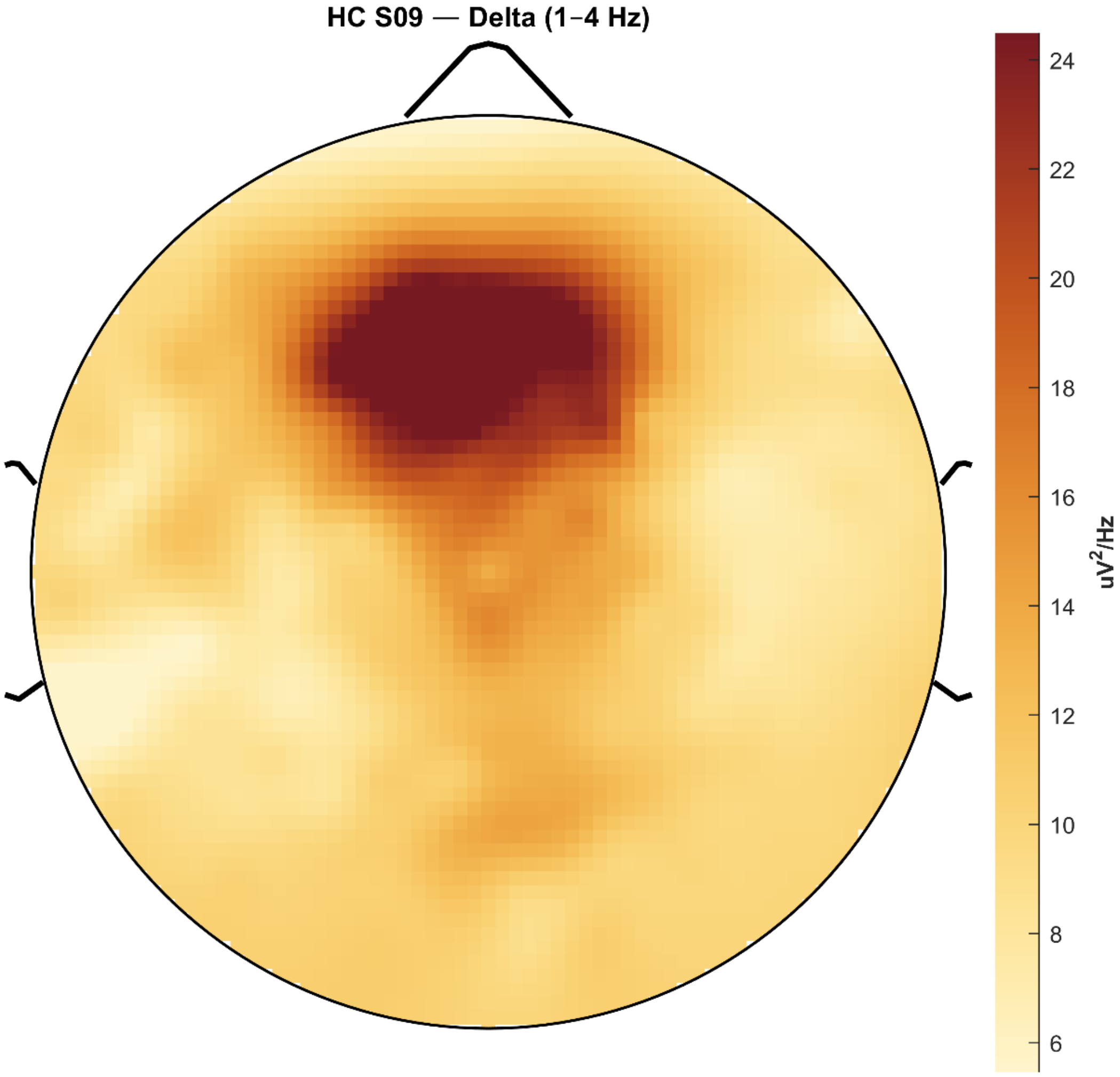

Supplementary Figure 88

Delta (1-4 Hz) power map for HC subject S09. The color scale represents delta power (µV²/Hz).

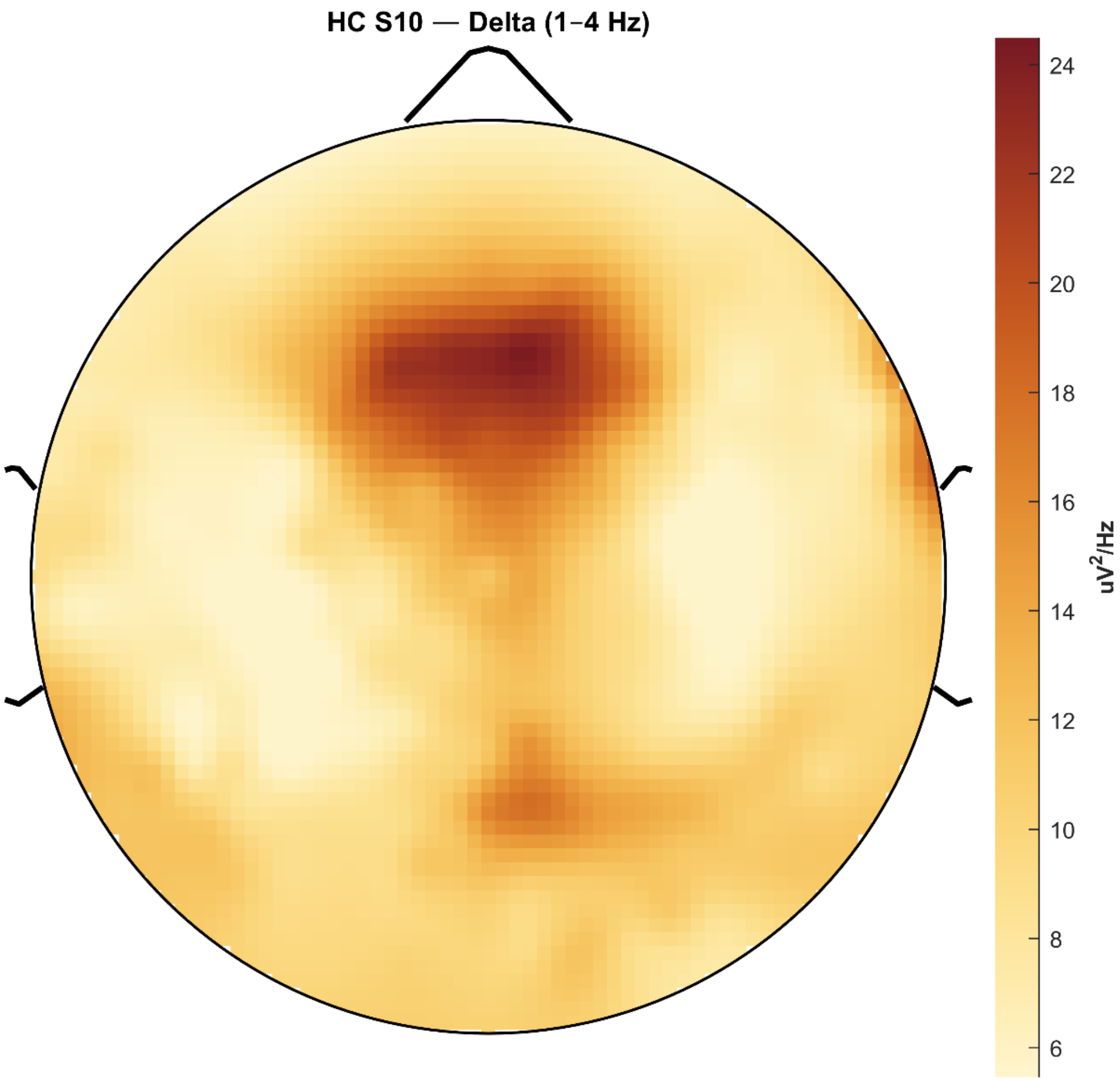

Supplementary Figure 89

Delta (1-4 Hz) power map for HC subject S10. The color scale represents delta power (μV²/Hz).

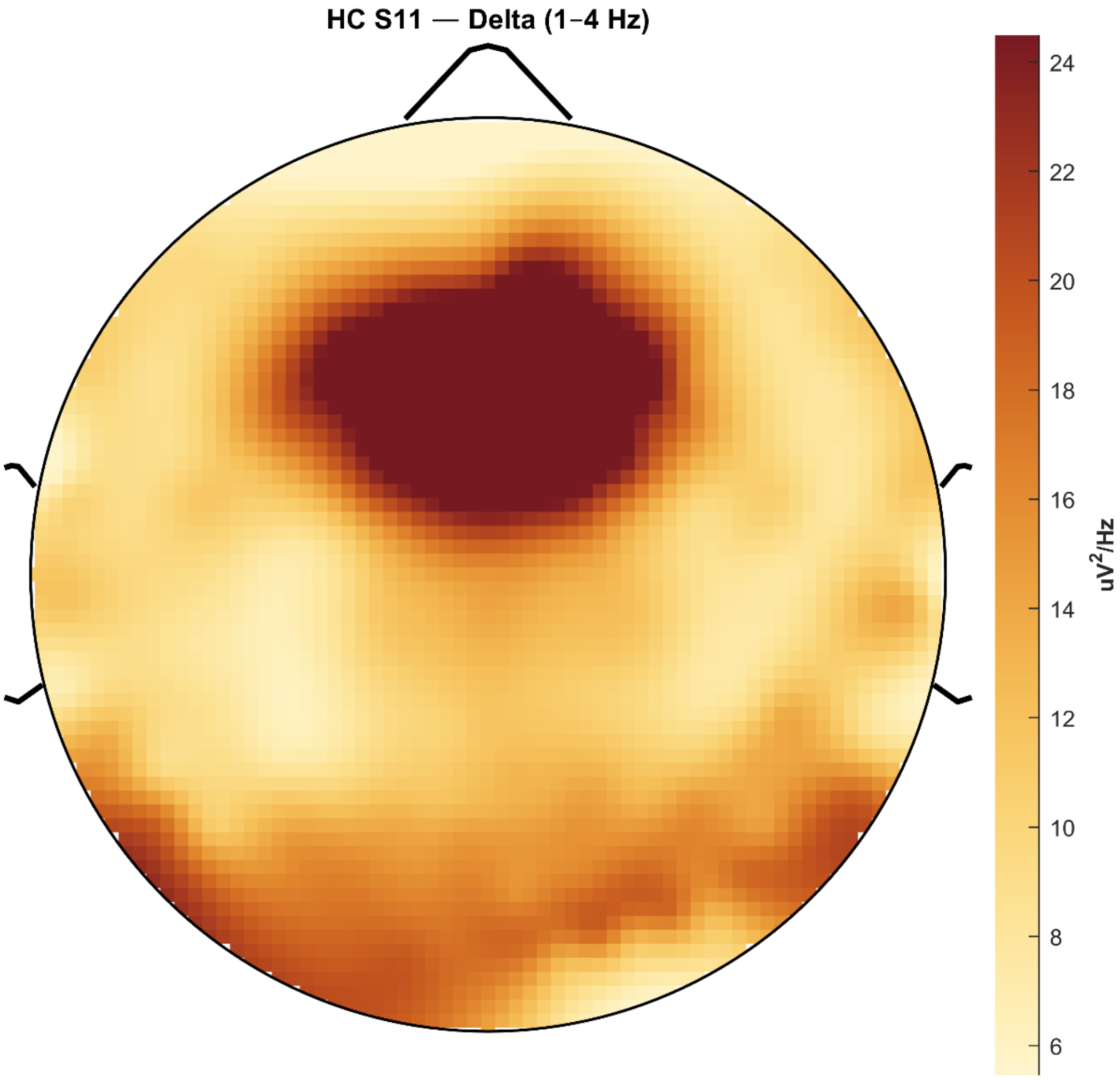

Supplementary Figure 90

Delta (1-4 Hz) power map for HC subject S11. The color scale represents delta power (μV²/Hz).

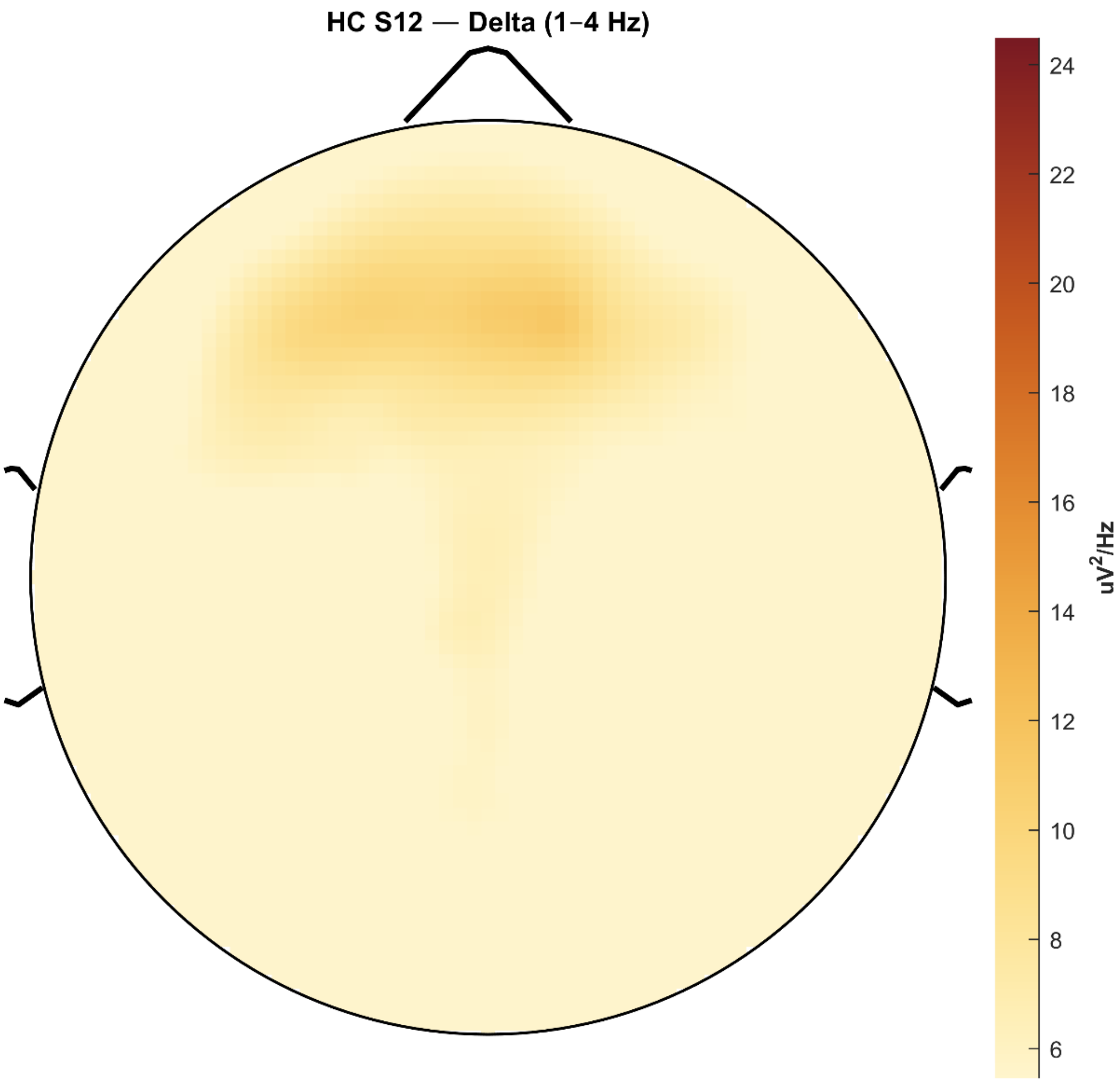

Supplementary Figure 91

Delta (1-4 Hz) power map for HC subject S12. The color scale represents delta power (μV²/Hz).

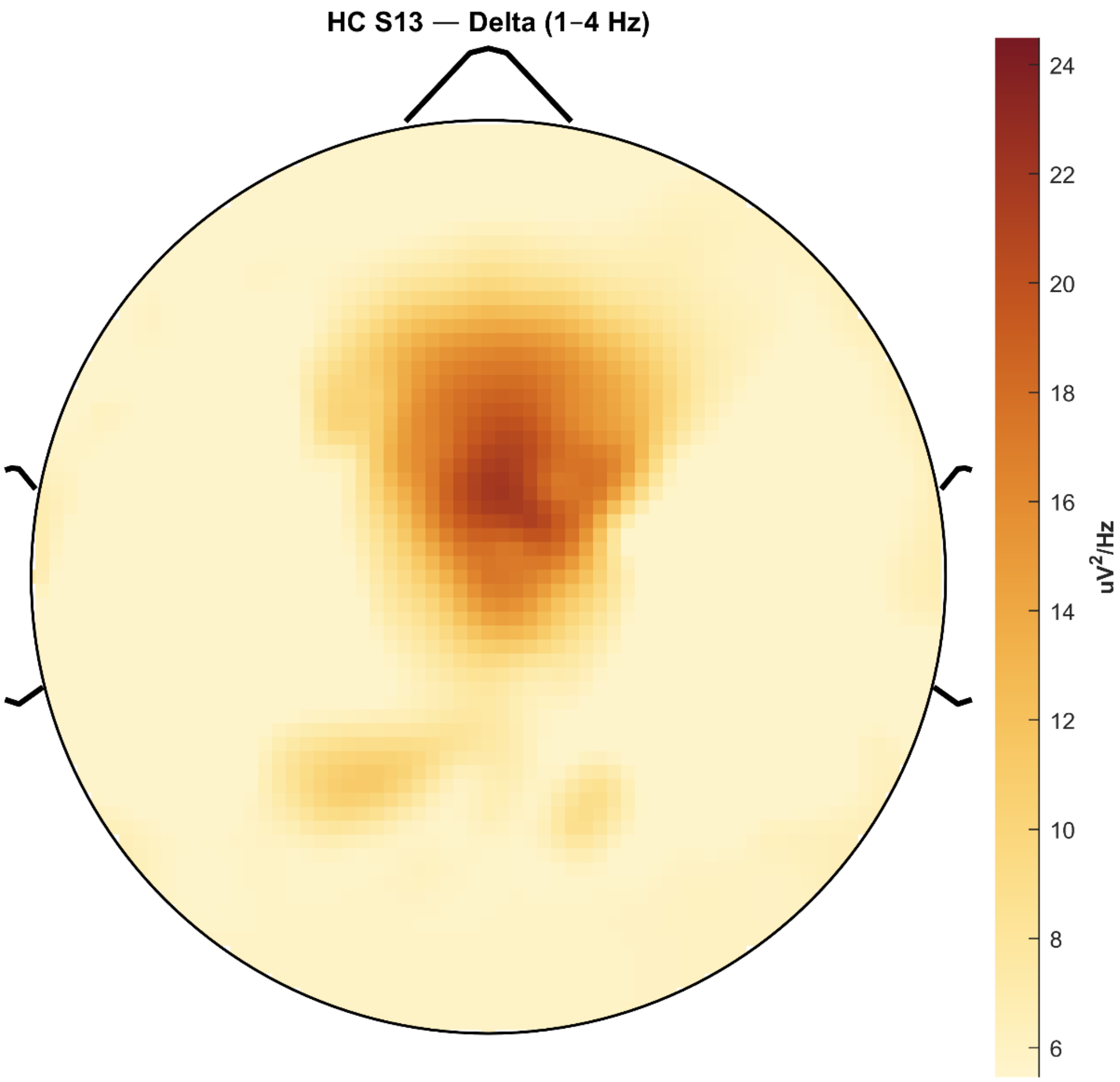

Supplementary Figure 92

Delta (1-4 Hz) power map for HC subject S13. The color scale represents delta power (μV²/Hz).

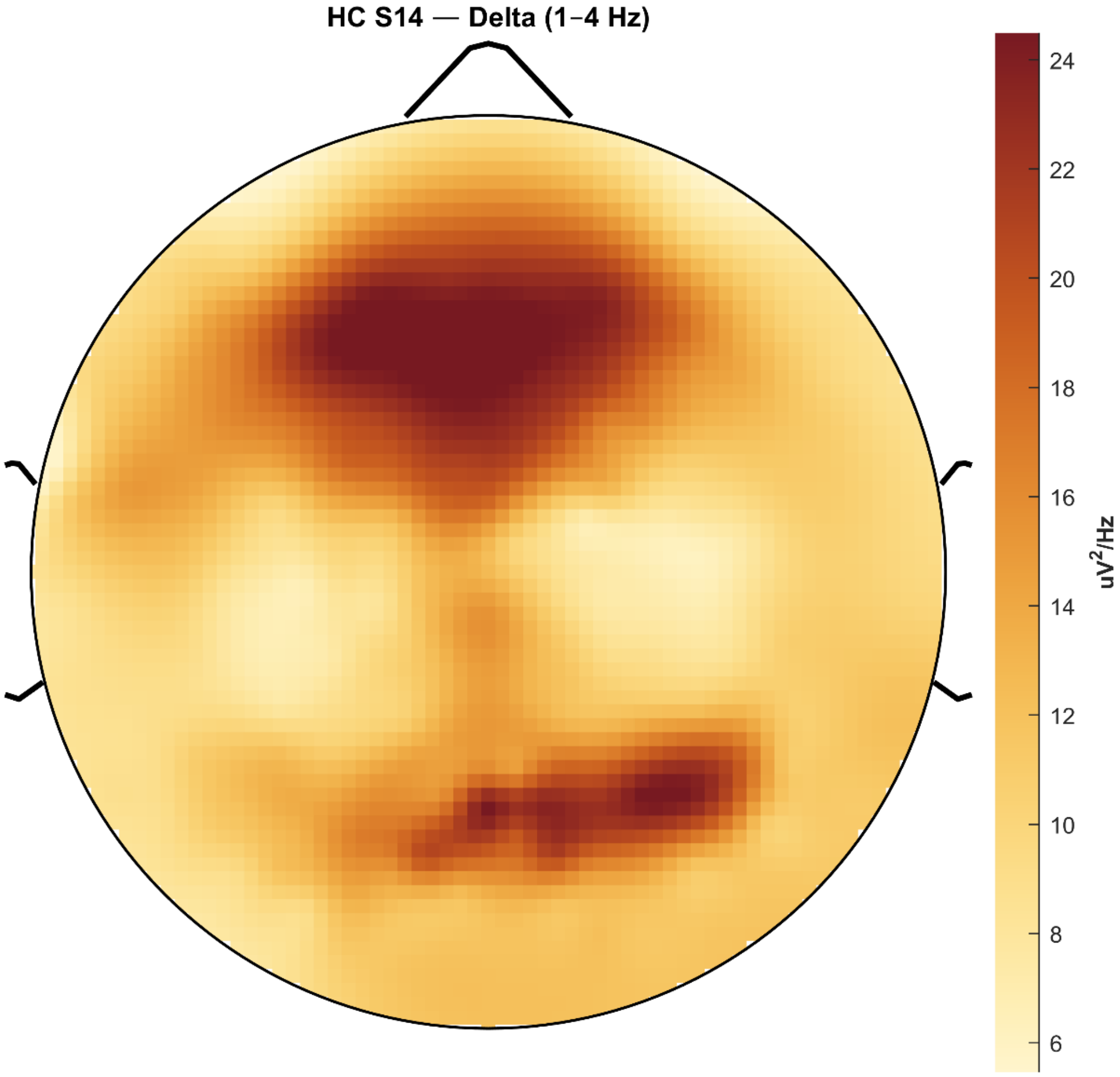

Supplementary Figure 93

Delta (1-4 Hz) power map for HC subject S14. The color scale represents delta power (μV²/Hz).

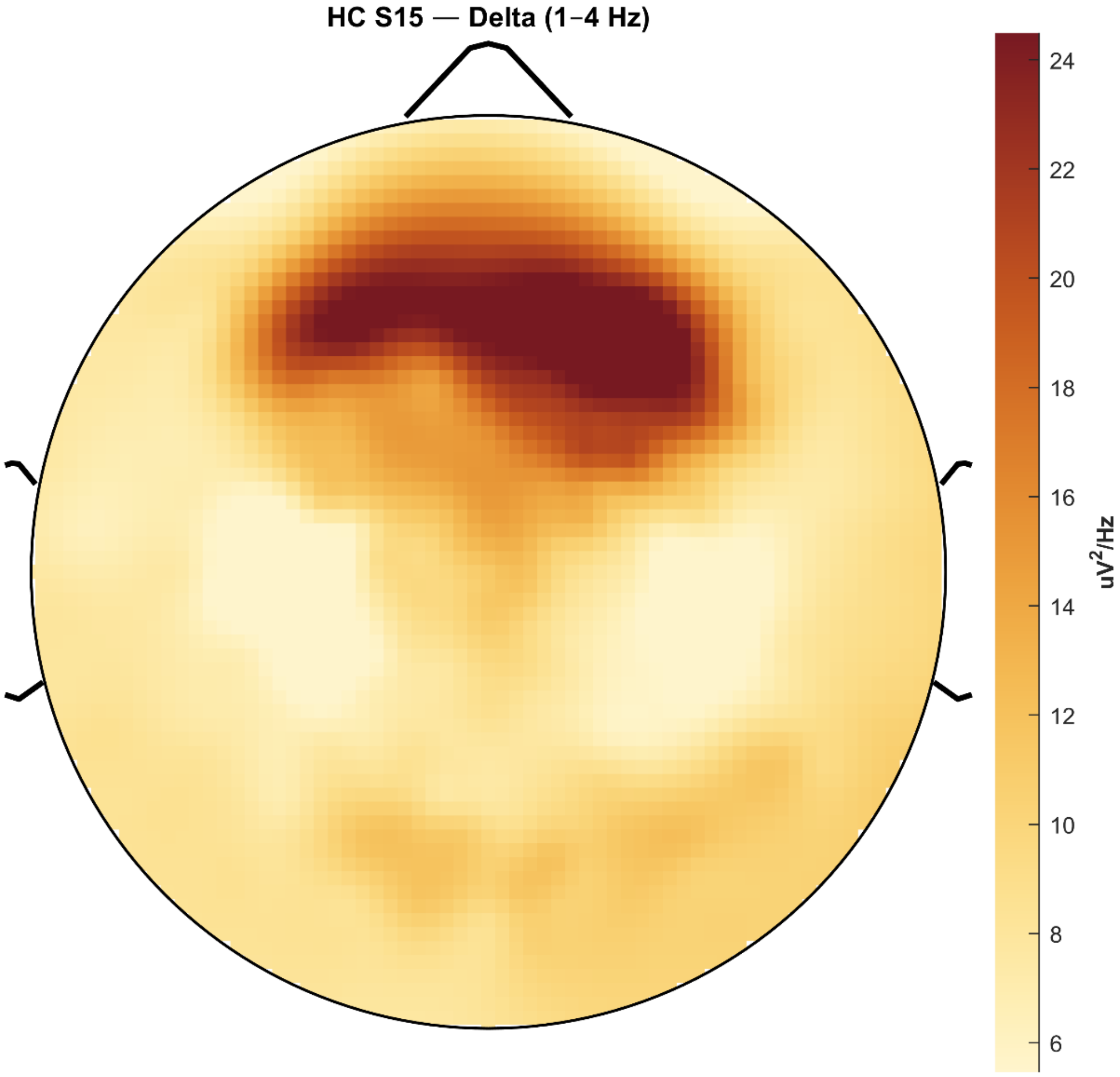

Supplementary Figure 94

Delta (1-4 Hz) power map for HC subject S15. The color scale represents delta power (μV²/Hz).

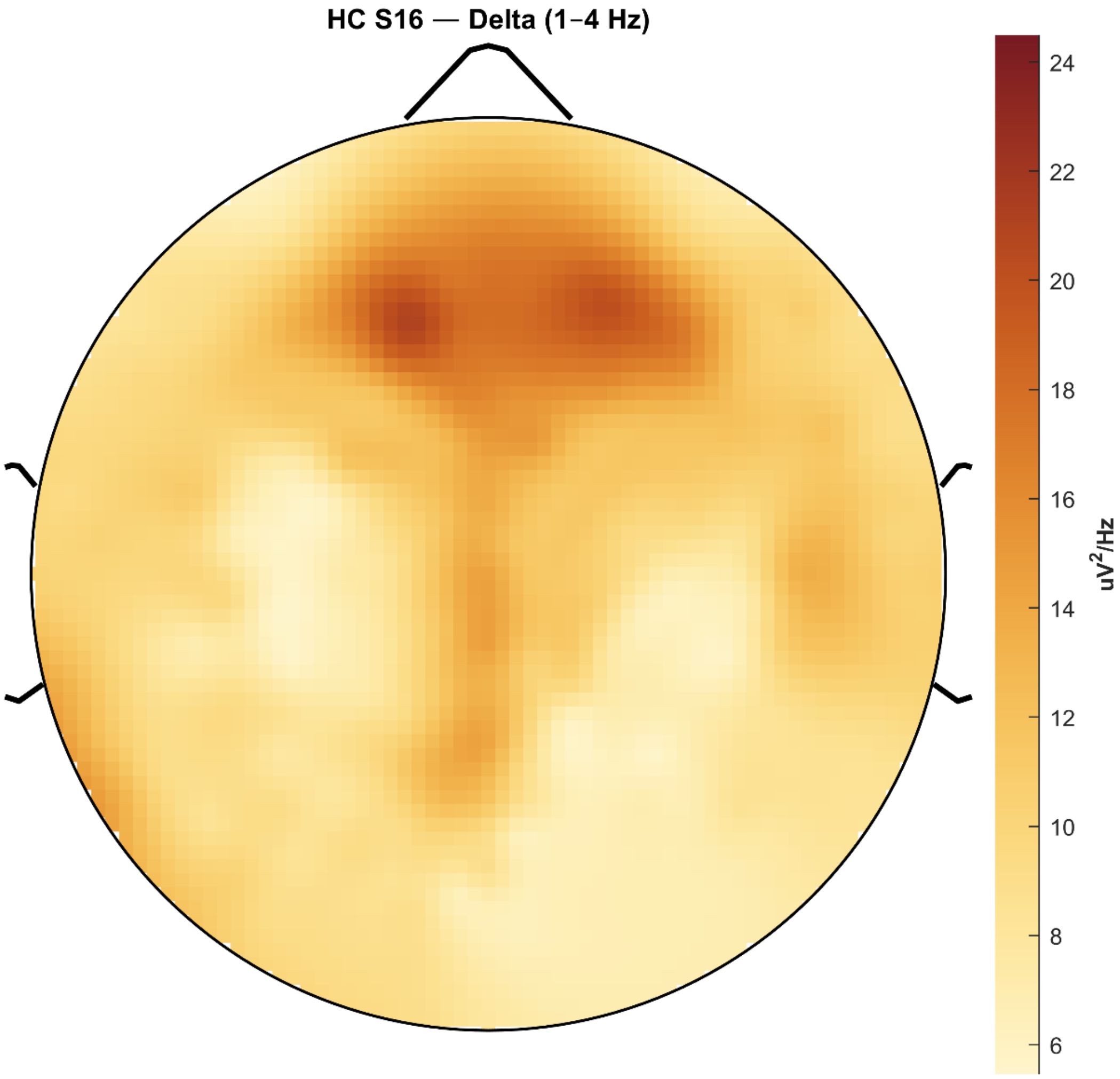

Supplementary Figure 95

Delta (1-4 Hz) power map for HC subject S16. The color scale represents delta power (μV²/Hz).

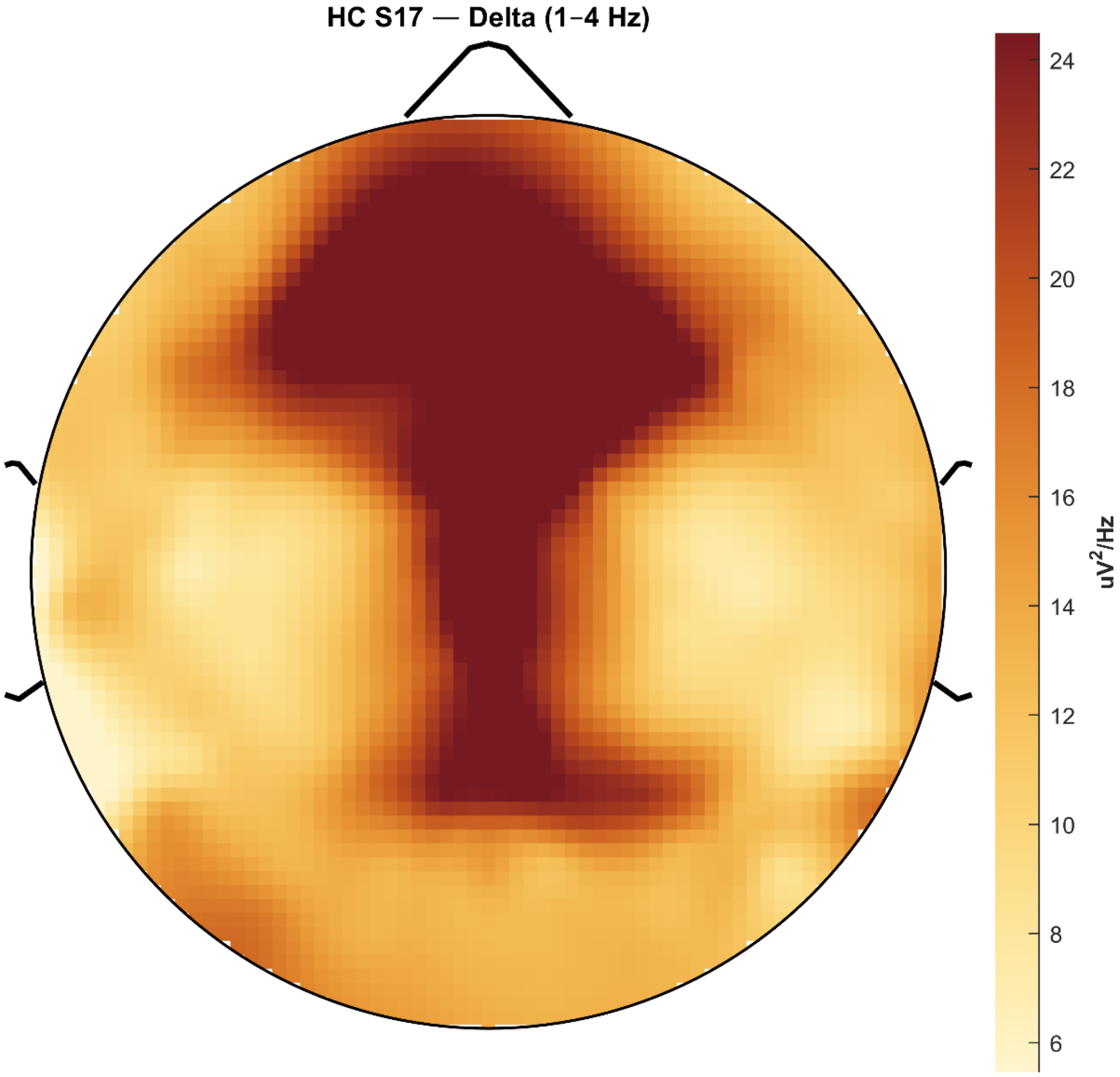

Supplementary Figure 96

Delta (1-4 Hz) power map for HC subject S17. The color scale represents delta power (μV²/Hz).

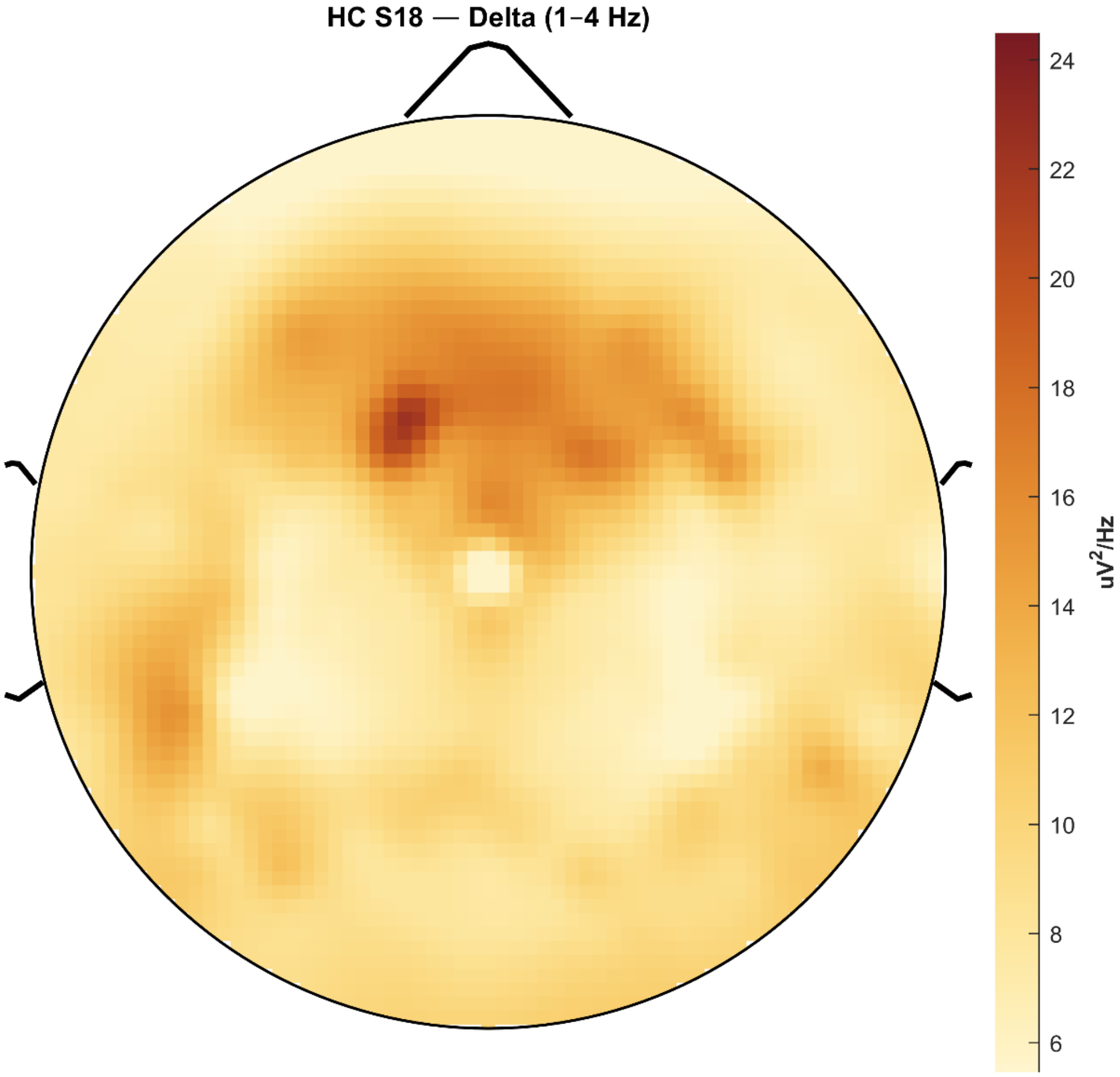

Supplementary Figure 97

Delta (1-4 Hz) power map for HC subject S18. The color scale represents delta power ($\mu V^2/Hz$).

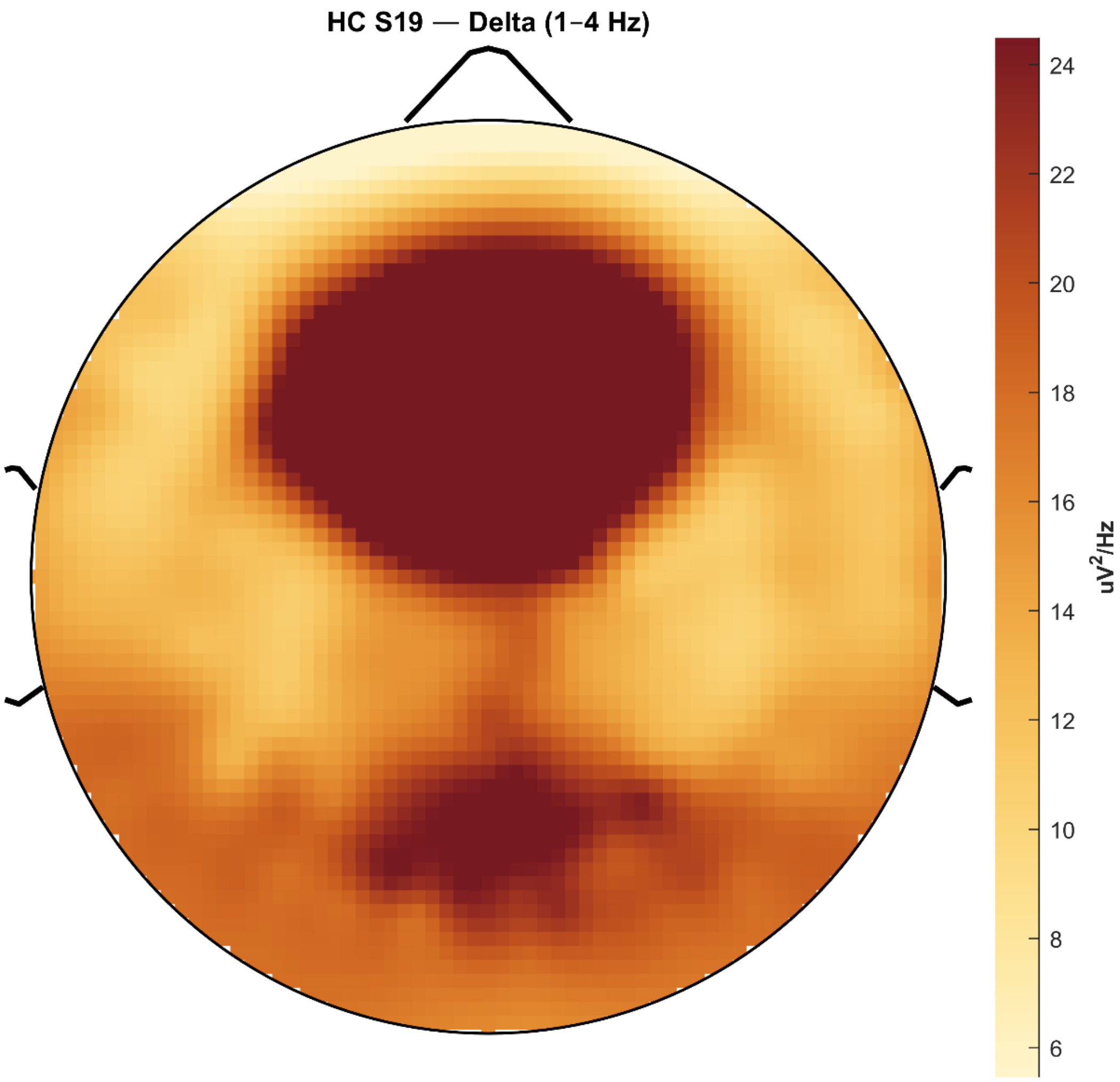

Supplementary Figure 98

Delta (1-4 Hz) power map for HC subject S19. The color scale represents delta power (μV²/Hz).

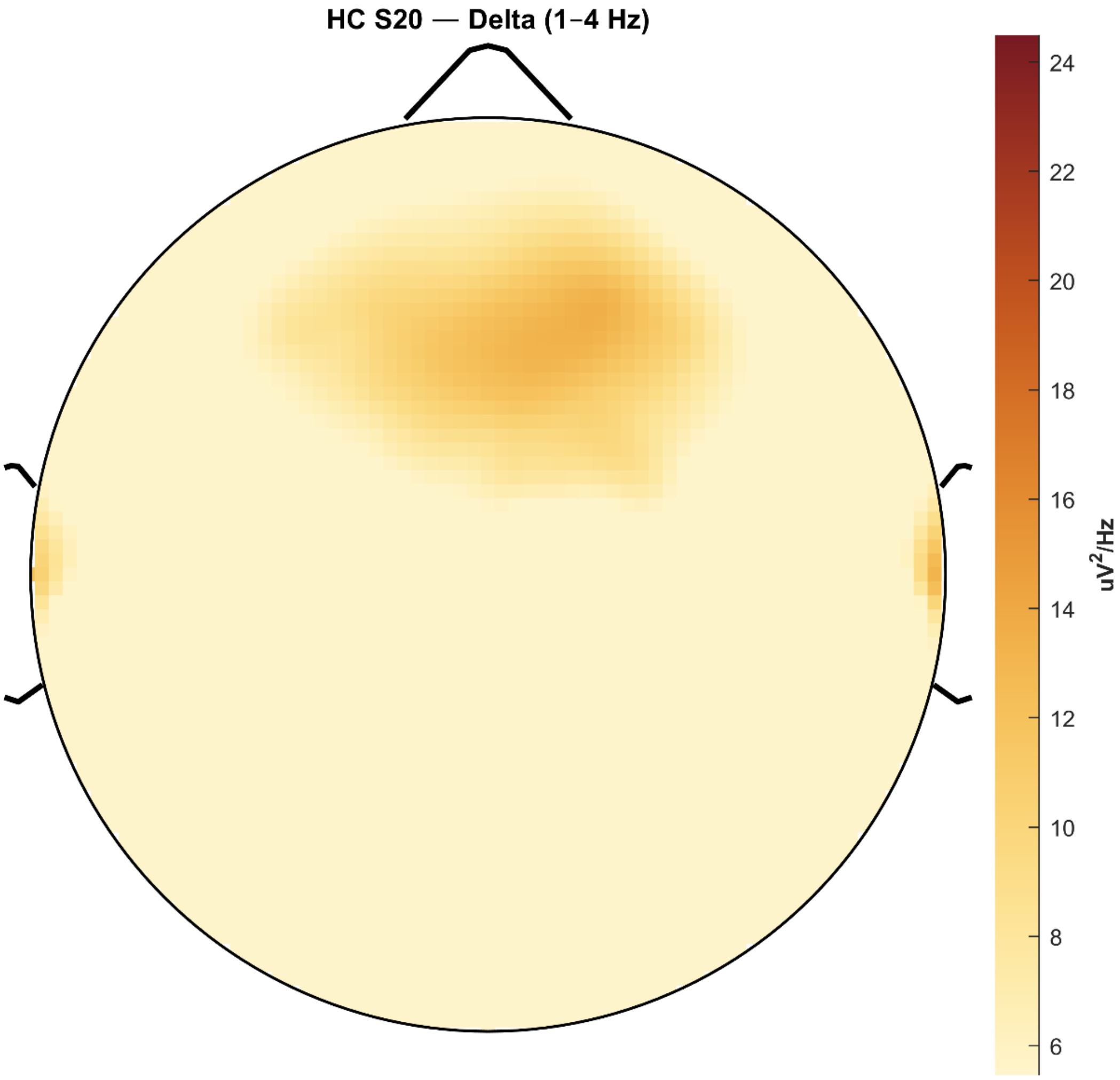

Supplementary Figure 99

Delta (1-4 Hz) power map for HC subject S20. The color scale represents delta power ($\mu V^2/Hz$).

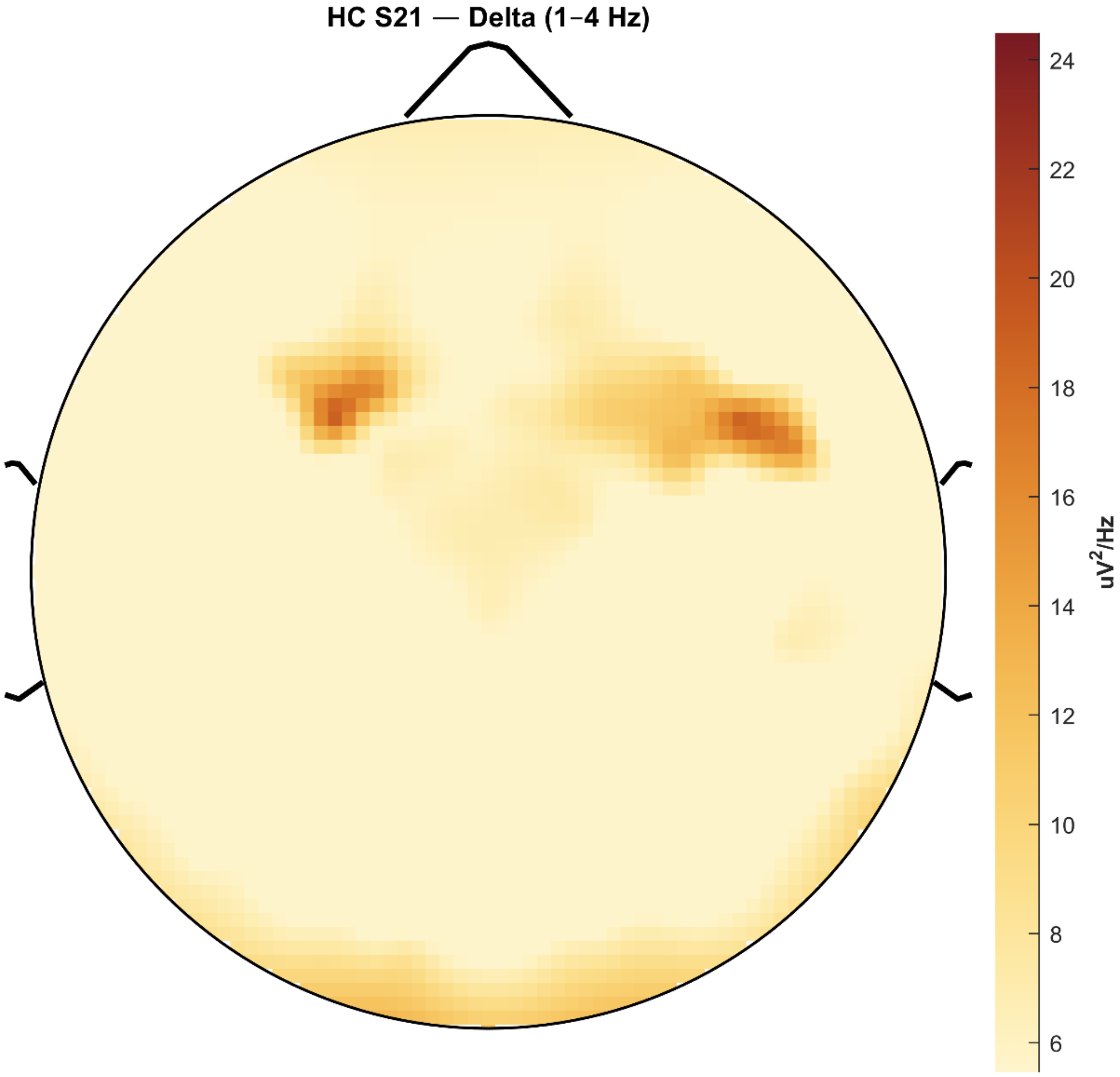

Supplementary Figure 100

Delta (1-4 Hz) power map for HC subject S21. The color scale represents delta power (μV²/Hz).

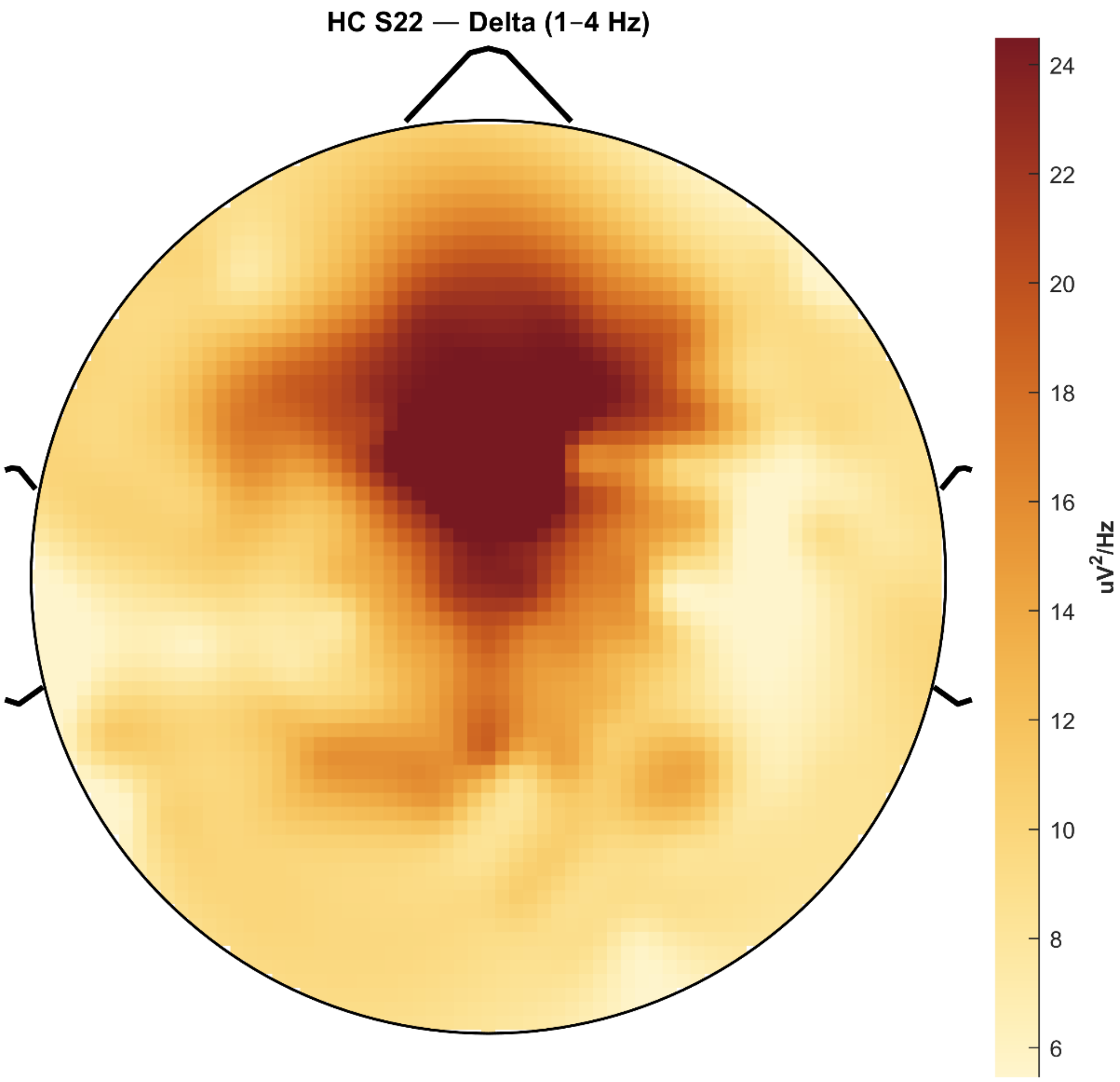

Supplementary Figure 101

Delta (1-4 Hz) power map for HC subject S22. The color scale represents delta power (µV²/Hz).

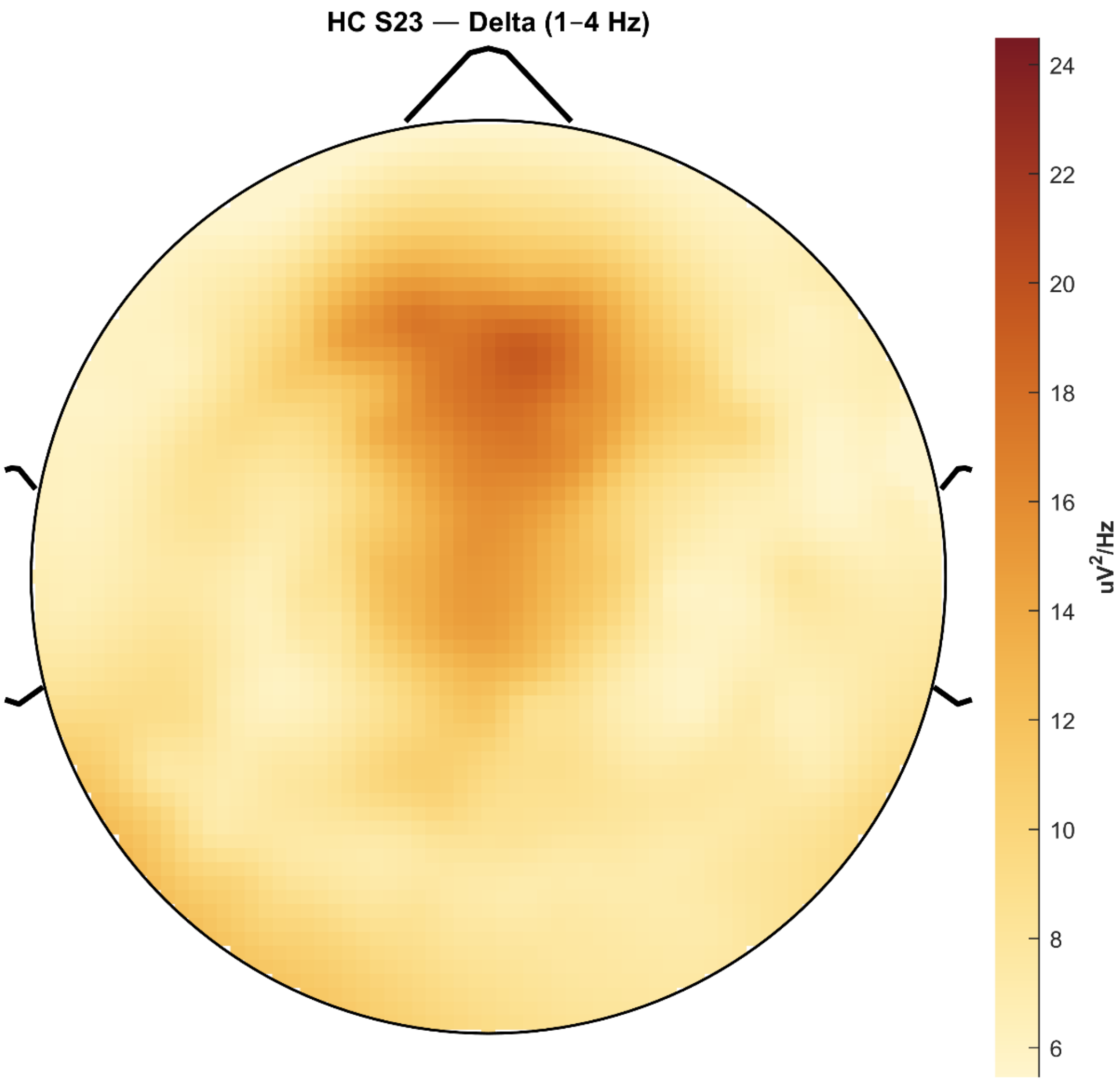

Supplementary Figure 102

Delta (1-4 Hz) power map for HC subject S23. The color scale represents delta power (μV²/Hz).

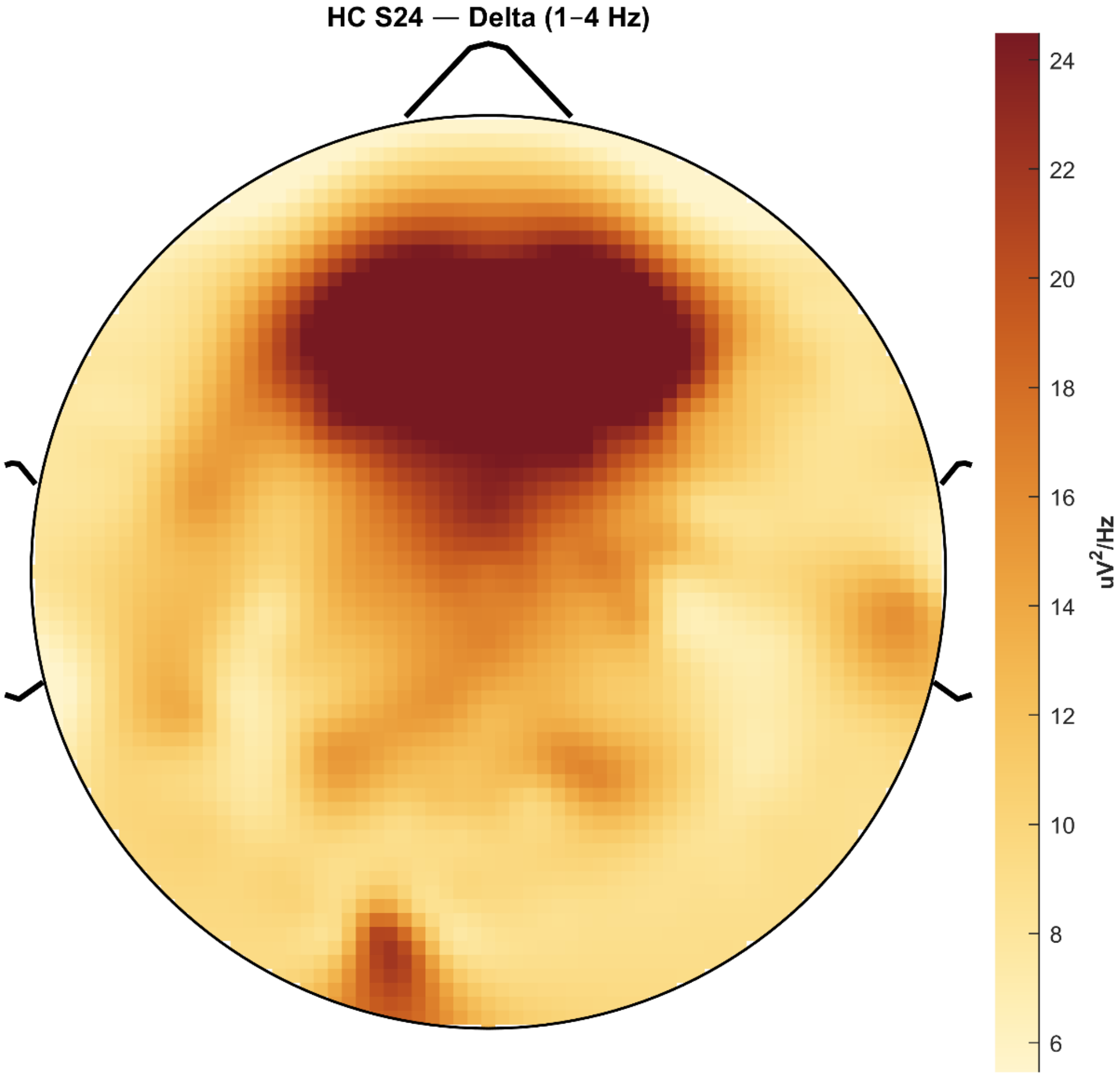

Supplementary Figure 103

Delta (1-4 Hz) power map for HC subject S24. The color scale represents delta power (μV²/Hz).

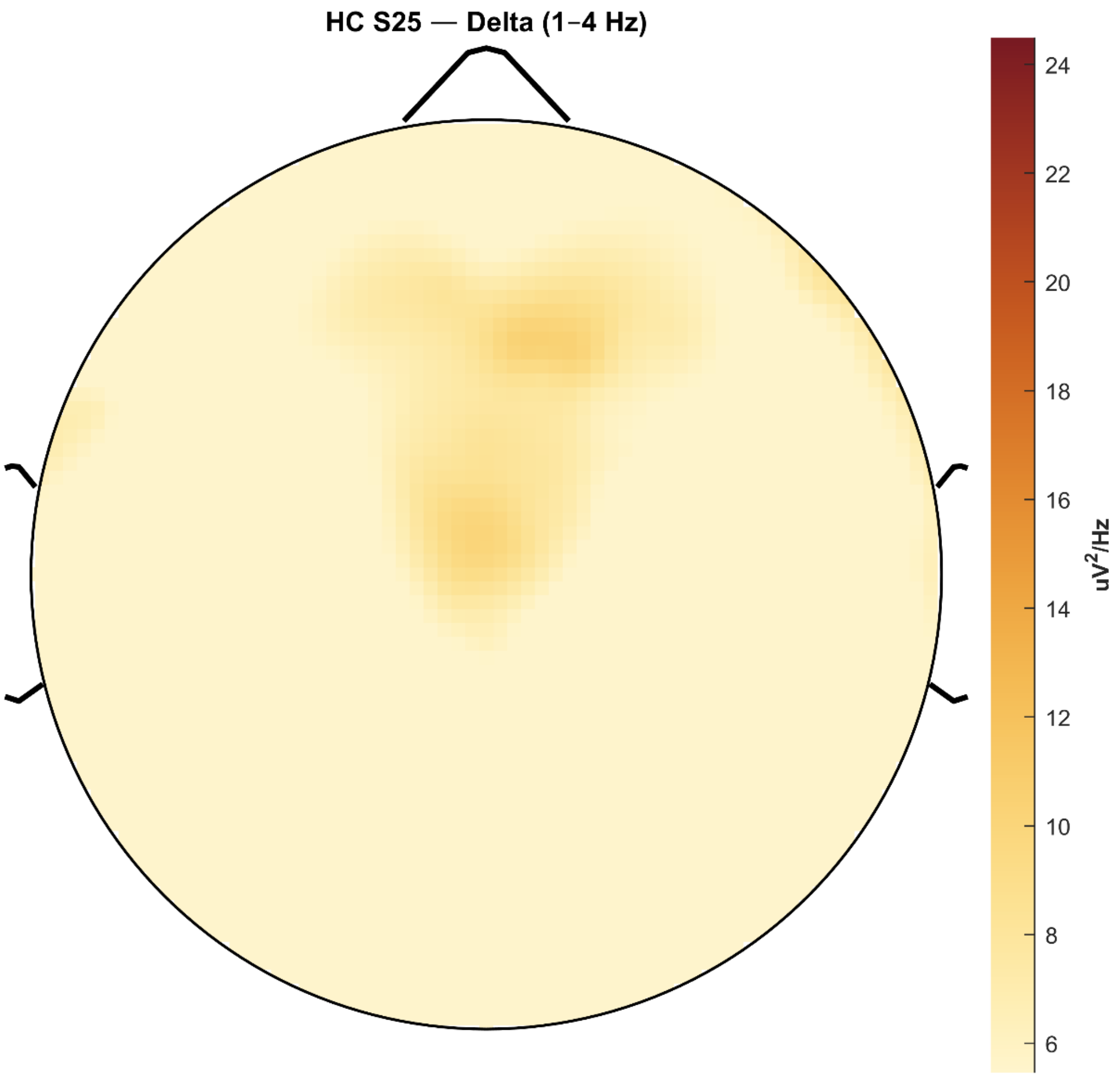

Supplementary Figure 104

Delta (1-4 Hz) power map for HC subject S25. The color scale represents delta power (µV²/Hz).

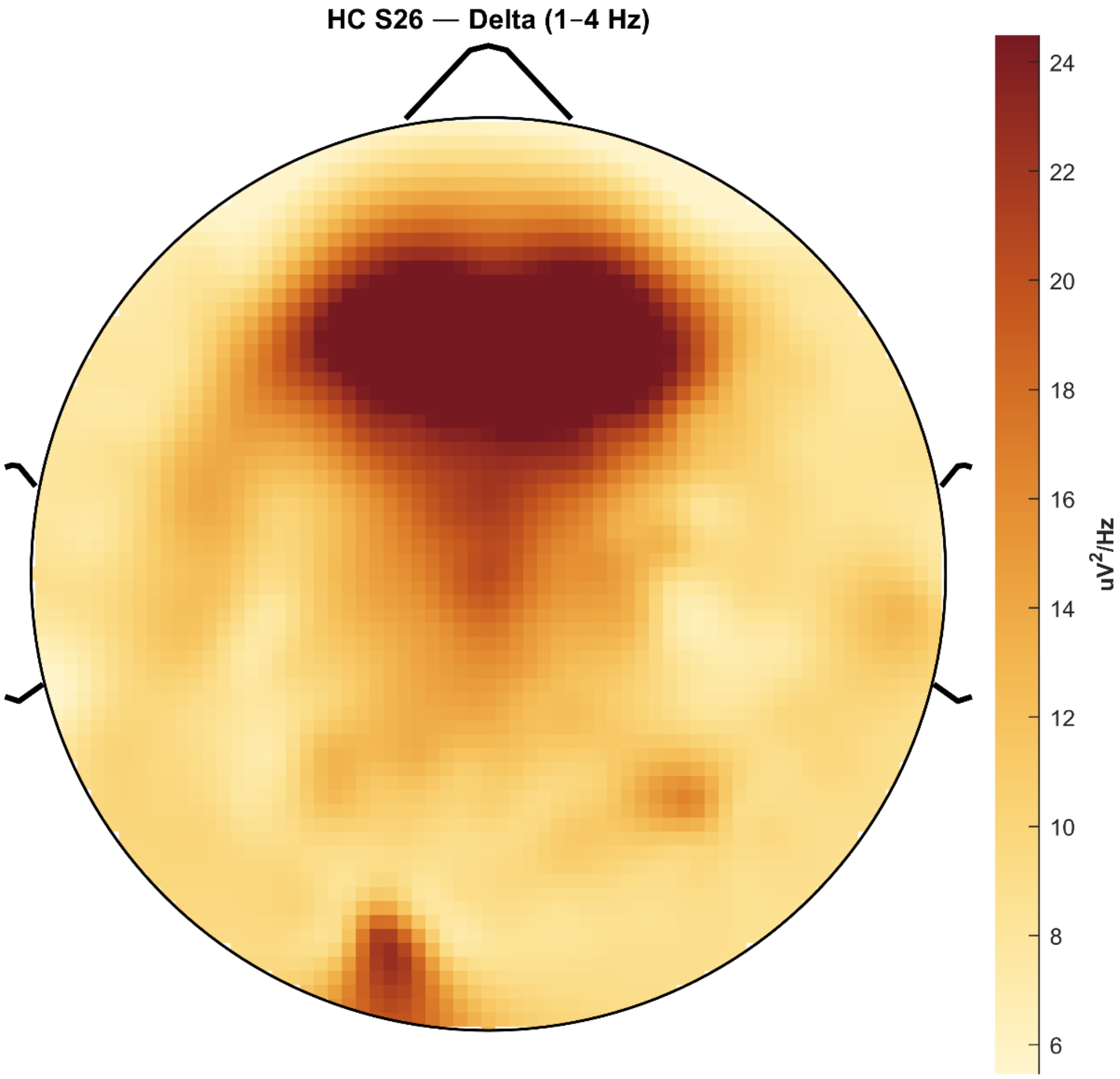

Supplementary Figure 105

Delta (1-4 Hz) power map for HC subject S26. The color scale represents delta power (μV²/Hz).

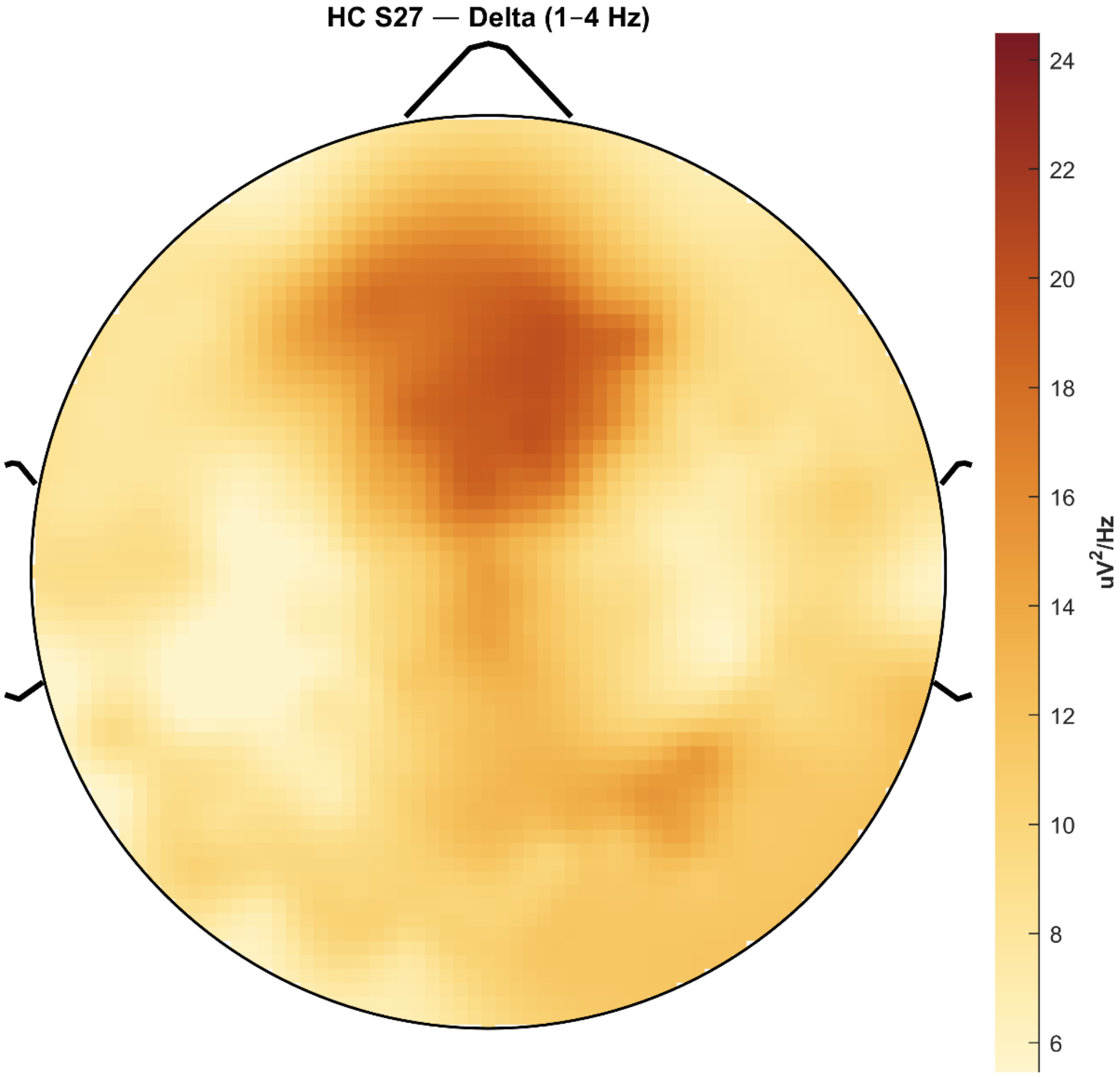

Supplementary Figure 106

Delta (1-4 Hz) power map for HC subject S27. The color scale represents delta power (μV²/Hz).

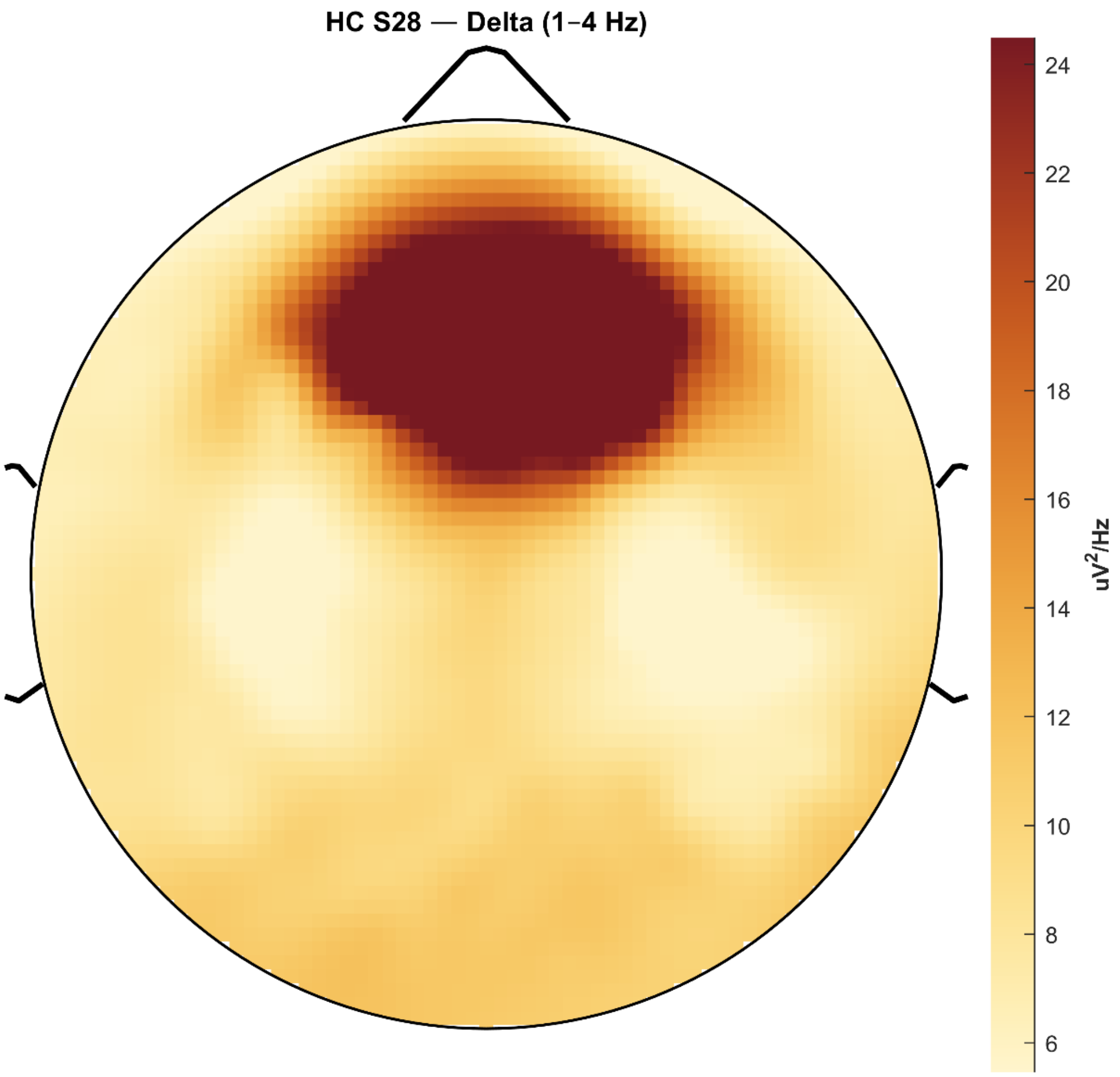

Supplementary Figure 107

Delta (1-4 Hz) power map for HC subject S28. The color scale represents delta power (μV²/Hz).

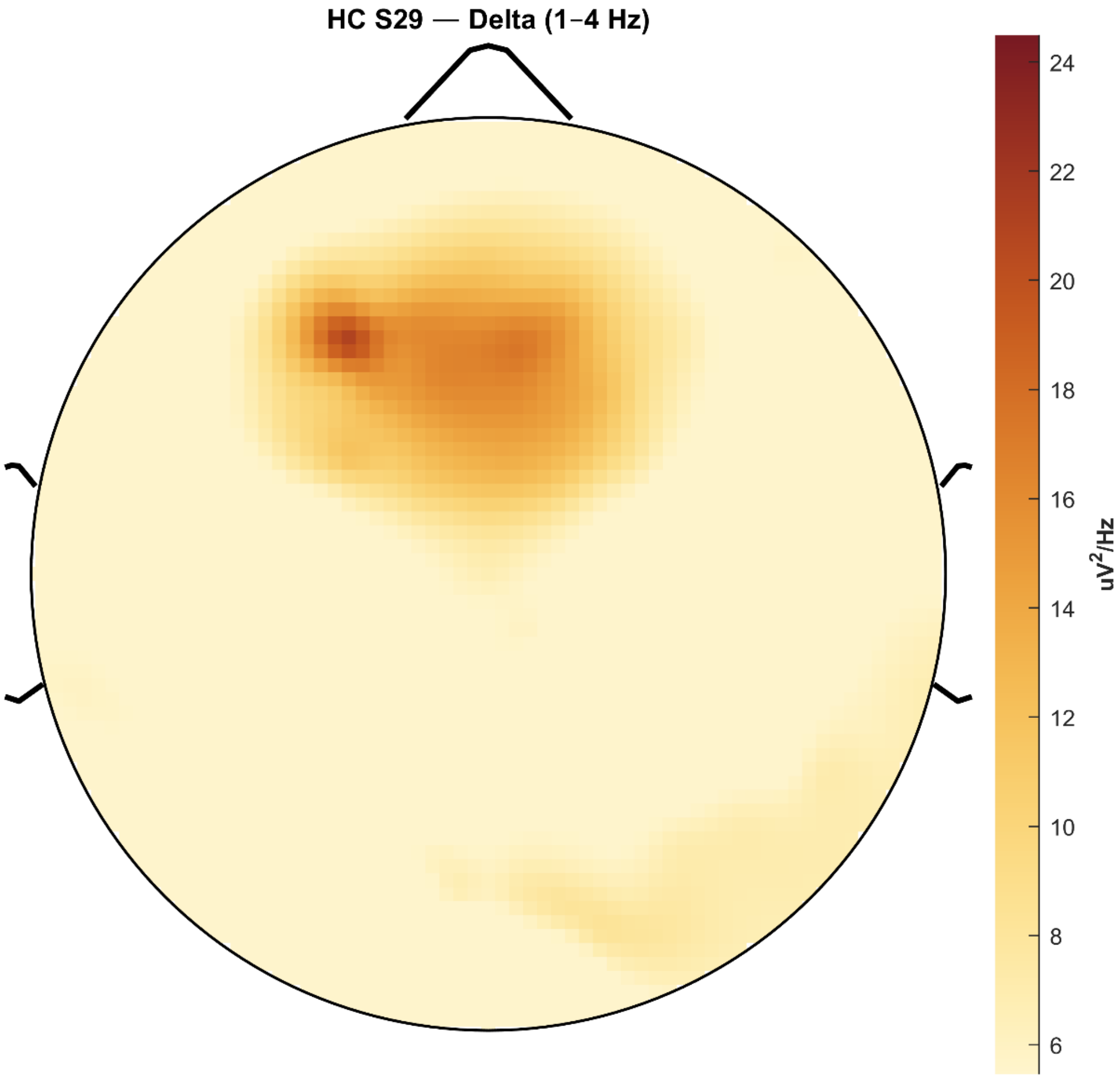

Supplementary Figure 108

Delta (1-4 Hz) power map for HC subject S29. The color scale represents delta power (μV²/Hz).

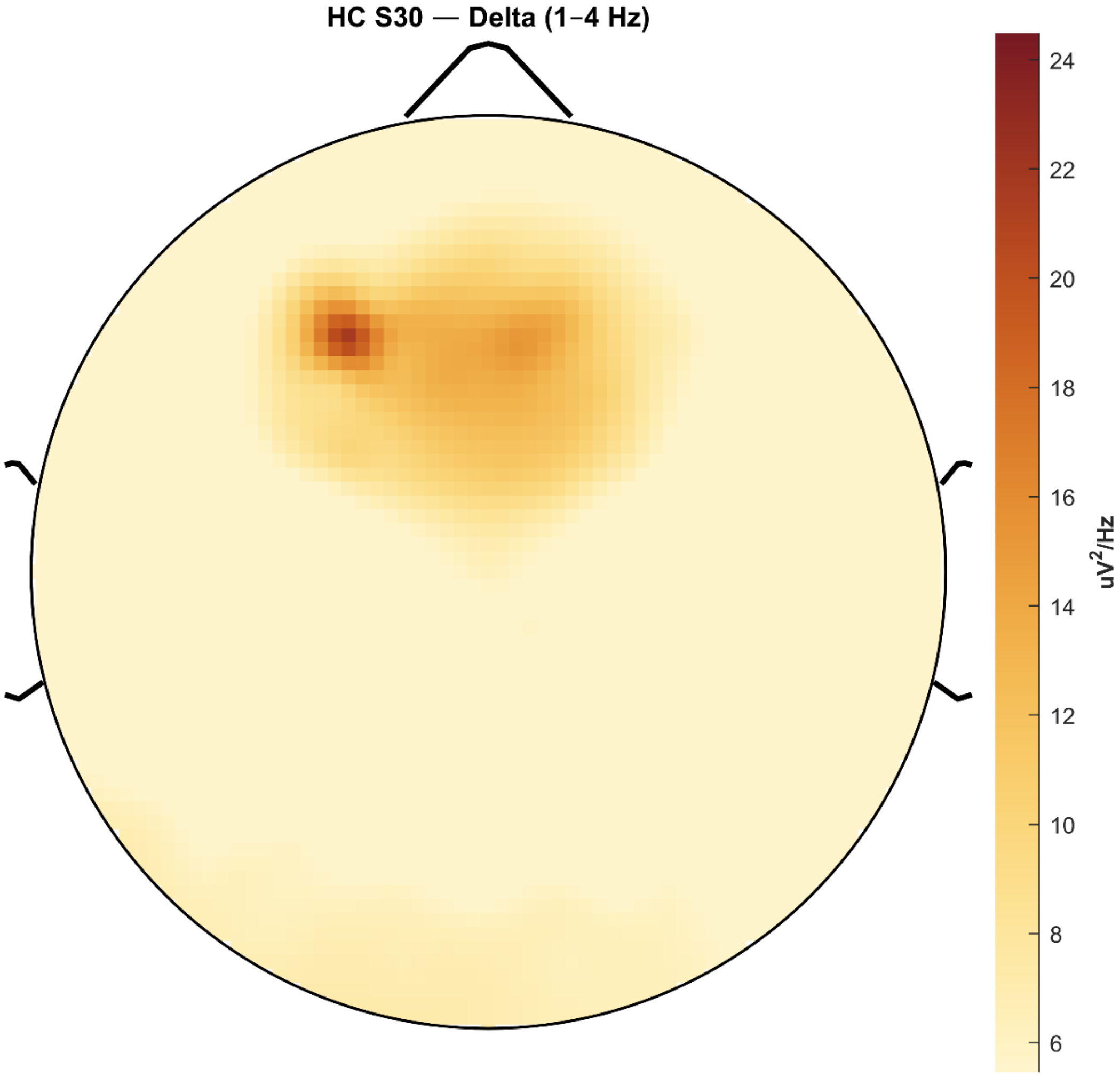

Supplementary Figure 109

Delta (1-4 Hz) power map for HC subject S30. The color scale represents delta power (μV²/Hz).

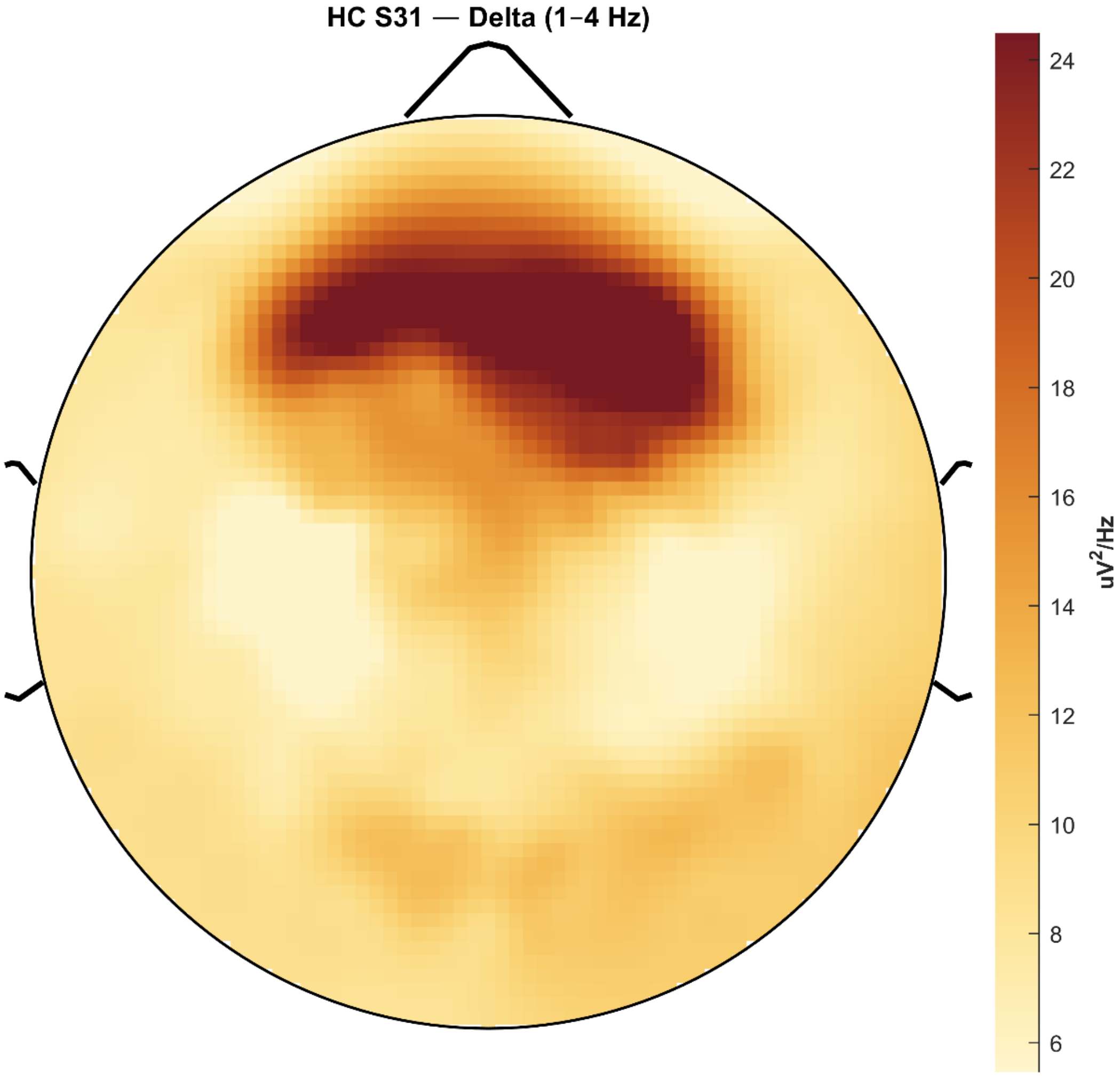

Supplementary Figure 110

Delta (1-4 Hz) power map for HC subject S31. The color scale represents delta power (μV²/Hz).

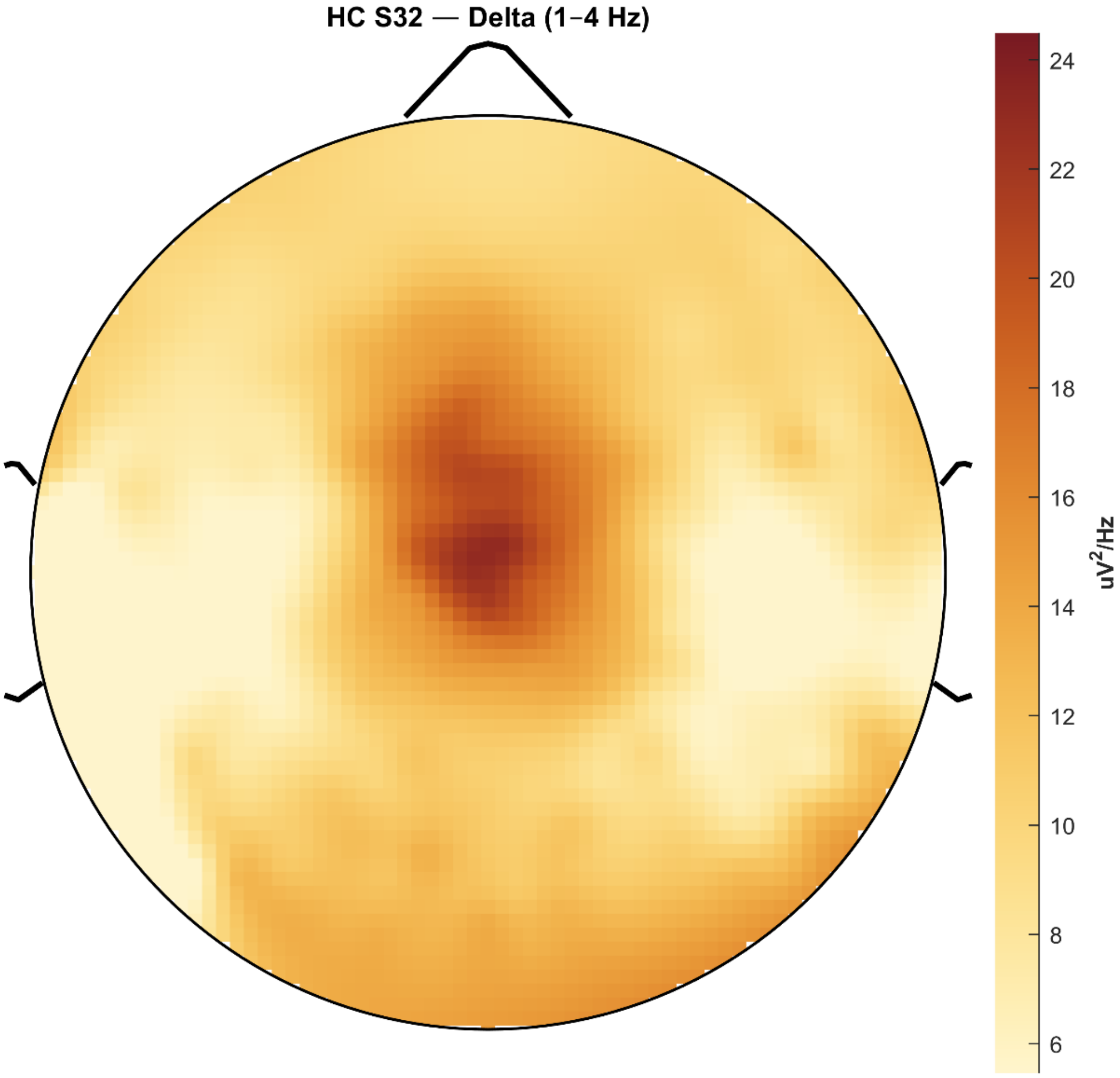

Supplementary Figure 111

Delta (1-4 Hz) power map for HC subject S32. The color scale represents delta power (μV²/Hz).

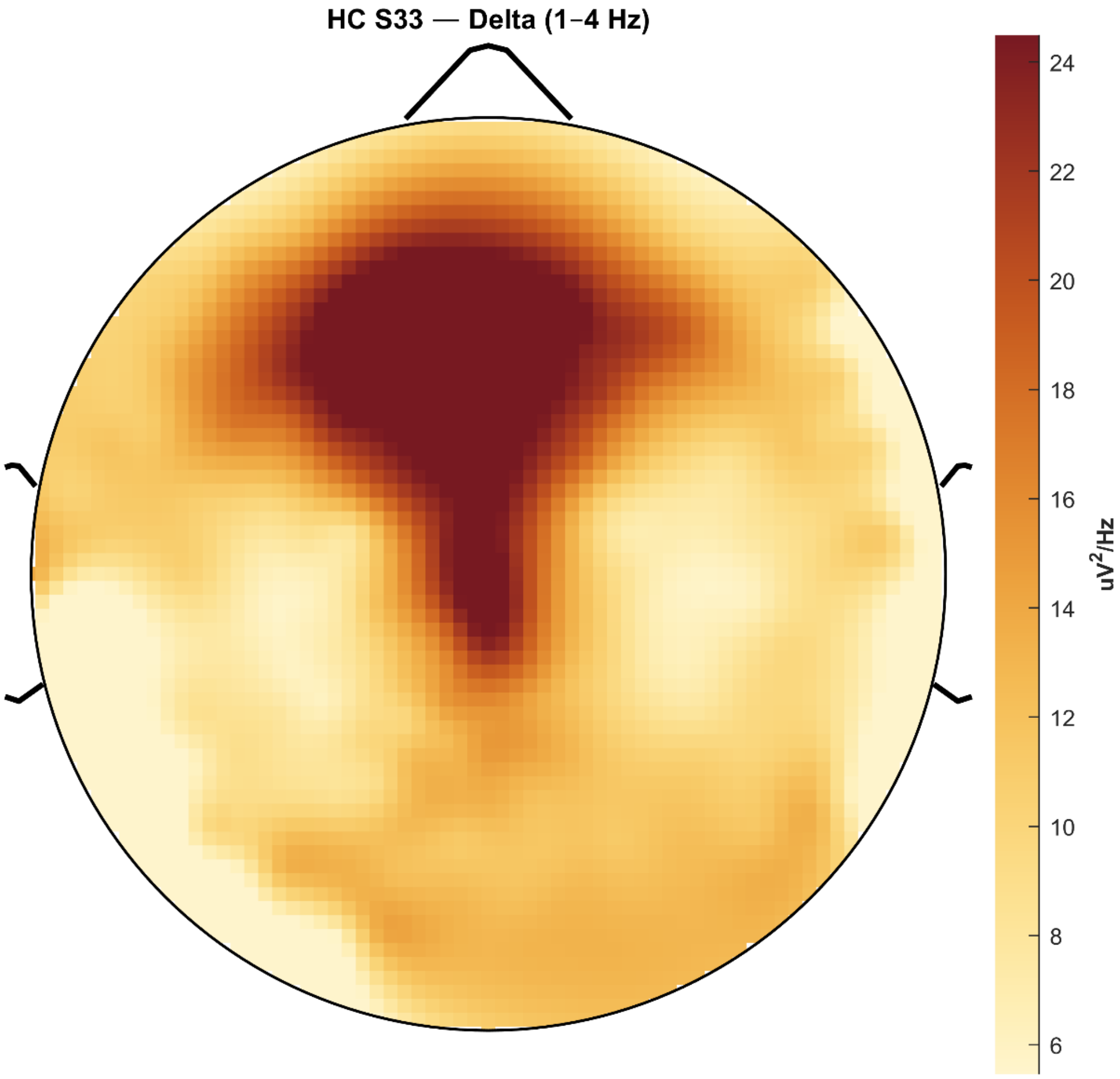

Supplementary Figure 112

Delta (1-4 Hz) power map for HC subject S33. The color scale represents delta power (µV²/Hz).

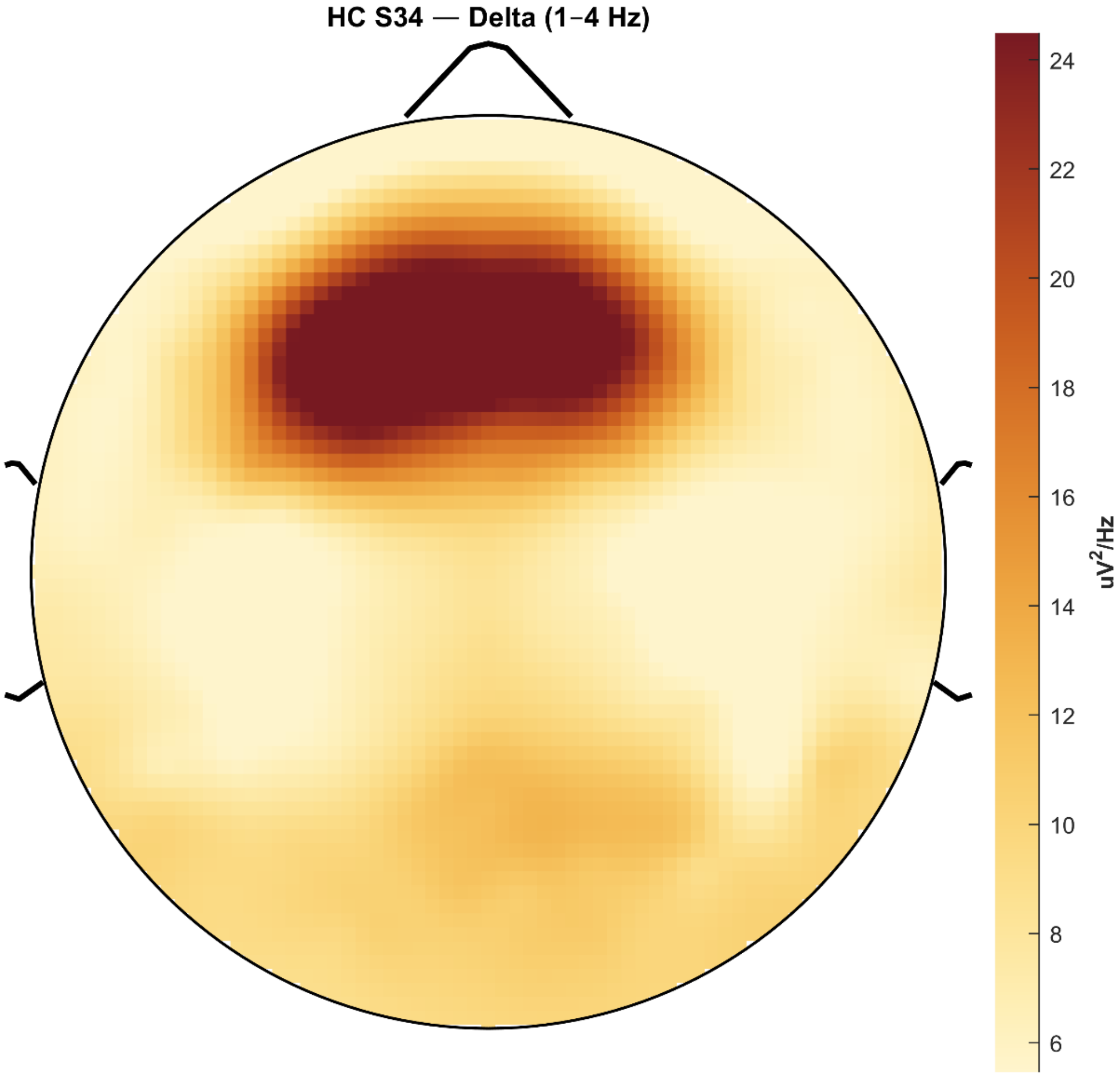

Supplementary Figure 113

Delta (1-4 Hz) power map for HC subject S34. The color scale represents delta power (μV²/Hz).

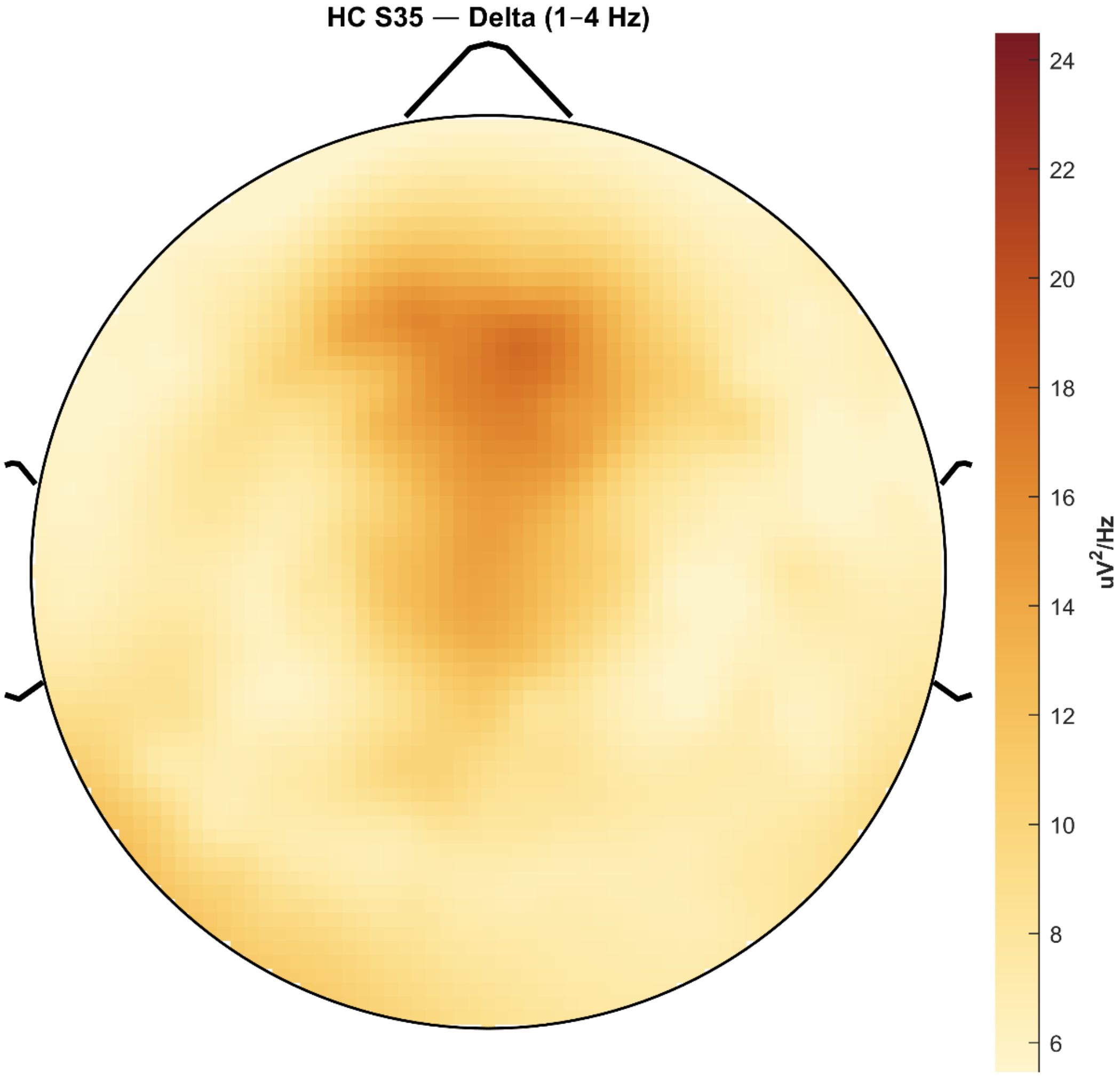

Supplementary Figure 114

Delta (1-4 Hz) power map for HC subject S35. The color scale represents delta power (μV²/Hz).

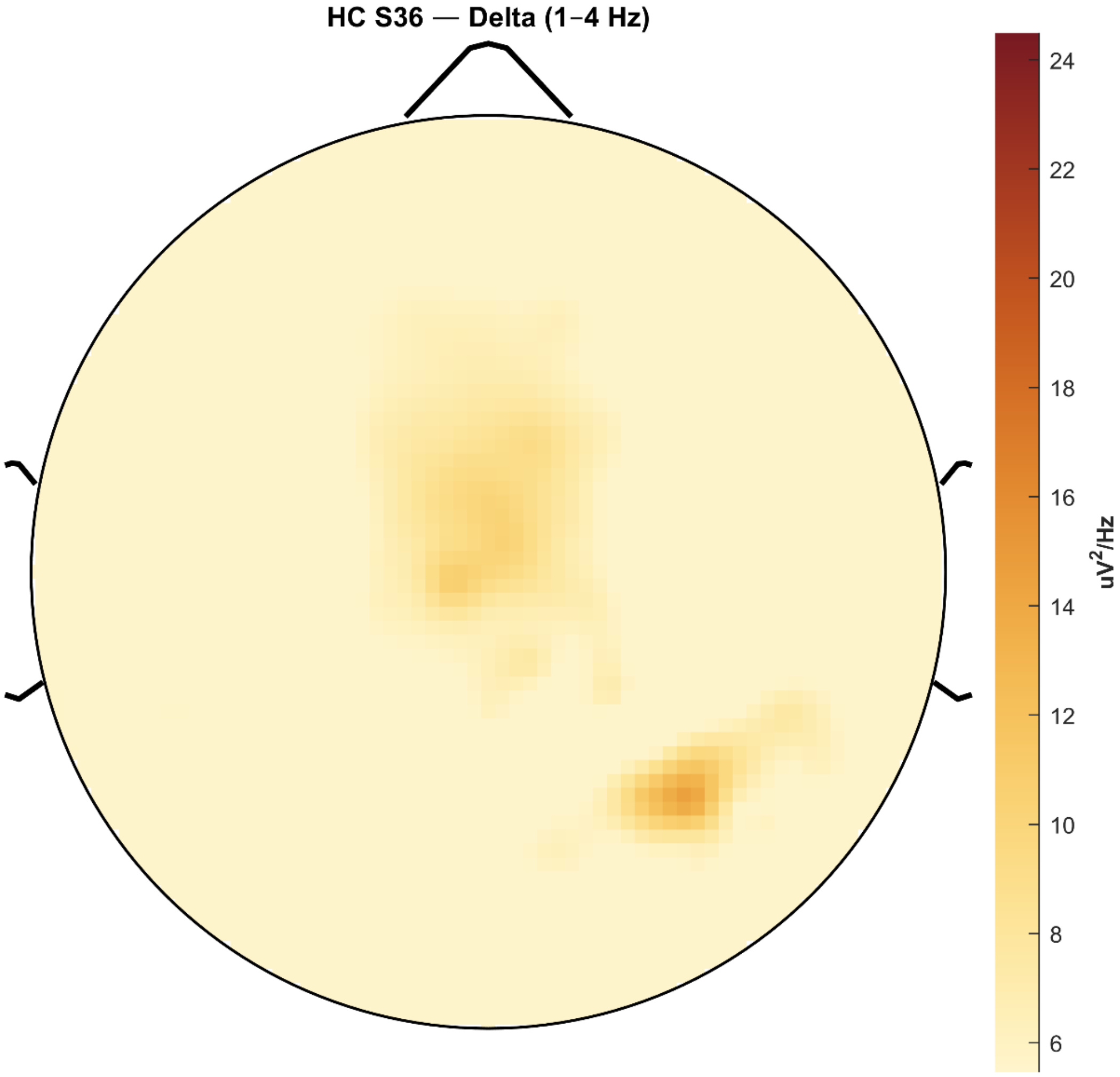

Supplementary Figure 115

Delta (1-4 Hz) power map for HC subject S36. The color scale represents delta power (μV²/Hz).

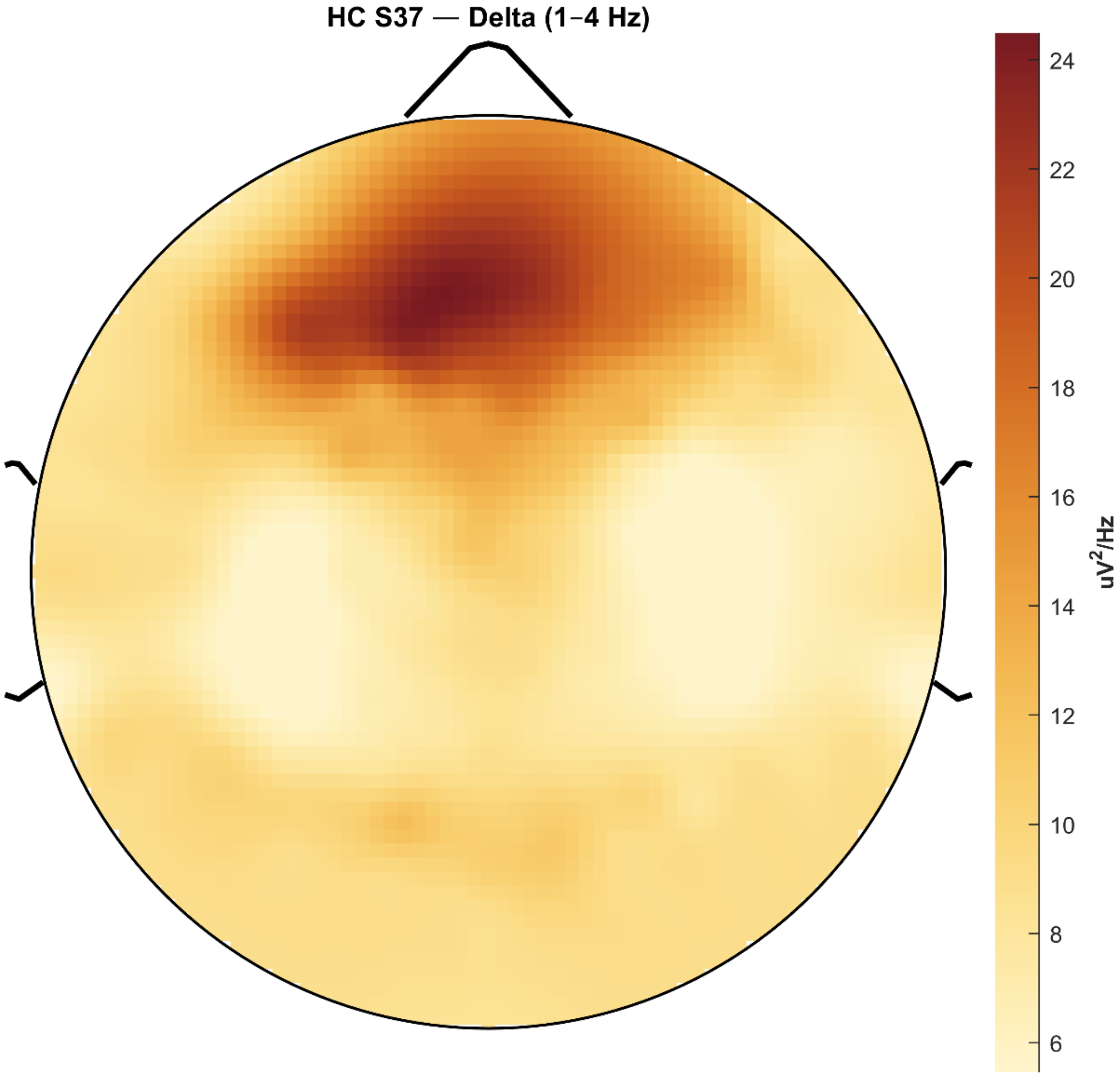

Supplementary Figure 116

Delta (1-4 Hz) power map for HC subject S37. The color scale represents delta power (μV²/Hz).

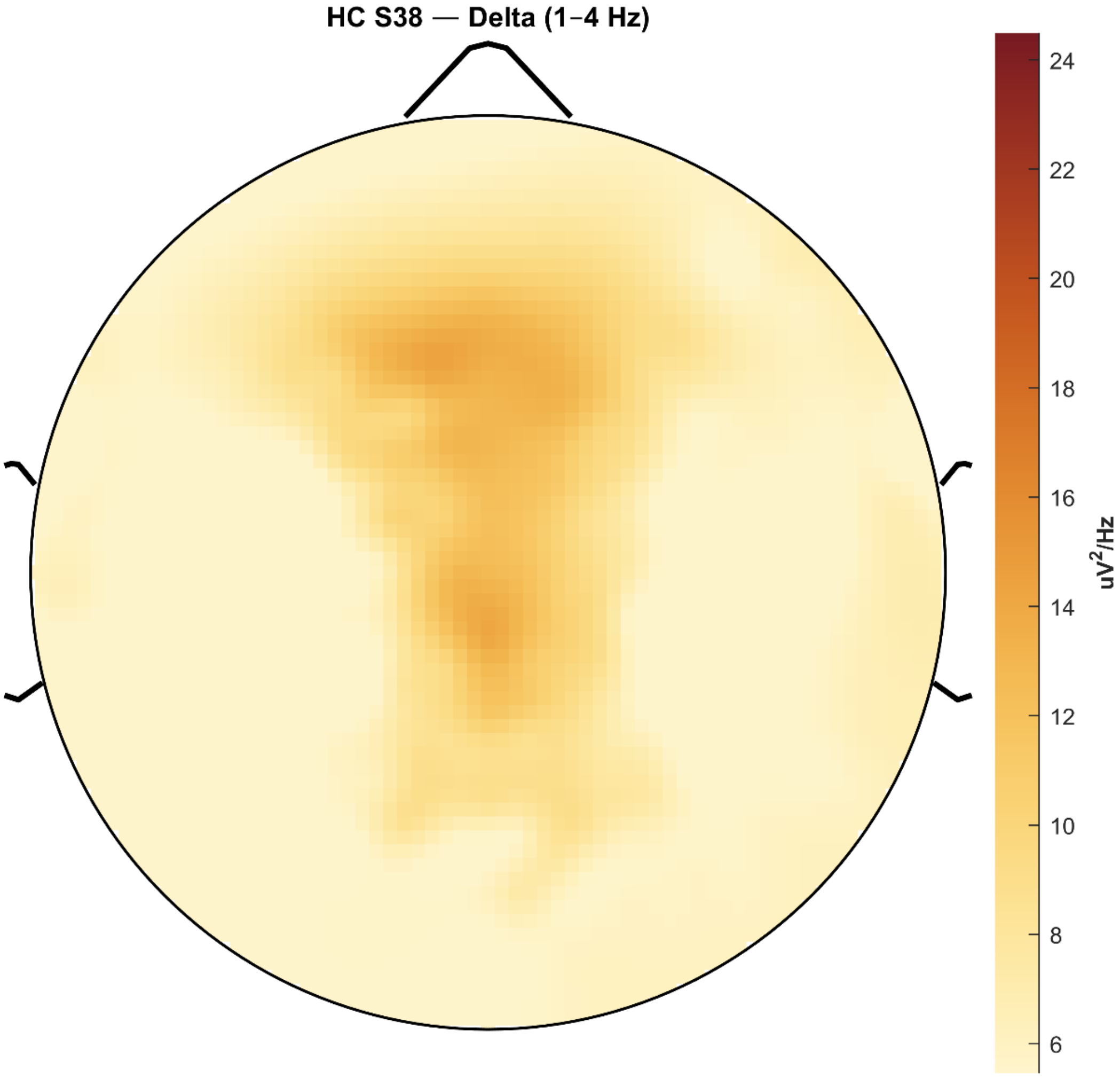

Supplementary Figure 117

Delta (1-4 Hz) power map for HC subject S38. The color scale represents delta power (μV²/Hz).

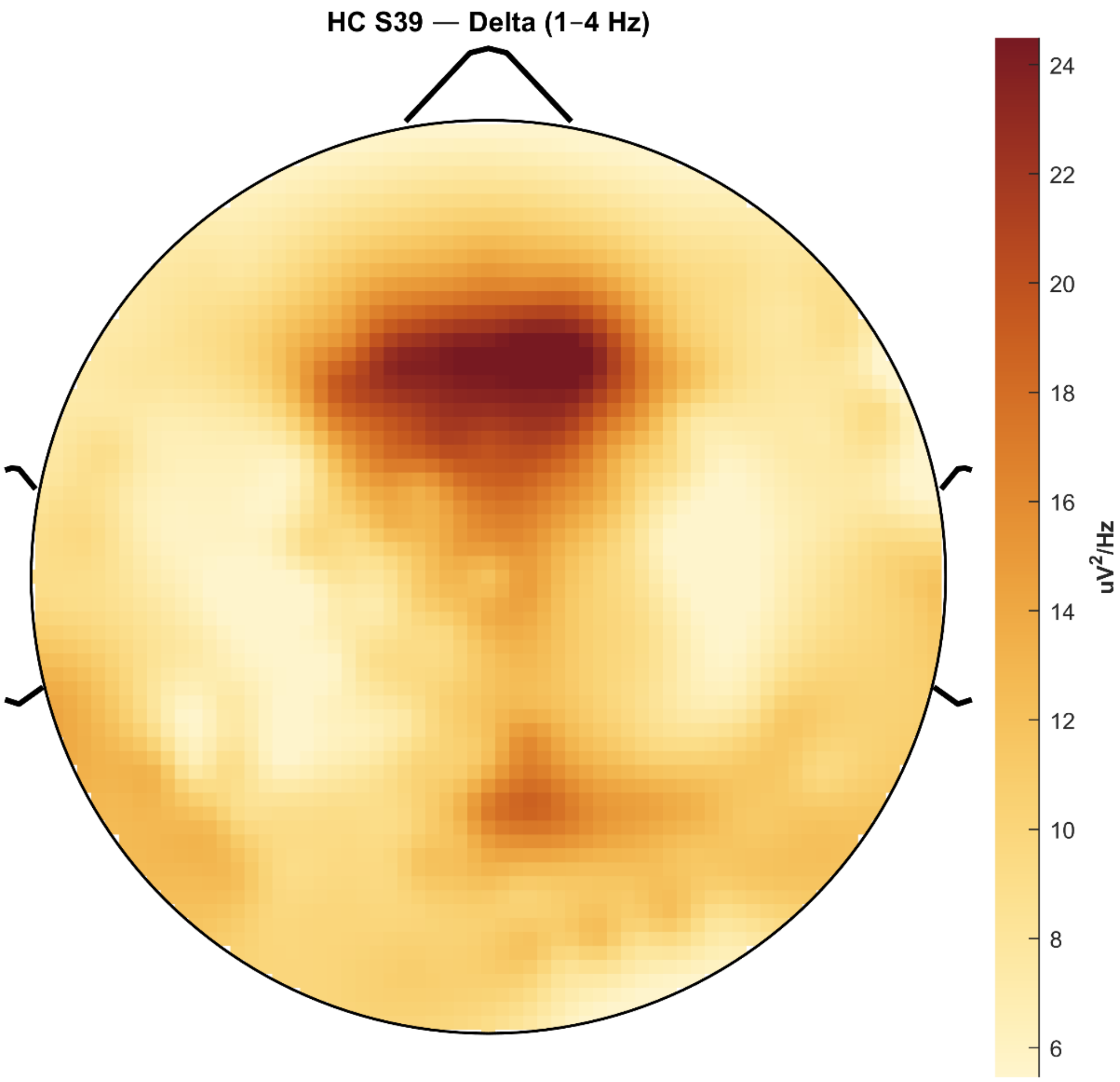

Supplementary Figure 118

Delta (1-4 Hz) power map for HC subject S39. The color scale represents delta power (μV²/Hz).

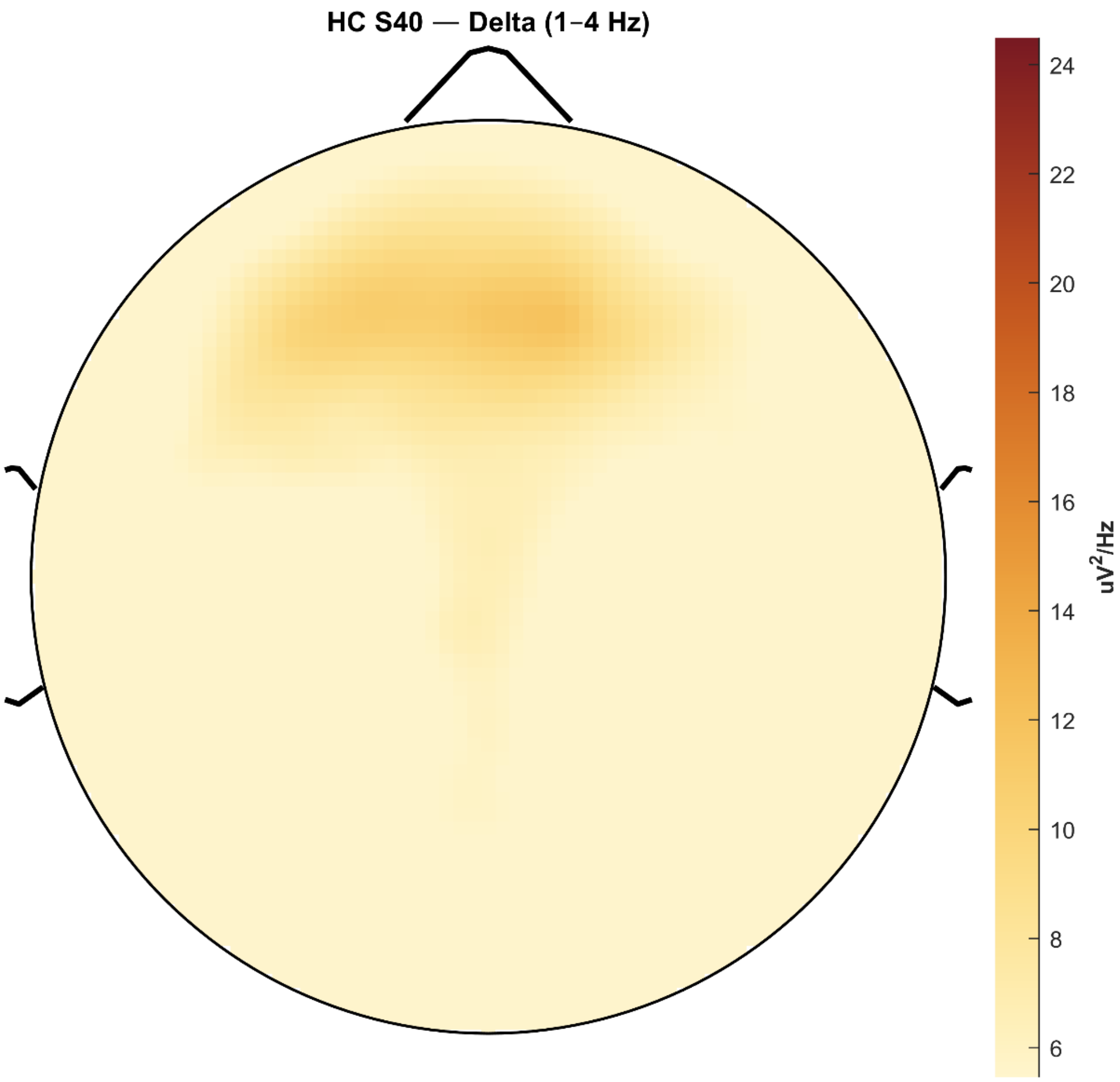

Supplementary Figure 119

Delta (1-4 Hz) power map for HC subject S40. The color scale represents delta power (μV²/Hz).

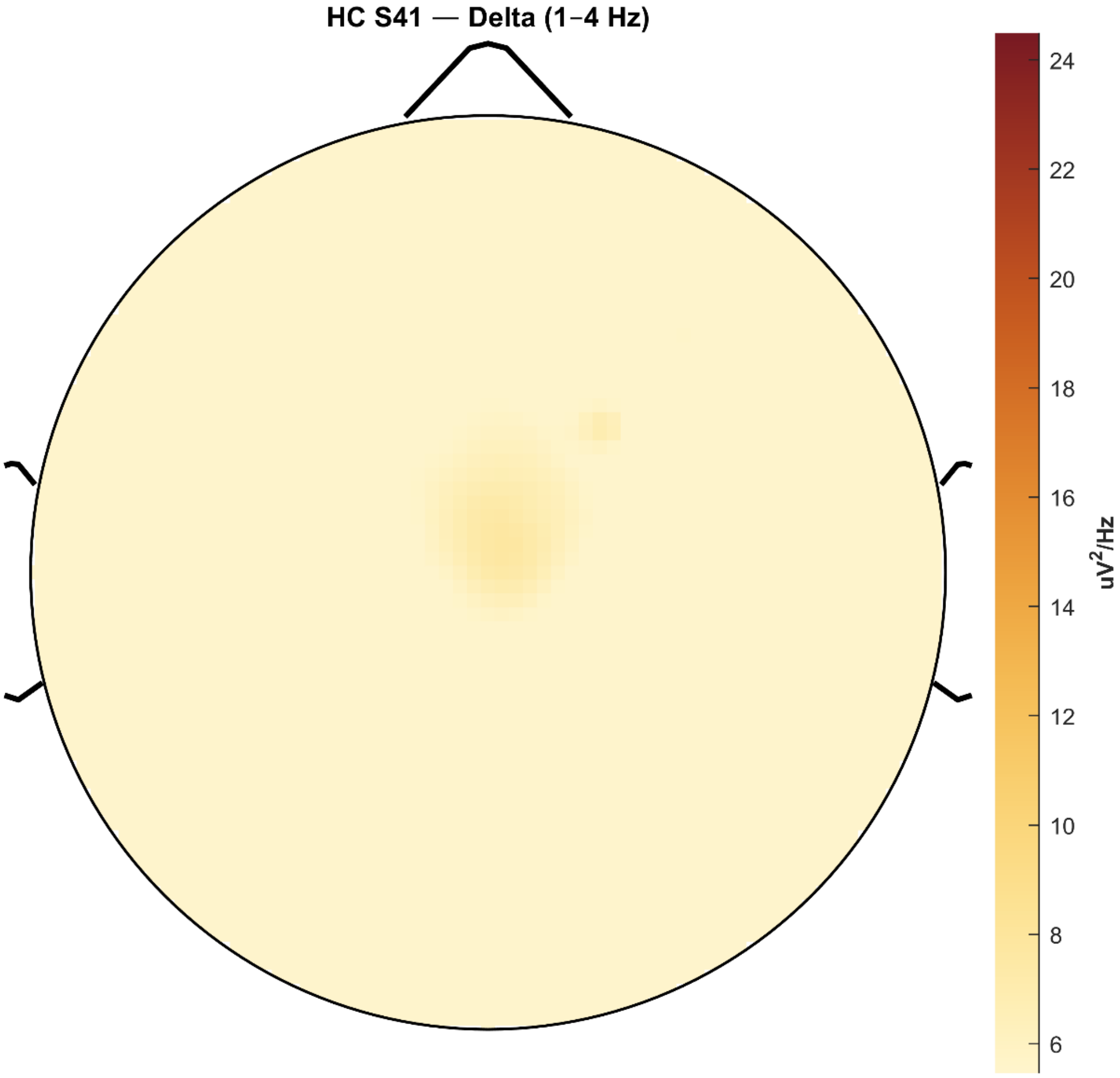

Supplementary Figure 120

Delta (1-4 Hz) power map for HC subject S41. The color scale represents delta power (μV²/Hz).

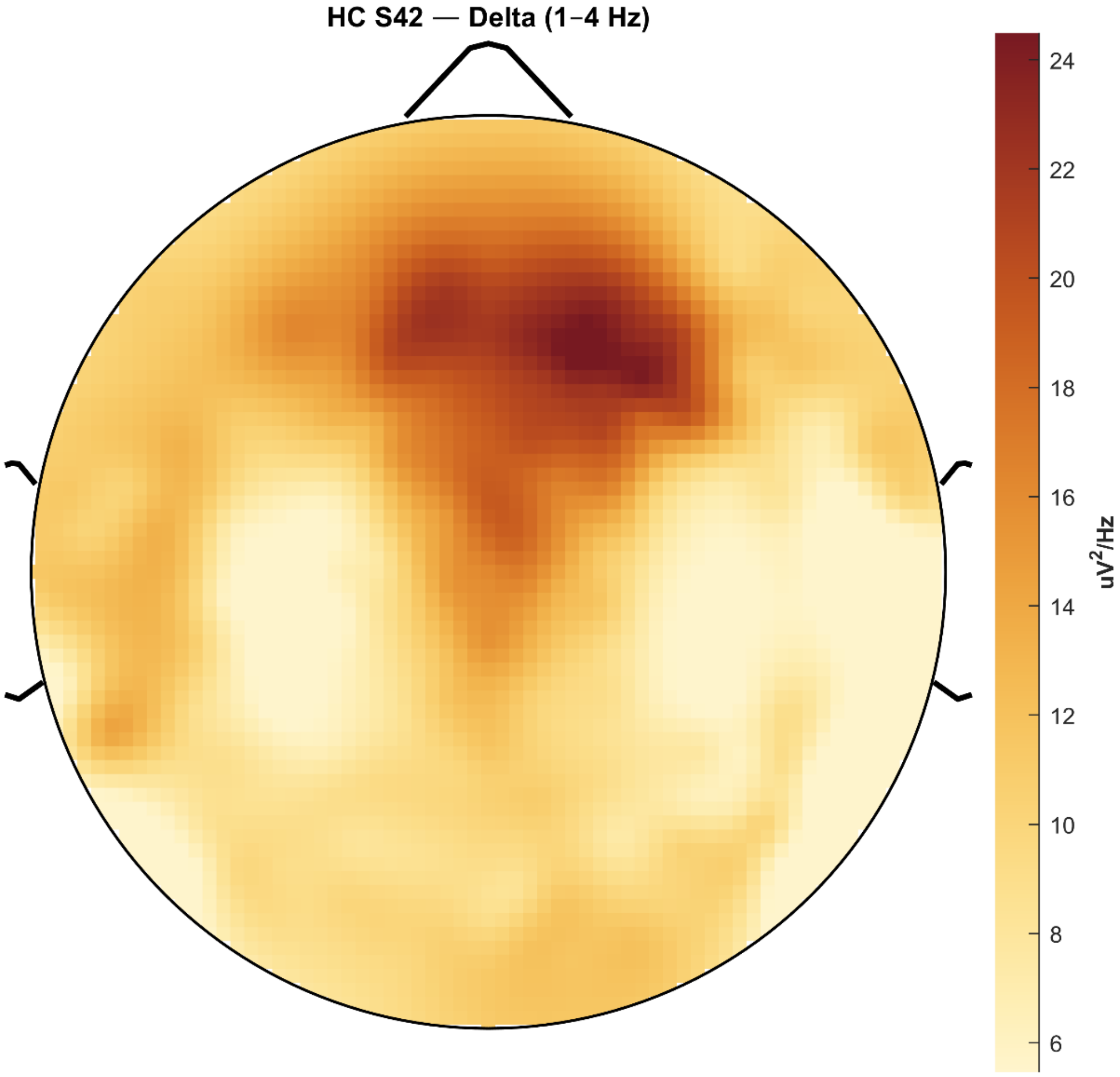

Supplementary Figure 121

Delta (1-4 Hz) power map for HC subject S42. The color scale represents delta power (μV²/Hz).

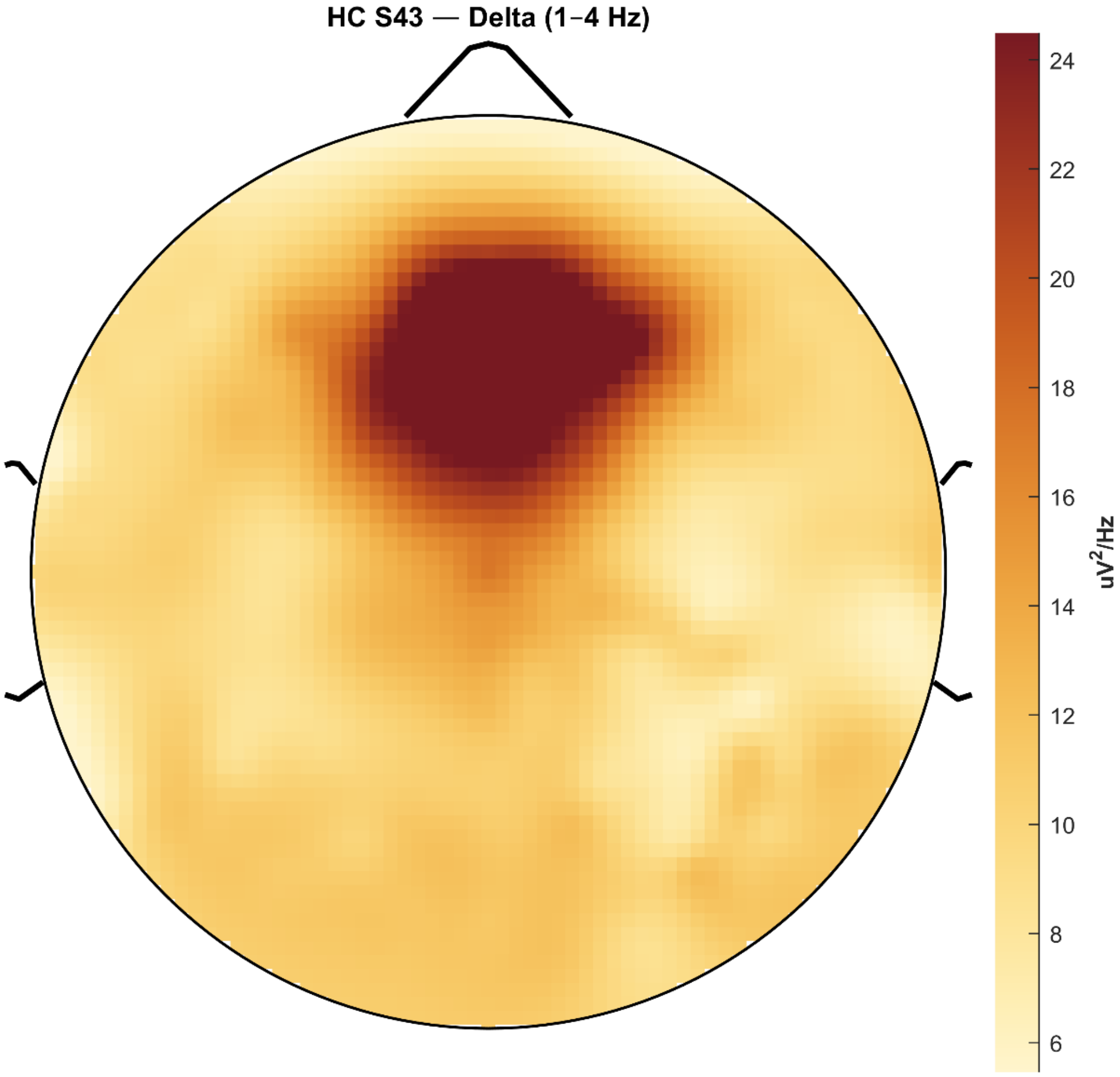

Supplementary Figure 122

Delta (1-4 Hz) power map for HC subject S43. The color scale represents delta power (μV²/Hz).

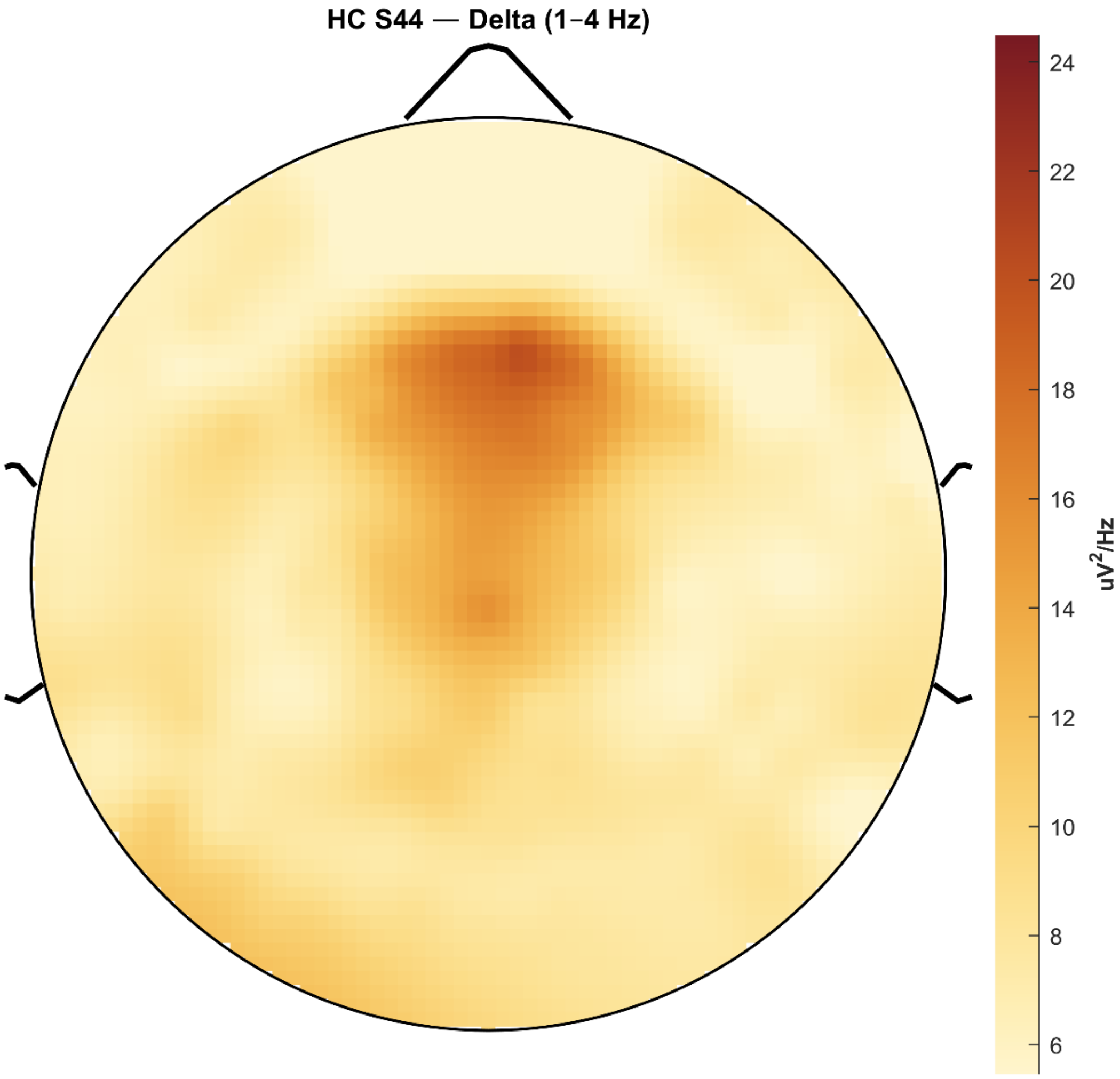

Supplementary Figure 123

Delta (1-4 Hz) power map for HC subject S44. The color scale represents delta power (μV²/Hz).

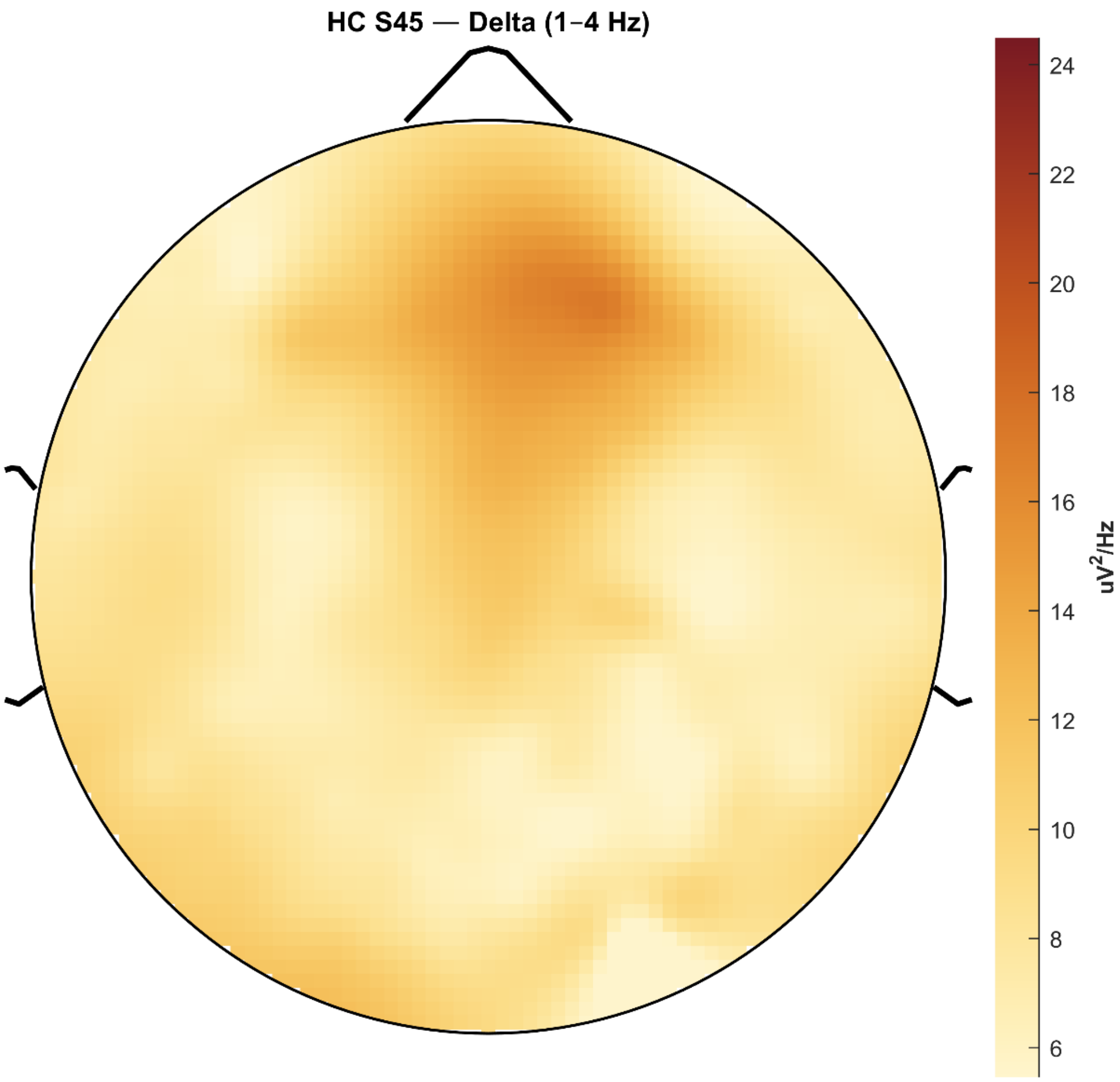

Supplementary Figure 124

Delta (1-4 Hz) power map for HC subject S45. The color scale represents delta power (μV²/Hz).

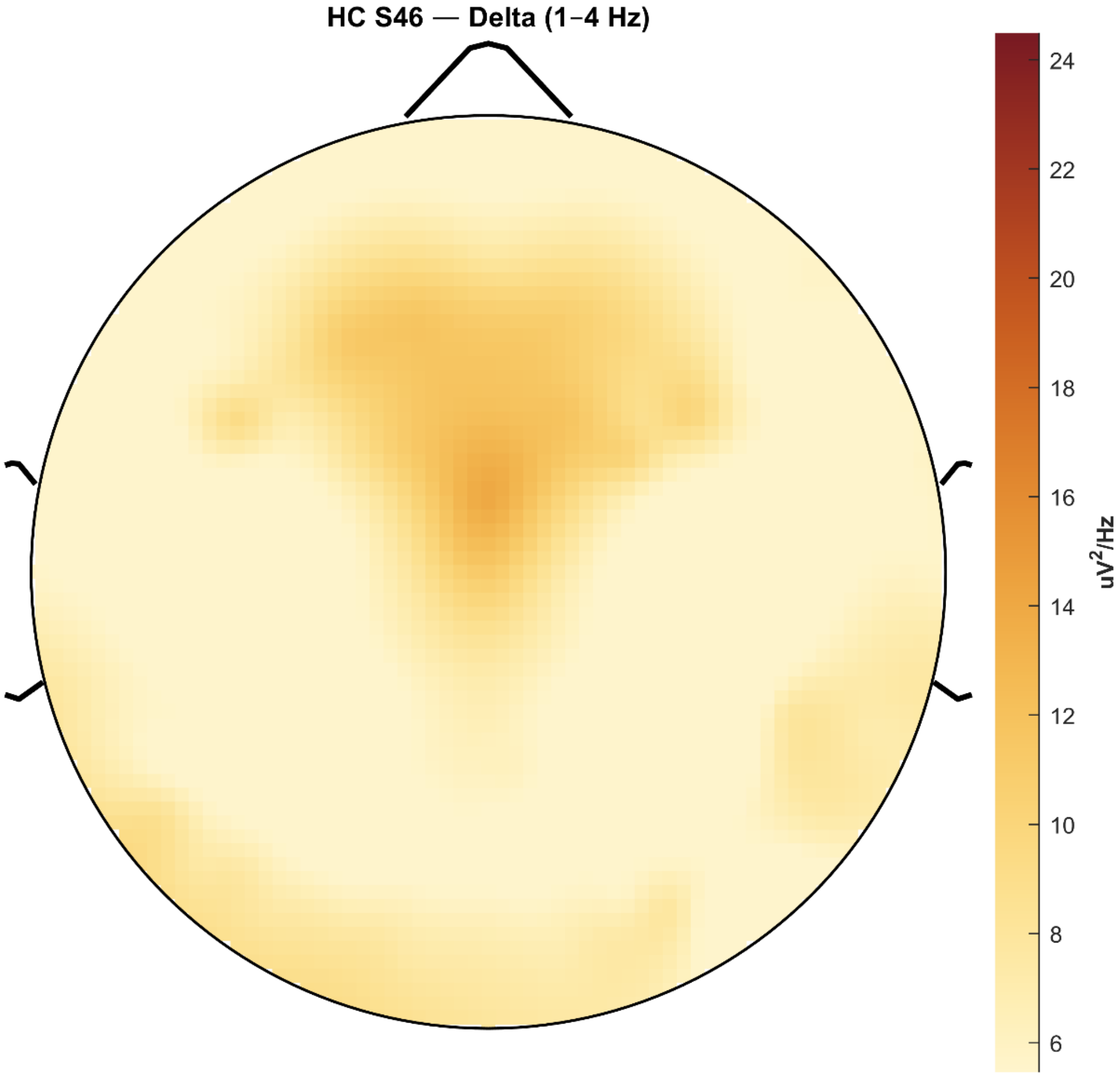

Supplementary Figure 125

Delta (1-4 Hz) power map for HC subject S46. The color scale represents delta power (μV²/Hz).

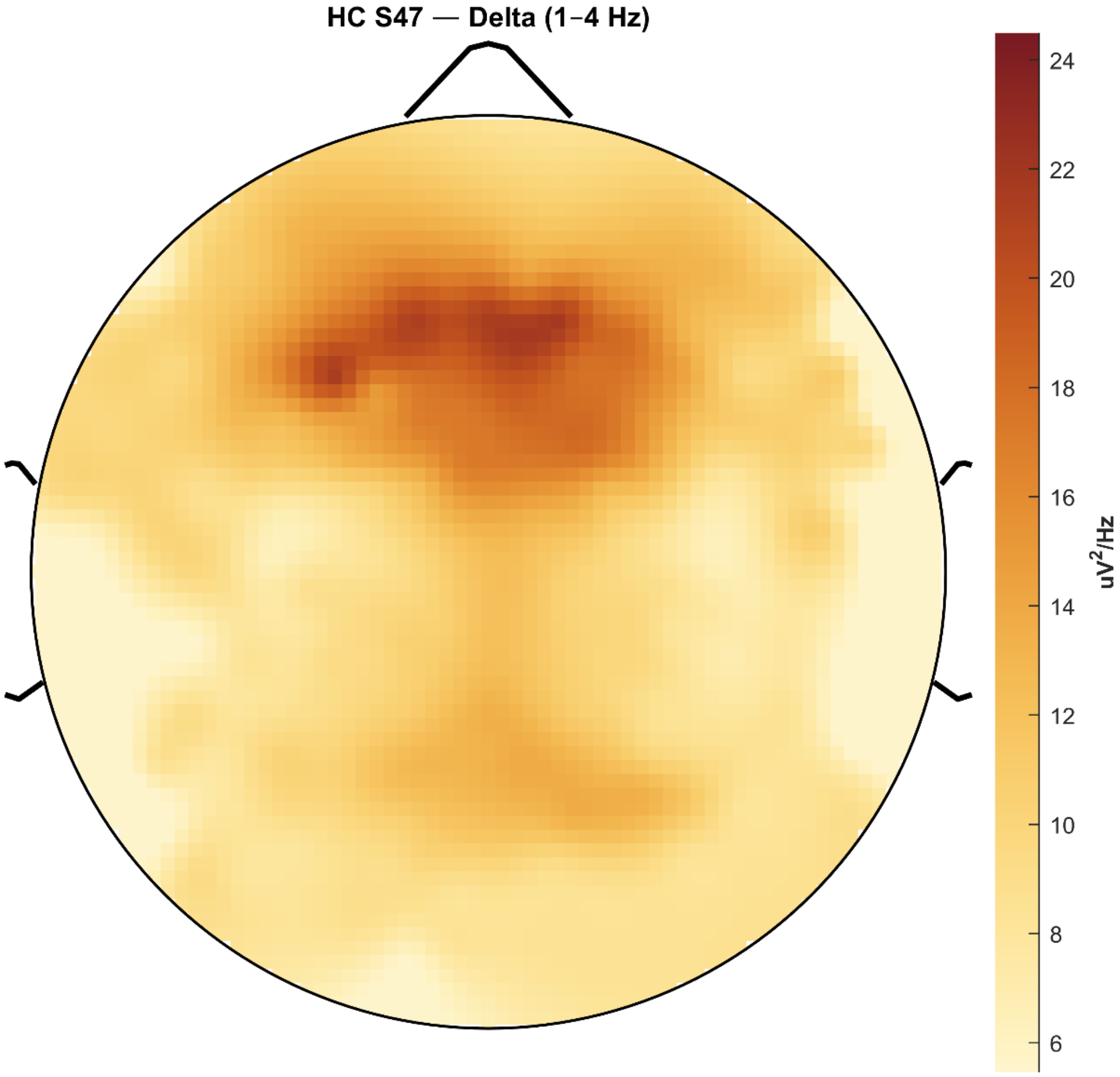

Supplementary Figure 126

Delta (1-4 Hz) power map for HC subject S47. The color scale represents delta power (µV²/Hz).

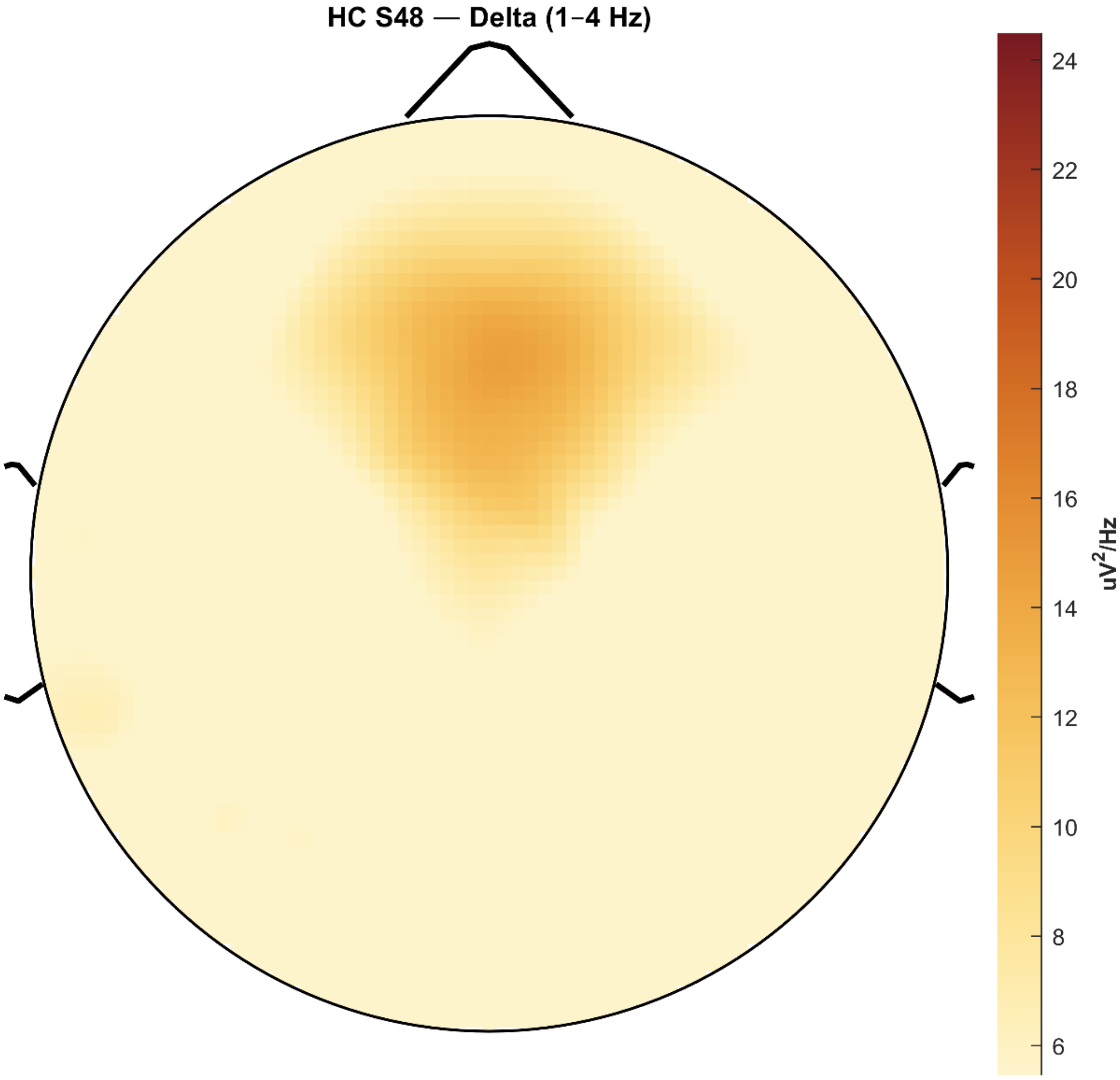

Supplementary Figure 127

Delta (1-4 Hz) power map for HC subject S48. The color scale represents delta power (µV²/Hz).

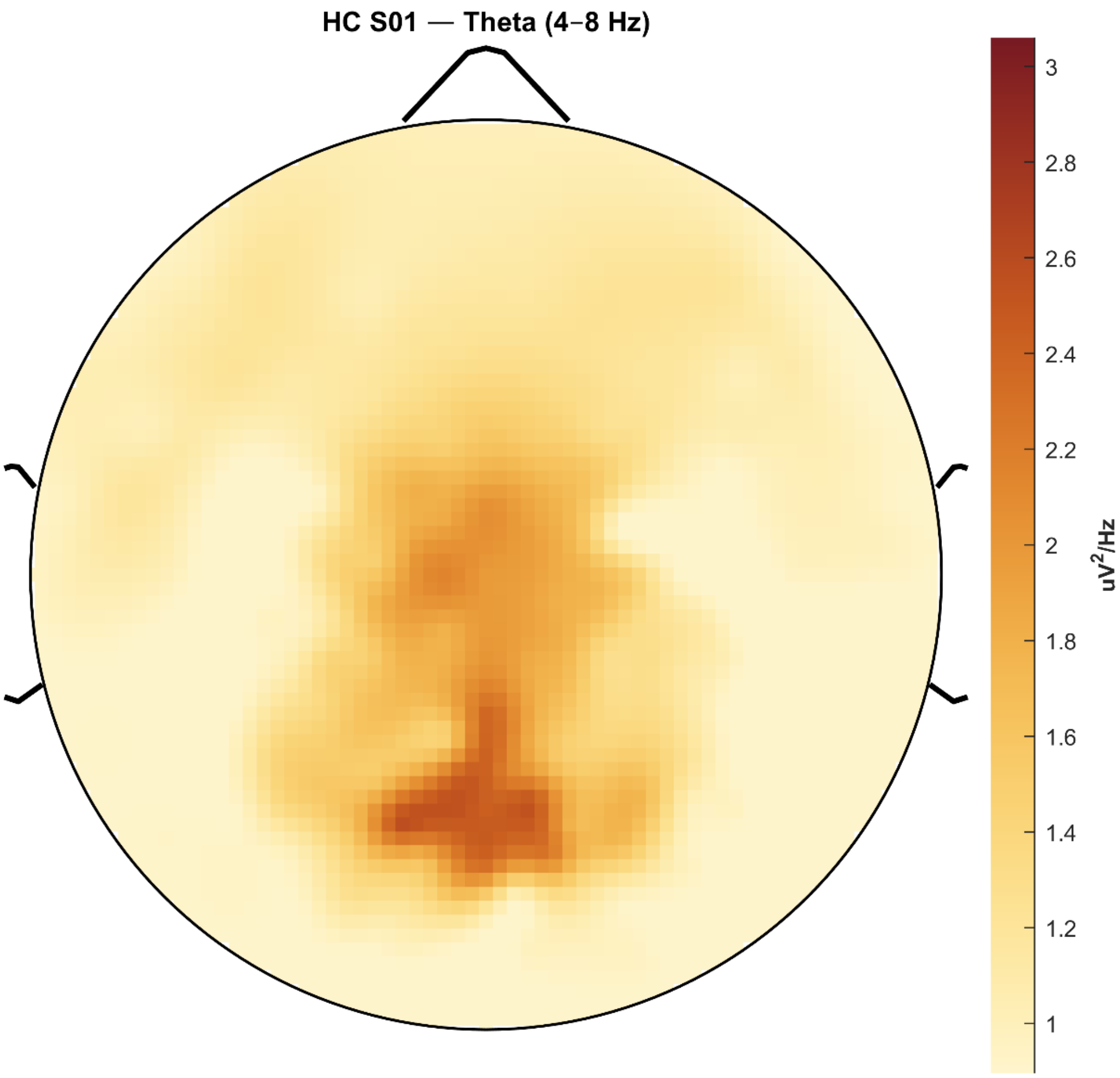

Supplementary Figure 128

Theta (4-8 Hz) power map for HC subject S01. The color scale represents theta power (μV²/Hz).

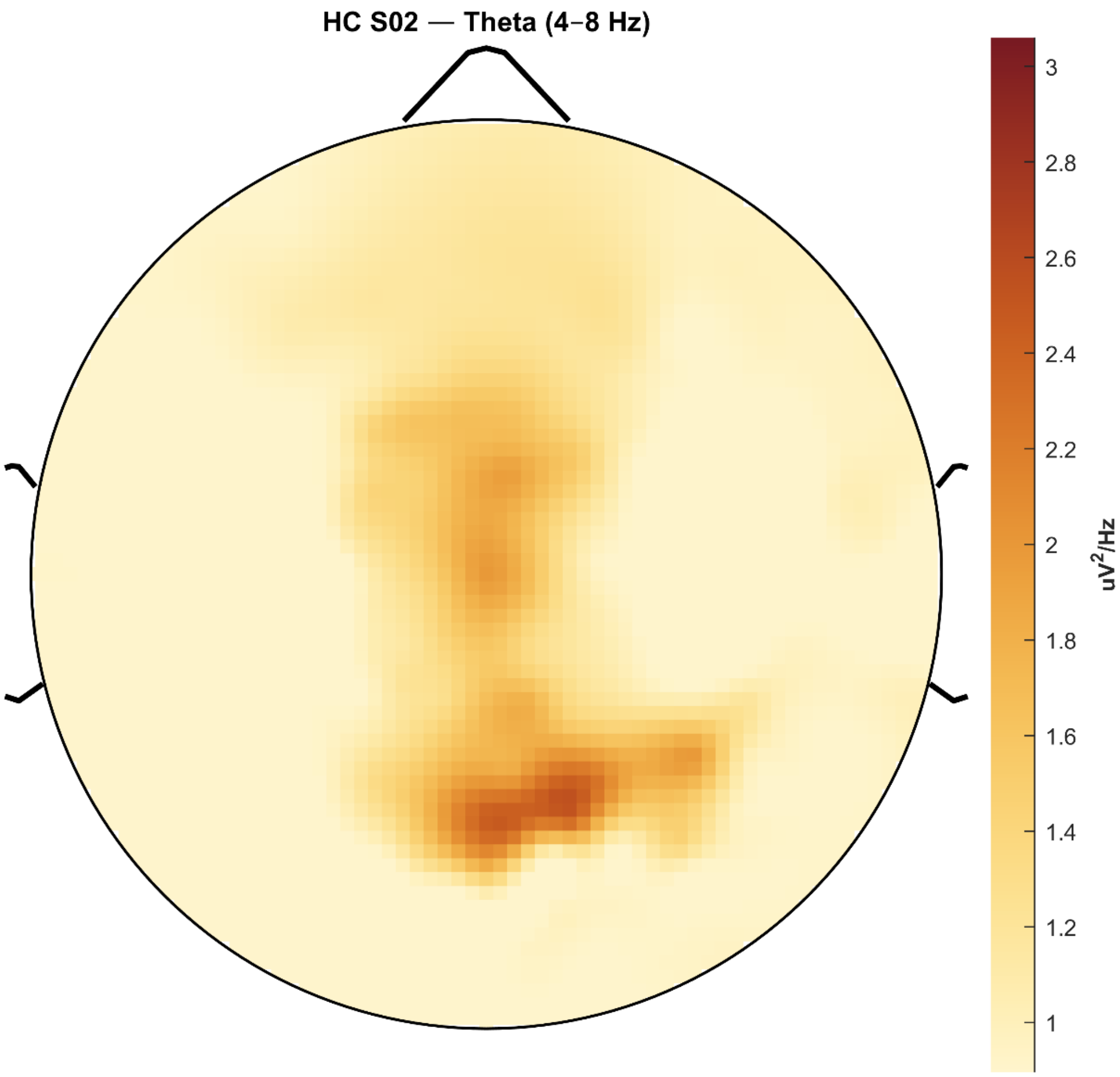

Supplementary Figure 129

Theta (4-8 Hz) power map for HC subject S02. The color scale represents theta power (μV²/Hz).

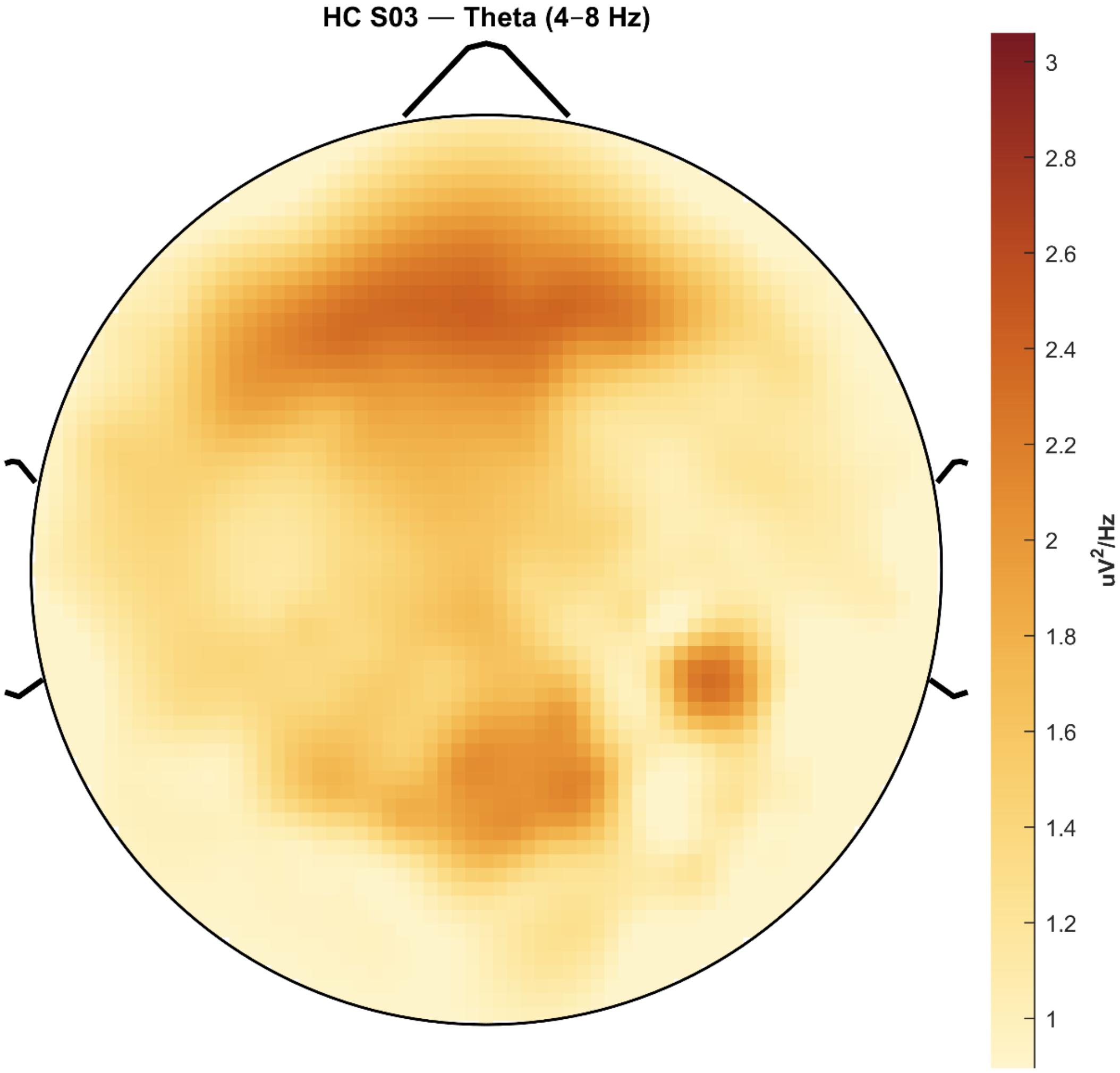

Supplementary Figure 130

Theta (4-8 Hz) power map for HC subject S03. The color scale represents theta power (μV²/Hz).

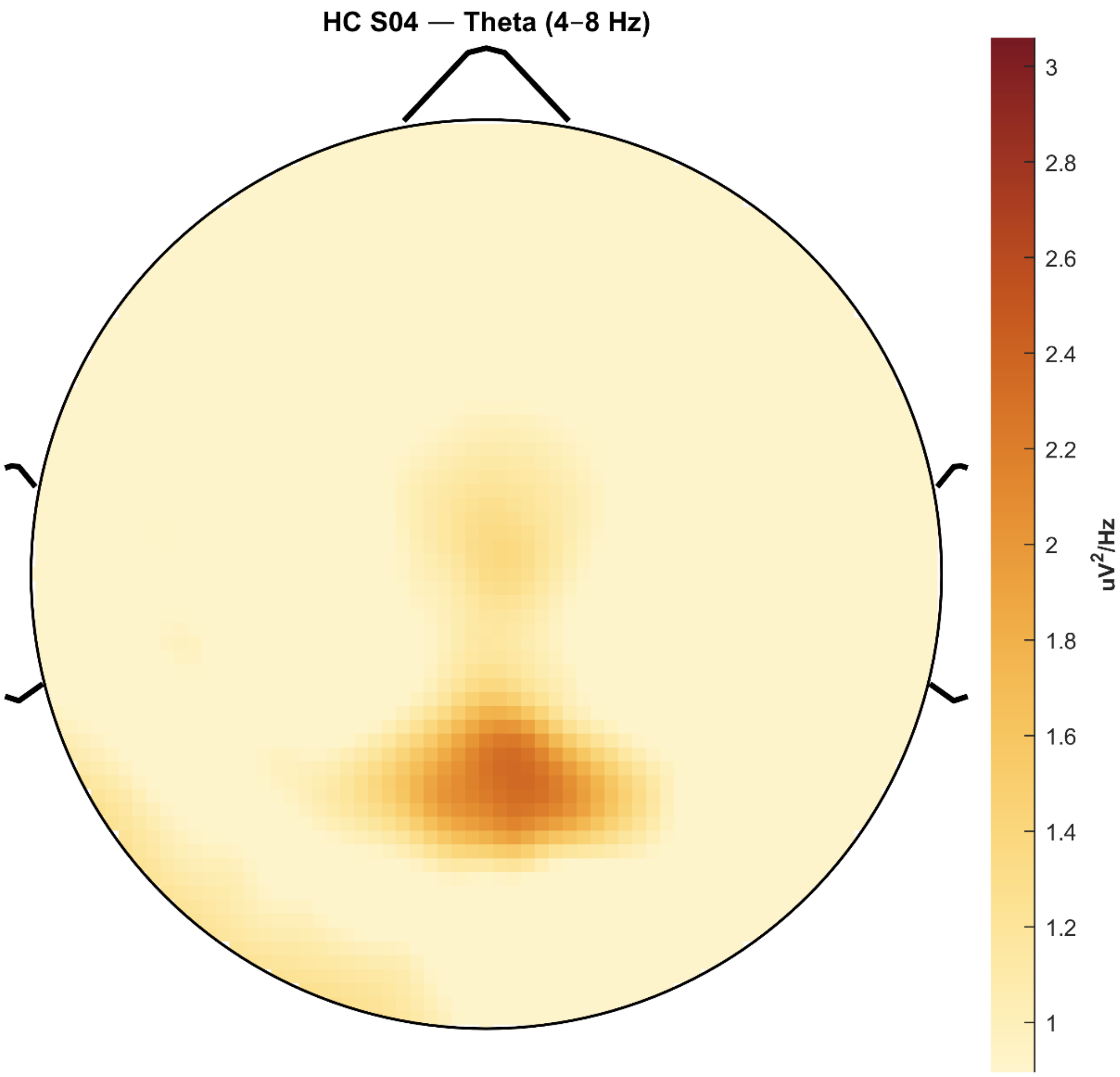

Supplementary Figure 131

Theta (4-8 Hz) power map for HC subject S04. The color scale represents theta power (μV²/Hz).

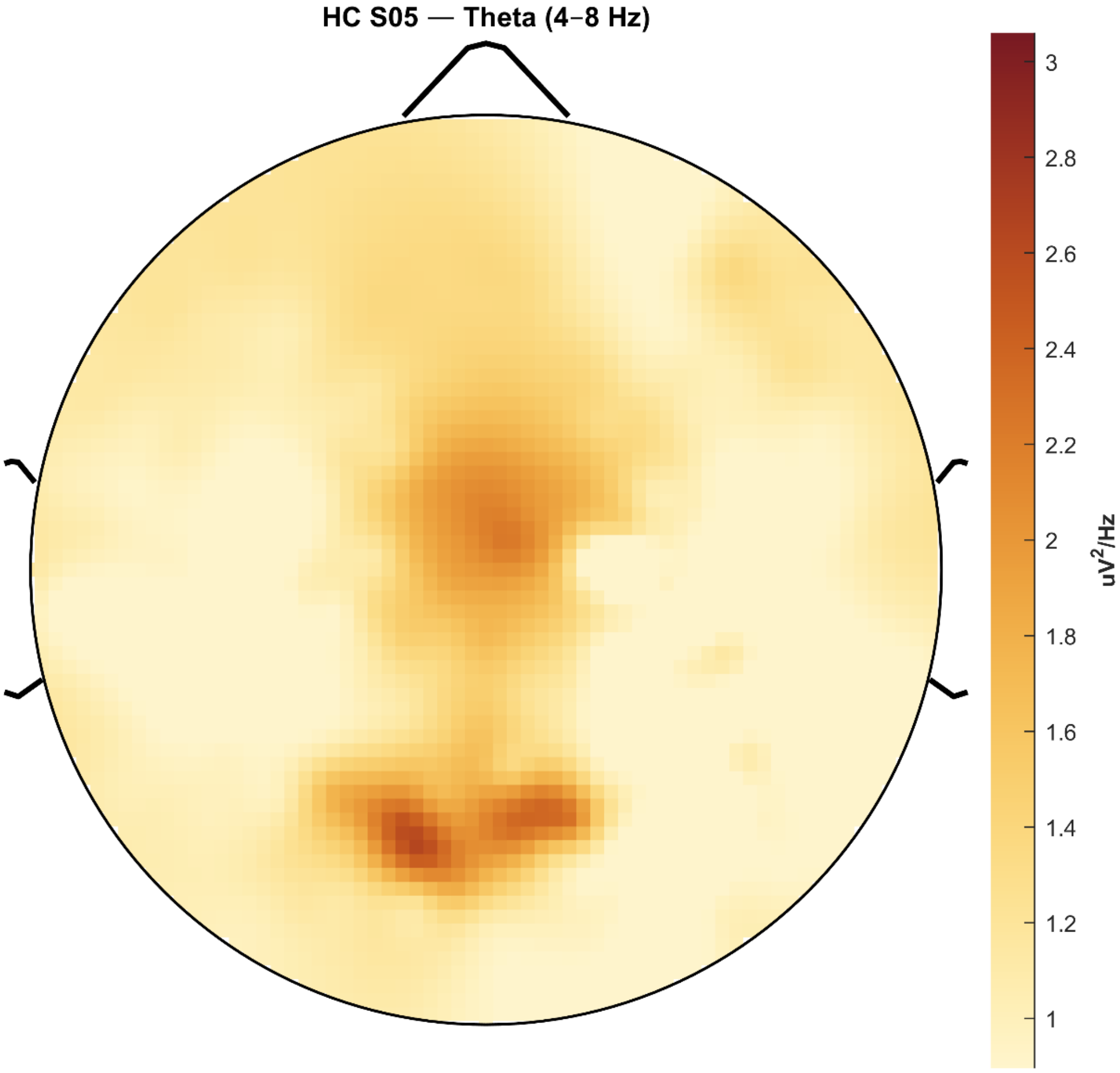

Supplementary Figure 132

Theta (4-8 Hz) power map for HC subject S05. The color scale represents theta power (μV²/Hz).

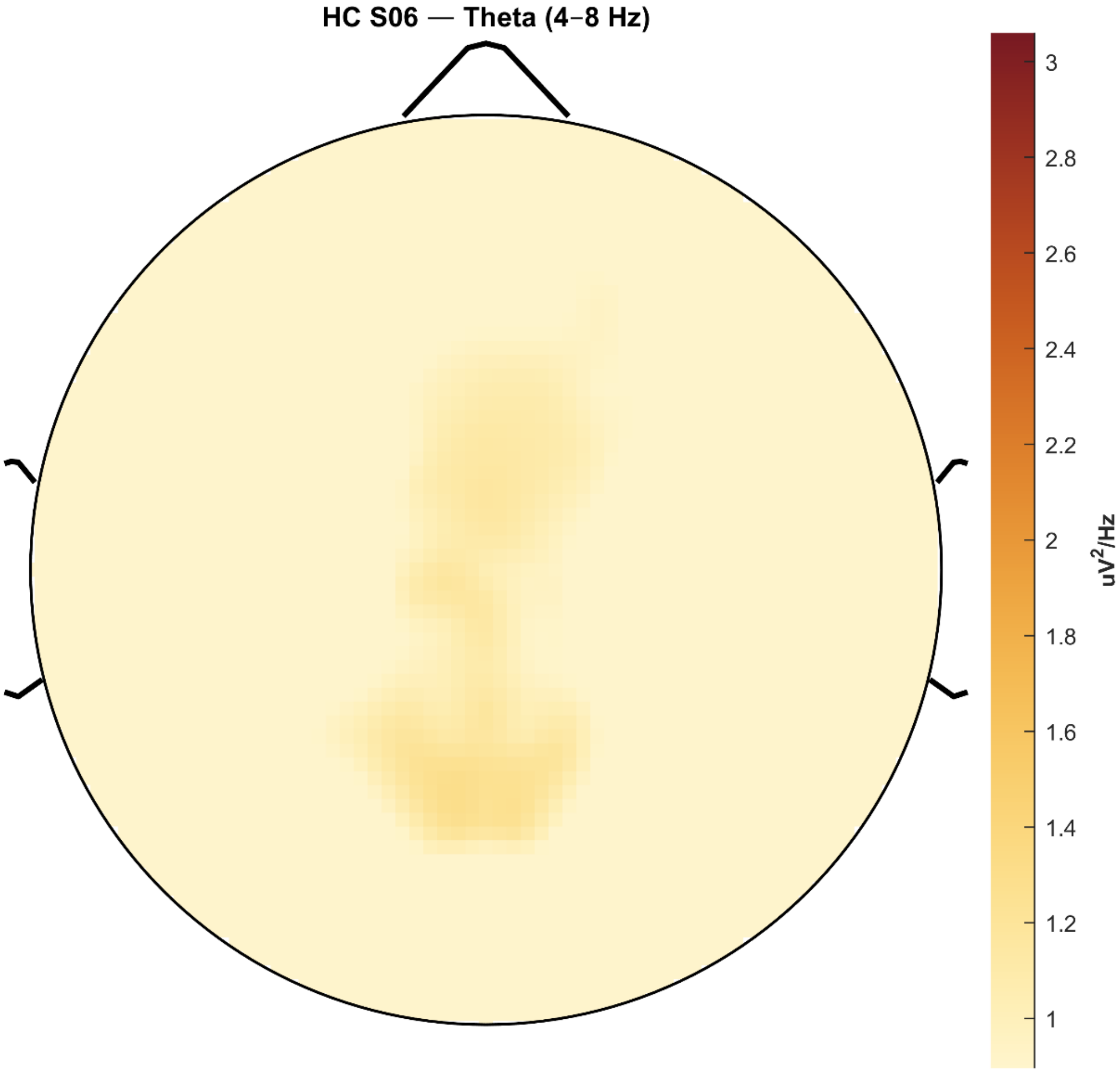

Supplementary Figure 133

Theta (4-8 Hz) power map for HC subject S06. The color scale represents theta power (μV²/Hz).

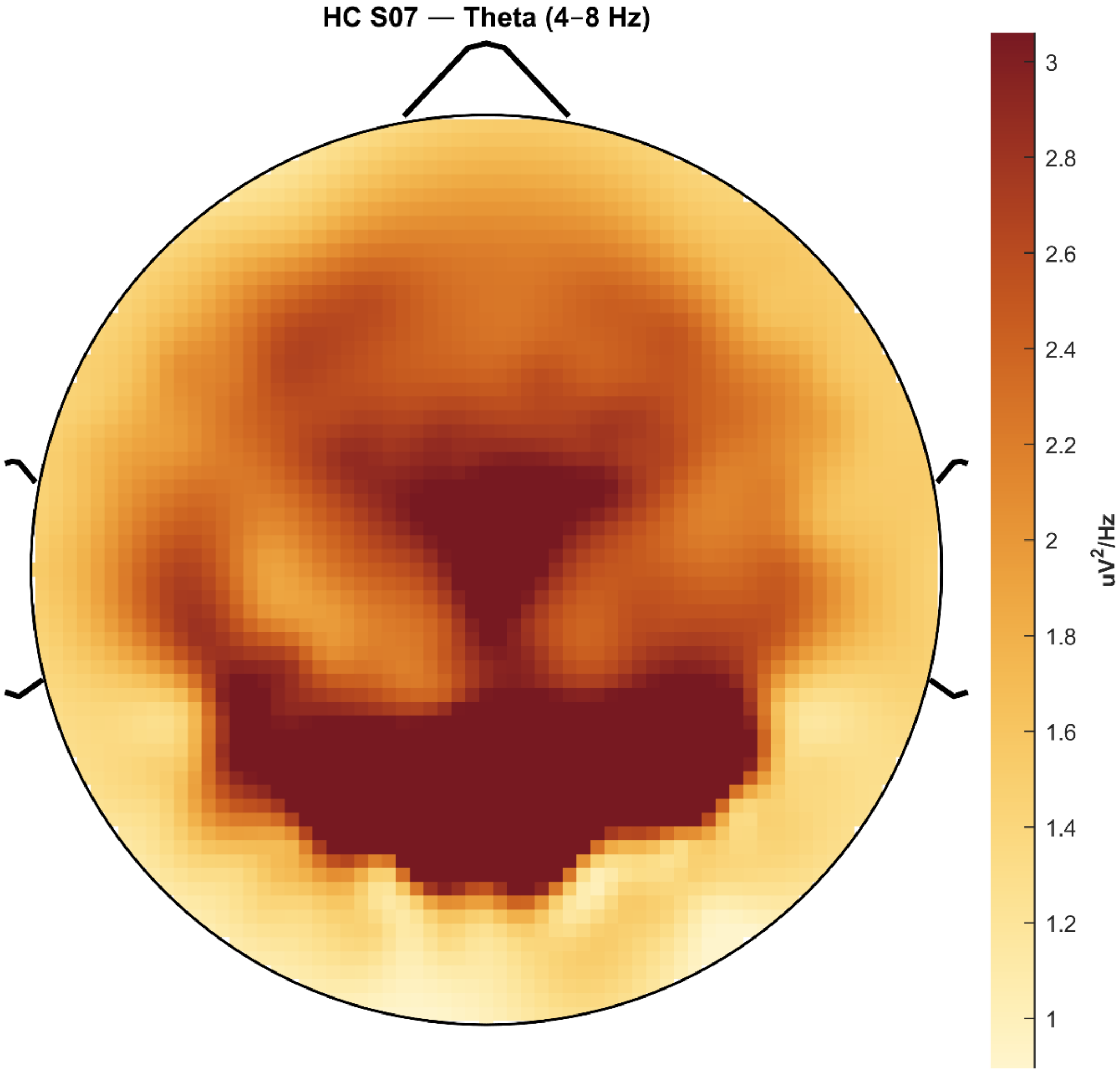

Supplementary Figure 134

Theta (4-8 Hz) power map for HC subject S07. The color scale represents theta power (μV²/Hz).

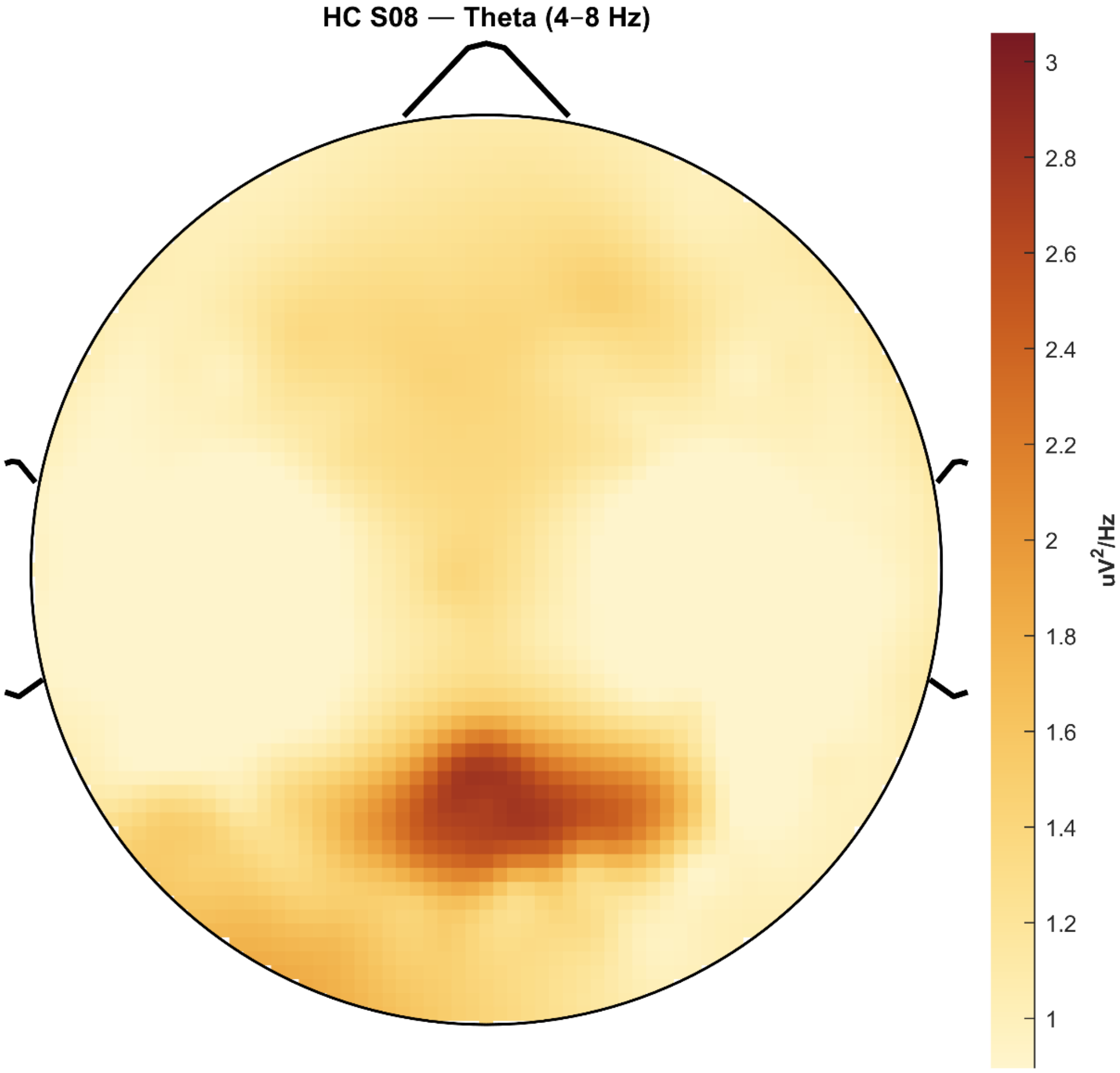

Supplementary Figure 135

Theta (4-8 Hz) power map for HC subject S08. The color scale represents theta power (μV²/Hz).

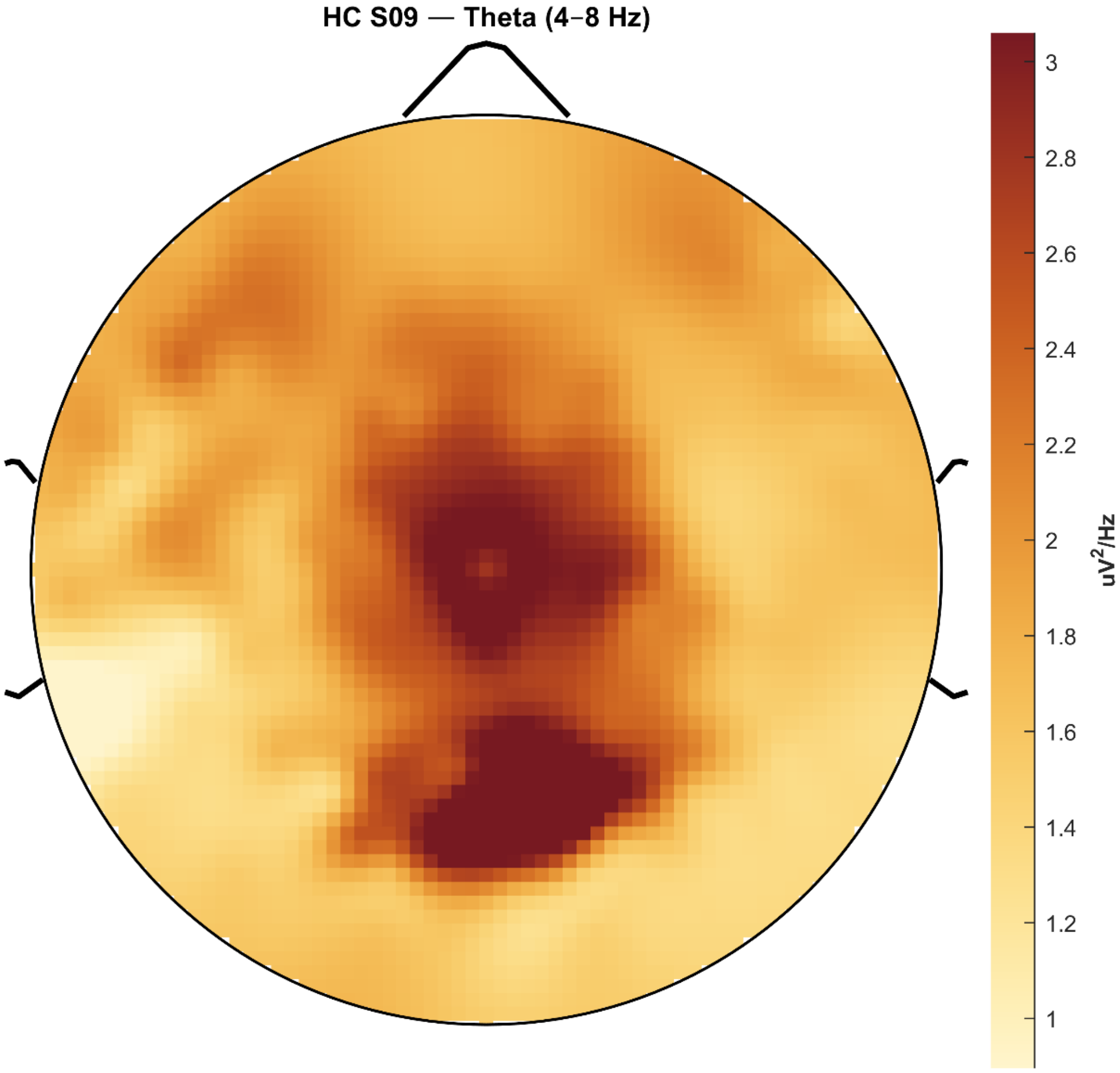

Supplementary Figure 136

Theta (4-8 Hz) power map for HC subject S09. The color scale represents theta power (µV²/Hz).

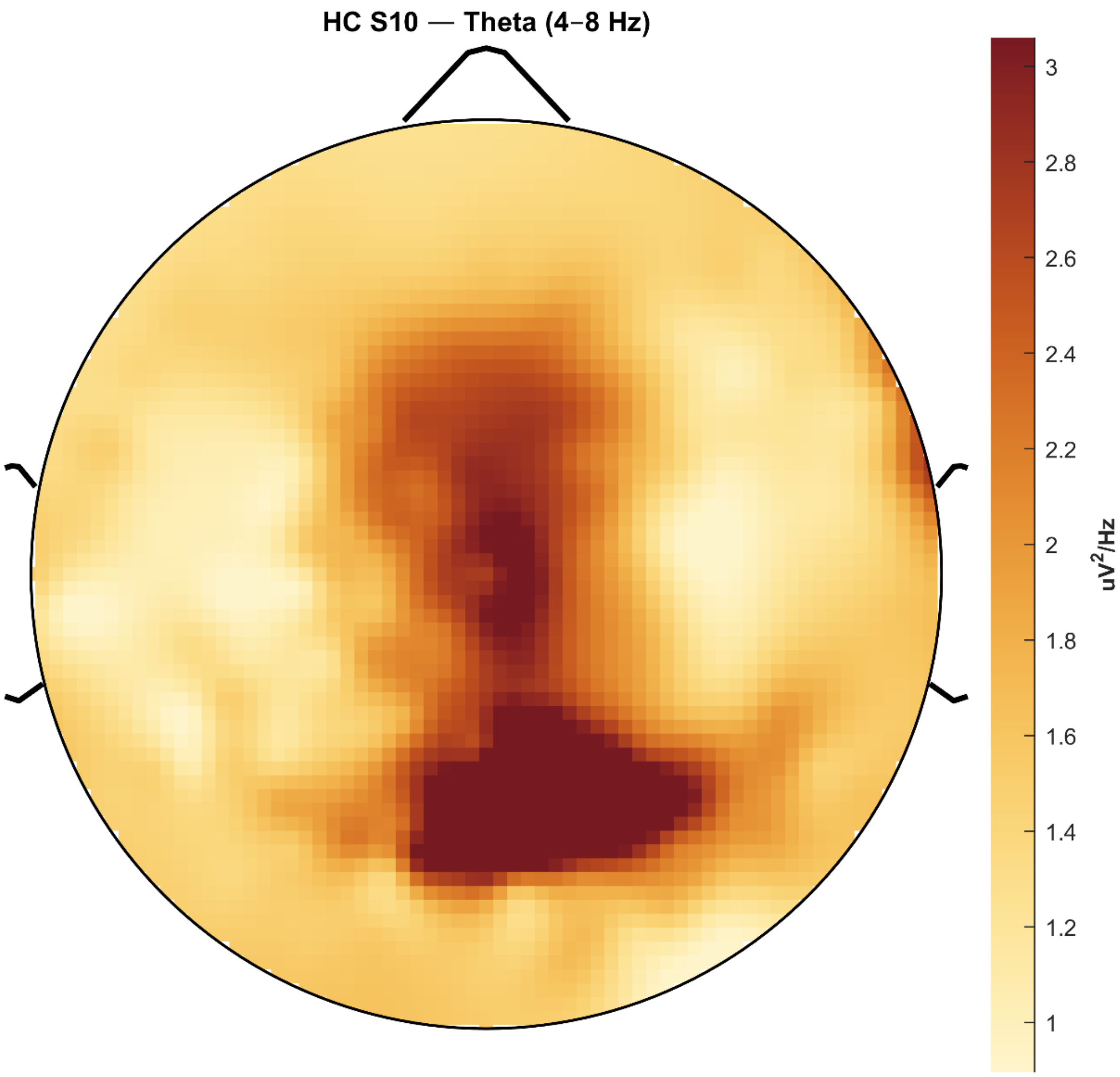

Supplementary Figure 137

Theta (4-8 Hz) power map for HC subject S10. The color scale represents theta power (μV²/Hz).

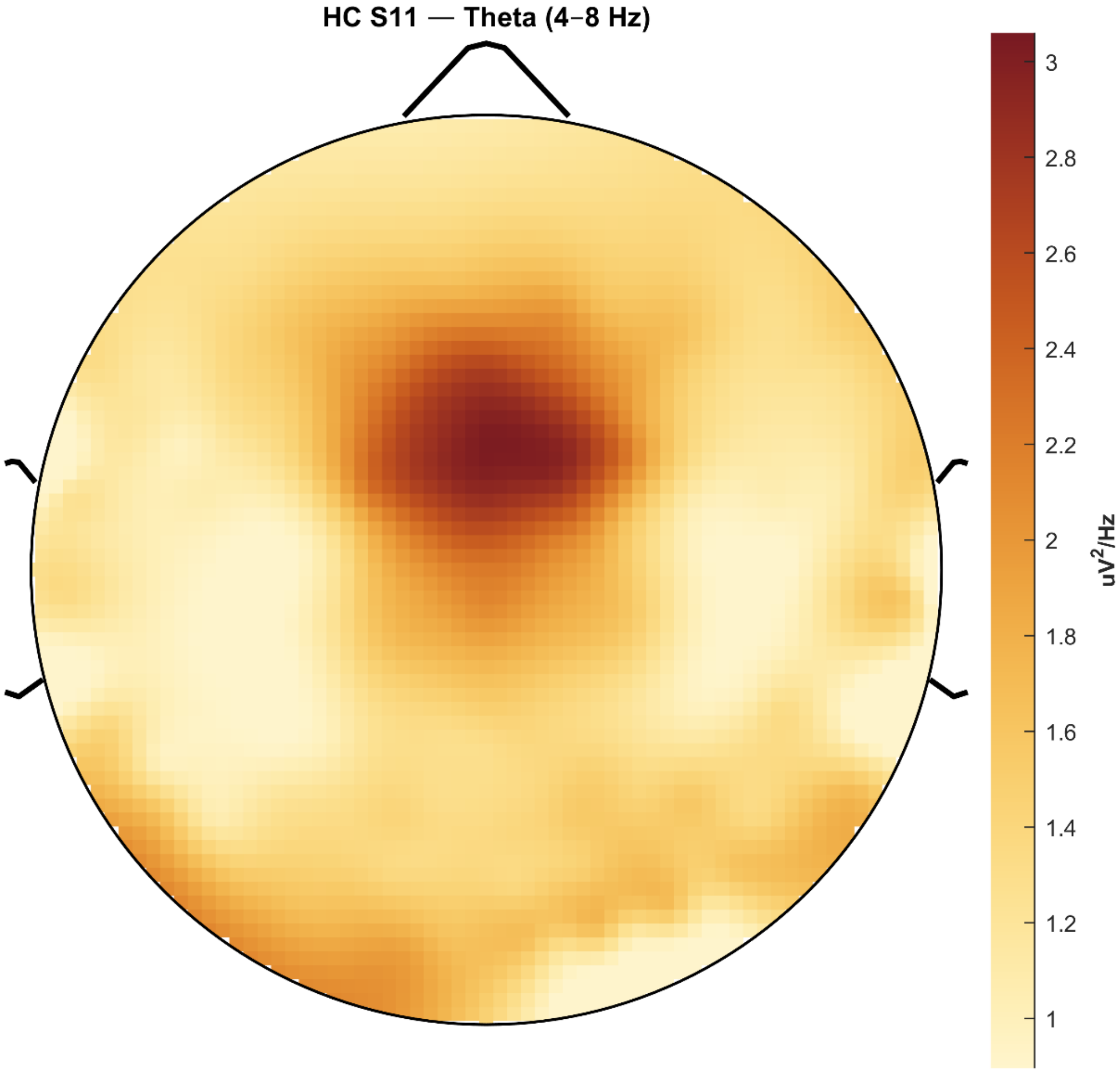

Supplementary Figure 138

Theta (4-8 Hz) power map for HC subject S11. The color scale represents theta power (µV²/Hz).

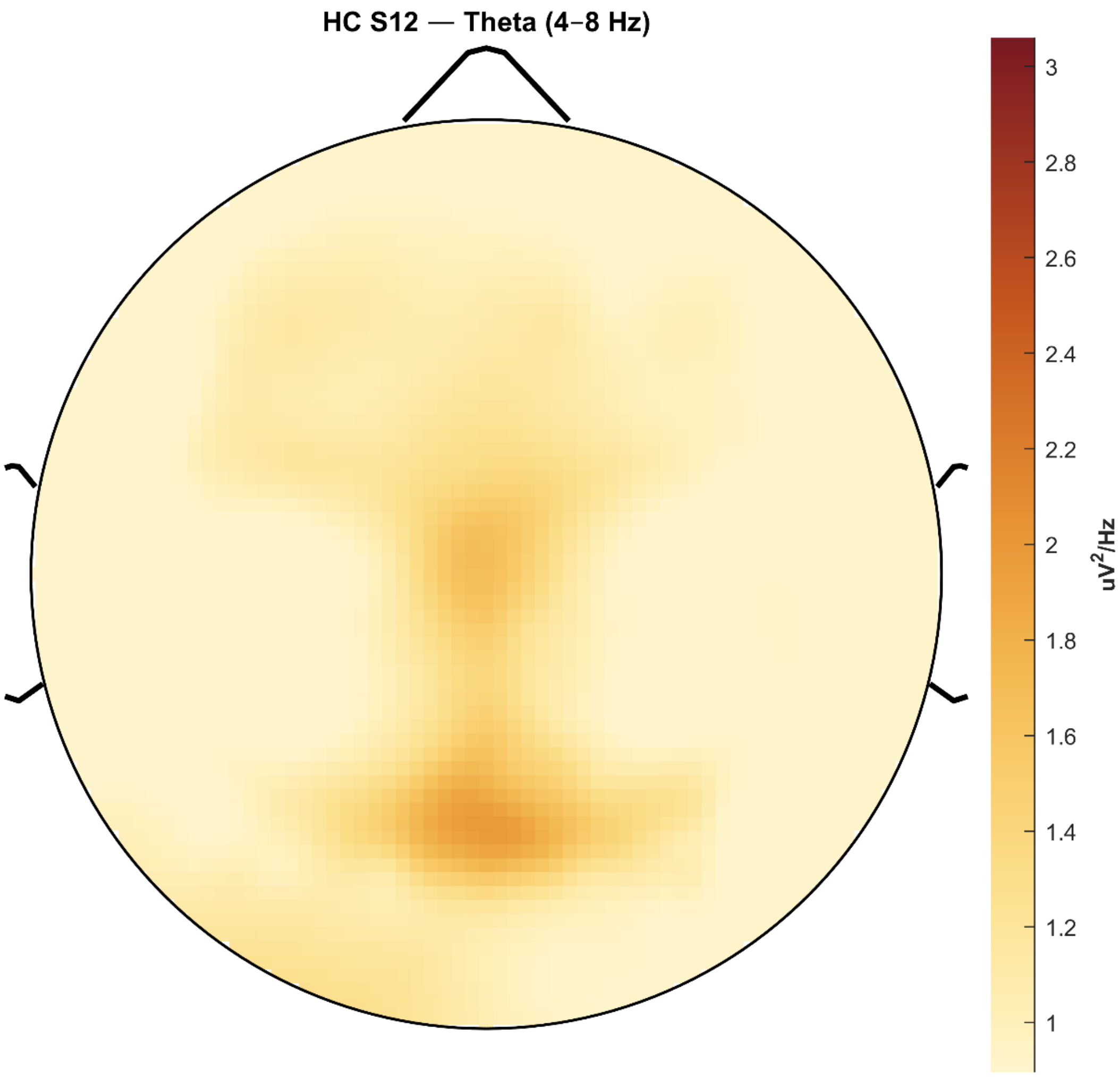

Supplementary Figure 139

Theta (4-8 Hz) power map for HC subject S12. The color scale represents theta power (μV²/Hz).

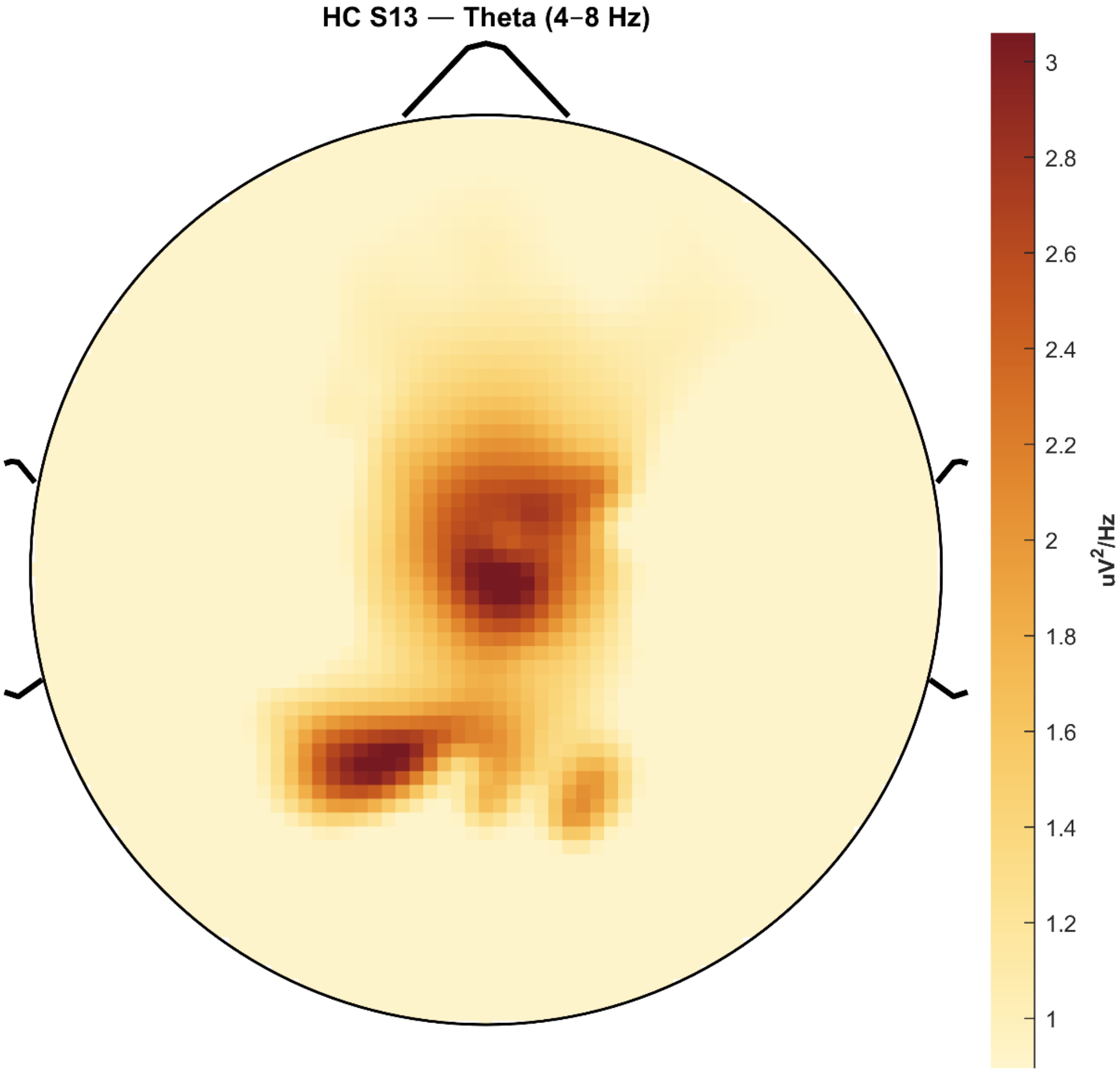

Supplementary Figure 140

Theta (4-8 Hz) power map for HC subject S13. The color scale represents theta power (µV²/Hz).

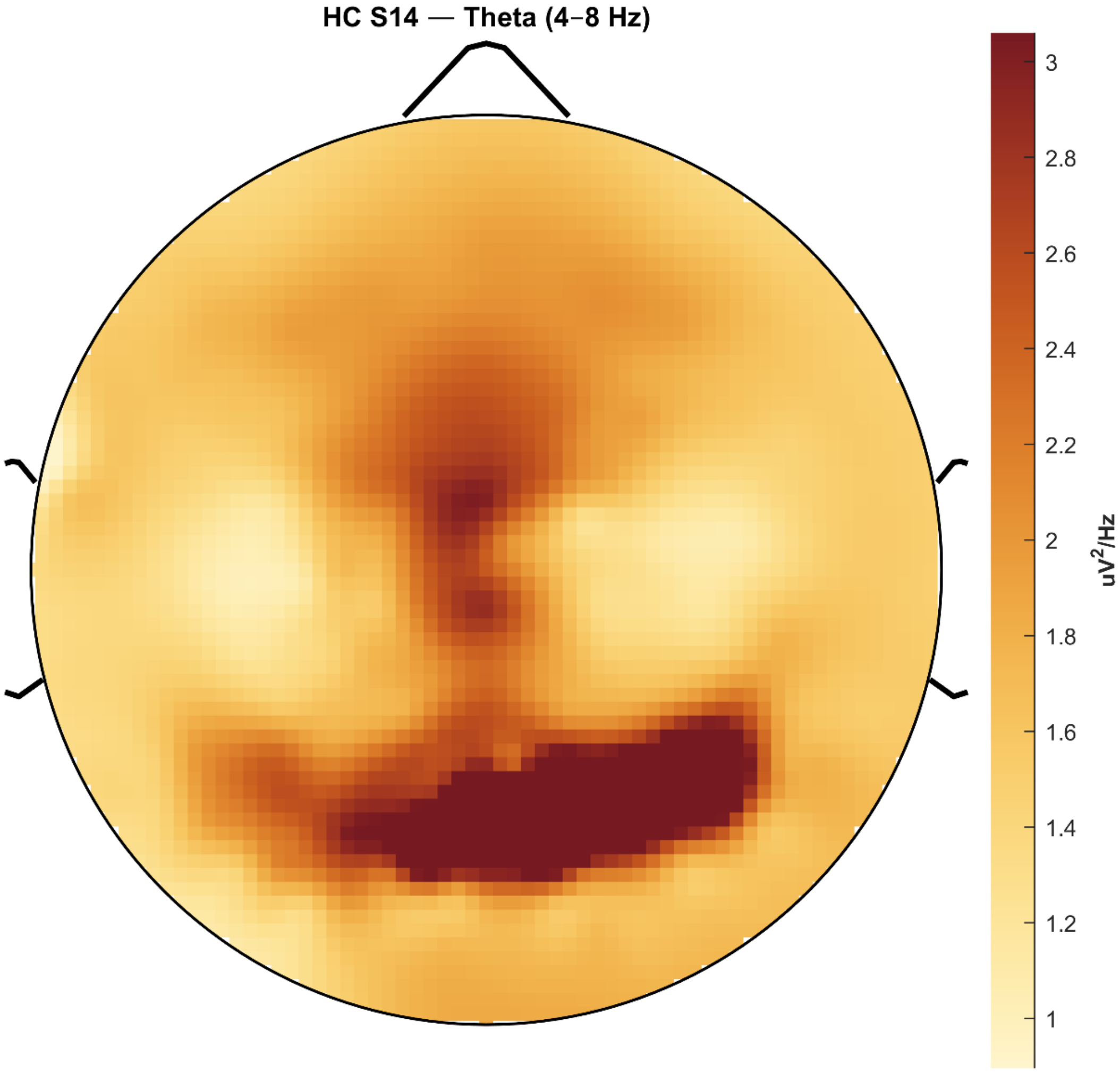

Supplementary Figure 141

Theta (4-8 Hz) power map for HC subject S14. The color scale represents theta power (µV²/Hz).

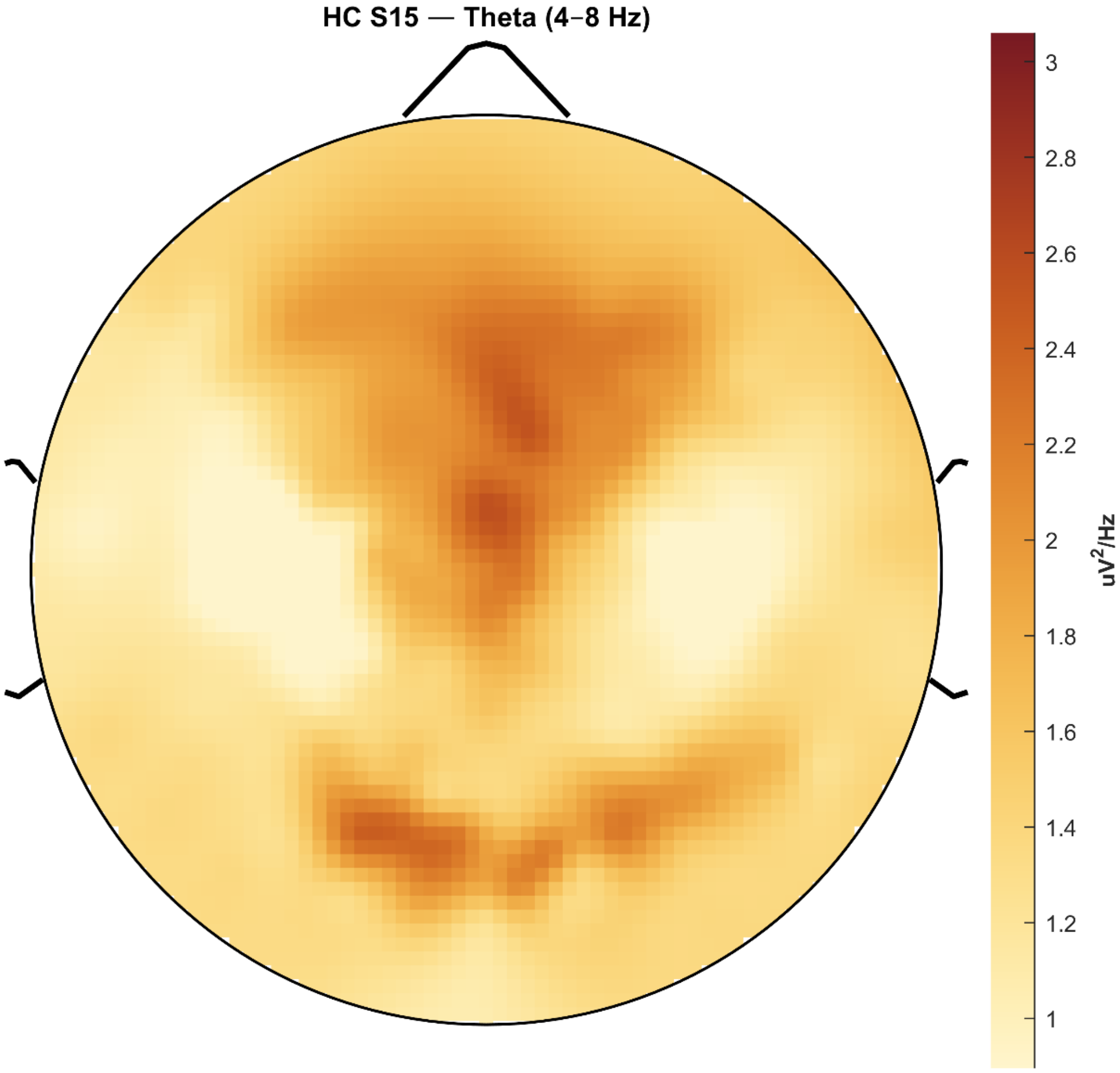

Supplementary Figure 142

Theta (4-8 Hz) power map for HC subject S15. The color scale represents theta power (µV²/Hz).

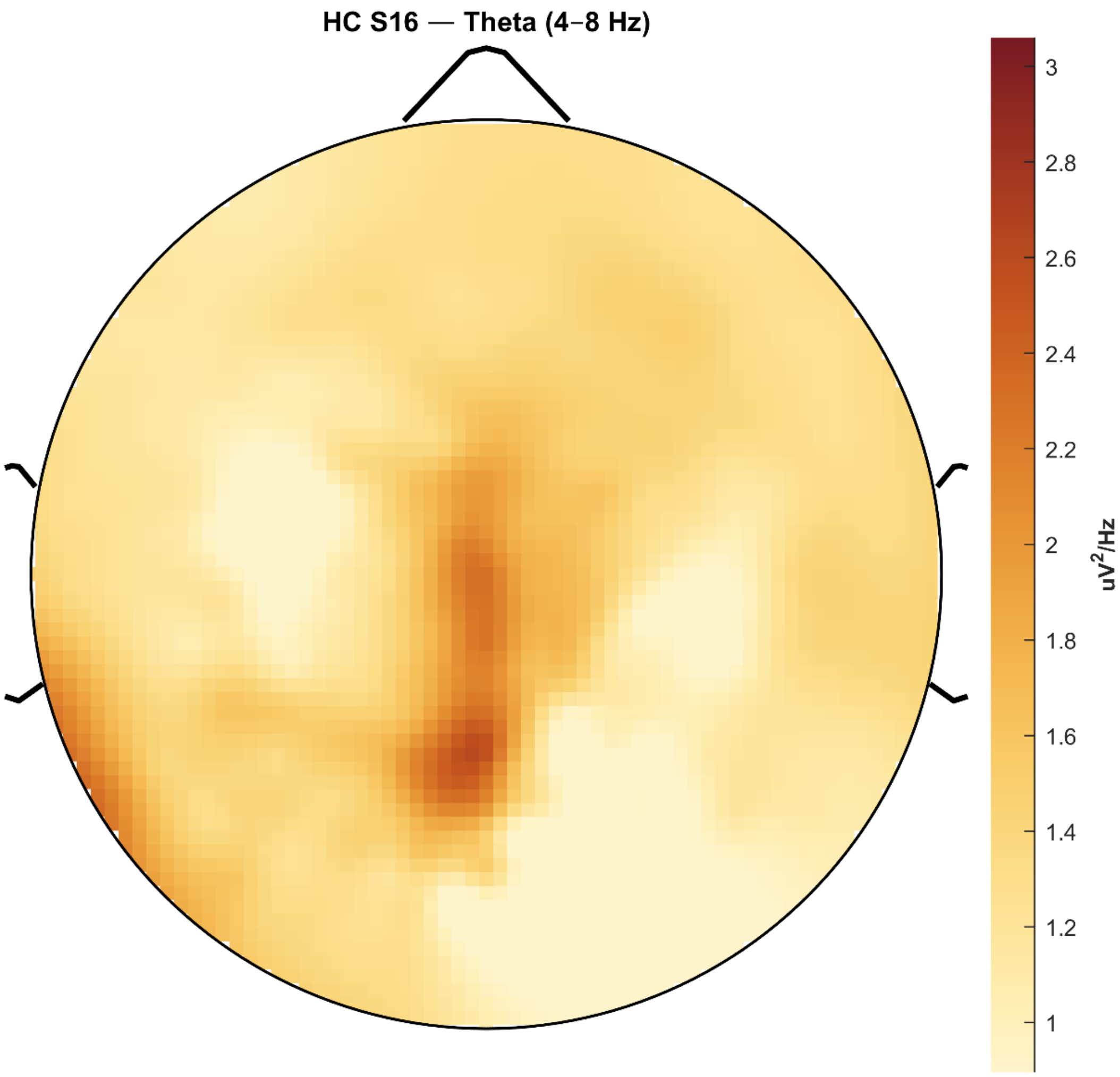

Supplementary Figure 143

Theta (4-8 Hz) power map for HC subject S16. The color scale represents theta power (μV²/Hz).

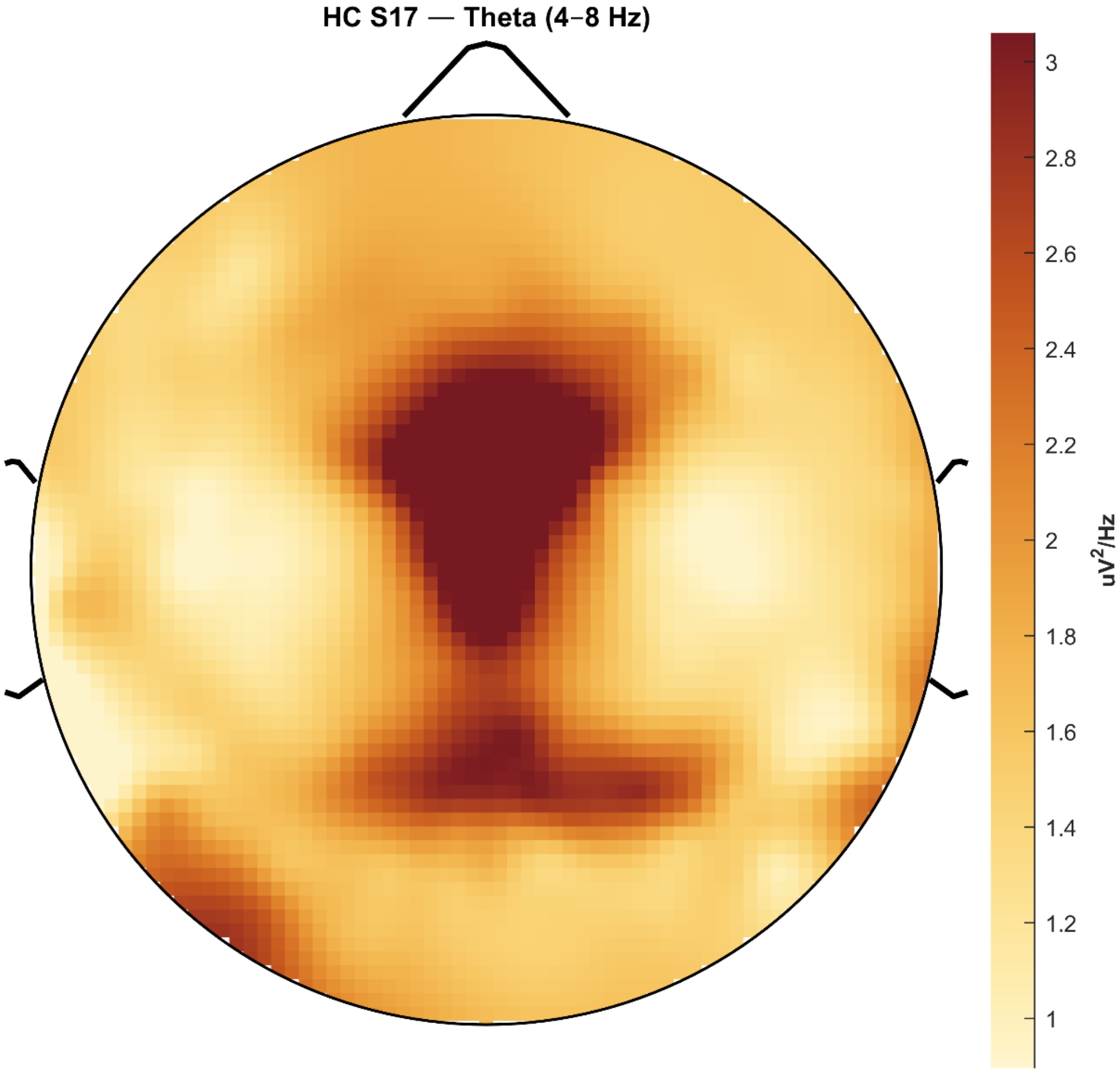

Supplementary Figure 144

Theta (4-8 Hz) power map for HC subject S17. The color scale represents theta power (μV²/Hz).

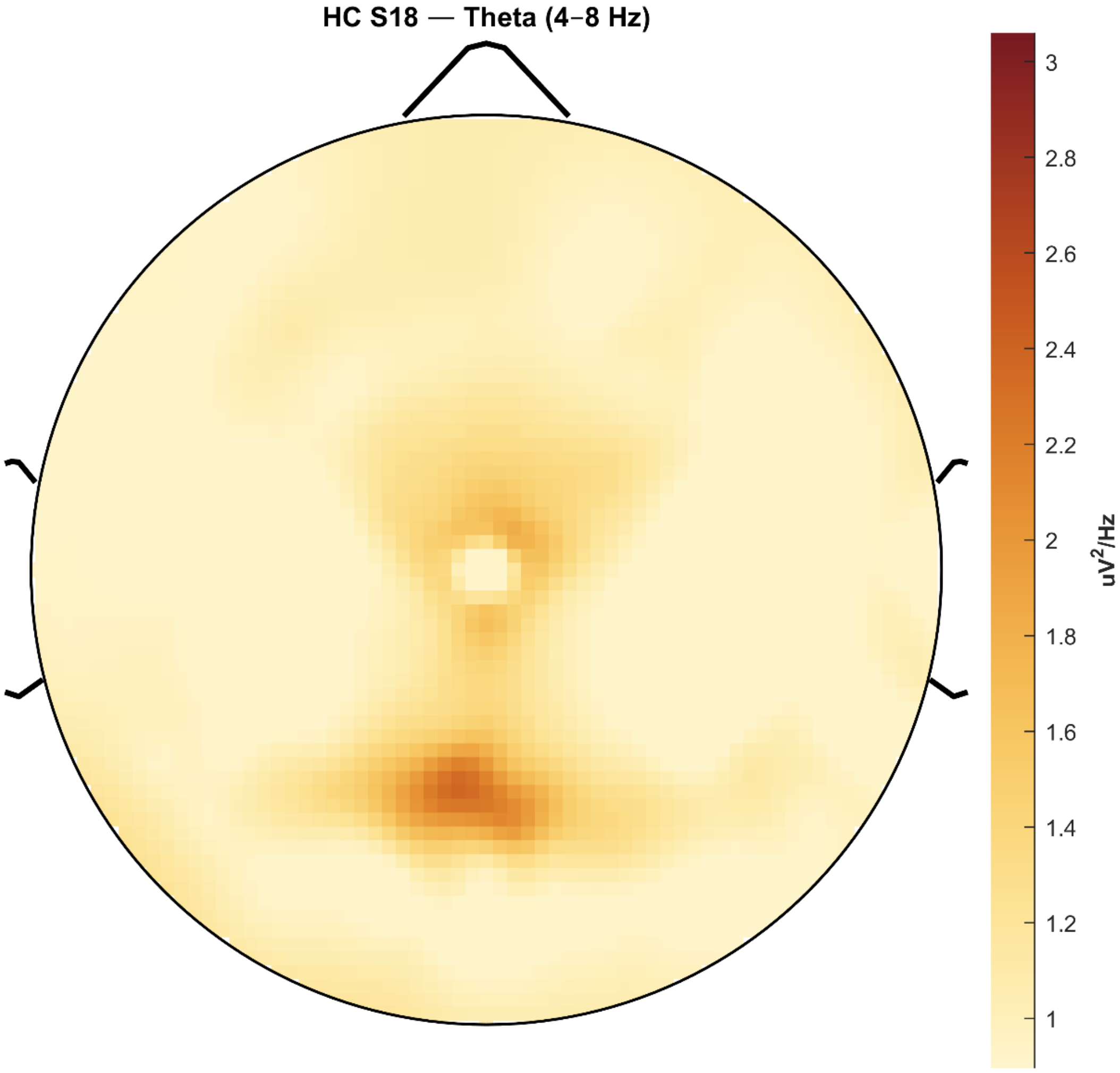

Supplementary Figure 145

Theta (4-8 Hz) power map for HC subject S18. The color scale represents theta power (μV²/Hz).

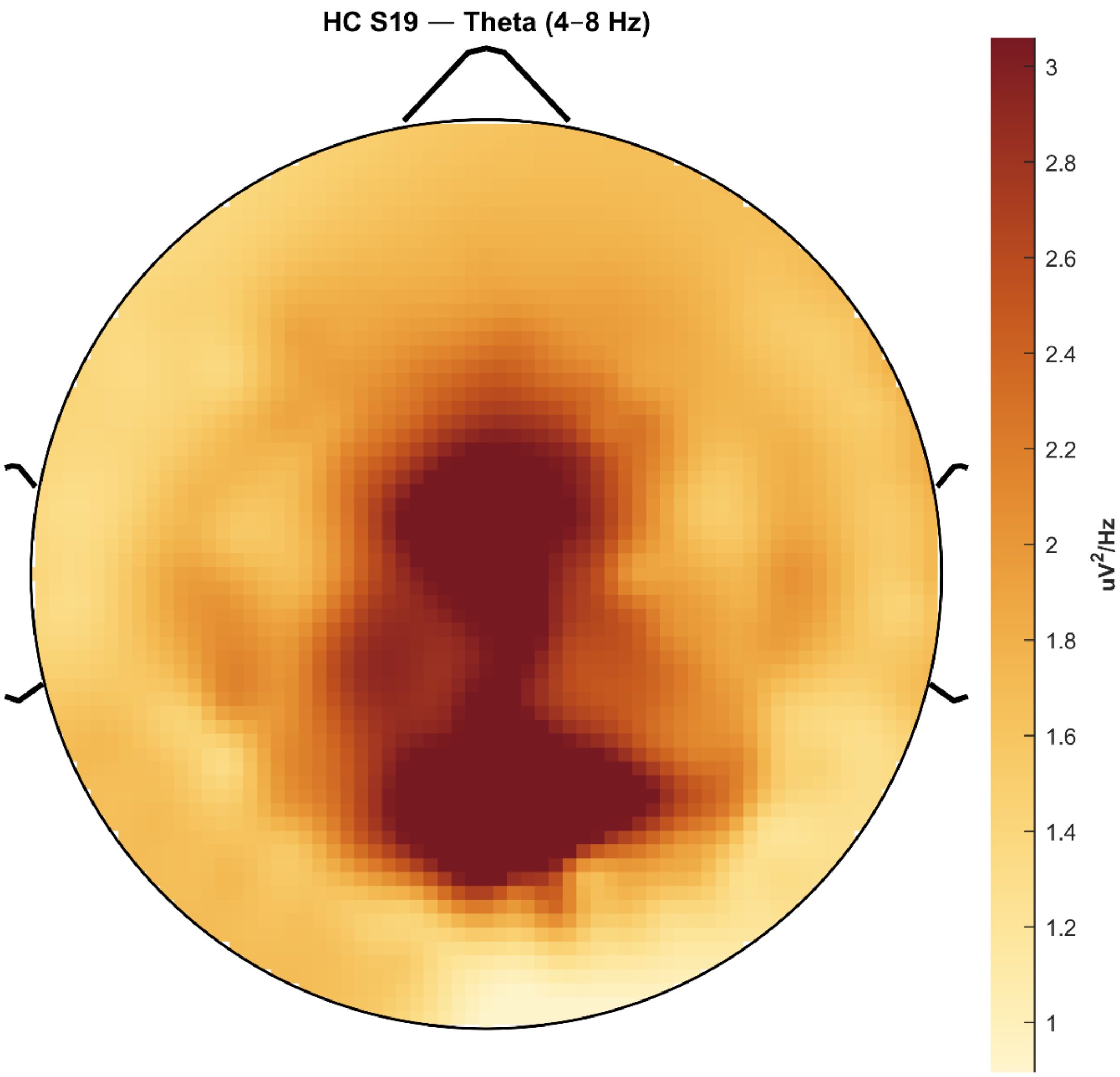

Supplementary Figure 146

Theta (4-8 Hz) power map for HC subject S19. The color scale represents theta power (µV²/Hz).

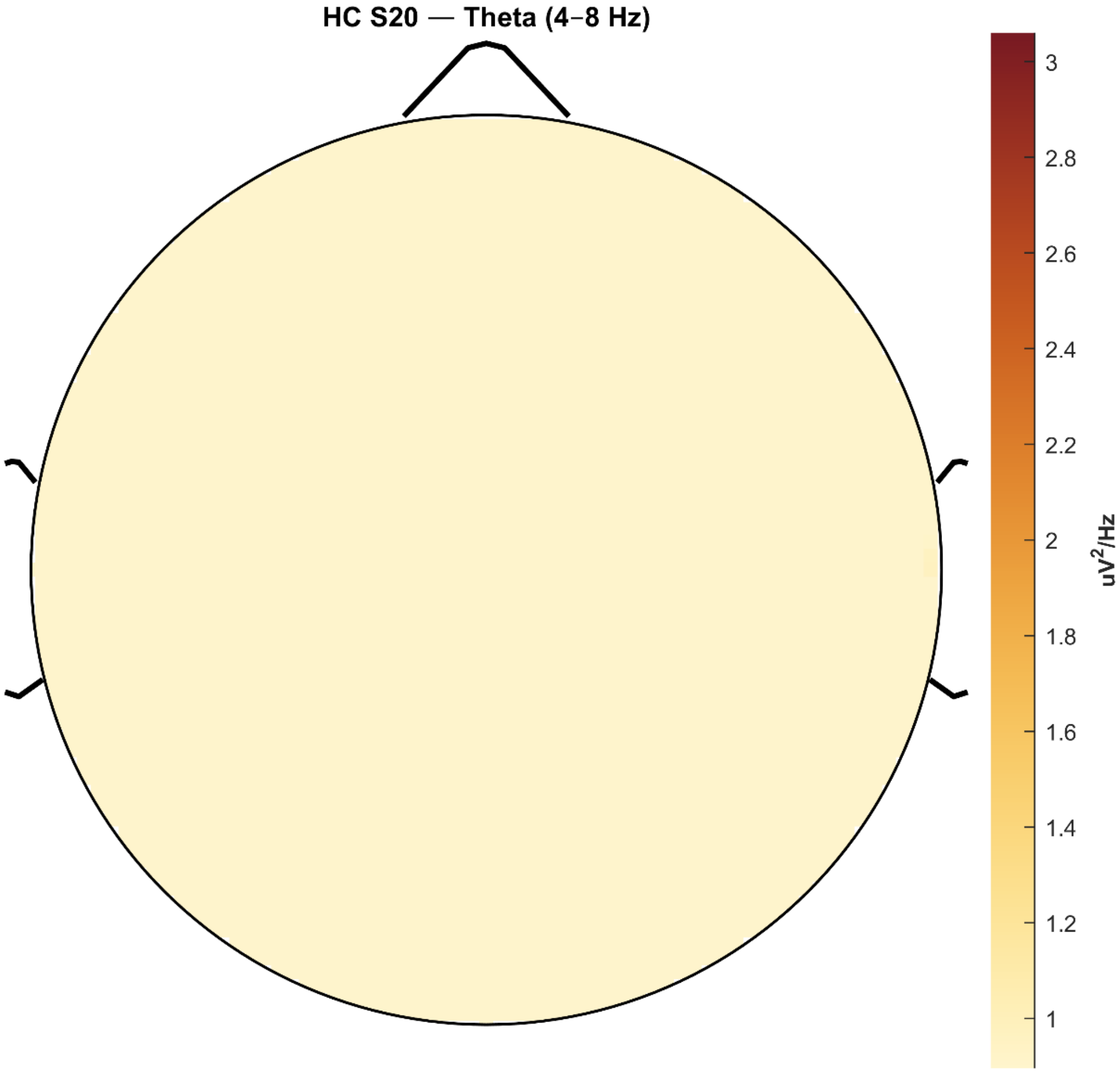

Supplementary Figure 147

Theta (4-8 Hz) power map for HC subject S20. The color scale represents theta power (µV²/Hz).

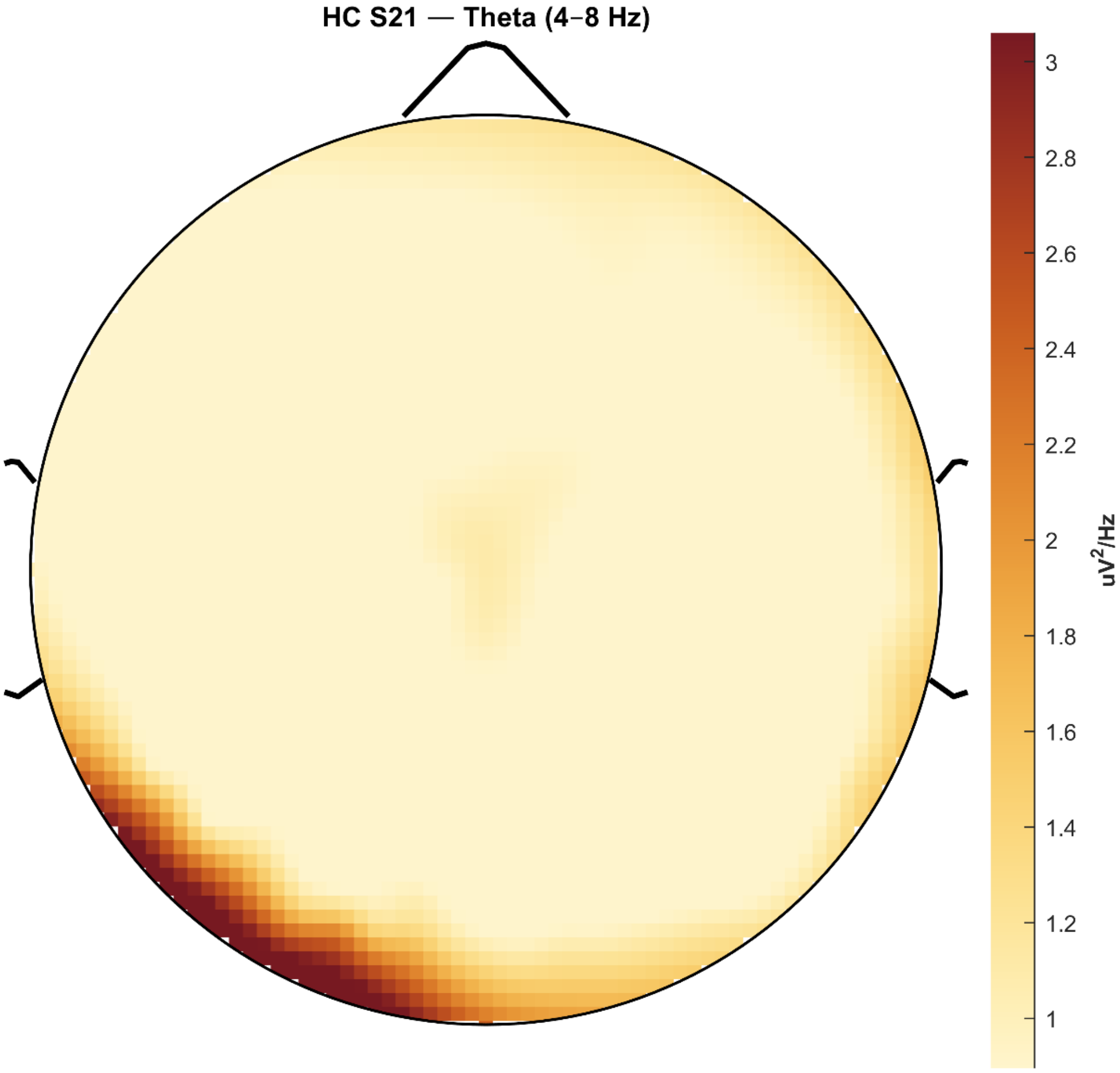

Supplementary Figure 148

Theta (4-8 Hz) power map for HC subject S21. The color scale represents theta power (µV²/Hz).

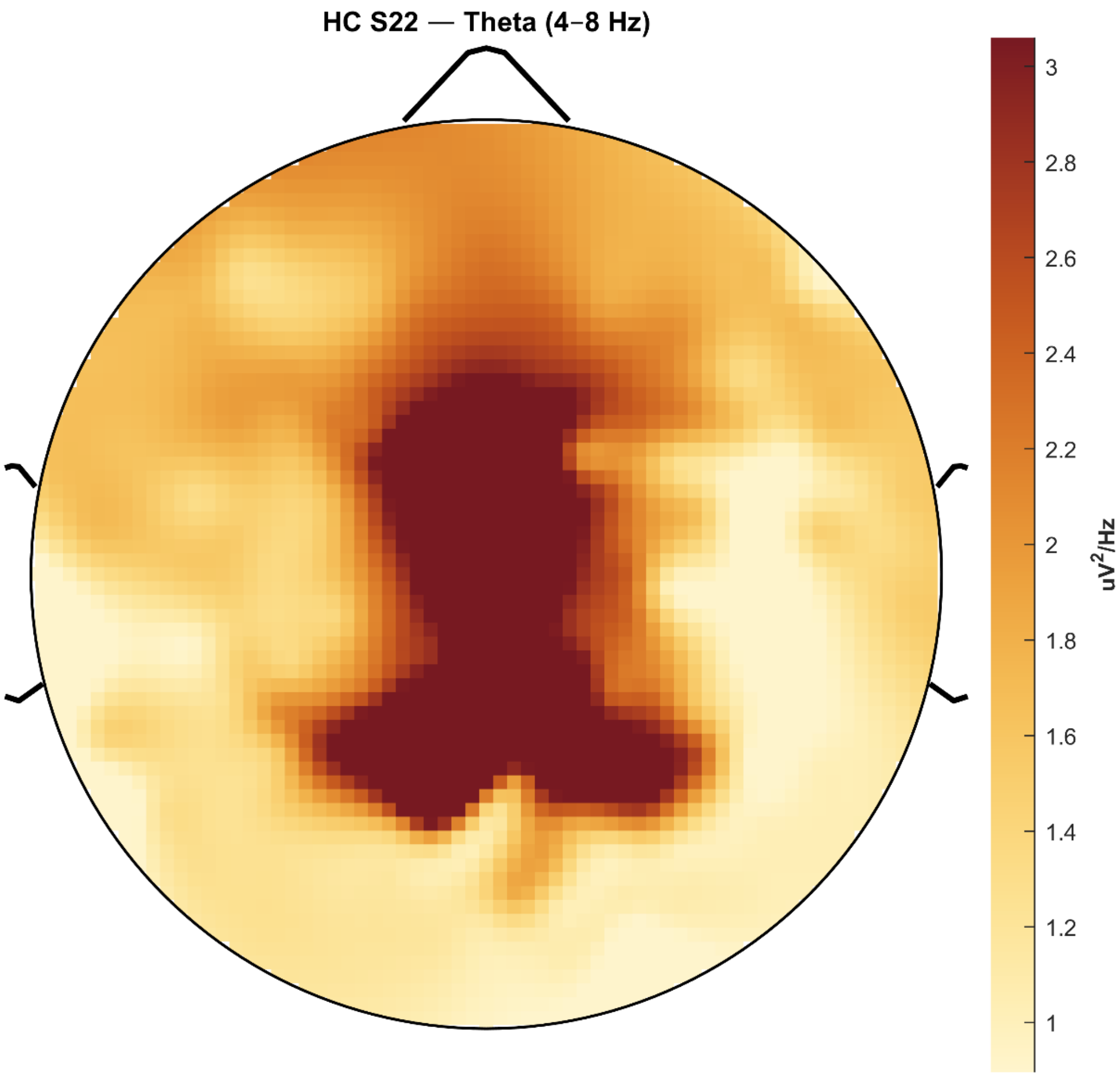

Supplementary Figure 149

Theta (4-8 Hz) power map for HC subject S22. The color scale represents theta power (μV²/Hz).

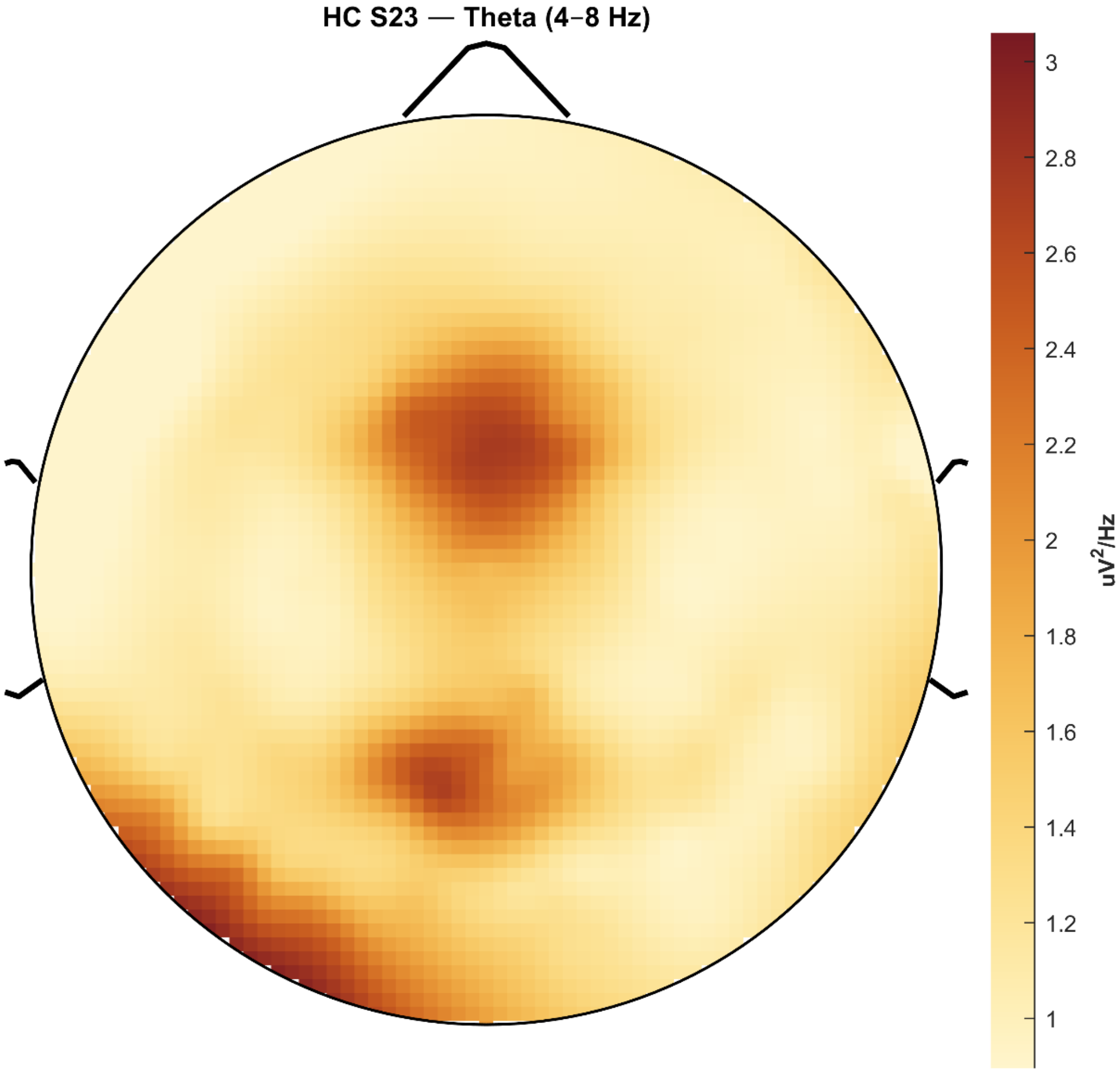

Supplementary Figure 150

Theta (4-8 Hz) power map for HC subject S23. The color scale represents theta power (μV²/Hz).

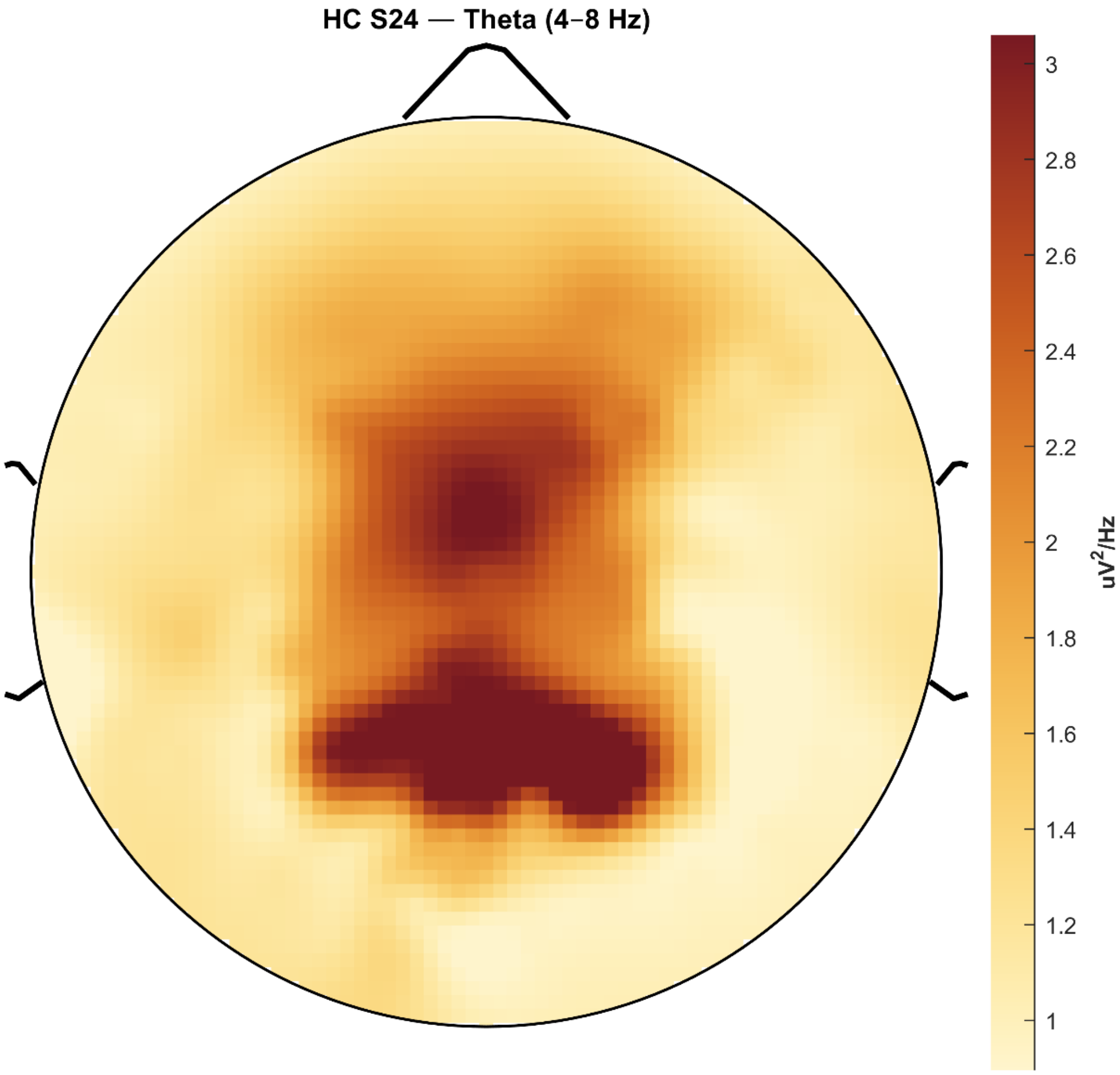

Supplementary Figure 151

Theta (4-8 Hz) power map for HC subject S24. The color scale represents theta power (µV²/Hz).

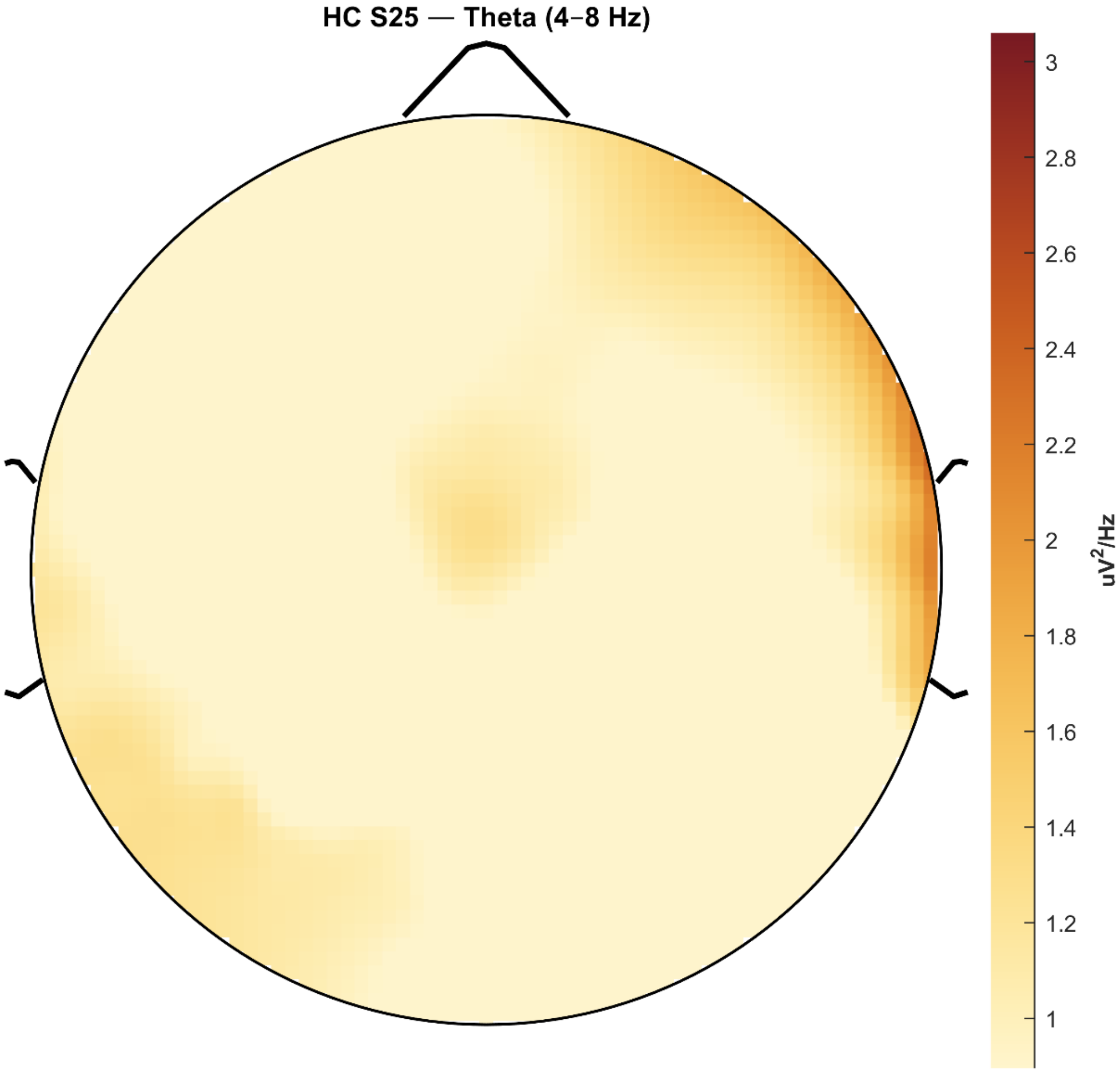

Supplementary Figure 152

Theta (4-8 Hz) power map for HC subject S25. The color scale represents theta power (μV²/Hz).

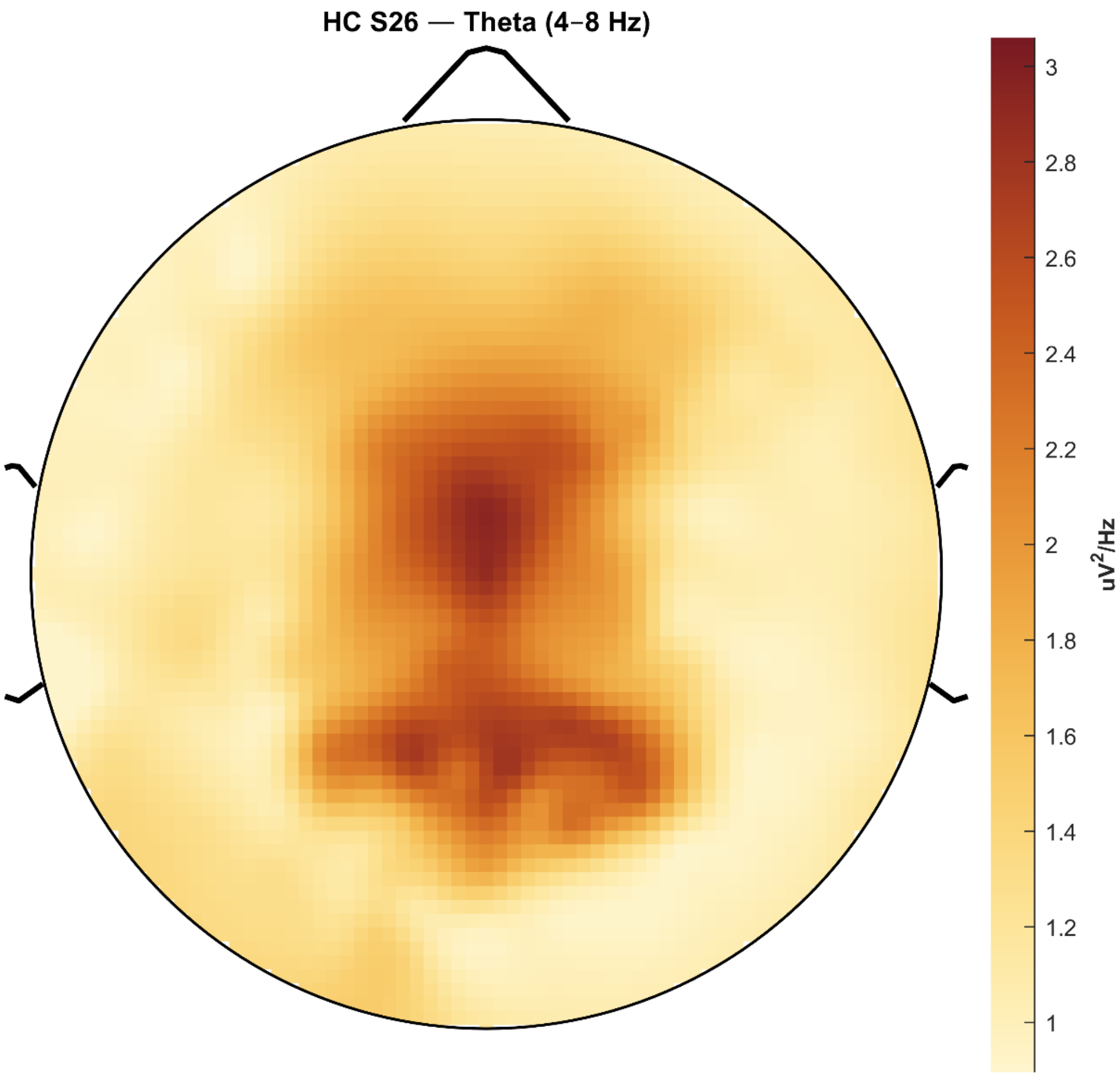

Supplementary Figure 153

Theta (4-8 Hz) power map for HC subject S26. The color scale represents theta power (µV²/Hz).

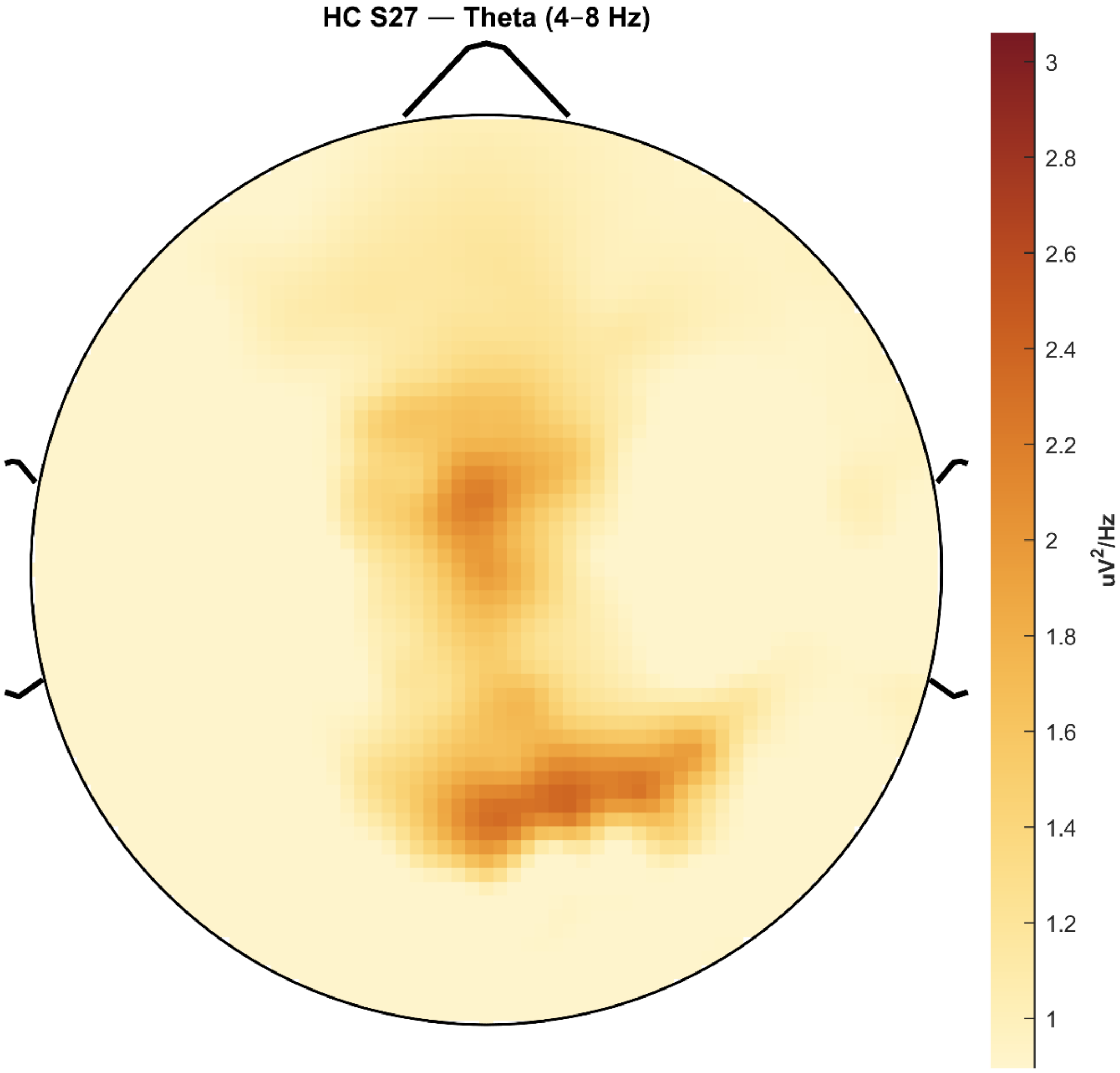

Supplementary Figure 154

Theta (4-8 Hz) power map for HC subject S27. The color scale represents theta power (µV²/Hz).

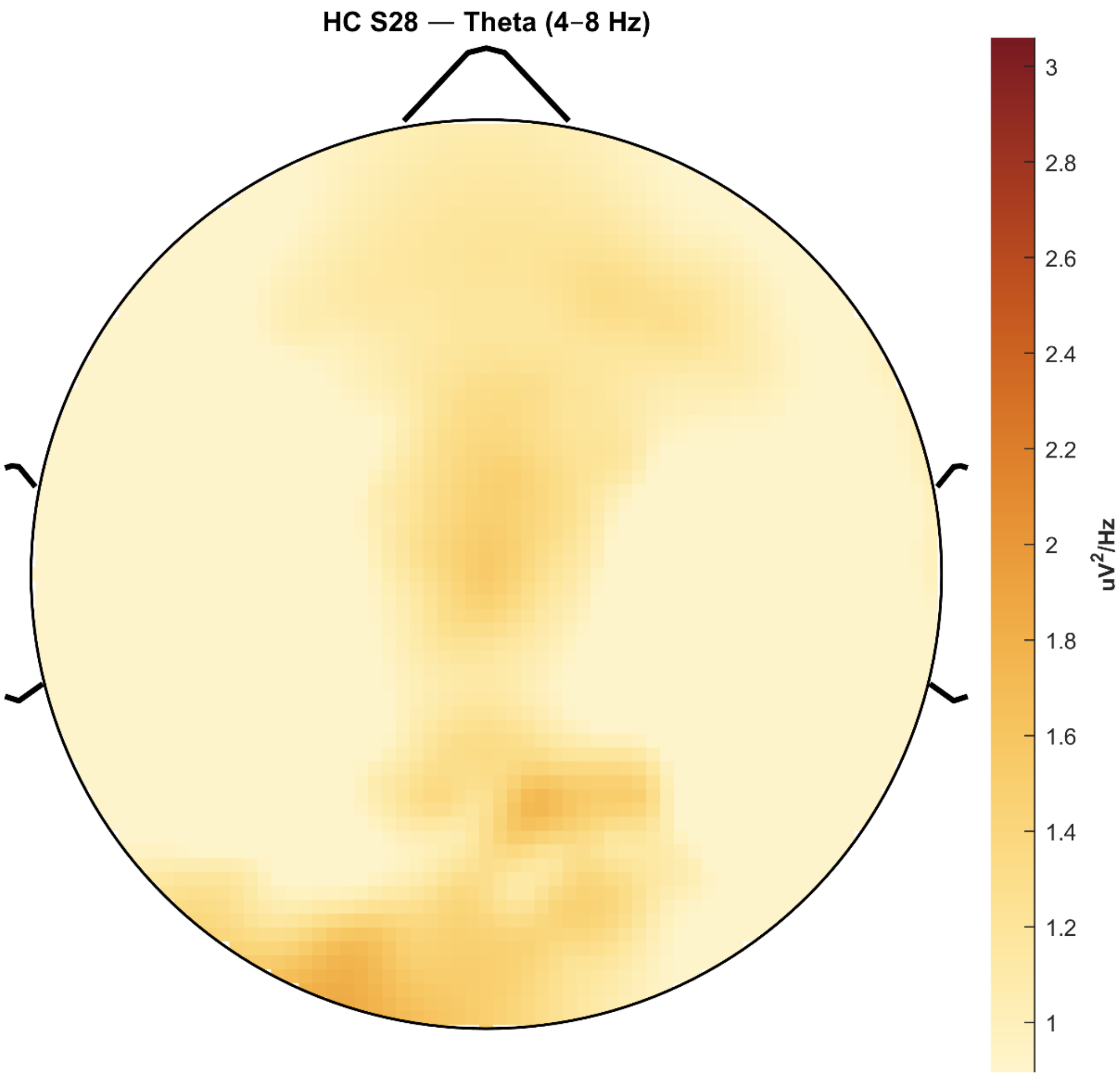

Supplementary Figure 155

Theta (4-8 Hz) power map for HC subject S28. The color scale represents theta power (µV²/Hz).

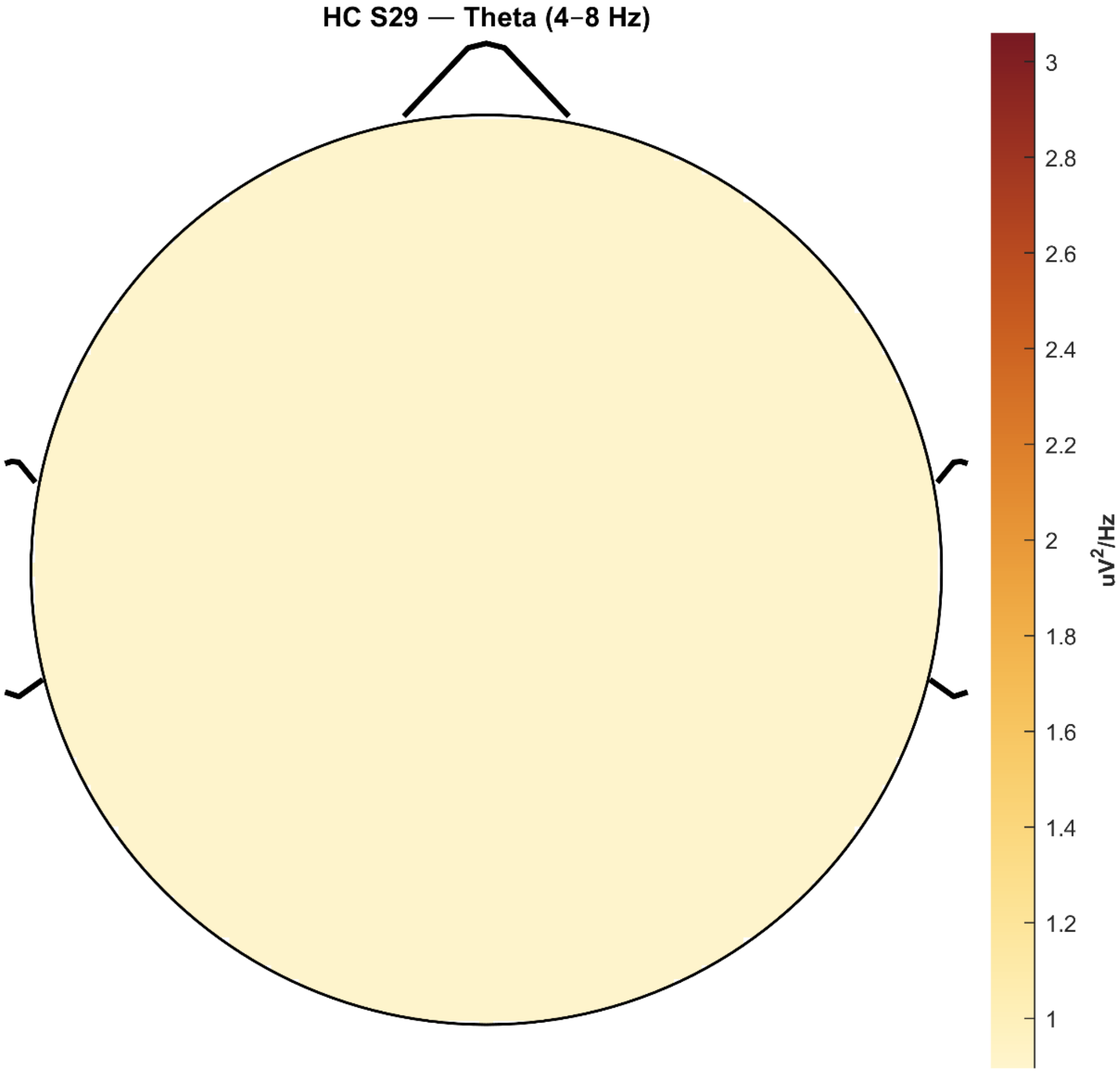

Supplementary Figure 156

Theta (4-8 Hz) power map for HC subject S29. The color scale represents theta power (μV²/Hz).

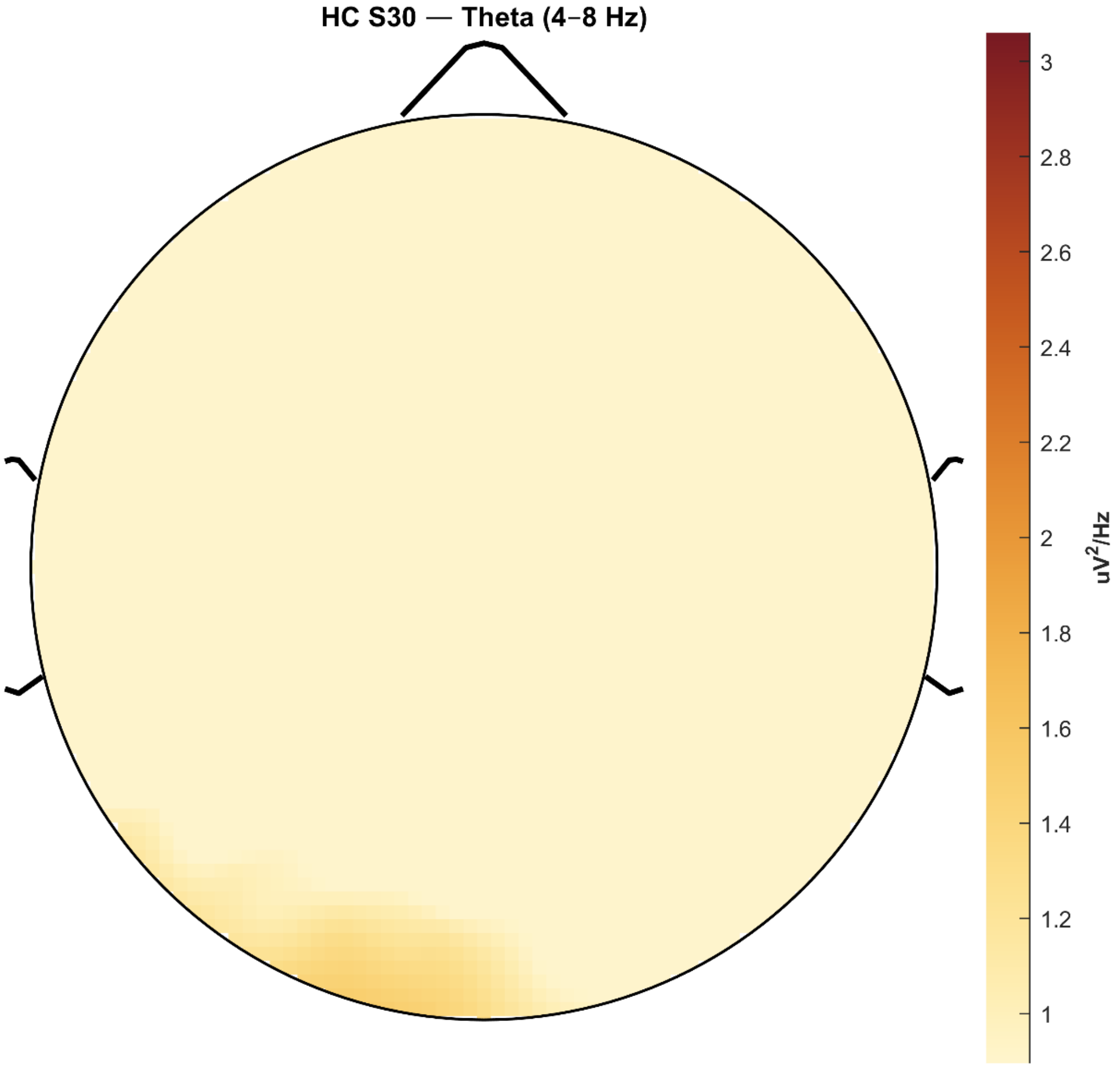

Supplementary Figure 157

Theta (4-8 Hz) power map for HC subject S30. The color scale represents theta power (μV²/Hz).

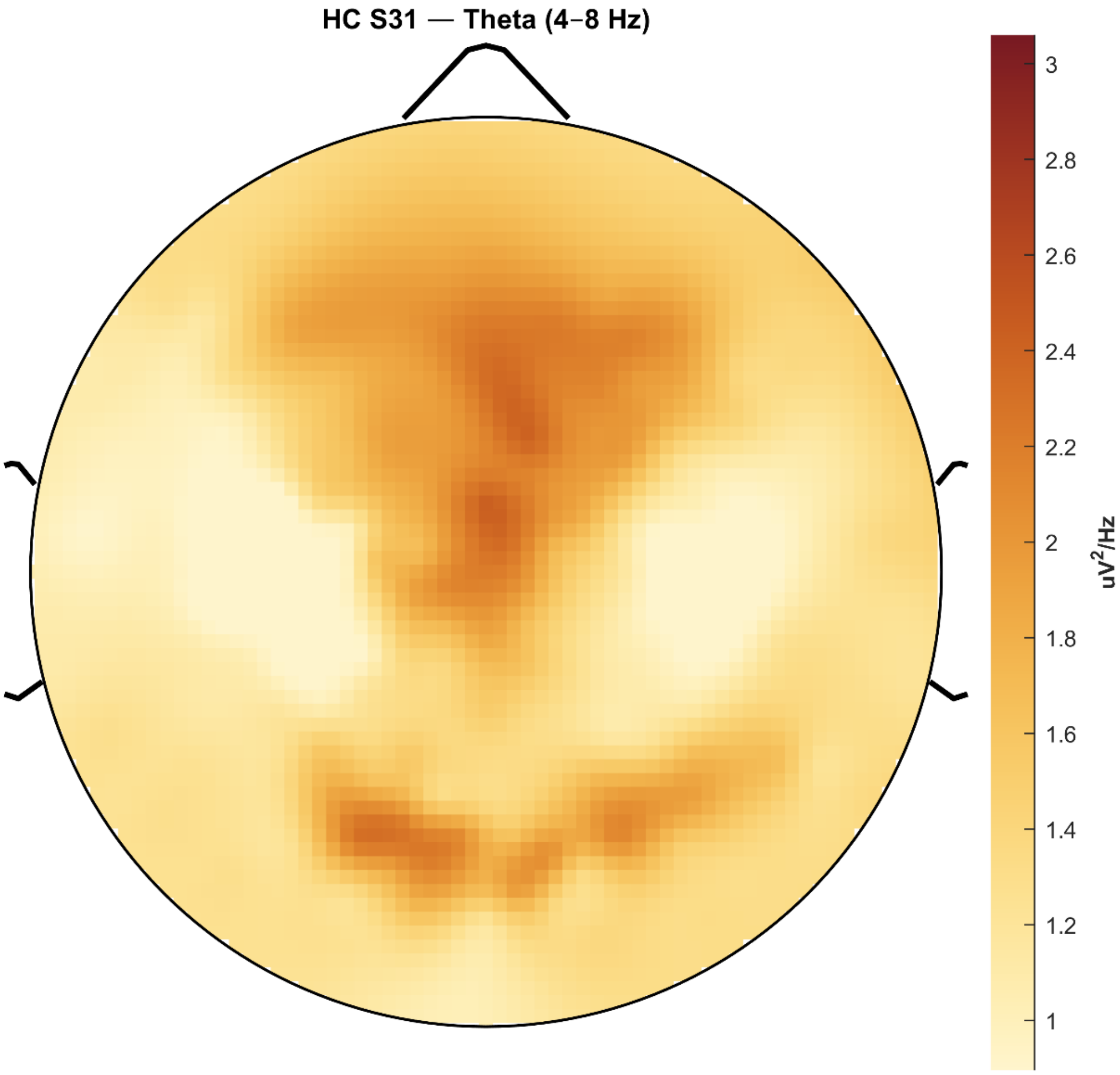

Supplementary Figure 158

Theta (4-8 Hz) power map for HC subject S31. The color scale represents theta power (μV²/Hz).

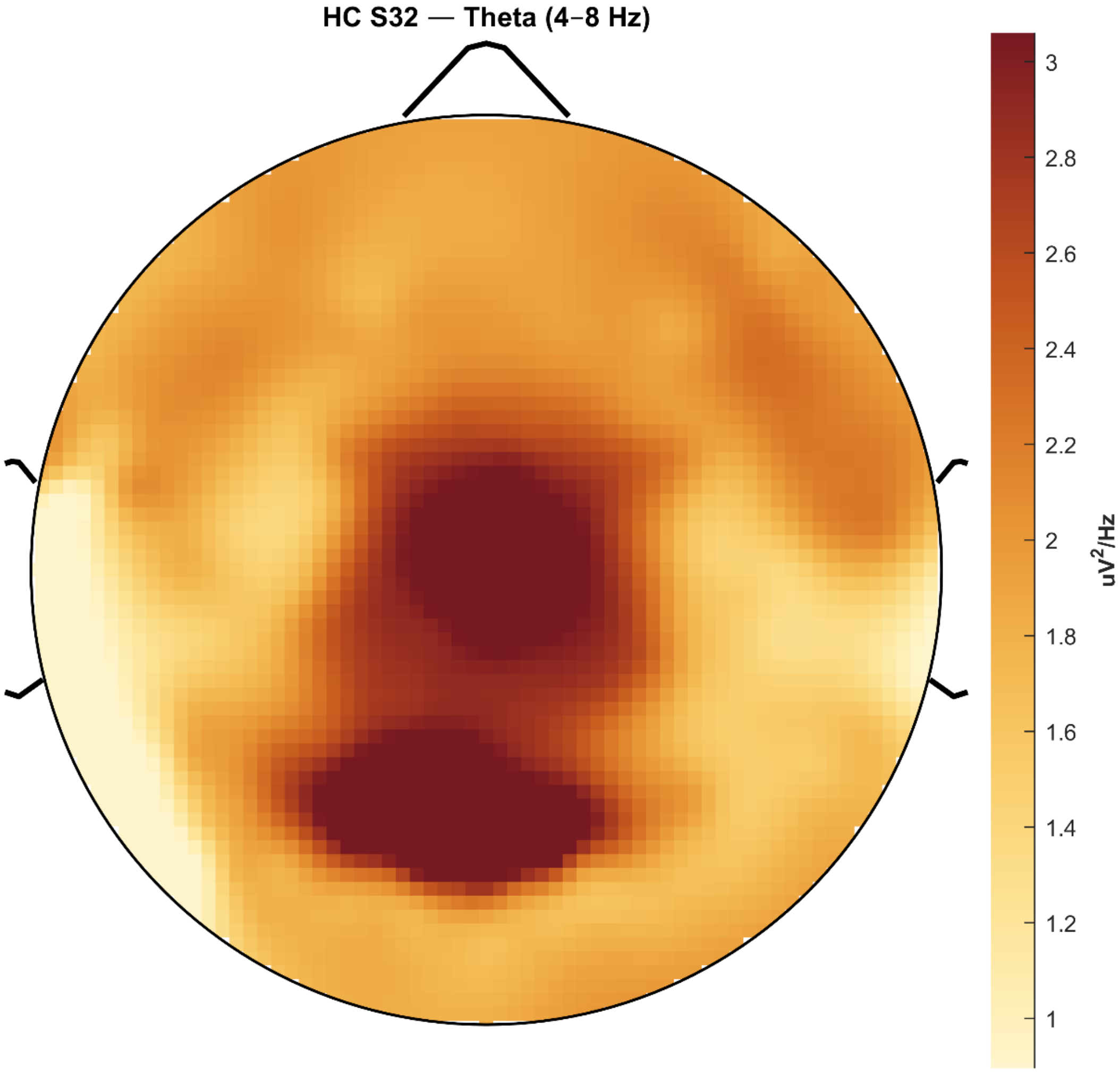

Supplementary Figure 159

Theta (4-8 Hz) power map for HC subject S32. The color scale represents theta power (μV²/Hz).

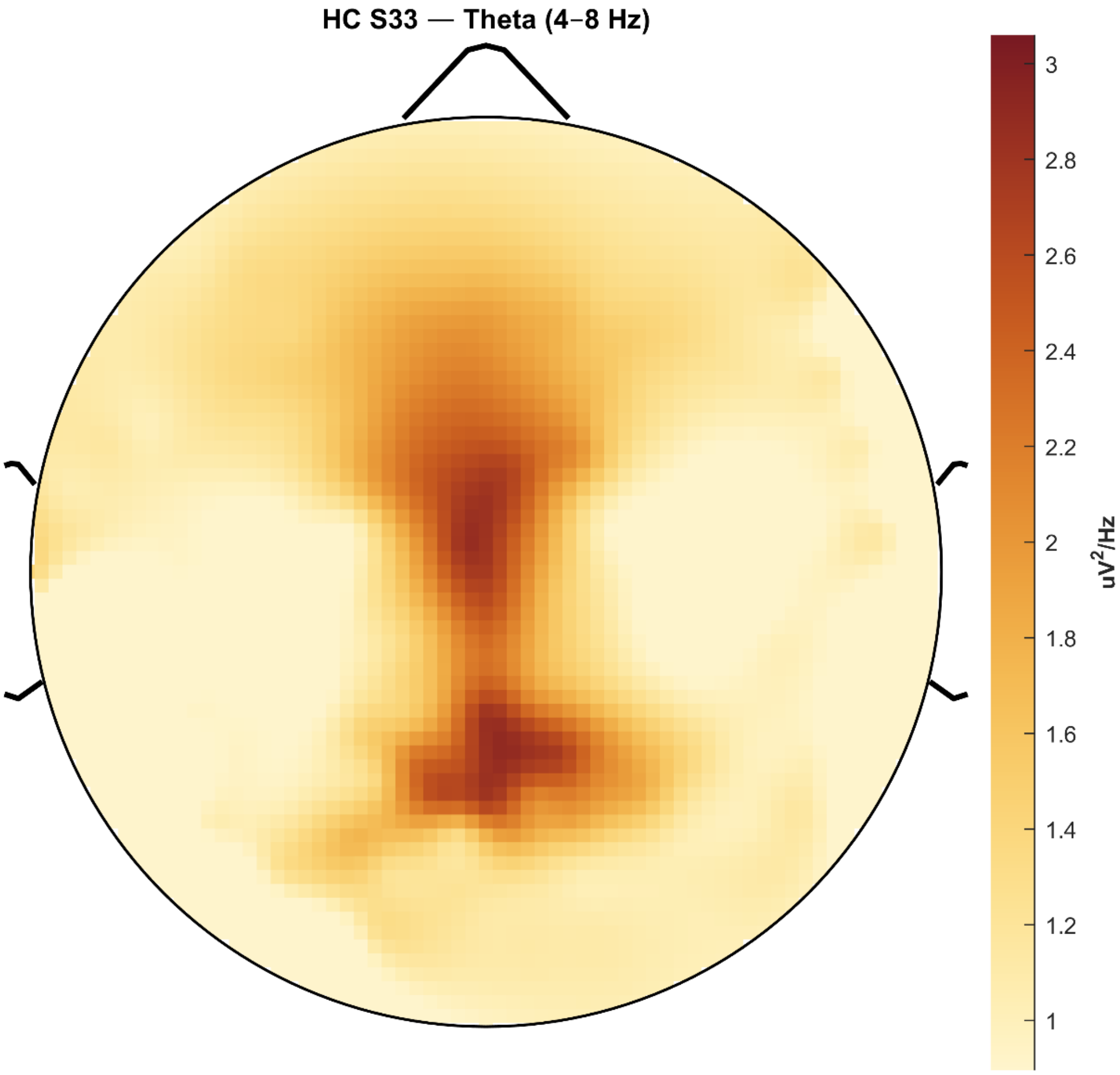

Supplementary Figure 160

Theta (4-8 Hz) power map for HC subject S33. The color scale represents theta power (µV²/Hz).

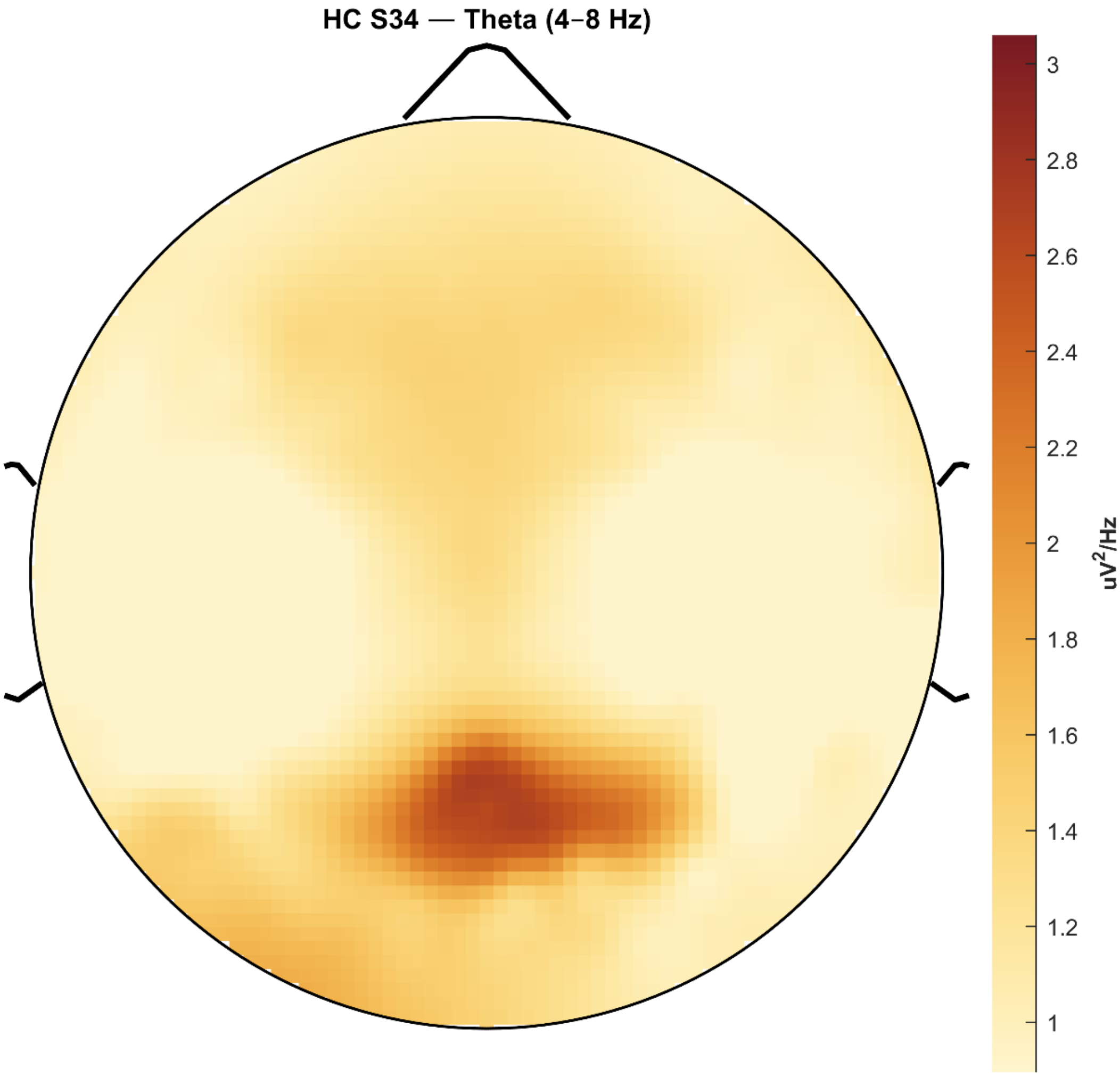

Supplementary Figure 161

Theta (4-8 Hz) power map for HC subject S34. The color scale represents theta power (μV²/Hz).

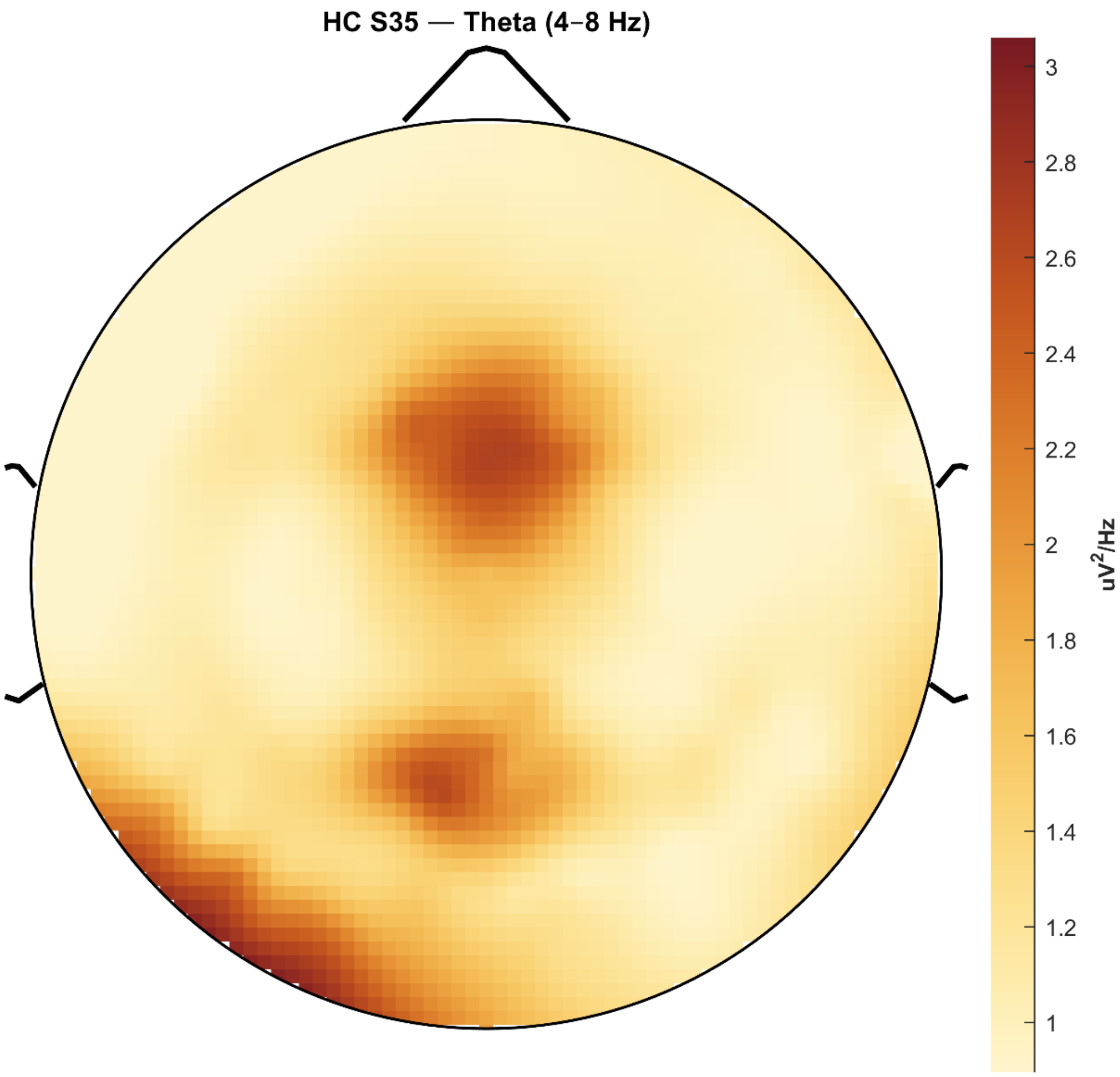

Supplementary Figure 162

Theta (4-8 Hz) power map for HC subject S35. The color scale represents theta power (μV²/Hz).

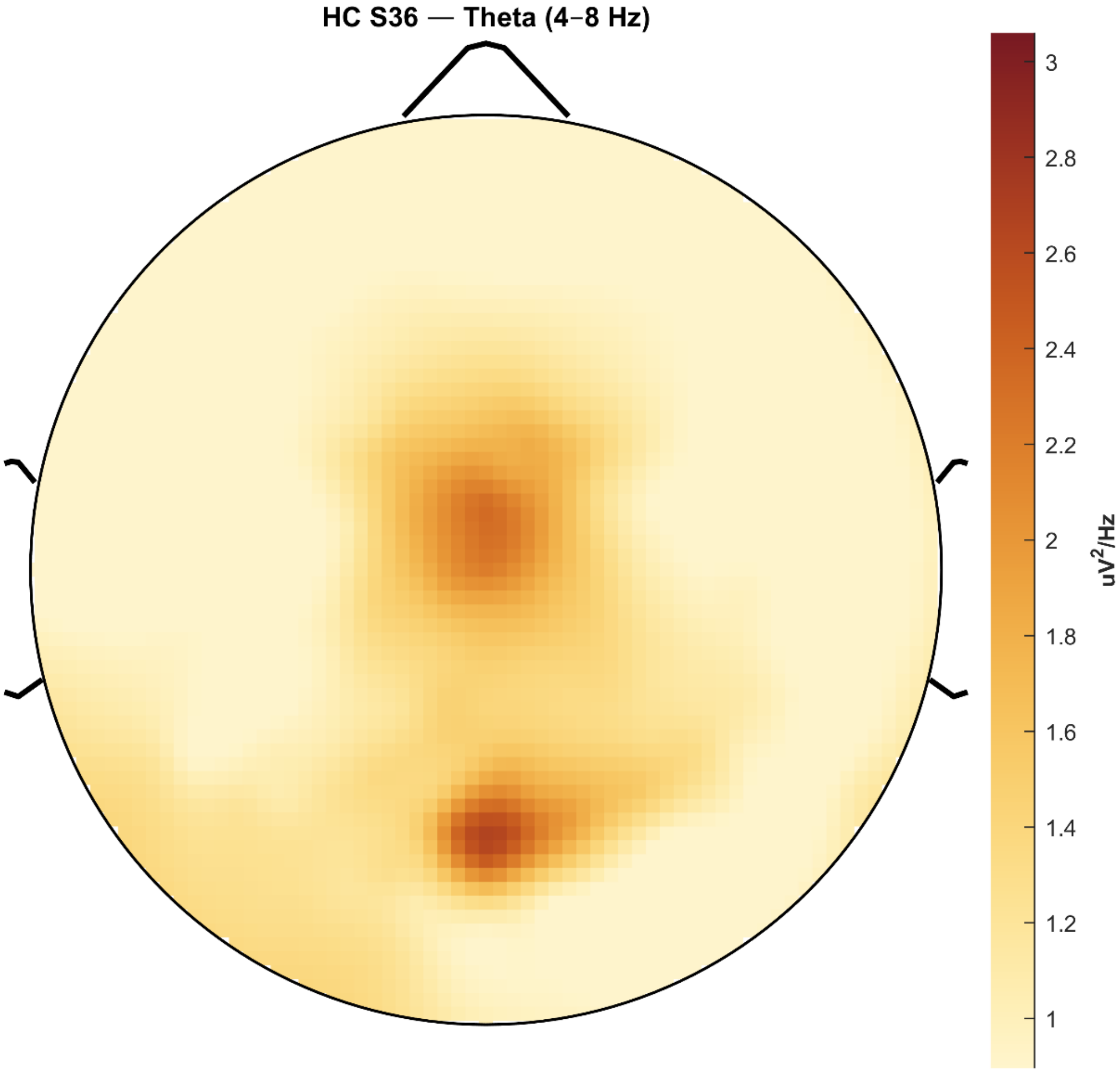

Supplementary Figure 163

Theta (4-8 Hz) power map for HC subject S36. The color scale represents theta power (µV²/Hz).

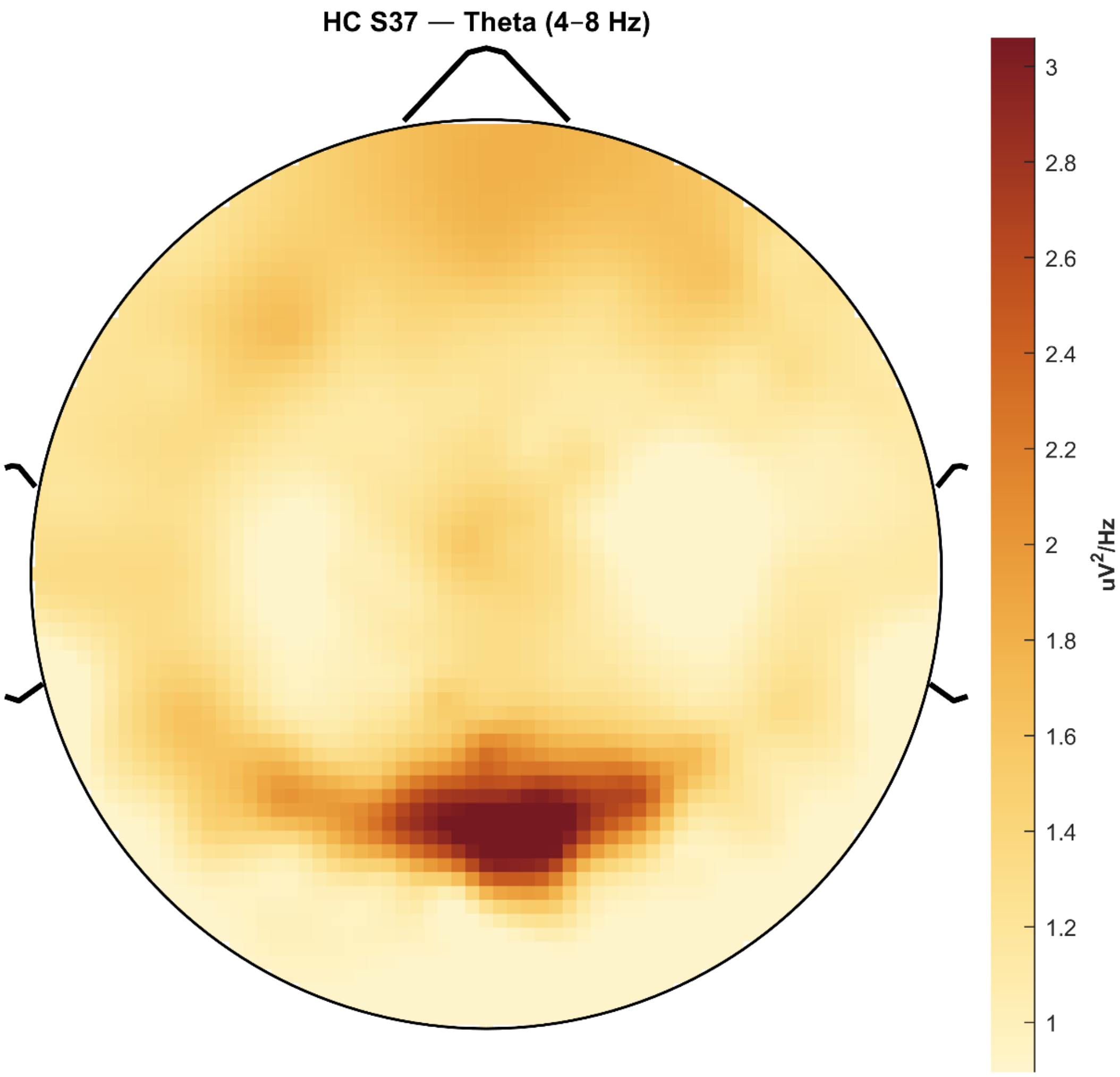

Supplementary Figure 164

Theta (4-8 Hz) power map for HC subject S37. The color scale represents theta power (µV²/Hz).

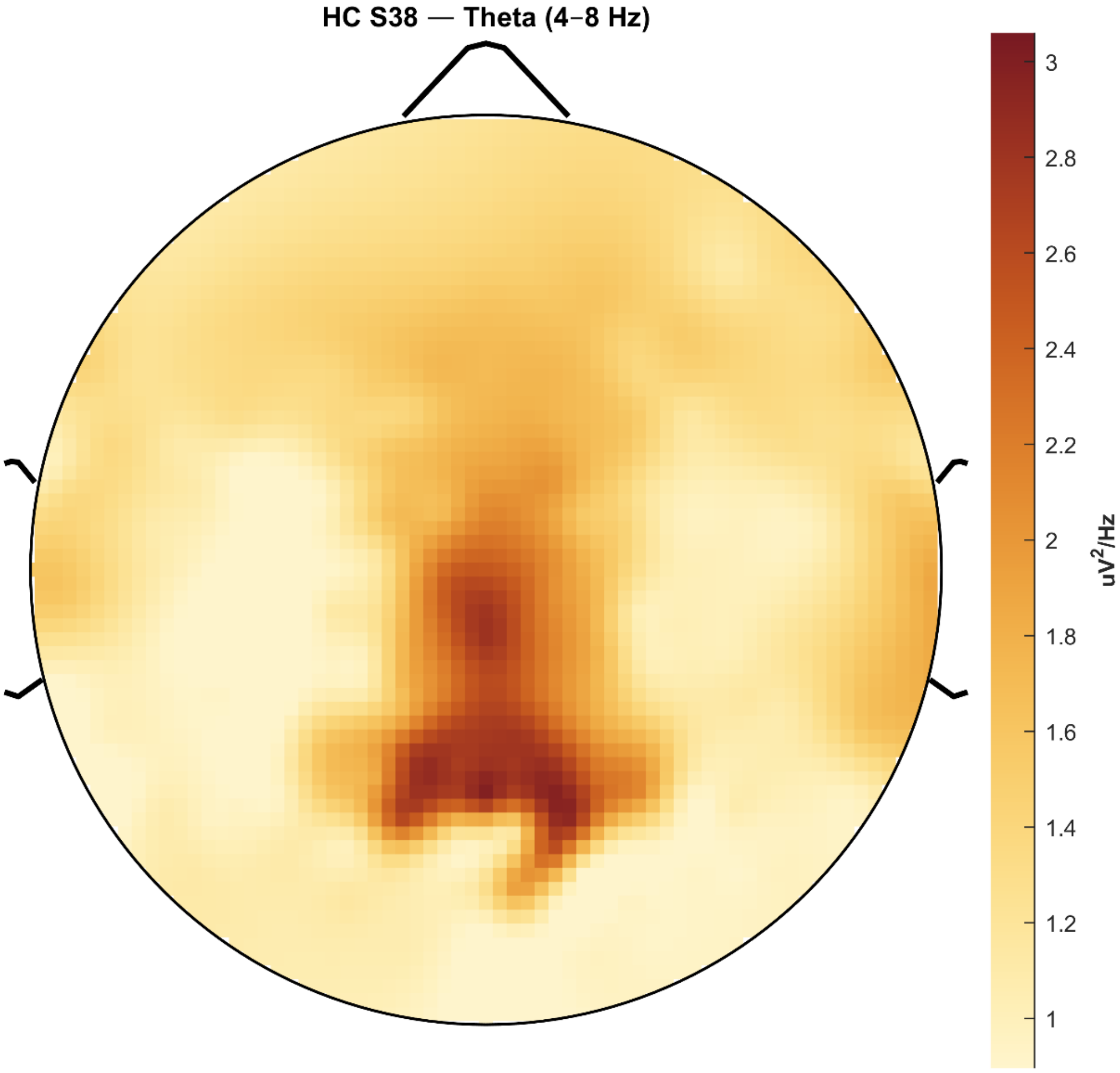

Supplementary Figure 165

Theta (4-8 Hz) power map for HC subject S38. The color scale represents theta power (µV²/Hz).

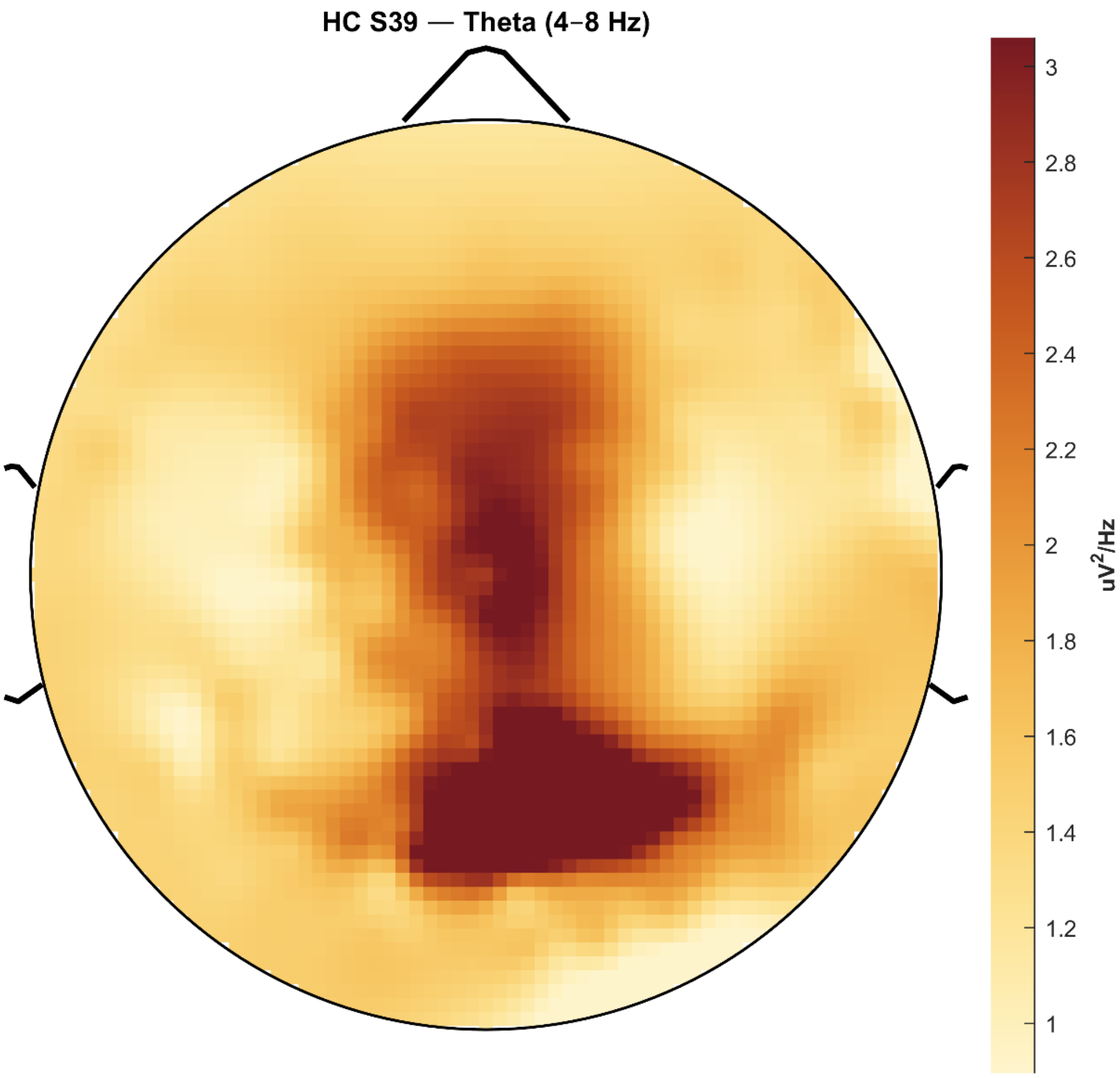

Supplementary Figure 166

Theta (4-8 Hz) power map for HC subject S39. The color scale represents theta power (µV²/Hz).

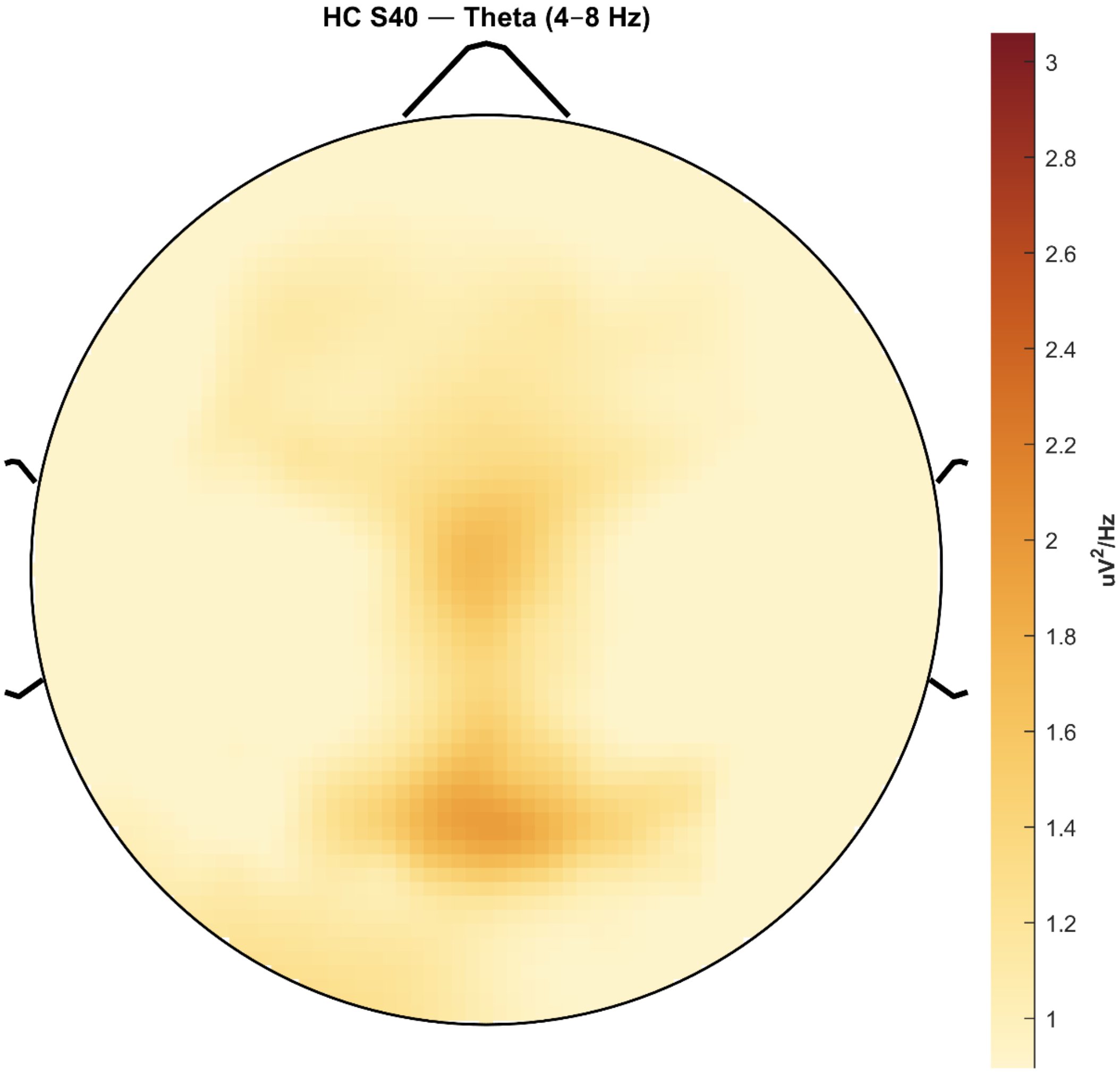

Supplementary Figure 167

Theta (4-8 Hz) power map for HC subject S40. The color scale represents theta power (µV²/Hz).

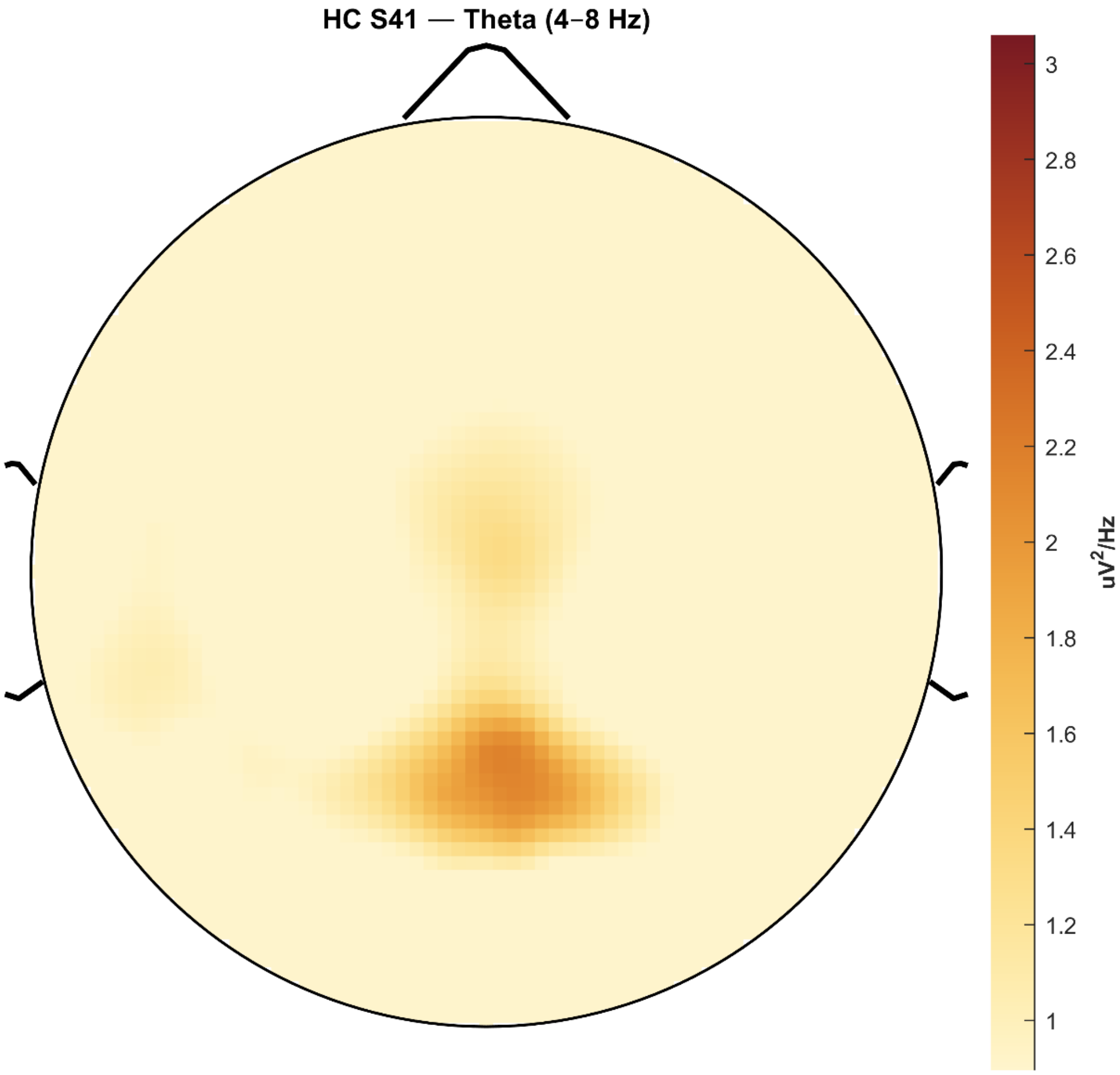

Supplementary Figure 168

Theta (4-8 Hz) power map for HC subject S41. The color scale represents theta power (µV²/Hz).

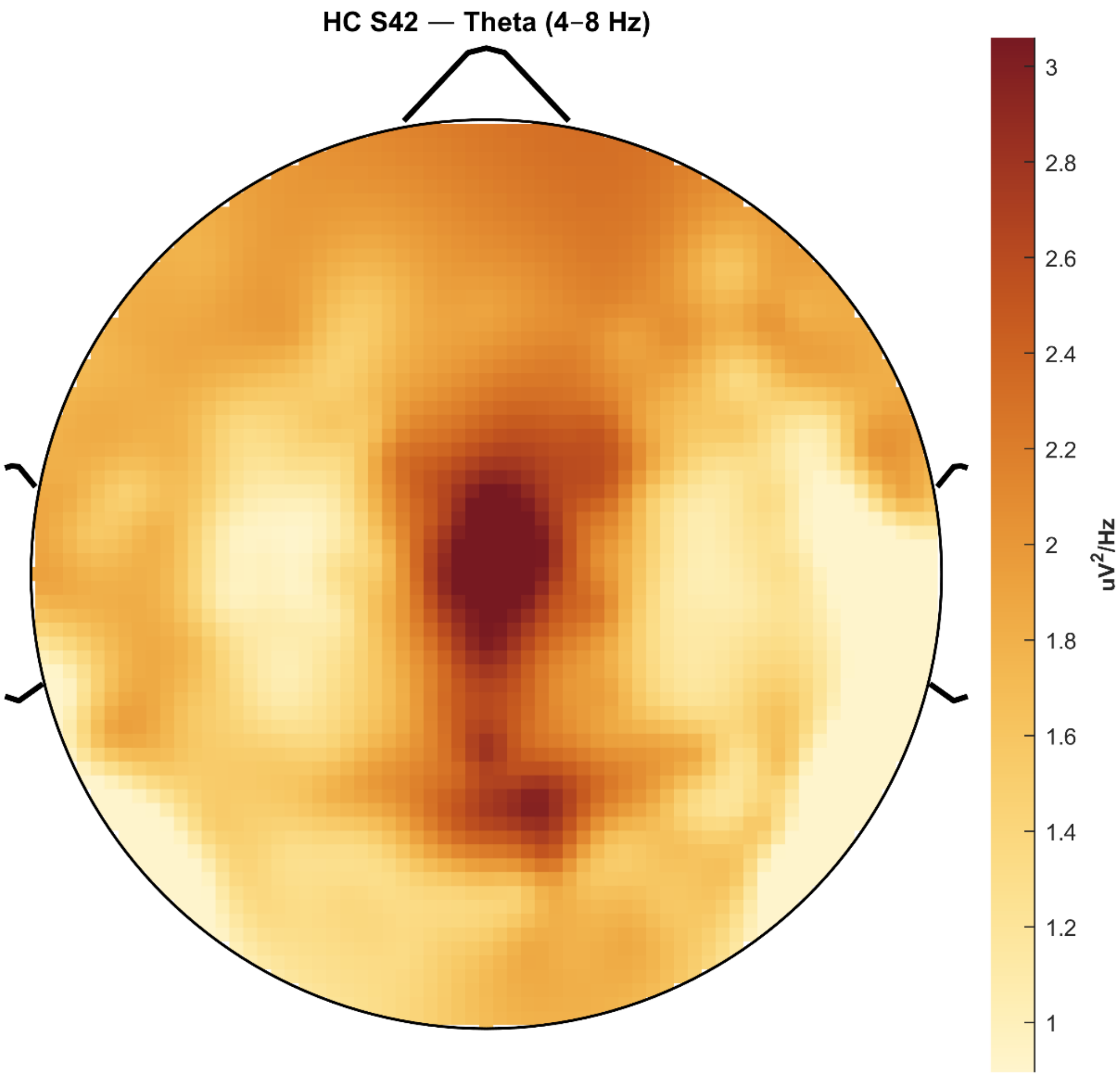

Supplementary Figure 169

Theta (4-8 Hz) power map for HC subject S42. The color scale represents theta power (μV²/Hz).

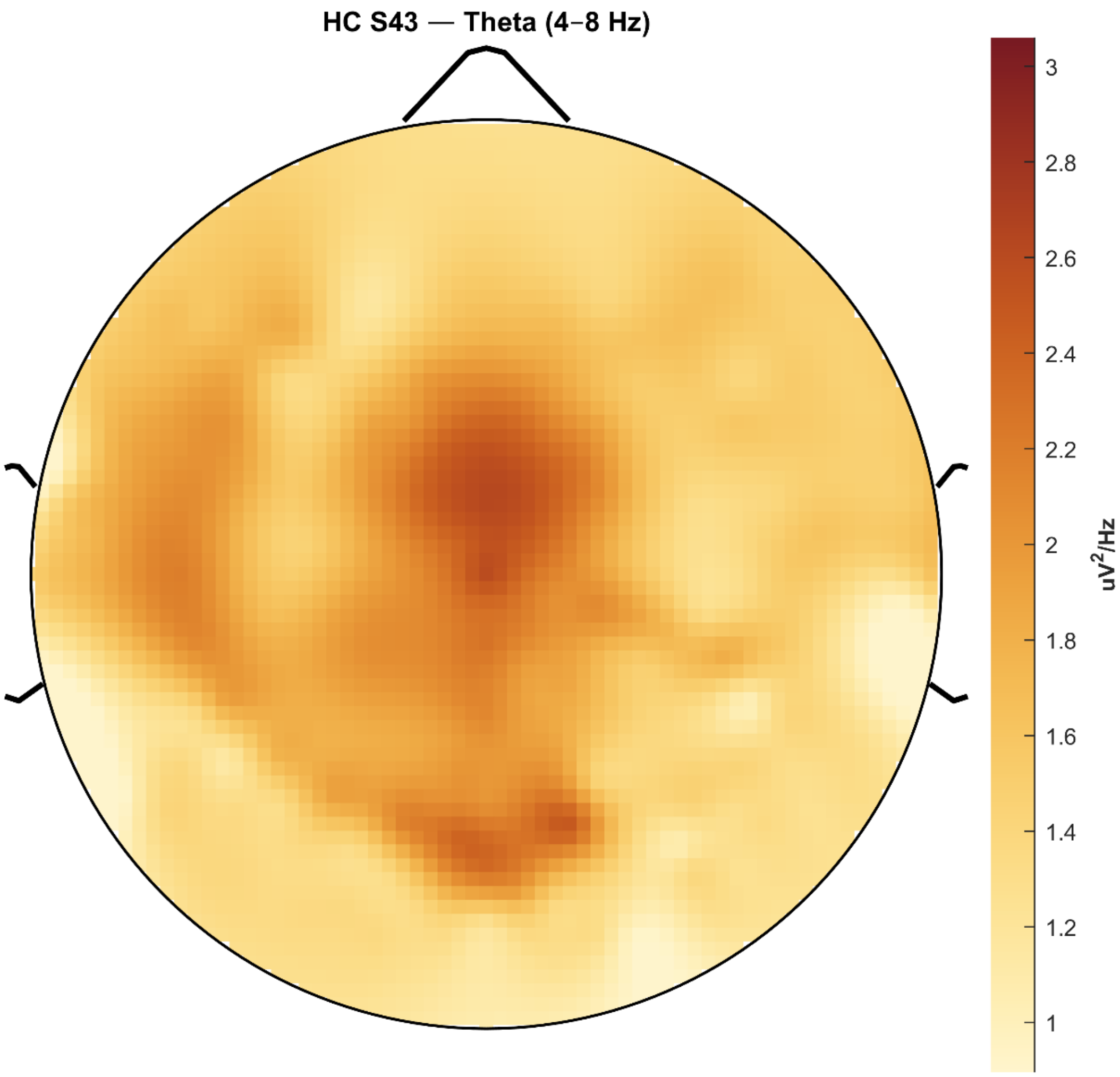

Supplementary Figure 170

Theta (4-8 Hz) power map for HC subject S43. The color scale represents theta power (µV²/Hz).

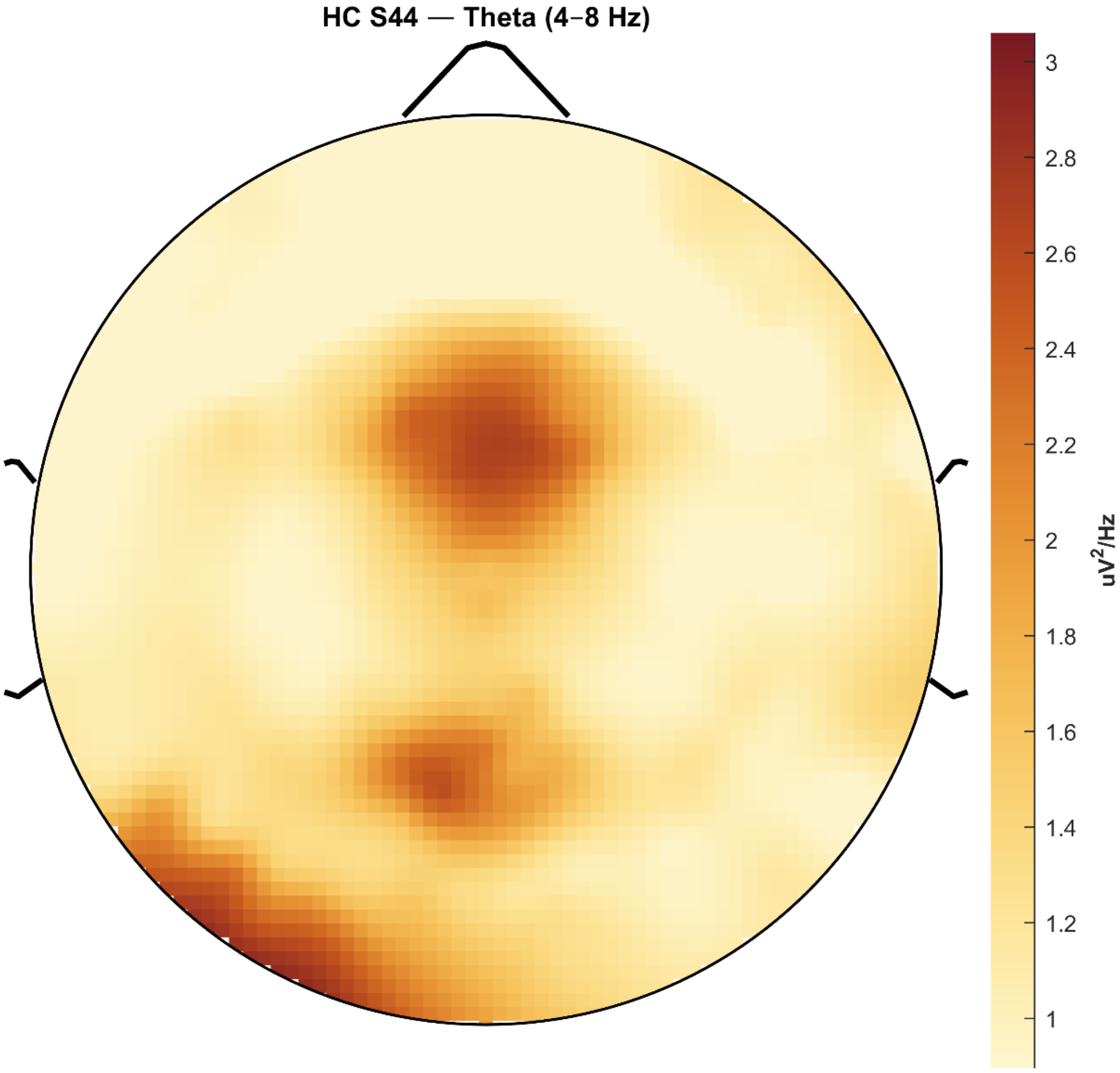

Supplementary Figure 171

Theta (4-8 Hz) power map for HC subject S44. The color scale represents theta power (μV²/Hz).

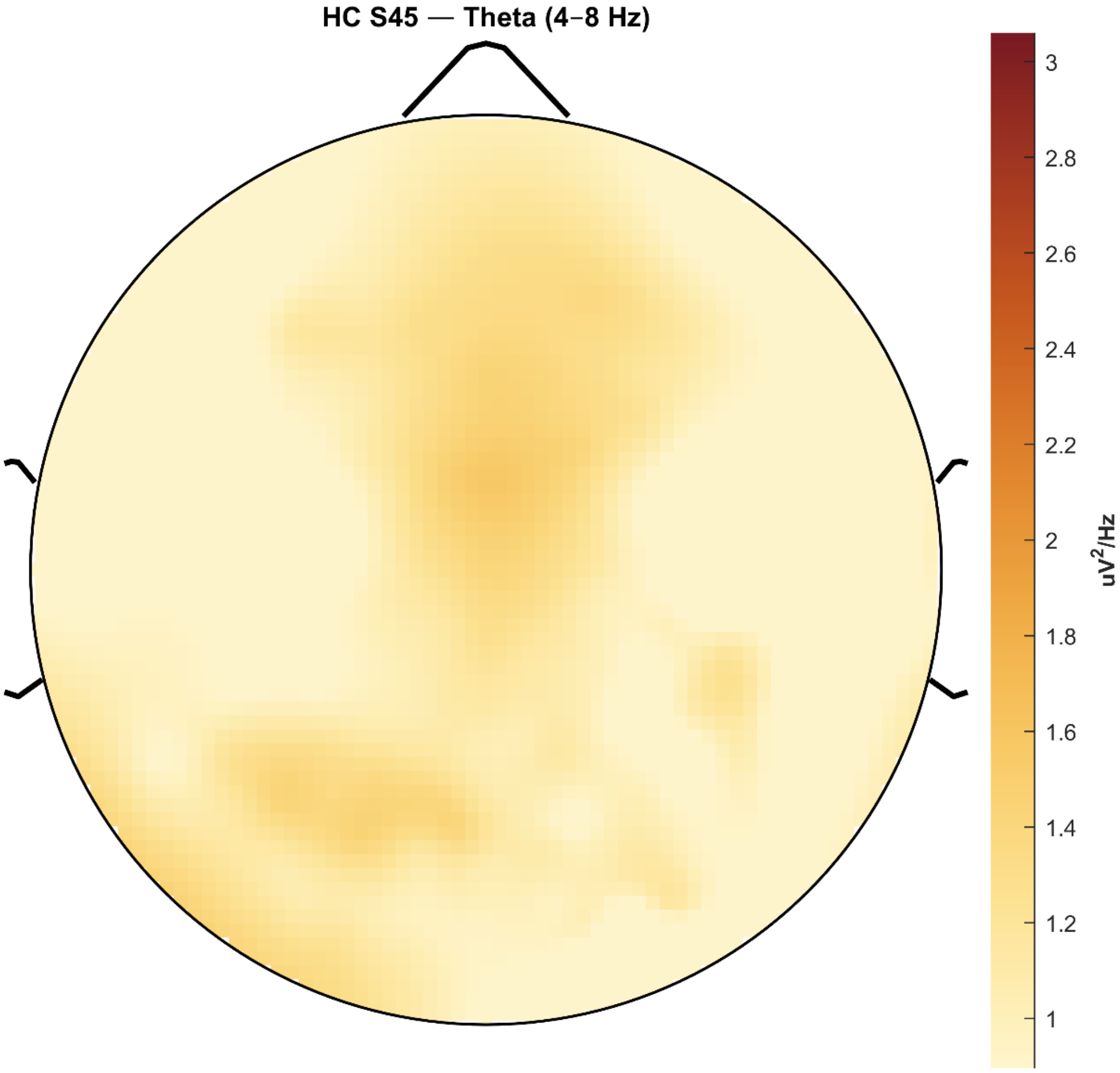

Supplementary Figure 172

Theta (4-8 Hz) power map for HC subject S45. The color scale represents theta power (μV²/Hz).

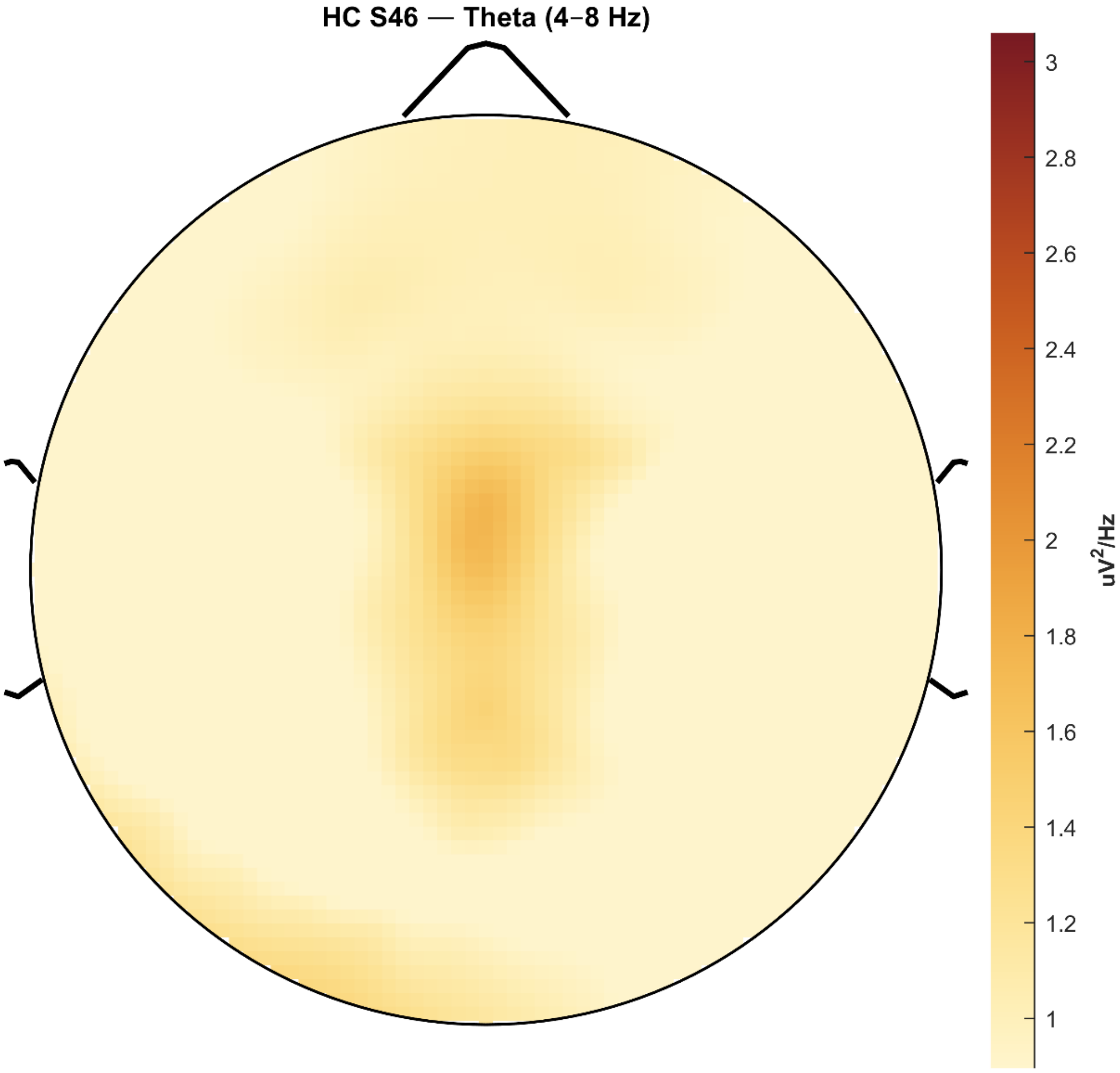

Supplementary Figure 173

Theta (4-8 Hz) power map for HC subject S46. The color scale represents theta power (μV²/Hz).

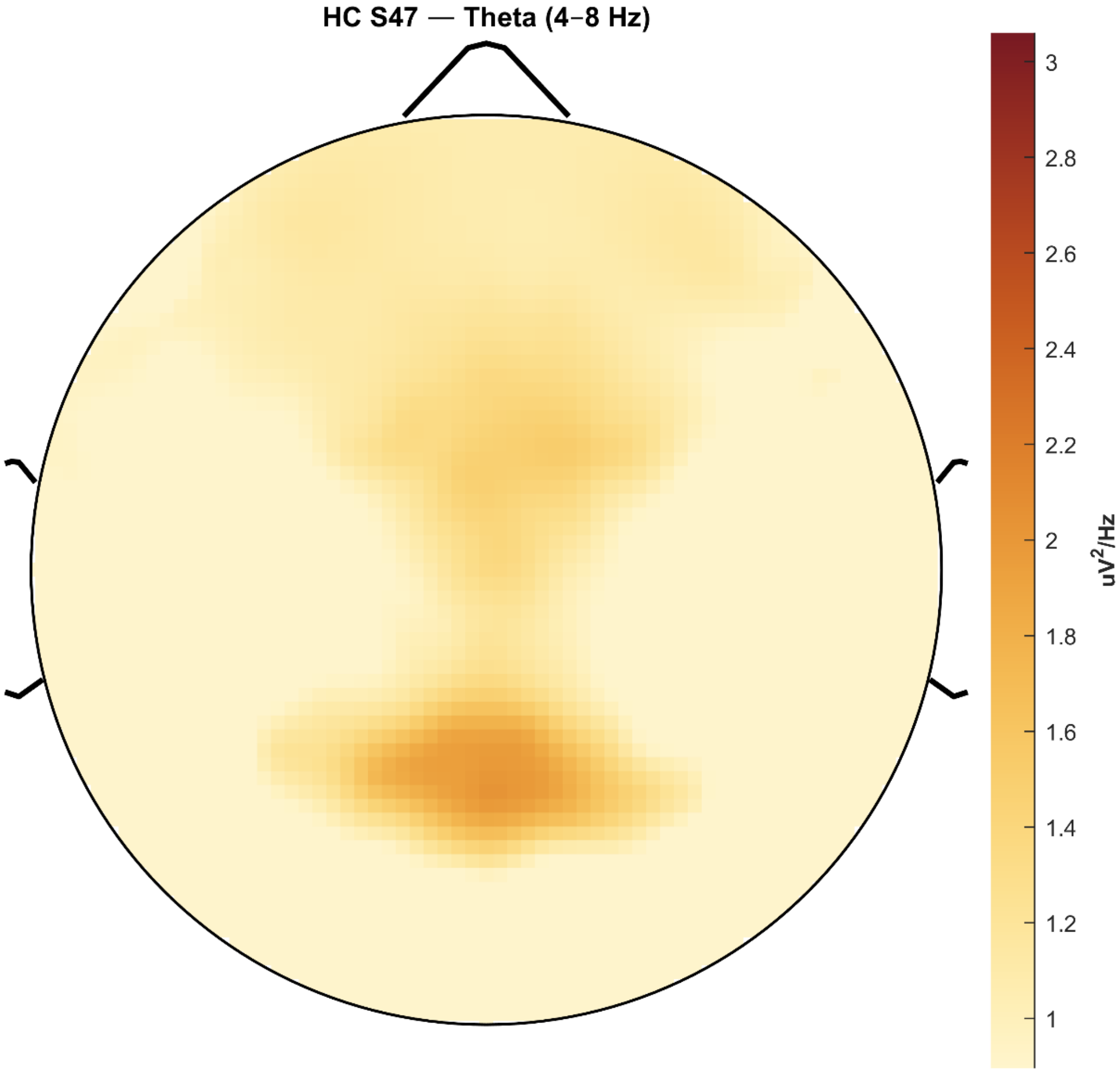

Supplementary Figure 174

Theta (4-8 Hz) power map for HC subject S47. The color scale represents theta power (μV²/Hz).

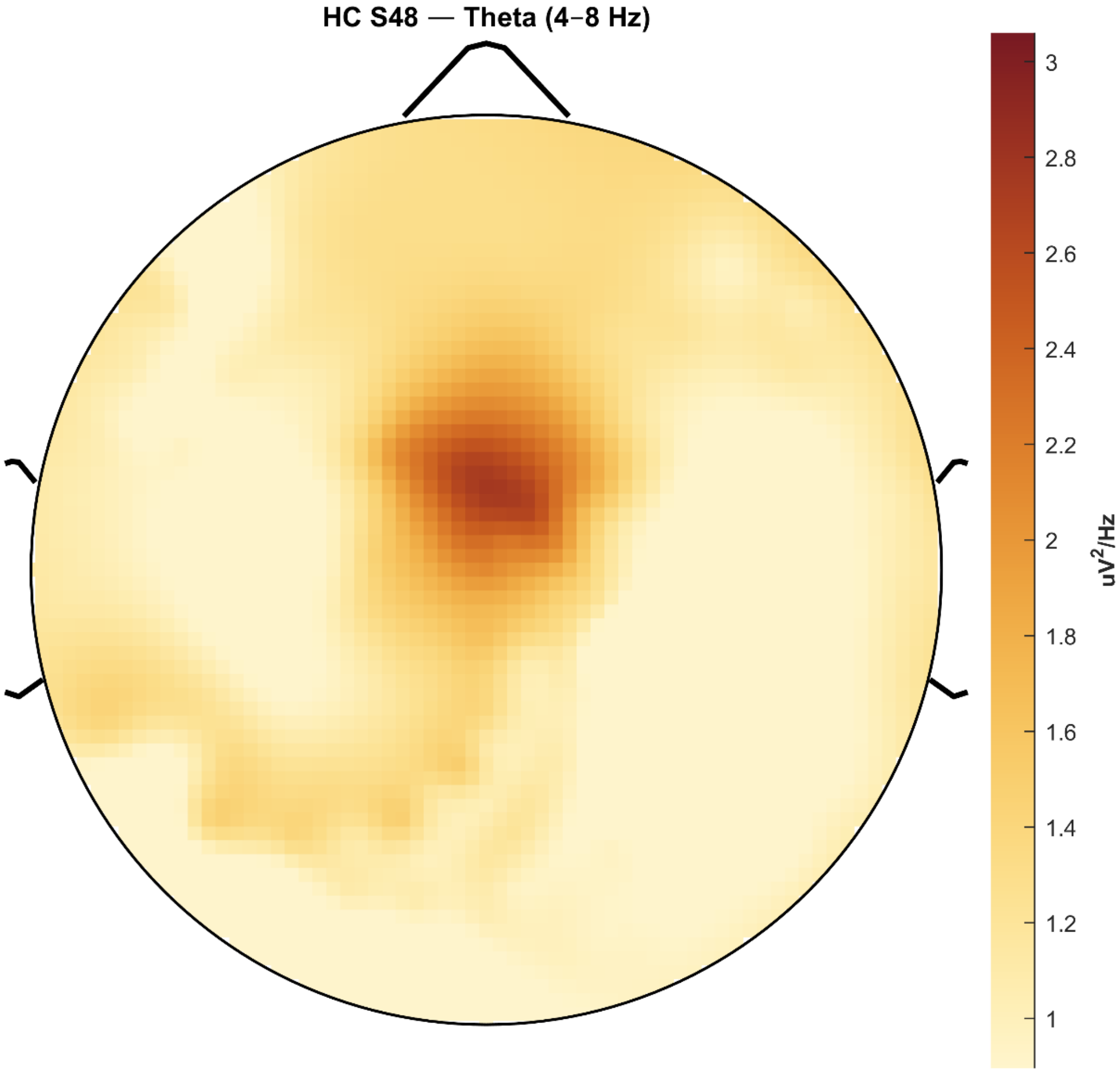

Supplementary Figure 175

Theta (4-8 Hz) power map for HC subject S48. The color scale represents theta power (μV²/Hz).
